# A comparative study of the efficacy of NAXOZOL compared to celecoxib in patients with osteoarthritis

**DOI:** 10.1371/journal.pone.0226184

**Published:** 2020-01-27

**Authors:** Moon Soo Park, Chang-Nam Kang, Woo-Suk Lee, Ho-Joong Kim, Sahnghoon Lee, Jin Hwan Kim, Sang-Jin Shin, Seong-Hwan Moon

**Affiliations:** 1 Department of Orthopaedic Surgery, Medical College of Hallym University, Gyeonggi-do, Republic of Korea; 2 Department of Orthopedic Surgery, Hanyang University College of Medicine, Seoul, Republic of Korea; 3 Department of Orthopedic Surgery, Gangnam Severance Hospital, Seoul, Republic of Korea; 4 Spine Center and Department of Orthopaedic Surgery, Seoul National University Bundang Hospital, Seoul National University College of Medicine, Gyeonggi-do, Republic of Korea; 5 Department of Orthopaedic Surgery, Seoul National University, Seoul National University College of Medicine, Seoul, Republic of Korea; 6 Department of Orthopaedic Surgery, Ilsan Paik Hospital, Inje University College of Medicine, Gyeonggi-do, Republic of Korea; 7 Department of Orthopaedic Surgery, Mokdong Hospital, Ewha Womans University College of Medicine, Seoul, Republic of Korea; 8 Department of Orthopaedic Surgery, Yonsei University College of Medicine, Seoul, Republic of Korea; Fondazione Toscana Gabriele Monasterio, ITALY

## Abstract

**Objective:**

Selective cyclooxygenase-2 inhibitors (celecoxib) can minimize the gastrointestinal complications related to non-steroidal anti-inflammatory drug (NSAID) use. NAXOZOL is a new combination formulation designed to provide sequential delivery of a non-enteric-coated, immediate-release esomeprazole strontium tetrahydrate 20 mg mantle followed by an enteric-coated naproxen 500 mg core. However, there have been no studies comparing NAXOZOL to celecoxib with respect to gastrointestinal tract protection and pain relief in patients with osteoarthritis. This study was undertaken to compare the effects of NAXOZOL and celecoxib with respect to gastrointestinal tract protection and pain relief in patients with osteoarthritis.

**Methods:**

The randomized enrolled patients were divided into two treatment groups: a NAXOZOL group and a celecoxib group. All participants received treatments (NAXOZOL, 500/20 mg (naproxen 500 mg, esomeprazole strontium tetrahydrate 20 mg) twice per day *versus* celecoxib, 200 mg daily) on a 1:1 allocation basis for 12 weeks. The primary outcome was the Leeds Dyspepsia Questionnaire (LDQ) score used for non-inferiority testing. Secondary outcome measures included the Gastrointestinal Symptom Rating Scale (GSRS) score, Visual Analogue Scale (VAS) score, European Quality of Life-5 dimensions (EQ-5D) scale and the EQ-5D Visual Analogue Scale (EQ VAS). Other outcome measures included the use of supplementary or rescue drugs, and the incidence of adverse events.

**Results:**

The baseline-adjusted LDQ scores immediately after 12 weeks of treatment in NAXOZOL group were not inferior to those in celecoxib group. The overall change in the baseline-adjusted GSRS score, VAS score, EQ-5D, and EQ VAS was not different between the two groups. The usage of supplementary drugs and the drug-related incidence of adverse events were not different. However, the days to use rescue drug were longer in celecoxib group than in NAXOZOL group.

**Conclusion:**

NAXOZOL was not inferior to celecoxib in protecting the gastrointestinal tract and providing pain relief in patients with osteoarthritis.

## Introduction

Dyspepsia related to the use of a nonsteroidal anti-inflammatory drug (NSAID) is common, and occurs in about 25–30% of NSAID users [1, 2]. Efforts have been made to reduce the rate of gastrointestinal complications related to the use of NSAIDs. Selective cyclooxygenase-2 inhibitors (COX-2 inhibitors such as celecoxib) can minimize gastrointestinal complications related to the use of NSAIDs [3, 4]. In addition, dyspepsia can be relieved with proton pump inhibitors (PPIs), and PPIs are more effective at relieving dyspepsia than ranitidine and misoprostol [5, 6]. According to the guidelines of the American College of Gastroenterology, patients with a moderate risk of gastrointestinal bleeding are recommended to take PPIs whenever they take NSAIDs [7].

A combination of enteric-coated naproxen 500 mg and immediate-release esomeprazole magnesium 20 mg has been designed to provide sequential delivery of an NSAID and a PPI in a single tablet [8]. This combination formulation demonstrated comparable analgesic efficacy in the treatment of osteoarthritis over a 3-month period [9]. There was no difference in dyspepsia compared to selective COX-2 inhibitors [10], and the incidence of endoscopic gastric ulcers in patients at risk of NSAID-associated ulceration was significantly reduced over a 6-month observation period [11]. In patients at risk of upper gastrointestinal complications who required NSAID therapy, treatment for more than one year with this combination formulation was not associated with any safety issues or adverse effects in the upper gastrointestinal tract or the cardiovascular system [12].

NAXOZOL is the new combination formulation designed to provide sequential delivery of a non-enteric-coated, immediate-release esomeprazole strontium tetrahydrate 20 mg mantle followed by an enteric-coated naproxen 500 mg core [13–15]. However, there have been no studies comparing NAXOZOL to celecoxib with respect to gastrointestinal tract protection and pain relief in patients with osteoarthritis. Therefore, the purpose of this study was to compare the efficacies of NAXOZOL and celecoxib as to gastrointestinal tract protection and pain relief in patients with osteoarthritis.

## Material and methods

### Study design

This study was a prospective, double-blind, double-dummy, active-controlled, two-arm parallel, randomized controlled preliminary trial that was designed to compare the effects of NAXOZOL (Hanmi Pharmaceutical Co., Ltd., Seoul, Republic of Korea) to celecoxib with respect to gastrointestinal tract protection and pain relief in individuals with osteoarthritis. The study was approved by the institutional review board of Severance Hospital, Yonsei University, College of Medicine, Seoul, Korea (IRB number: 4-2014-0906). All participants provided written informed consent before study enrollment.

The enrolled patients were divided into two treatment groups: a NAXOZOL group and a celecoxib group. All participants received treatments (NAXOZOL, 500/20 mg (naproxen 500 mg, esomeprazole strontium tetrahydrate 20 mg) twice per day *versus* celecoxib, 200 mg daily) on a 1:1 allocation basis for 12 weeks. The randomization was made by the permuted block randomization method. The permuted block size was 2 by the site. All treatment regimens were administered in a double-blind and double-dummy manner. Both investigator and subject were blinded to study group allocation. Hence, they were blinded as to whether the subject received the experimental drug or the control drug. The investigational products for this study (experimental drug: NAXOZOL tablet; control drug: celecoxib capsule) differed in color and packaging, so this study was a double-placebo study that used a placebo for the experimental drug that is equal in form, shape, and packaging to the experimental drug, and a different placebo for the comparator that is equal in form, shape, and packaging to the comparator, ensuring that the double-blinding of the investigator and subjects was maintained during the study period. Neither the investigator nor the subject could be aware of whether the experimental drug or the control drug was administered to the subject until the termination of the study. A follow-up period of 12 weeks was determined based on data obtained from previous studies on dyspepsia [16, 17]. This study is registered at ClinicalTrials.gov (number: NCT02355236). The authors confirm that all ongoing and related trials for this drug/intervention are registered.

### Eligibility criteria

The inclusion criteria were as follows: (1) age ≥ 50 years old, (2) symptomatic osteoarthritis diagnosed on radiographs, with a visual analog scale (VAS) score > 4 for pain. Diagnosis of osteoarthritis was made based on history, clinical examination and radiographic changes [18]. The typical changes seen on radiographs were joint space narrowing, subchondral sclerosis), subchondral cyst formation, and osteophytes. The exclusion criteria were: any history of gastrointestinal ulcer including gastrointestinal bleeding, perforation, penetration, gastric outlet obstruction, or gastrointestinal cancer; gastroesophageal reflux; gastrointestinal infection with Helicobacter; a diagnosis of septic arthritis, rheumatic arthritis, gout, Paget’s disease, acromegaly, or Ehlers Danlos Syndrome; any history of joint surgery for the osteoarthritis; pregnancy, or the possibility of pregnancy in women who did not agree to use proper contraception; heavy alcohol intake; allergy to NSAIDs or PPIs; severe liver disease (equal to or greater than Child-Pugh Class II); chronic kidney disease (creatinine clearance < 30 mL/min); heart disease with a history of coronary artery bypass graft; uncontrolled hypertension; enrollment in other clinical trials within the prior 1 month; any concurrent serious medical condition such as sepsis or cancer that would cause disability or compromise the patients’ general health status.

### Efficacy assessments

Baseline data collected by a blinded clinical research assistant included sex, birth date, height, weight, smoking status, status of alcohol drinking, and medication use. The primary outcome was the Leeds Dyspepsia Questionnaire (LDQ) score immediately after 12 weeks of treatment. The LDQ was based on a self-administered questionnaire that measured “gastrointestinal symptoms” [19]. It is a Likert scale that evaluates gastrointestinal symptoms including: indigestion (pain in the upper abdomen), heartburn (burning sensation behind the sternum), a sensation of food stuck in the throat, regurgitation (an acid taste in the mouth from stomach contents), burping or belching, nausea, vomiting, and excessive fullness. Secondary outcome measures included the Gastrointestinal Symptom Rating Scale (GSRS) score, Visual Analogue Scale (VAS) score for osteoarthritis, European Quality of life-5 Dimensions (EQ-5D) scale, and EQ-5D Visual Analogue Scale (EQ VAS). The GSRS was based on a self-administered questionnaire with 15 questions measuring the “gastrointestinal symptoms of gastroesophageal reflux, functional dyspepsia and irritable bowel syndrome” [20]. The VAS for arthritic pain comprised a 10-cm line with “none” (0) on one end of the scale and “disabling pain” (10) on the other [21]. On the EQ-5D scale, scores range from 0 to 1, with 1 indicating perfect health [22]. The EQ VAS is the second part of the EQ-5D questionnaire, with instructions that state to mark the current health status on a 20-cm vertical scale, from 0 ("the worst health you can imagine") to 100 ("the best health you can imagine"). The LDQ score, GSRS score, VAS score, EQ-5D scale, and EQ VAS were assessed at enrollment and at 12 weeks after treatment.

Other outcome measures included the use of supplementary or rescue drugs, and the incidence of adverse events. The patients that had severe dyspepsia during the study were provided the supplementary drug (almagate 500 mg) to alleviate their symptoms. The patients that had severe arthritis pain that was uncontrolled with our treatment regimens were provided the rescue drug (acetaminophen 650 mg) to alleviate their symptoms. The investigator monitored for adverse events during each visit for all participants. Adverse events were recorded in detail irrespective of causality or association with the investigational drug. The type and severity of adverse events, date, and the investigator’s opinion regarding any associations between the drug and the adverse events were recorded.

### Statistical analysis

Using data from previous studies [23, 24], we calculated that a minimum sample of 53 participants per group would be required for the current study, using a non-inferior design, based on an alpha of 0.025, power of 0.80, a minimal clinically important difference (margin of non-inferiority) of 0.40, and a follow-up loss rate of 20%.

All efficacy data were analyzed based on the full analysis set and the per protocol set. The supplementary and rescue drug usage data were analyzed based on the safety analysis, full analysis, and per protocol sets.

Participants’ baseline characteristics and drug compliance were compared using the Chi-square test, Fisher’s exact test, independent two-sample t-test, or Wilcoxon rank sum test according to the normal distribution of the study population. LDQ was evaluated from “No” to “Very severe” of symptom level for eight gastrointestinal symptoms. Each symptom level was converted as follows: 0: “No”, 1: “Very mild”, 2: “Mild”, 3: “Moderate”, 4: “Severe”, 5: “Very severe”. The LDQ score was calculated as the total sum of eight symptoms and used for the analysis. The baseline-adjusted LDQ score was defined as the difference between the LDQ scores at the initial and final visits. The baseline-adjusted LDQ scores at 12 weeks after treatment were compared between the study group and the control group using a non-inferiority test (one-sided 95% confidence interval (CI), the margin of non-inferiority 0.40). The alpha level of significance for the non-inferiority test was set at 0.025. Secondary outcome measures, including the baseline-adjusted GSRS score, VAS score, EQ-5D scale, EQ VAS, use of the supplementary drug, and use of the rescue drug were assessed for superiority between the two groups. According to the normal distribution of the study population, an independent two-sample t-test or Wilcoxon rank sum test was performed to examine the secondary outcome measures between the two groups. The alpha level of significance for the other statistical test was set at 0.05. All statistical analyses were performed using SAS 9.3(SAS Institute, Cary, NC, USA).

## Results

### Characteristics of the subjects

**Fig 1:**
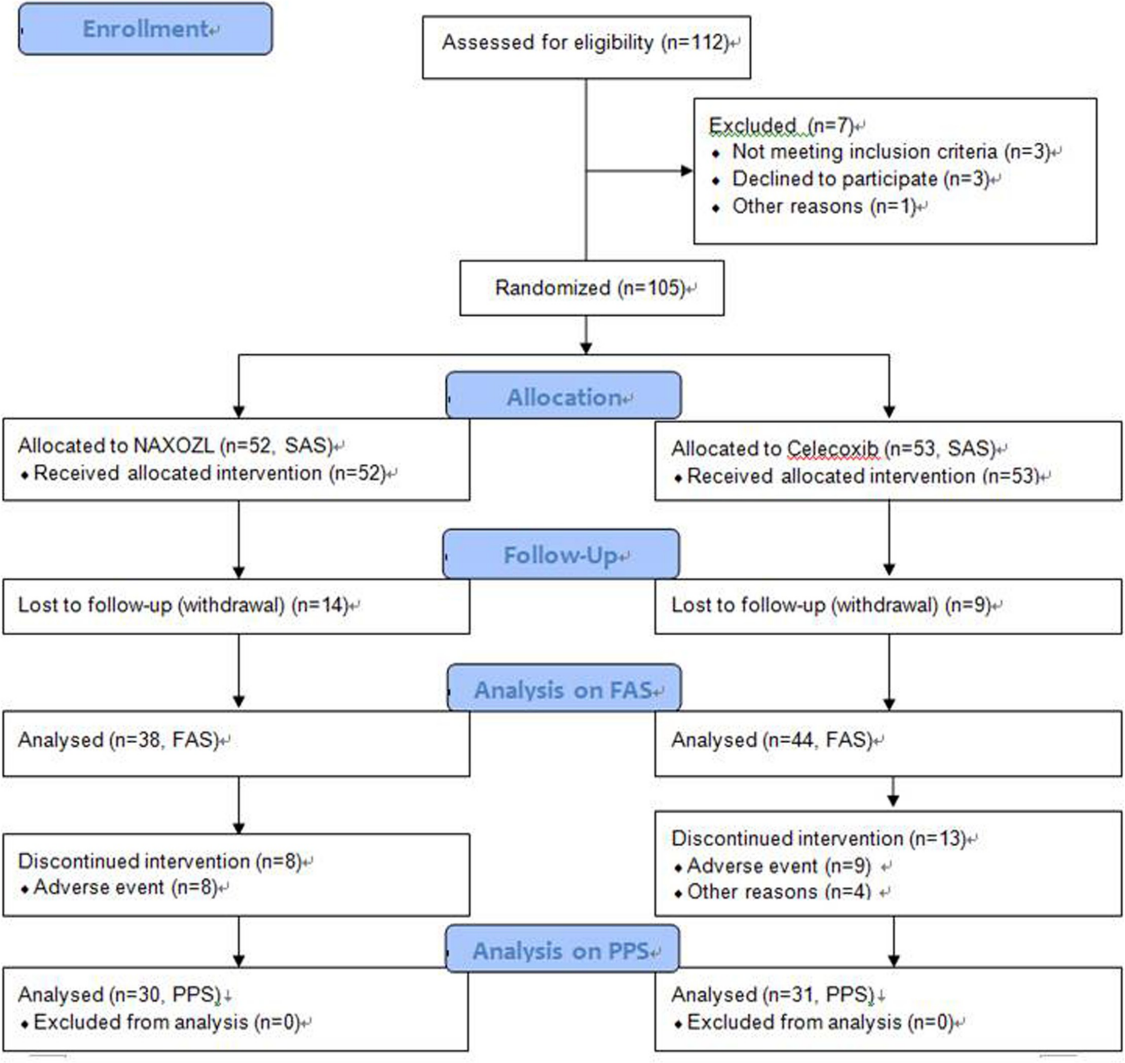
CONSORT flow diagram: enrollment, randomization, assigned interventions, and follow-up of the study participants showing the safety analysis set (SAS), full analysis set (FAS), and per protocol set (PPS).

Participants were enrolled between March 2015 and April 2017. A total of 112 patients were assessed for eligibility for the study. Finally, 105 patients met the inclusion criteria and were randomly assigned to one of the study groups (52 and 53 patients in the NAXOZOL and celecoxib group, respectively). Fig 1 shows the number of subjects involved in the current trial, from the eligibility assessment through the 12-week follow-up. At the 12-week assessment, complete data were available for 61 of the 105 participants (58.0%), which was different from our initial expectation of the rate of follow-up loss. However, the number of complete enrolled participants was sufficient to analyze the non-inferiority between the groups with the LDQ scores as a primary outcome.

There was no difference in participant baseline characteristics between the two groups (Table 1). The mean ages of patients in the NAXOZOL and celecoxib groups were 65.44±8.49 and 66.09±7.16 years, respectively. There was no statistically significant difference between the two groups in sex, height, weight, smoking history, status of alcohol drinking, and the pathophysiology and sites of osteoarthritis. The most common etiology of osteoarthritis was primary or idiopathic and the most common affected site was the spine in the both groups.

**Table 1:** Baseline characteristics of participants in the study.

	NAXOZOL (n = 52)	Celecoxib (n = 53)	P value
Age, years	65.44±8.49	66.09±7.16	0.6711
Women, n (%)	39 (75.00)	35 (66.04)	0.3141
Height, cm	157.04±8.24	159.00±8.54	0.2340
Weight, kg	62.06±9.44	63.63±9.87	0.4067
Smoker, n (%)	3 (5.77)	6 (11.32)	0.6486
Alcohol, n (%)	13 (25.00)	13 (24.53)	1.0000
Medication use, n (%)	29 (55.77)	30 (56.60)	0.9313
Osteoarthritis pathophysiology, n (%)			0.9695
Primary or idiopathic	45 (86.54)	46 (86.79)	
Secondary	7 (13.46)	7 (13.21)	
Osteoarthritis site, n (%)			0.3694
Shoulder	3 (5.77)	3 (5.66)	
Hand and wrist	0 (0.00)	0 (0.00)	
Elbow	1 (1.92)	0 (0.00)	
Foot and ankle	1 (1.92)	2 (3.77)	
Knee	11 (21.15)	12 (22.64)	
Knee and spine	1 (1.92)	6 (11.32)	
Spine	35 (67.31)	30 (56.60)	

Values are mean ± standard deviation.

Smoker: smoking on a regular basis for a period exceeding 12 months

Alcoholic drinker: drinking alcohol on a regular basis for a period exceeding 12 months

The rate of missing data and drug compliance were similar between the groups throughout the follow-up period based on an evaluation of the safety analysis, full analysis, and per protocol sets (Table 2).

**Table 2:** Drug compliance between the two groups.

		NAXOZOL	Celecoxib	P value
Safety analysis set	Number	52	53	
	Drug compliance (%)	75.49±33.49	84.70±25.96	0.1482
Full analysis set	Number	38	44	
	Drug compliance (%)	85.72±23.87	86.82±23.02	0.8347
Per protocol set	Number	30	31	
	Drug compliance (%)	94.53±5.47	95.00±5.66	0.7415

Values are mean ± standard deviation.

### Efficacy

The baseline-adjusted LDQ score (primary outcome) at 12 weeks after treatment in the NAXOZOL group was not inferior to those in the celecoxib groups (Tables 3 and 4). At 12 weeks after treatment, the 95% CI of the difference was within the predetermined margin of non-inferiority (LDQ score, 0.40) based on an evaluation of the full analysis set (0.30, Table 3) and the per protocol set (0.11, Table 4). The overall change in baseline-adjusted GSRS scores, VAS scores, the EQ-5D, and the EQ VAS was not different between the two groups (Tables 3 and 4).

**Table 3:** Comparison of the effect estimates between the two groups based on the full analysis set.

		NAXOZOL (n = 38)	Celecoxib (n = 44)	P value	95% CI
LDQ	Initial	0.55±1.11	0.30±0.82		-0.1683, 0.6826
	Final	0.34±1.02	0.23±0.74		-0.2740, 0.5037
	Difference	-0.21±1.23	-0.07±0.73	[-∞, 0.30]	-0.5806, 0.2959
GSRS	Initial	0.24±0.59	0.34±1.03		-0.4818, 0.2736
	Final	0.24±0.59	0.16±0.48		-0.1573, 0.3128
	Difference	0.00±0.66	-0.18±1.06	0.9464	-0.2140, 0.5777
VAS	Initial	59.76±15.16	58.73±14.72		-5.5409, 7.6126
	Final	39.05±24.63	38.66±21.76		-9.7994, 10.5865
	Difference	-20.71±22.43	-20.07±20.89	0.8936	-10.1676, 8.8829
EQ-5D	Initial	8.32±1.34	8.18±1.21		-0.4251, 0.6931
	Final	7.34±1.83	7.43±1.52		-0.8261, 0.6467
	Difference	-0.97±1.40	-0.75±1.35	0.6278	-0.8296, 0.3822
EQ VAS	Initial	61.55±17.41	61.34±14.02		-6.6984, 7.1218
	Final	69.08±19.79	63.34±16.24		-2.1814, 13.6575
	Difference	7.53±16.68	2.00±14.90	0.1170	-1.4143, 12.4669

Values are mean ± standard deviation.

95% CI; 95% confidence interval of differences between NAXOZOL and Celecoxib groups.

Leeds Dyspepsia Questionnaire (LDQ); Gastrointestinal Symptom Rating Scale (GSRS)

Visual Analogue Scale (VAS); European Quality of Life-5 dimensions scale (EQ-5D); European Quality of Life-5 dimensions scale Visual Analogue Scale (EQ VAS)

**Table 4:** Comparison of the effect estimates between the two groups based on the per protocol set.

		NAXOZOL (n = 30)	Celecoxib (n = 31)	P value	95% CI
LDQ	Initial	0.60±1.19	0.29±0.69		-0.1878, 0.8071
	Final	0.17±0.65	0.19±0.54		-0.3327, 0.2789
	Difference	-0.43±0.86	-0.10±0.87	[-∞,0.11]	-0.7795, 0.1064
GSRS	Initial	0.23±0.57	0.32±1.14		-0.5522, 0.3737
	Final	0.13±0.43	0.16±0.45		-0.2558, 0.1999
	Difference	-0.10±0.48	-0.16±1.21	0.4010	-0.4147, 0.5373
VAS	Initial	58.90±13.81	58.65±14.60		-7.0311, 7.5408
	Final	34.43±21.04	31.97±16.44		-7.1918, 12.1230
	Difference	-24.47±22.02	-26.68±17.95	0.3262	-8.0639, 12.4854
EQ-5D	Initial	8.47±1.31	8.32±1.08		-0.4683, 0.7564
	Final	7.23±1.76	7.32±1.64		-0.9595, 0.7810
	Difference	-1.23±1.38	-1.00±1.18	0.6740	-0.8917, 0.4250
EQ VAS	Initial	62.97±14.19	60.29±14.24		-4.6083, 9.9610
	Final	71.83±16.94	63.35±17.14		-0.2555, 17.2125
	Difference	8.87±16.70	3.06±15.15	0.1602	-2.3613, 13.9656

Values are mean ± standard deviation.

95% CI; 95% confidence interval of differences between NAXOZOL and Celecoxib groups.

Leeds Dyspepsia Questionnaire (LDQ); Gastrointestinal Symptom Rating Scale (GSRS); Visual Analogue Scale (VAS); European Quality of Life-5 dimensions scale (EQ-5D)

European Quality of Life-5 dimensions scale Visual Analogue Scale (EQ VAS)

The use of the supplementary drug was not different between the two groups (Table 5).

**Table 5:** Comparison of the use of supplementary drug between the two groups.

Safety analysis set	NAXOZOL (n = 52)	Celecoxib (n = 53)	P value
Number of patients using, n (%)	8 (15.38)	14 (26.42)	0.1650
Days of use	8.00±10.13	15.21±12.59	0.1825
Average amount during study period	0.26±0.37	0.19±0.14	0.6146
Full analysis set	NAXOZOL (n = 38)	Celecoxib (n = 44)	P value
Number of patients using, n (%)	8 (21.05)	13 (29.55)	0.3796
Days of use	8.00±10.13	15.31±13.10	0.1944
Average amount during study period	0.26±0.37	0.18±0.14	0.5776
Per protocol set	NAXOZOL (n = 30)	Celecoxib (n = 31)	P value
Number of patients using, n (%)	6 (20.00)	11 (35.48)	0.1775
Days of use	9.67±11.41	17.82±12.68	0.2103
Average amount during study period	0.15±0.15	0.21±0.14	0.4785

Values are mean ± standard deviation.

The most significant parameters for the use of the rescue drug were not different between the two groups (Table 6). However, the days to use the rescue drug were longer in the celecoxib group than in the NAXOZOL group.

**Table 6:** Comparison of the use of the rescue drug between the two groups.

Safety analysis set	NAXOZOL (n = 52)	Celecoxib (n = 53)	P value
Number of patients using, n (%)	15 (28.85)	17 (32.08)	0.7193
Days of use	6.87±6.10	15.47±11.63	**0.0134**
Average amount during study period	0.12±0.10	0.19±0.13	0.1026
Full analysis set	NAXOZOL (n = 38)	Celecoxib (n = 44)	P value
Number of patients using, n (%)	15 (39.47)	16 (36.36)	0.7721
Days of use	6.87±6.10	15.56±12.01	**0.0175**
Average amount during study period	0.12±0.10	0.18±0.14	0.1432
Per protocol set	NAXOZOL (n = 30)	Celecoxib (n = 31)	P value
Number of patients using, n (%)	13 (43.33)	12 (38.71)	0.7136
Days of use	7.00±6.47	17.75±12.54	**0.0170**
Average amount during study period	0.12±0.10	0.21±0.14	0.0775

Values are mean ± standard deviation.

A total of 8 and 9 cases of drug-related adverse events occurred in the NAXOZOL and celecoxib groups, respectively (Table 7). There was no statistical difference between the two groups (p = 0.8243, Table 7).

**Table 7:** Summary of adverse events between the two groups based on the safety analysis set (p value = 0.8243).

	NAXOZOL	Celecoxib	Total
	(n = 52)	(n = 53)	(n = 105)
Patient number (%) [event number]	8 (15.38) [14]	9 (16.98) [13]	17 (16.19) [27]
Gastrointestinal disorders	3 (5.77) [3]	1 (1.89) [1]	4 (3.81) [4]
Dyspepsia	2 (3.85) [2]	0 (0.00) [0]	2 (1.90) [2]
Diarrhea	0 (0.00) [0]	1 (1.89) [1]	1 (0.95) [1]
Glossodynia	1 (1.92) [1]	0 (0.00) [0]	1 (0.95) [1]
Infections and infestations	1 (1.92) [1]	3 (5.66) [3]	4 (3.81) [4]
Nasopharyngitis	1 (1.92) [1]	2 (3.77) [2]	3 (2.86) [3]
Bacterial infections NEC	0 (0.00) [0]	1 (1.89) [1]	1 (0.95) [1]
Skin and subcutaneous tissue disorders	0 (0.00) [0]	3 (5.66) [3]	3 (2.86) [3]
Nail fold inflammation	0 (0.00) [0]	1 (1.89) [1]	1 (0.95) [1]
Pruritus	0 (0.00) [0]	1 (1.89) [1]	1 (0.95) [1]
Skin lesion	0 (0.00) [0]	1 (1.89) [1]	1 (0.95) [1]
Investigations	2 (3.85) [2]	0 (0.00) [0]	2 (1.90) [2]
Liver function tests increased	1 (1.92) [1]	0 (0.00) [0]	1 (0.95) [1]
Renal function tests abnormal	1 (1.92) [1]	0 (0.00) [0]	1 (0.95) [1]
Metabolism and nutrition disorders	0 (0.00) [0]	2 (3.77) [2]	2 (1.90) [2]
Hypercholesterolemia	0 (0.00) [0]	1 (1.89) [1]	1 (0.95) [1]
Hyperlipidemia	0 (0.00) [0]	1 (1.89) [1]	1 (0.95) [1]
Musculoskeletal and connective tissue disorders	1 (1.92) [1]	1 (1.89) [1]	2 (1.90) [2]
Arthralgia	0 (0.00) [0]	1 (1.89) [1]	1 (0.95) [1]
Back pain	1 (1.92) [1]	0 (0.00) [0]	1 (0.95) [1]
Nervous system disorders	0 (0.00) [0]	2 (3.77) [2]	2 (1.90) [2]
Essential tremor	0 (0.00) [0]	1 (1.89) [1]	1 (0.95) [1]
Headache	0 (0.00) [0]	1 (1.89) [1]	1 (0.95) [1]
Injury, poisoning and procedural complications	1 (1.92) [2]	0 (0.00) [0]	1 (0.95) [2]
Pathological fracture	1 (1.92) [1]	0 (0.00) [0]	1 (0.95) [1]
Ulna fracture	1 (1.92) [1]	0 (0.00) [0]	1 (0.95) [1]
Surgical and medical procedures	1 (1.92) [2]	0 (0.00) [0]	1 (0.95) [2]
Bone graft	1 (1.92) [1]	0 (0.00) [0]	1 (0.95) [1]
Open reduction of fracture	1 (1.92) [1]	0 (0.00) [0]	1 (0.95) [1]
Cardiac disorders	1 (1.92) [1]	0 (0.00) [0]	1 (0.95) [1]
Angina unstable	1 (1.92) [1]	0 (0.00) [0]	1 (0.95) [1]
Eye disorders	1 (1.92) [1]	0 (0.00) [0]	1 (0.95) [1]
Lacrimation increased	1 (1.92) [1]	0 (0.00) [0]	1 (0.95) [1]
General disorders and administration site conditions	1 (1.92) [1]	0 (0.00) [0]	1 (0.95) [1]
Suprapubic pain	1 (1.92) [1]	0 (0.00) [0]	1 (0.95) [1]
Neoplasms benign, malignant and unspecified	0 (0.00) [0]	1 (1.89) [1]	1 (0.95) [1]
Benign ovarian tumor	0 (0.00) [0]	1 (1.89) [1]	1 (0.95) [1]

## Discussion

To our knowledge, this is the first study comparing the efficacy of NAXOZOL and celecoxib with respect to gastrointestinal tract protection and pain relief in patients with osteoarthritis.

The baseline-adjusted LDQ scores at 12 weeks after treatment in the NAXOZOL group were not inferior to those in the celecoxib group. The overall change in the baseline-adjusted GSRS scores, VAS scores, the EQ-5D, and the EQ VAS, was not different between the two groups. The use of the supplementary drugs and the incidence of drug-related adverse events were not different. However, the days to use the rescue drug were longer in the celecoxib group than in the NAXOZOL group.

Celecoxib can minimize gastrointestinal complications related to the use of NSAIDs [3, 4]. However, there has been controversy over the gastrointestinal tract-related adverse events with the use of celecoxib. Based on a meta-analysis to determine efficacy and safety, the incidence of adverse gastrointestinal events in patients with osteoarthritis taking celecoxib is higher than in those given a placebo [25].

VIMOVO is a combination formulation of naproxen and esomeprazole magnesium. Naproxen is a commonly used NSAID that is effective at relieving pain in patients with osteoarthritis or rheumatoid arthritis [26]. In addition, the incidence of myocardial infarction was decreased in patients using naproxen compared with those using other NSAIDs [27]. Esomeprazole magnesium is a commonly used PPI that can control gastrointestinal bleeding and dyspepsia [28]. Patients taking naproxen/esomeprazole magnesium had less upper gastrointestinal complaints compared to those treated with naproxen only [29]. A meta-analysis showed that there was no difference in dyspepsia when comparing naproxen/esomeprazole magnesium with celecoxib as well as with PPI [10]. Based on these data, naproxen/esomeprazole magnesium has been approved for use in Europe. It has been approved for use in the USA to relieve the symptoms of osteoarthritis and to decrease the risk of ulcers in patients at-risk for developing NSAID-associated gastric ulcers.

NAXOZOL is a new combination of naproxen and esomeprazole strontium tetrahydrate. Like other NSAIDs, naproxen predominantly inhibits the activity of cyclooxygenase-2 (COX-2), thereby decreasing the synthesis of prostaglandin and thromboxane from arachidonic acid, exhibiting anti-inflammatory, analgesic, and anti-pyretic effects [26]. Esomeprazole strontium offered an innovative delivery mechanism compared to conventional PPIs [13]. Esomeprazole inhibits H+, K+-ATPase in gastric parietal cells. It is able to maintain high gastric pH for a longer period of time than other proton pump inhibitors (the maintenance time at pH >4 is 12 hours for other proton pump inhibitors, but is 16.8 hours for esomeprazole) [28]. The FDA has approved esomeprazole strontium for use in adults under the same indications as for esomeprazole magnesium [14]. Pharmacokinetic results showed that the geometric mean ratio and 90% confidence interval of Cmax and AUClast were 0.99 (0.94–1.06) and 1.00 (0.98–1.01) for naproxen, and 0.99 (0.82–1.18) and 1.04 (0.91–1.18) for esomeprazole, both of which are within the range that allows for acceptance of biological equivalence (0.8–1.25), showing similar systemic exposure, and, ultimately, the pharmacokinetic equivalence of naproxen and esomeprazole between naproxen/esomeprazole strontium (NAXOZOL) and naproxen /esomeprazole magnesium (VIMOVO) [15]. Based on a clinical study of the pharmacokinetics and safety of naproxen/esomeprazole strontium compared to VIMOVO, the pharmacokinetics of naproxen/esomeprazole strontium was comparable to that of VIMOVO [15]. Both drugs were well-tolerated with no safety issues [15]. However, there have been no studies comparing the combination of naproxen and esomeprazole strontium to celecoxib.

As with any study, our investigation has several limitations. First, the final number of study participants was small because of the high rate of losses to follow-up. This might be explained by the fact that the life style in Korean society is different from that in western European society or American society. However, the rate of missing data was similar between the groups throughout the follow-up period. Additionally, this study is a preliminary study. Second, the patients included in this study had mild dyspepsia. We excluded patients with severe dyspepsia based on exclusion criteria including gastrointestinal ulcers and gastrointestinal bleeding, perforation, penetration, gastric outlet obstruction, gastrointestinal cancer, gastroesophageal reflux, and gastrointestinal infection with Helicobacter. Therefore, mean LDQ scores of the study population at the initial visit was between 0.29 and 0.60, and the baseline-adjusted LDQ score after treatment (primary outcome) could not be larger than 0.40 of a minimal clinically important difference. In addition, the standard deviation based on the previous studies to compute the sample size was 0.65 [23, 24], and it is considerably larger in the analysis of the current study. So, the current study was underpowered. In the future, we plan to evaluate the efficacy of NAXOZOL in patients with severe dyspepsia. Despite these limitations, this study, to the best of our knowledge, represents the first study to compare the efficacy of NAXOZOL and celecoxib with respect to gastrointestinal tract protection and pain relief in patients with osteoarthritis.

## Conclusions

NAXOZOL was not inferior to celecoxib in protecting the gastrointestinal tract and alleviating pain in patients with osteoarthritis.

## Supporting information

S1 Table Treatment groups and number of subjects per group. (DOCX)

S2 Table List of Laboratory Safety Tests. (DOCX)

S3 Table Study schedule. (DOCX)

S1 Fig The general structure of the study. (TIF)

S2 Fig Investigator survey. (DOCX)

S3 Fig Subject survey. (DOCX)

S4 Fig Subject log. (DOCX)

S1 File English study protocol. (DOCX)

S2 File Korean study protocol. (DOCX)

S3 File CONSORT checklist. (DOCX)
