# Supplementary material for: A comparative study of the efficacy of NAXOZOL compared to celecoxib in patients with osteoarthritis
Source: PLoS One. 2020 Jan 27;15(1):e0226184. doi: 10.1371/journal.pone.0226184 (PMC6984721; doi:10.1371/journal.pone.0226184)
Supplement: S1 Table — (DOCX) [file pone.0226184.s001.docx]

#### S1 Table. Treatment groups and the number of subjects per group

|  | Dose | Number of subjects | Administered drug |
| --- | --- | --- | --- |
| Experimental | 1 tablet, twice a day | 53 | Naxozol tablet |
| Control | 1 capsule, once daily | 53 | Celebrex capsule |
