# Supplementary material for: A comparative study of the efficacy of NAXOZOL compared to celecoxib in patients with osteoarthritis
Source: PLoS One. 2020 Jan 27;15(1):e0226184. doi: 10.1371/journal.pone.0226184 (PMC6984721; doi:10.1371/journal.pone.0226184)
Supplement: S2 Table — (DOCX) [file pone.0226184.s002.docx]

#### S2 Table. List of laboratory safety tests

| **Hematology** | **Serum Chemistry** |
| --- | --- |
| Hemoglobin (Hgb) | Alanine aminotransferase (ALT) |
| Hematocrit (Hct) | Aspartate aminotransferase (AST) |
| RBC count | Blood urea nitrogen (BUN) |
| Platelet | Creatinine |
| WBC with differential count | Glucose  Total bilirubin |
