# Supplementary material for: A comparative study of the efficacy of NAXOZOL compared to celecoxib in patients with osteoarthritis
Source: PLoS One. 2020 Jan 27;15(1):e0226184. doi: 10.1371/journal.pone.0226184 (PMC6984721; doi:10.1371/journal.pone.0226184)
Supplement: S3 Table — (DOCX) [file pone.0226184.s003.docx]

## S3 Table. Study schedule

| Schedule | Screening & Washout period | | Treatment & Follow-up Period* | |
| --- | --- | --- | --- | --- |
|  | Screening1 | Randomization | | End of Study |
| Weeks | -2 - 0 | 0 | | 12 |
| Day of visit | -14 - 1 | 1 | | 84-87 |
| Visit category | V1 | V2 | | V3 |
| Consent |  |  | |  |
| Screening number assignment |  |  | |  |
| Inclusion / exclusion criteria |  |  | |  |
| Demographic information / medical history / medication history |  |  | |  |
| Physical examination 2 | (including weight) |  | |  |
| Vital signs3 |  |  | |  |
| X-ray (lesion)4 |  |  | |  |
| Pain VAS5 | - |  | |  |
| Randomization |  |  | |  |
| Blood test |  |  | |  |
| Blood chemistry test 6 |  |  | |  |
| Hematological test 7 |  |  | |  |
| Pregnancy test8 |  |  | |  |
| Drug administration |  |  | |  |
| Distribution of study drug / ancillary drug / rescue drug |  |  | |  |
| Collection of study drug / ancillary drug / rescue drug |  |  | |  |
| LDQ, GSRS, EQ-5D |  |  | |  |
| Distribution of subject log |  |  | |  |
| Collection of subject log |  |  | |  |
| Confirmation of co-administered drugs |  |  | |  |
| Adverse event monitoring |  |  | |  |

1. If additional washout is not needed, screening and randomization can be conducted on Day 1.
2. Height (history), weight (measured). Weight measured only at visit 1.
3. Blood pressure, pulse rate, respiration rate.
4. Not conducted if there is an X-ray result of the lesion taken within 6 months.
5. Conducted only at visit 2 and not at visit 1.
6. ALT, AST, BUN, creatinine, glucose, total bilirubin.
7. CBC (hemoglobin, hematocrit, RBC count, WBC with differential count, platelet count).
8. Conducted only in women of childbearing potential.

- **Additional visit**: Additional visits may be conducted if follow-up observation of adverse events is needed.
