# Supplementary material for: A comparative study of the efficacy of NAXOZOL compared to celecoxib in patients with osteoarthritis
Source: PLoS One. 2020 Jan 27;15(1):e0226184. doi: 10.1371/journal.pone.0226184 (PMC6984721; doi:10.1371/journal.pone.0226184)
Supplement: S2 Fig — (DOCX) [file pone.0226184.s005.docx]

## S2 Fig. Investigator survey

#### (Leeds Dyspepsia Questionnaire, LDQ)

| 1 | Over the last FOUR WEEKS have you had any **indigestion** (a pain in the upper abdomen) (see picture)?  YES [ ] NO [ ]  *If the answer is no please go to question 2.*  How severe has your indigestion been over the last FOUR WEEKS?  Very mild [ ] Mild [ ] Moderate [ ] Severe [ ]  Very severe [ ] | 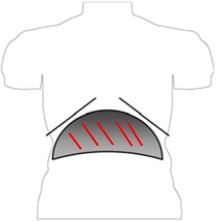 |
| --- | --- | --- |
|  |  | epigastric pain, discomfort, or burning.  * Note the difference in location from #2! |
| 2 | Over the last FOUR WEEKS have you experienced heartburn (a burning feeling behind the breast bone) (see picture)?  YES [ ] NO [ ]  *If the answer is no please go to question 3.*  How severe has your heartburn been over the last FOUR WEEKS?  Very mild [ ] Mild [ ] Moderate [ ] Severe [ ]  Very severe [ ] | 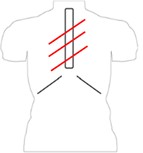 |
|  |  | Chest burning, discomfort, or pain. |
| 3 | Over the last FOUR WEEKS has **food or drink ever stuck behind your breast bone as it went down**?  YES [ ] NO [ ]  *If the answer is no please go to question 4.*  How severe has your symptom been over the last FOUR WEEKS?  Very mild [ ] Mild [ ] Moderate [ ] Severe [ ]  Very severe [ ] | Chest discomfort or pain when swallowing, odynophagia (ddx) or dysphagia. |
| 4 | Over the last FOUR WEEKS have you experienced any **regurgitation** (an acid taste coming up into your mouth from your stomach)?  YES [ ] NO [ ]  *If the answer is no please go to question 5.*  How severe has your regurgitation been over the last FOUR WEEKS?  Very mild [ ] Mild [ ] Moderate [ ] Severe [ ]  Very severe [ ] | Reflux of gastric contents (reflux) (ddx) belching, vomiting, or rumination. |

| 5 | Over the last FOUR WEEKS have you noticed excessive **burping or belching**?  YES [ ] NO [ ]  *If the answer is no please go to question 6.*  How severe has your belching been over the last FOUR WEEKS?  Very mild [ ] Mild [ ] Moderate [ ] Severe [ ]  Very severe [ ] | Belching, oral expulsion of gas only. |
| --- | --- | --- |
| 6 | Over the last FOUR WEEKS have you experienced any **nausea** (a feeling of sickness without actually being sick)?  YES [ ] NO [ ]  *If the answer is no please go to question 7.*  How severe has your nausea been over the last FOUR WEEKS?  Very mild [ ]  Mild [ ] Moderate [ ]  Severe [ ]  Very severe [ ] | Nausea, retching. |
| 7 | Over the last FOUR WEEKS have you experienced any **vomiting**?  YES [ ] NO [ ]  *If the answer is no please go to question 8.*  How severe has your vomiting been over the last FOUR WEEKS?  Very mild [ ] Mild [ ] Moderate [ ] Severe [ ]  Very severe [ ] | Accompanied by nausea or retching (ddx) reflux, rumination, belching. |
| 8 | Over the last FOUR WEEKS have you noticed an  **excessive feeling of fullness after eating**?  YES [ ] NO [ ]  *If the answer is no please go to question 9.*  How severe has your fullness been over the last FOUR WEEKS?  Very mild [ ] Mild [ ] Moderate [ ] Severe [ ]  Very severe [ ] | Post-prandial fullness, incorrect sensation of fullness. |
| 9 | Which, if any, of these symptoms has been the most troublesome to you over the last FOUR WEEKS?  TICK ONE BOX ONLY   1. Heartburn [ ] 2. Regurgitation [ ] 3. Indigestion [ ] 4. Belching [ ] 5. Nausea [ ] 6. Vomiting [ ] 7. Excessive fullness [ ] 8. None of these has troubled me [ ] |  |
