# Supplementary material for: A comparative study of the efficacy of NAXOZOL compared to celecoxib in patients with osteoarthritis
Source: PLoS One. 2020 Jan 27;15(1):e0226184. doi: 10.1371/journal.pone.0226184 (PMC6984721; doi:10.1371/journal.pone.0226184)
Supplement: S3 Fig — (DOCX) [file pone.0226184.s006.docx]

## S3 Fig. Subject survey

| S |  |  |  |  |  |
| --- | --- | --- | --- | --- | --- |
|  |  | - |  |  |  |
|  |  |  |  |  |  |

| **Protocol number** | **Naxozol_P4_1** | | | | | | | | | | | | |
| --- | --- | --- | --- | --- | --- | --- | --- | --- | --- | --- | --- | --- | --- |
| **Screening number** |  | | | | | | | | | | | | |
| **Randomization number** |  | | | | | | | | | | | | |
|  |  | **R** |  | | |  | |  | |  | |  |  |
|  |  |  |  |  |  | - | |  |  |  |  |  |  |
|  |  |  |  | | |  | |  | |  | |  |  |
| **Subject initials** |  | | | | | | | | | | | | |
|  |  | | |  | | |  | |  | |  | | |
|  |  |  |  |  | | |  | |  | |  |  |  |
| **Date of visit** | **Visit 2** | | | | YY MM DD | | | | | | | | |
|  | **Visit 3** | | | |  |  |  |  |  |  |  |  |  |
| **Investigator** |  | | | | | | | | | | | | |

- - Thank you very much for participating in our clinical trial.
  - The information provided on this form will not be used for purposes other than this study.
  - Please fill out the form as accurately and credibly as possible.
  - Please return this form after completion.
  - Thank you again for participating in this study.

**Pain (VAS)**

| Protocol number | Visit | Screening number | | | | Randomization number | | | | Initials |
| --- | --- | --- | --- | --- | --- | --- | --- | --- | --- | --- |
| Naxozol_P4_1 |  | S |  |  |  | R |  |  |  |  |

Please indicate your pain level by drawing a line across the straight line below (VAS):

**(Pain VAS)**

| **No pain** |  | **Worst pain imaginable** |
| --- | --- | --- |
|  |  |  |

#### Gastrointestinal Symptom Rating Scale (GSRS)

Please read the following and check the number that corresponds to your experience over the past 3 months.

- 1.
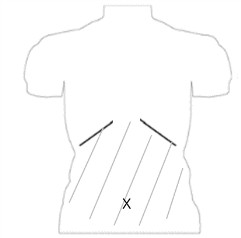
**Abdominal pain**

This means pain in the entire area of the abdomen (below the ribs, not in the chest).

[0]. No pain, or momentary pain.

[1]. Pain or discomfort that occurs intermittently and hinders activity.

[2]. Prolonged pain or discomfort that required alleviation and hinders many social activities.

[3]. Severe and debilitating pain that affects all social activities.

#### Heartburn


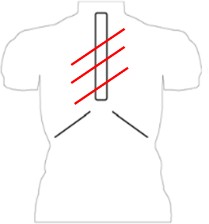
This means burning pain in the esophagus area (post-sternal).

[0]. No burning, or momentary burning.

[1]. Short-term discomfort that occurs intermittently.

[2]. Prolonged discomfort that occurs often and requires alleviation.

[3]. Prolonged discomfort that is temporarily alleviated by antacids.

#### Acid regurgitation

This means unpleasant reflux of acidic fluid from the stomach towards the chest.

[0]. No regurgitation, or momentary regurgitation.

[1]. Bothersome regurgitation that occurs intermittently.

[2]. Regurgitation that occurs 1-2 times a day and requires alleviation.

[3]. Regurgitation that occurs many times a day and is only temporarily and ineffectively alleviated by antacids.

#### Burning sensation in the epigastrium


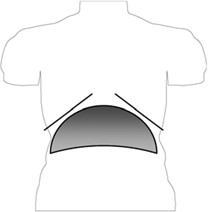
This means a burning sensation in the middle of the upper abdomen.

[0]. No burning sensation, or momentary sensation.

[1]. Short-term discomfort that occurs intermittently; no antacids or food needed between meals.

[2]. Prolonged discomfort that occurs frequently and requires food or antacids between meals.

[3]. Prolonged discomfort that often requires food or antacids between meals.

#### Nausea and vomiting

[0]. No nausea.

[1]. Short-term nausea that occurs intermittently.

[2]. Prolonged nausea that occurs frequently without vomiting.

[3]. Continuous nausea with frequent vomiting.

#### Borborygmus

This means a gurgling sound in the abdomen.

[0]. No borborygmus or intermittent borborygmi.

[1]. Bothersome, short-term borborygmi that occur intermittently.

[2]. Prolonged, frequent borborygmi that are inhibited by movement and do not hinder social activities.

[3]. Continuous borborygmi that severely hinder social activities.

#### Abdominal distension

This means fullness, a feeling of indigestion, and flatulence.

[0]. No distension, or momentary distension.

[1]. Short-term discomfort that occurs intermittently.

[2]. Prolonged discomfort that occurs frequently and can be increased by tight-fitting clothes.

[3]. Continuous discomfort that severely hinders social activities.

#### Eructation

This is expulsion of gas through the mouth without rumination, which is regurgitation of gastric contents, and vomiting.

[0]. No eructation, or momentary eructation.

[1]. Bothersome eructation that occurs intermittently.

[2]. Frequent eructation that hinders some social activities.

[3]. Frequent eructation that severely hinders social activities.

#### (EQ-5D)

Please read the following and check the number that best corresponds to your status.

1. **Exercise**
2. I have no issues with walking.
3. I have some issues with walking.
4. .I need to sit / lie down all the time.

#### Self-care

1. I have no issues with bathing or dressing.
2. I have some issues with bathing or dressing.
3. I cannot bathe or dress by myself.

#### Daily activities (work, study, housework, family, leisure)

1. I have no issues with daily activities.
2. I have some issues with daily activities.
3. I cannot perform daily activities.

#### Pain / discomfort

1. I do not have pain or discomfort.
2. I have moderate pain or discomfort.
3. I have severe pain or discomfort.

#### Anxiety / depression

1. I am neither anxious nor depressed.
2. I am somewhat anxious or depressed.
3. I am severely anxious or depressed.

####
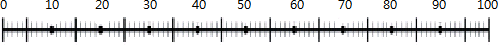
How would you rate your overall health status?

Very bad Very good

#### Thank you for taking the time to answer the questions above.
