# Supplementary material for: A comparative study of the efficacy of NAXOZOL compared to celecoxib in patients with osteoarthritis
Source: PLoS One. 2020 Jan 27;15(1):e0226184. doi: 10.1371/journal.pone.0226184 (PMC6984721; doi:10.1371/journal.pone.0226184)
Supplement: S4 Fig — (DOCX) [file pone.0226184.s007.docx]

## S4 Fig. Subject log

| Protocol number | Screening number | | | | Randomization number | | | | Subject initials | | | | |
| --- | --- | --- | --- | --- | --- | --- | --- | --- | --- | --- | --- | --- | --- |
| Naxozol_P4_1 | S |  |  |  | R |  |  |  |  |  |  |  |  |

If you have taken the ancillary drug (Hanmi Almagate 500 mg tablet) or the rescue drug (Hanmi Susphen ER 650 mg tablet), please fill in the form below.

| **Date administered**  (e.g.: 2014 July 10  = **1 4 0 7 1 0**  Please enter one number per box). | | | | | | | **ⓛ Ancillary drug** | **② Rescue drug** |
| --- | --- | --- | --- | --- | --- | --- | --- | --- |
|  |  |  |  |  |  |  | **Hanmi Almagate 500 mg tablet**  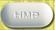 | **Susphen ER 650 mg tablet**  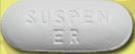 |
|  |  |  |  |  |  |  | Take if gastrointestinal dysfunction is severe beyond toleration. Do not exceed 6 tablets per day. | Take if pain is severe beyond toleration. Do not exceed 4 tablets per day. |
| 1 |  |  |  |  |  |  | Tablets | Tablets |
|  | Y | Y | M | M | D | D |  |  |
| 2 |  |  |  |  |  |  | Tablets | Tablets |
|  | YY MM DD | | | | | |  |  |
| 3 |  |  |  |  |  |  | Tablets | Tablets |
|  | YY MM DD | | | | | |  |  |
| 4 |  |  |  |  |  |  | Tablets | Tablets |
|  | YY MM DD | | | | | |  |  |
| 5 |  |  |  |  |  |  | Tablets | Tablets |
|  | YY MM DD | | | | | |  |  |
| 6 |  |  |  |  |  |  | Tablets | Tablets |
|  | YY MM DD | | | | | |  |  |
| 7 |  |  |  |  |  |  | Tablets | Tablets |
|  | YY MM DD | | | | | |  |  |
| 8 |  |  |  |  |  |  | Tablets | Tablets |
|  | YY MM DD | | | | | |  |  |
| 9 |  |  |  |  |  |  | Tablets | Tablets |
|  | YY MM DD | | | | | |  |  |
| 10 |  |  |  |  |  |  | Tablets | Tablets |
|  | YY MM DD | | | | | |  |  |
