# Supplementary material for: A comparative study of the efficacy of NAXOZOL compared to celecoxib in patients with osteoarthritis
Source: PLoS One. 2020 Jan 27;15(1):e0226184. doi: 10.1371/journal.pone.0226184 (PMC6984721; doi:10.1371/journal.pone.0226184)
Supplement: S1 File — (DOCX) [file pone.0226184.s008.docx]

**Protocol**

| **Protocol number** | **Naxozol_P4_1** |
| --- | --- |
| **Title** | A prospective, randomized, double-blind, double-placebo, active-controlled, multicenter, interventional study to compare the gastroprotective and pain-relieving effects of Naxozol and celecoxib in patients with osteoarthritis |
| **Phase** | **Phase 4** |
| **Sponsor** | **Sung Hwan Moon (Severance Hospital, Department of Orthopedics)** |
| **Protocol** | **Version 1.2** |

**Table of contents**

[Terminologies and Abbreviations 5](#_Toc534043372)

[Synopsis 7](#_Toc534043373)

[1. Sponsor, investigator, and administrative information 11](#_Toc534043374)

[1.1. Title 11](#_Toc534043375)

[1.2. Sponsor and address 11](#_Toc534043376)

[1.3. Institution/principle investigator/sub-investigator/manager 11](#_Toc534043377)

[1.3.1. Institution 11](#_Toc534043378)

[1.3.2. Name and title of principle investigator and sub-investigator 11](#_Toc534043379)

[1.3.2.1. Name and title of principle investigator and sub-investigator 11](#_Toc534043380)

[1.3.2.2. Name of pharmacist managing investigational products 11](#_Toc534043381)

[1.4. Data Safety Monitoring Committee 11](#_Toc534043382)

[2. Background and Rationale 12](#_Toc534043383)

[2.1. Name of medicinal product and summary of physicochemical characteristics 12](#_Toc534043384)

[2.2. Summary of preclinical metabolic, pharmacokinetic, and ADME study results 12](#_Toc534043385)

[2.3. Mechanism of action and summary of biological target organ 12](#_Toc534043386)

[2.4. Summary of clinical pharmacological study 13](#_Toc534043387)

[2.5. Summary of study subject characteristics 13](#_Toc534043388)

[2.6. Rationale for administration of investigational product in study subjects 14](#_Toc534043389)

[2.7. Summary of efficacy and safety of investigational product 14](#_Toc534043390)

[2.8. Experience with investigational product 14](#_Toc534043391)

[2.9. Discussion of risk-benefit of investigational product administration 14](#_Toc534043392)

[2.10. Discussion on study design 15](#_Toc534043393)

[2.11. Conclusion 15](#_Toc534043394)

[3. Purpose 16](#_Toc534043395)

[4. Plan 17](#_Toc534043396)

[4.1. General study methodology and plan 17](#_Toc534043397)

[4.2. Review and discussion on study design 19](#_Toc534043398)

[4.2.1. Review of study design 19](#_Toc534043399)

[4.2.2. Review of treatment duration 19](#_Toc534043400)

[4.2.3. Dose selection and rationale 19](#_Toc534043401)

[4.2.4. Review of control group selection 20](#_Toc534043402)

[4.2.5. Review of study group allocation 20](#_Toc534043403)

[4.2.6. Inclusion of special populations as subjects 20](#_Toc534043404)

[4.3. Subject selection 20](#_Toc534043405)

[4.3.1. Inclusion criteria 20](#_Toc534043406)

[4.3.2. Exclusion criteria 20](#_Toc534043407)

[4.4.1. Assignment of screening number and subject number 21](#_Toc534043408)

[4.4.2. Study group allocation 21](#_Toc534043409)

[4.5. Subject withdrawal criteria 22](#_Toc534043410)

[4.5.1. Study withdrawal 22](#_Toc534043411)

[4.5.2. Discontinuation of investigational product use 23](#_Toc534043412)

[4.6. Substitution of subject 23](#_Toc534043413)

[4.7. Addition of new dosage groups 23](#_Toc534043414)

[4.8. Early termination of study 23](#_Toc534043415)

[4.9. Termination of study 24](#_Toc534043416)

[4.10. Other considerations 24](#_Toc534043417)

[5. Investigational product and other study drugs 25](#_Toc534043418)

[5.1. Investigational product 25](#_Toc534043419)

[5.1.1. Experimental drug 25](#_Toc534043420)

[5.1.1.1. Product name/code 25](#_Toc534043421)

[5.1.1.2. Pharmaceutical form 25](#_Toc534043422)

[5.1.1.3. Qualitative and quantitative composition 25](#_Toc534043423)

[5.1.1.4. Storage condition 25](#_Toc534043424)

[5.1.2. Placebo of experimental drug 25](#_Toc534043425)

[5.1.3. Control drug 25](#_Toc534043426)

[5.1.3.1. Product name/code 25](#_Toc534043427)

[5.1.3.2. Pharmaceutical form 25](#_Toc534043428)

[5.1.3.3. Qualitative and quantitative composition 25](#_Toc534043429)

[5.1.3.4. Storage condition 25](#_Toc534043430)

[5.1.4. Placebo of control drug 25](#_Toc534043431)

[5.2. Expected dosage and administration 25](#_Toc534043432)

[5.2.1. Expected dosage and administration of experimental drug 25](#_Toc534043433)

[5.2.2. Expected dosage and administration of control drug 26](#_Toc534043434)

[5.3. Other drugs used in the study 26](#_Toc534043435)

[5.3.1. Concomitant pharmacological therapy and treatment 26](#_Toc534043436)

[5.3.1.1. Supplementary medication 26](#_Toc534043437)

[5.3.1.2. Rescue medication 27](#_Toc534043438)

[5.3.2. Drugs or therapies contraindicated prior to study participation 27](#_Toc534043439)

[5.3.3. Drugs or therapies contraindicated during study participation 27](#_Toc534043440)

[5.3.4. Precautions regarding experimental drug 28](#_Toc534043441)

[5.3.4.1. Adverse events associated with experimental drug 28](#_Toc534043442)

[5.3.4.2. Contraindications for experimental drug 31](#_Toc534043443)

[5.3.4.3. Warnings for experimental drug 32](#_Toc534043444)

[5.3.4.4. Precautions for experimental drug 32](#_Toc534043445)

[5.3.4.5. Drug Interactions 34](#_Toc534043446)

[5.3.4.6. Special Population 36](#_Toc534043447)

[5.3.5. Precautions for Control Drug 37](#_Toc534043448)

[5.3.5.1. Adverse Events Associated with Control Drug 37](#_Toc534043449)

[5.3.5.2. Contraindications for control drug 39](#_Toc534043450)

[5.3.5.3. Warnings for Control Drug 40](#_Toc534043451)

[5.3.5.4. Precautions regarding control drug 40](#_Toc534043452)

[5.4. Packaging and labelling 46](#_Toc534043453)

[5.5. Supply, handling, and storage 47](#_Toc534043454)

[5.6. Distribution of the Investigational Product 47](#_Toc534043455)

[5.7. Treatment compliance 48](#_Toc534043456)

[5.8. Blinding 48](#_Toc534043457)

[5.9. Emergency unblinding 49](#_Toc534043458)

[5.10. Management of overdose 49](#_Toc534043459)

[5.11. Evaluation and treatment of subjects after study termination 50](#_Toc534043460)

[6. Study procedure and evaluation 51](#_Toc534043461)

[6.1. Evaluation schedule 51](#_Toc534043462)

[6.2. Demographic information and other baseline evaluations 53](#_Toc534043463)

[6.3. Safety evaluation 53](#_Toc534043464)

[6.3.1. Adverse events 54](#_Toc534043465)

[6.3.1.1. Definition of adverse events 54](#_Toc534043466)

[6.3.2. Exposure of pregnant women 60](#_Toc534043467)

[6.3.3. Laboratory tests 60](#_Toc534043468)

[6.3.4. Vital signs, physical examination, and other evaluations 61](#_Toc534043469)

[6.4. Efficacy evaluation 61](#_Toc534043470)

[6.4.1. Primary evaluation 61](#_Toc534043471)

[6.4.2. Secondary evaluation 61](#_Toc534043472)

[6.5. Other evaluations 62](#_Toc534043473)

[7. Statistical analysis plan 63](#_Toc534043474)

[7.1. Determination of subject number 63](#_Toc534043475)

[7.2. Randomization 64](#_Toc534043477)

[7.3. Evaluation endpoints 64](#_Toc534043478)

[7.3.1. Primary evaluation endpoint 64](#_Toc534043479)

[7.3.2. Secondary evaluation endpoint 64](#_Toc534043480)

[7.3.3. Other evaluation endpoints 65](#_Toc534043481)

[7.4. Definition of analysis sets 65](#_Toc534043482)

[7.5. Statistical analysis plan 66](#_Toc534043483)

[7.5.1. General considerations 66](#_Toc534043484)

[7.5.2. Primary outcome analysis 66](#_Toc534043485)

[7.5.3. Secondary outcome analysis 66](#_Toc534043486)

[7.5.4. Demographic information 70](#_Toc534043487)

[7.6. Interim analysis 70](#_Toc534043488)

[8. Ethico-legal aspect of the trial 71](#_Toc534043489)

[8.1. Responsibilities of the investigator 71](#_Toc534043490)

[8.2. Subject information and consent 71](#_Toc534043491)

[8.3. Subject identification and confidentiality 71](#_Toc534043492)

[8.4. Subject compensation and insurance 72](#_Toc534043493)

[8.5. Institutional review board (IRB) 72](#_Toc534043494)

[8.6. Government authorities 72](#_Toc534043495)

[9. Clinical Trial Management 73](#_Toc534043496)

[9.1. Management of case report forms 73](#_Toc534043497)

[9.2. Evidence and subject files 73](#_Toc534043498)

[9.3. Storage of the investigator file and data 74](#_Toc534043499)

[9.4. Monitoring quality control, government audit 75](#_Toc534043500)

[9.5. Change of protocol 75](#_Toc534043501)

[9.6. Principles of clinical study reports and data presentation 76](#_Toc534043502)

[9.6.1. Clinical study report 76](#_Toc534043503)

[9.6.2. Data presentation 76](#_Toc534043504)

[10. References 77](#_Toc534043505)

[11. Appendices 87](#_Toc534043506)

[Appendix 1. Study schedule 87](#_Toc534043507)

[Appendix 2. Investigator survey 88](#_Toc534043508)

[Appendix 3. Subject survey 90](#_Toc534043509)

[Appendix 4. Subject log 94](#_Toc534043510)

[Appendix 5. Signature form 95](#_Toc534043511)

[Sponsor’s signature 95](#_Toc534043512)

[Principal Investigator’s Signature 96](#_Toc534043513)

[Appendix 6. Compensation clause 97](#_Toc534043514)

[12. Attachments : Documents Controlled & Filed Seperately 98](#_Toc534043515)

Institution, principal investigator, sub-investigator, managing pharmacist, and sponsor/contract research organization  [98](#_Toc534043516)

Explanation and consent for study subject  [99](#_Toc534043517)

# Terminologies and Abbreviations

| ACE | Angiotensin Converting Enzyme |
| --- | --- |
| ACG | American College of Gastroenterology |
| AGEP | Acute Generalized Exanthematous Pustulosis |
| ALB | Albumin |
| ALP | Alkaline Phosphatase |
| ALT | Alanine Aminotransferase |
| AST | Aspartate Aminotransferase |
| BUN | Blood Urea Nitrogen |
| CABG | Coronary Artery Bypass Graft |
| CBC | Complete Blood Count |
| COX | Cyclo-Oxygenase |
| CPK | Creatine Phosphokinase |
| CRC | Clinical Research Coordinator |
| CRF | Case Report Form |
| CRO | Contract Research Organization |
| CTCAE | Common Terminology Criteria for Adverse Events |
| CYP | Cytochrome |
| DBP | Diastolic Blood Pressure |
| DRESS | Drug Rash with Eosinophilia and Systemic Symptom |
| DSMB | Data and Safety Monitoring Board |
| ECL | Enterochromaffin-Like |
| ELISA | Enzyme-Linked Immunosorbent Assay |
| FAS | Full Analysis Set |
| GCP | Good Clinical Practice |
| GFR | Glomerular Filtration Rate |
| γ-GT | Gamma Glutamyltransferase |
| GSRS | Gastrointestinal Symptom Rating Scale |
| Hb | Hemoglobulin |
| HCV | Hepatitis C Virus |
| HR | Heart Rate |
| ICH | International Conference on Harmonisation of Technical Requirements for Registration of Pharmaceuticals for Human Use |
| IND | Investigational New Drug |
| ITT | Intent-To-Treat |
| KGCP | Korean Good Clinical Practice |
| LDA | Low Dose Aspirin |
| LDH | Lactate Dehydrogenase |

| LDQ | Leeds Digestion Questionnaire |
| --- | --- |
| MCD | Minimal Change Disease |
| MCID | Minimal Clinically Important Difference |
| MCTD | Mixed Connective Tissue Disease |
| MedDRA | Medical Dictionary for Regulatory Activities |
| MRI | Magnetic Resonance Imaging |
| NPN | Non-Protein Nitrogen |
| PLT | Platelet |
| PR | Pulse Rate |
| PPI | Proton Pump Inhibitor |
| PTT | Partial Thromboplastin Time |
| PT | Preferred Term |
| RBC | Red Blood Cell |
| SAE | Serious Adverse Event |
| SLE | Systemic Lupus Erythematosus |
| SOC | System Organ Class |
| SODA | Severity of Dyspepsia Assessment |
| SUSAR | Suspected Unexpected Serious Adverse Reaction |
| TEN | Toxic Epidermal Necrolysis |
| UNL | Upper Normal Limit |
| VAS | Visual Analogue Scale |
| WBC | White Blood Cell |
| WHO | World Health Organization |
| WHOART | WHO Adverse Reactions Terminology |

# Synopsis

| Trial Title | A prospective, randomized, double-blind, double-placebo, active-controlled, multicenter, interventional study to compare the gastroprotective and pain-relieving effects of Naxozol and celecoxib in patients with osteoarthritis | | |
| --- | --- | --- | --- |
| Trial Number | Naxozol_P4_1 | Version | 1 |
| Sponsor | Sung Hwan Moon (Severance Hospital, Department of Orthopedics) | | |
| Phase | Phase 4 (investigator-initiated trial) | | |
| IND | Applicable 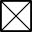 Not applicable | | |
| Institutions / Countries | 10 institutions / Korea | | |
| Expected duration of study | Approximately 24 months after study initiation | | |
| Purpose | **Primary Purpose**  To prove non-inferiority of the gastroprotective effect of a 12-week administration of Naxozol to osteoarthritis patients compared to that of celecoxib, as evaluated by the Leeds Dyspepsia Questionnaire (LDQ).  **Secondary Purpose**   - To compare the gastroprotective effect, as evaluated by the Gastrointestinal Symptom Rating Scale (GSRS), of 12 weeks of treatment of osteoarthritis patients with Naxozol or celecoxib. - To compare the incidence rate of gastrointestinal (GI) adverse events, as well as the drug discontinuation rate due to gastrointestinal (GI) adverse events, after 12 weeks of treatment of osteoarthritis patients with Naxozol or celecoxib. - To compare the pain improvement effect, as evaluated by the visual analogue scale (VAS), of 12 weeks of treatment of osteoarthritis patients with Naxozol or celecoxib. - To compare the improvement in quality of life (QoL), as evaluated by EQ-5D, of 12 weeks of treatment of osteoarthritis patients with Naxozol or celecoxib. - To compare patient compliance during 12 weeks of treatment of osteoarthritis patients with Naxozol or celecoxib. - To compare overall safety of Naxozol and celecoxib after 12 weeks of treatment of osteoarthritis patients. | | |
|  |  |  |  |
|  |  |  |  |

| Planned study design | Prospective, randomized, double-blind, double-placebo, active-controlled, two-arm, parallel, multi-center trial. |
| --- | --- |
| Expected number of study subjects | A minimum of 106 (53 in experimental group, 53 in control group), considering a 20% discontinuation rate, with the final aim of 42 subjects in each of the two groups. |
| Visit schedule and evaluation | Screening & washout period: ~14 days (Day -14 to Day 1 [Week -2 to 0]).  Treatment and F/U period: 12 weeks (Day 1 to Day 84 [Week 12]).  **Visit Schedule for Evaluation**  Visit 1: screening & washout, Day -14 to Day 1.  Visit 2: randomization, Day 1.  Visit 3: end of study, Day 84±7 from date of randomization.  **Schedule for Evaluation**  **1) Safety and tolerability evaluation**  Adverse events, physical examination, vital signs, laboratory tests (hematological test, blood chemistry), co-administered drugs.  Adverse events: V1-V3, at every visit.  Co-administered drugs: V1-V3, at every visit.  Vital signs (VS): at V1 and V3.  Physical examination: at V1 and V3.  Laboratory tests:  - Hematological test: at V1 and V3.  - Blood chemistry: at V1 and V3. |
| Inclusion criteria | 1. Subjects of Korean nationality who voluntarily decide to participate in the study after receiving and understanding a detailed explanation of the study and provide written consent. 2. Men and women who are ≥50 years old at the time of consent. 3. Persons who are available for follow-up for the duration of the study and who are able to perform tasks for the study, such as reading and filling out EQ-5D and VAS surveys. 4. Persons with a confirmed medical history of physically symptomatic osteoarthritis who score ≥40 mm on pain VAS performed at V2. 5. Persons deemed appropriate for the study based on the screening results (N.B.: subjects can be included at the discretion of the investigator even if laboratory test results are outside reference ranges). |
| Exclusion criteria | 1. Persons who are participating in a clinical study or have participated in a clinical study <30 days prior (participation is based on the date of final investigational product administration). 2. Persons with a history of abuse of alcohol or other drugs <6 months prior to screening. 3. Persons who drink 21 standard drinks or more per week (generally, 55 mL soju is equated to 200 mL beer or 30 mL spirits). 4. Persons with a history of peptic ulcer disease with complications (bleeding, perforation, penetration, and gastric outlet obstruction) within the last 5 years. 5. Persons with a history of peptic ulcer disease without complications within 6 months prior to screening visit or persons with active peptic ulcer disease. 6. Persons who have been infected with *Helicobacter* but did not undergo eradication therapy. 7. Persons with a known history of gastroesophageal reflux disease. 8. Persons with a confirmed diagnosis or clinical suspicion of the following arthropathies that may affect safety and efficacy evaluation: septic arthritis, inflammatory arthropathy such as rheumatoid arthritis, gout, recurrent pseudopain, Paget’s disease, fracture, ochronosis, acromegaly, hemochromatosis, Wilson’s disease, primary osteomalacia, and Ehlers Danlos Syndrome and other genetic collagen disorders. 9. Persons with surgery planned during the study period. 10. Persons with a history of gastrointestinal cancer. 11. Persons with a GI disorder due to drug absorption malfunction. 12. Persons with gastrointestinal bleeding, cerebrovascular bleeding, other bleeding disorders, or severe hematological disorders. 13. Persons with moderate to severe hepatic impairment (Child Pugh Class II or higher). 14. Persons with a history of severe heart failure or coronary artery bypass graft (CABG). 15. Persons with severe renal impairment (CrCl <30 mL / min). 16. Persons with severe hypertension (SBP ≥160 mmHg or DBP ≥100 mmHg) refractory to treatment. 17. Persons with a history of allergy to components of the investigational product. 18. Persons with hypersensitivity or allergy to other NSAIDs or PPI. 19. Subjects who received a contraindicated drug or therapy within a pre-specified period prior to study participation (Section 5.3.2). 20. Patients who are receiving a contraindicated co-administration drug (Section 5.3.3). 21. Patients who have a history of surgery on the osteoarthritic joint within the past year. 22. Women who are pregnant or breastfeeding, or women of childbearing age who are not using appropriate contraception (condom, diaphragm, intrauterine contraceptive device, or hormonal contraceptive drug used from ≥21 days prior to investigational product administration, or male vasectomy); this clause does not apply to menopausal women. 23. Persons deemed inappropriate for study enrollment by the investigator due to clinically relevant medical or psychiatric conditions. |
| **Experimental Drug** | Naxozol tablet: combination (naproxen 500 mg + esomeprazole 20 mg).  Dosage and administration: 1 tablet, twice daily. Do not split, chew, or crush tablet. Take 30 - 60 minutes before meal.  *Experimental group: Administer 1 Naxozol tablet 30 - 60 minutes before breakfast and 1 Naxozol tablet with 1 Celebrex placebo capsule 30 - 60 minutes before dinner. |
| **Control Drug** | Celebrex capsule: celecoxib 200 mg.  Dosage and administration: 1 capsule, once daily. Do not split, chew, or crush tablet. Take 30 - 60 minutes before meal (approved dosage and administration: 1 capsule, once daily, regardless of meal time).  *Control group: Administer 1 Naxozol placebo tablet 30 - 60 minutes before breakfast and 1 Naxozol placebo tablet with 1 Celebrex capsule 30 - 60 minutes before dinner. |
| **Planned Duration of Study** | 12 weeks total per subject. |
| **Primary outcome** | Average change in the Leeds Dyspepsia Questionnaire (LDQ) from baseline after 12 weeks of oral administration of investigational product. |
| **Secondary outcomes** | - Average change in the Leeds Dyspepsia Questionnaire (LDQ) from baseline after 12 weeks of oral administration of the investigational product. - Average change in the Gastrointestinal Symptom Rating Scale (GSRS) from baseline after 12 weeks of oral administration of the investigational product. - The incidence rate of GI adverse events (dyspepsia, diarrhea, nausea, abdominal pain, heartburn) after 12 weeks of oral administration of the investigational product. - The discontinuation rate due to GI adverse events after 12 weeks of oral administration of the investigational product. - Average change in the pain Visual Analogue Scale (VAS) from baseline after 12 weeks of oral administration of the investigational product. - Average change in the average EQ-5D score from baseline after 12 weeks of oral administration of the investigational product. - Drug compliance during 12 weeks of oral administration of the investigational product. - Ancillary and rescue drug use during 12 weeks of oral administration of the investigational product: average number of days used; average daily amount used (total amount used / total number of days used); and average amount of use during study participation (total amount used / study duration). - The incidence, frequency, and characteristics (clinical presentation, severity, result, etc) of adverse events during 12 weeks of oral administration of the investigational product. - Results of physical examination, vital signs, and radiological tests after 12 weeks of oral administration of the investigational product. |
| **Data analysis** | Definition of the analysis groups   - Safety evaluation group: subject group in which safety follow-up was performed at least once after randomization and investigational product administration. - FAS evaluation group: subject group in which efficacy evaluation was performed at least once after randomization and investigational product administration. - Per Protocol(PP) evaluation group: subject group in which efficacy evaluation was performed at least once after randomization and investigational product administration, with no major protocol violation.   Statistical analysis   - FA group (Modified ITT) and PP group are both considered the main analysis groups according to ICH recommendations, as this is a non-inferiority study. - If missing data occur during the study, they are substituted using the multiple imputation method. - To prove non-inferiority of the experimental drug, the one-sided 97.5% confidence interval for the difference in change in LDQ at 12 weeks compared to baseline between the two groups is obtained. If the 97.5% upper confidence limit is less than the pre-specified non-inferiority margin of 0.40, the null hypothesis is rejected, and non-inferiority is proven. - All statistical analyses, except the non-inferiority analysis, are fundamentally based on the two-sided test with an alpha level of 0.05. - No interim analysis is to be performed. - The mean, standard deviation, median, minimum and maximum values for continuous variables, and frequency and proportion for categorical variables, must be presented on principle, and the incidence frequency is presented if necessary.   The change between each time of measurement, or from the baseline of demographical and other baseline data, safety data, and efficacy data, are summarized in descriptive statistical values by treatment group. Additionally, normal / abnormal changes in physical examination and laboratory test results are shown in partition tables by treatment group.  Co-administered drugs are recorded in ATC terminologies, and adverse events are standardized to system organ class (SOC) or preferred term (PT) using a medical dictionary (MedDRA or WHOART). |

# Sponsor, investigator, and administrative information

## Title

A prospective, randomized, double-blind, double-placebo, active-controlled, multicenter, interventional study to compare the gastroprotective and pain-relieving effects of Naxozol and celecoxib in patients with osteoarthritis.

## Sponsor and address

| Sponsor: | Sung Hwan Moon MD, PhD. (Severance Hospital, Department of Orthopedics) |
| --- | --- |
| Address | Yonsei-ro 50-1, Seodaemun-gu, Seoul |

## Institution / principle investigator / subinvestigator / manager

## Institution

This study will be conducted in 10 institutions across Korea (for the institution names refer to: 12. Attachments). The list of institutions will be managed as a separate document to that of the protocol document.

## Name and title of principle investigator, sub-investigator and manager

## Name and title of principle investigator and sub-investigator

The signatures and signature forms of the principal investigators at participating institutions are contained within Appendix 5.

The names and titles of the principal investigators, co-investigators, and sub-investigators at participating institutions will be managed as separate documents (see 12. Attachments).

## Name of pharmacist managing investigational products

The list of names of the pharmacists managing the investigational products at participating institutions will be managed as separate documents (see 12. Attachments).

## 1.4. Data Safety Monitoring Committee

A data safety monitoring committee will not be appointed for this study.

# Background and Rationale

## Name of medicinal product and summary of physicochemical characteristics

Product name: Naxozol tablet

Active ingredients: Naproxen (USP) 500 mg and esomeprazole strontium tetrahydrate 24.60 mg (20 mg as esomeprazole)

Form: rectangular pale-yellow film-coated tablet

Summary: Naxozol tablets contain 500 naproxen and 24.60 mg esomeprazole strontium tetrahydrate. Naxozol is a combination drug coated with fast-release esomezol and containing enteric-coated naproxen. The active ingredients contained within Naxozol tablets are as follows:

#### Naproxen


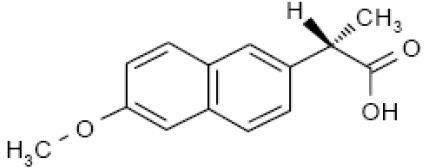


Chemical name: (S)-6-methoxy-α-methyl-2-naphthaleneacetic acid Molecular formula: C14H14O3

Molecular weight: 230.26

**Esomeprazole strontium tetrahydrate**


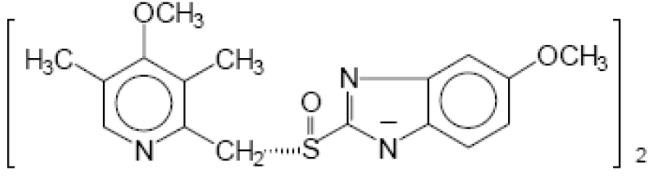
Sr2+ • 4H2O

Chemical name: bis (5-methoxy-2-[(S)-[(4-methoxy-3,5-dimethyl-2-pyridinyl)methyl] sulfinyl]-1H-benzimidazole-1-yl) strontium tetrahydrate

Molecular formula: C34H36N6O6S2Sr. 4H2O

Molecular weight: 848.50

## Summary of preclinical metabolic, pharmacokinetic, and ADME study results

Naproxen is a widely used non-steroidal anti-inflammatory drug (NSAID) with a well-known toxicity profile. No carcinogenicity was seen after long-term naproxen administration in a carcinogenicity study in rats. (1)

Esomeprazole is an enantiomer of omeprazole. Hyperplasia of gastric ECL cells was seen in a carcinogenicity study of omeprazole in rats. In a carcinogenicity study in mice, no difference in tumor incidence due to omeprazole administration was seen, although the results were inconclusive. A mutation study of esomeprazole strontium yielded negative results. (2)

## Mechanism of action

Naxozol consists of naproxen and esomeprazole. Like other NSAIDs, naproxen predominantly inhibits the activity of cyclooxygenase-2 (COX-2), thereby decreasing the synthesis of prostaglandin and thromboxane from arachidonic acid, exhibiting anti-inflammatory, analgesic, and anti-pyretic effects. (3)

Esomeprazole is a proton pump inhibitor belonging to the benzimidazole family. It inhibits H+, K+-ATPase in gastric parietal cells. It is able to maintain high gastric pH for a longer period of time than other proton pump inhibitors (the maintenance time at pH >4 is 12 hours for other proton pump inhibitors, but is 16.8 hours for esomeprazole). (4)

## Clinical pharmacological study

A clinical pharmacological study on single-dose oral administration of the Naxozol tablet under fasted conditions indicated that it has a similar safety and tolerability profile as that of the control drug tablet, Vimovo. (5)

Pharmacokinetic results showed that the geometric mean ratio and 90% confidence interval of Cmax and AUClast were 0.99 (0.94 - 1.06) and 1.00 (0.98 - 1.01) for naproxen, and 0.99 (0.82 - 1.18) and 1.04 (0.91 - 1.18) for esomeprazole, both of which are within the range that allows for acceptance of biological equivalence (0.8 - 1.25), showing similar systemic exposure, and, ultimately, the pharmacokinetic equivalence of naproxen and esomeprazole between experimental and control drugs. (5)

## Summary of study subject characteristics

This study will be conducted on Korean osteoarthritis patients older than 50 years who are in need of NSAID therapy.

Osteoarthritis is an indication for this investigational product. Therefore, osteoarthritis patients were considered an appropriate subject disease group and may well benefit therapeutically from treatment with this investigational product.

Upper gastrointestinal bleeding is known to occur in 15-20% of peptic ulcer disease patients and is reported to be the most common complication of peptic ulcer disease. (6,7,8) In particular, while the incidence rate of intestinal perforation or pyloric obstruction is decreasing, that of gastrointestinal bleeds is increasing, presumably as a result of the increased use of NSAIDs, including aspirin, in elderly patients. (6,8) A fifth of elderly patients on NSAIDs develop bleeding from asymptomatic ulcers, requiring caution during history taking and physical examination. (6,9) NSAID use is also reported to increase the incidence of peptic ulcer perforation. Open perforation and resultant bacterial peritonitis are indications for emergency surgery. (6)

Therefore, combination therapy with NSAIDs and gastrointestinal protective drugs such as PPIs or misoprostol is recommended for patients on NSAIDs who have risk factors for peptic ulcer disease (including a history of peptic ulcers, age > 65 years, high-dose NSAID use, concomitant steroid use, concomitant anticoagulant use, or redundant NSAID use including low-dose aspirin) for prevention of peptic ulcers. (10) In the high risk group for peptic ulcer disease, alternative methods for gastrointestinal protection or combination therapy with a selective COX-2 inhibitor and a gastrointestinal protective agent are recommended, rather than the combination of a non-selective NSAID and a gastrointestinal protective agent (e.g. Naxozol tablet). (10) The inclusion / exclusion criteria therefore excluded high risk osteoarthritis patients as subjects in this study.

## Rationale for administration of the investigational product to the study subjects

Symptoms of dyspepsia associated with NSAID use are very common and are reported to occur in 20-25% of NSAID users. (11,12,13,14) However, 40% of NSAID users with endoscopically confirmed erosive gastritis did not show symptoms of dyspepsia, and 50% of patients with symptoms of dyspepsia have normal gastrointestinal mucosa, indicating that symptoms of dyspepsia do not predict damage to gastrointestinal mucosal lining. (11,13,14,15) When compared to non-selective NSAID use, COX-2 inhibitor use is reported to decrease the symptoms of dyspepsia associated with NSAID use. (11,16,17)

Additionally, symptoms of dyspepsia associated with non-selective NSAID use can be reduced with PPIs. PPIs such as omeprazole or esomeprazole are reported to be more effective in symptomatic improvement of dyspepsia than ranitidine or misoprostol. (13,14,18,19)

Therefore, to adequately consider both treatment-related efficacy and safety, either Naxozol tablets or a COX-2 inhibitor were administered to the osteoarthritis patients participating in this study.

## Summary of the efficacy and safety of the investigational product

A clinical study was conducted on the Naxozol tablet, the investigational product for this study. Safety information, including adverse event incidence, is included in Section 5.3.4.1.

In other clinical trials of naproxen and esomeprazole combination drugs, the combination of the two compounds reduced the incidence of gastrointestinal bleeding, upper gastrointestinal adverse events, and the discontinuation rate due to duodenal ulcers, when compared to naproxen alone. (20)

In addition, the long-term safety of combination therapy with the two drugs was confirmed in a 12-month clinical study. (21)

## Experience with the investigational product

The investigational product in this study, the Naxozol tablet, has been used for the treatment of musculoskeletal diseases including osteoarthritis, the indication in this study, since its approval in August 2013.

## Discussion of the risk-benefit of the administration of the investigational product

This study is a phase 4 clinical study on osteoarthritis patients requiring NSAID administration.

The investigational product, the Naxozol tablet, is a slow-release tablet that contains naproxen, an NSAID, and esomeprazole, a PPI.

The clinical efficacy of NSAIDs in pain control in osteoarthritis patients is well accepted. Naproxen, an NSAID, and esomeprazole, a PPI, are both widely used and are safe drugs for pain improvement in osteoarthritis patients and for treatment of gastrointestinal bleeding or symptoms of dyspepsia associated with NSAID use, respectively.

The American College of Gastroenterology (ACG) guideline also recommends co-administration of PPI with NSAID use in patients with a moderate risk of gastrointestinal bleeding. (10)

Furthermore, in conducting this study, the contraindications for the investigational product have already been reflected in the inclusion / exclusion criteria, ensuring exclusion of high risk groups from the study.

In conclusion, a 12-week administration of Naxozol to osteoarthritis patients would not greatly increase the risk of harm to subjects if preceded by appropriate subject selection and followed-up by monitoring of adverse events. The safety of a 12-week oral administration within the given dose range is expected to be satisfactory with careful monitoring of adverse events and vital signs.

## Discussion on study design

This study is a prospective, randomized, double-blind, double-placebo, active-controlled, two-arm parallel, multicenter study. The duration of treatment and observation per subject is 12 weeks, during and after which the safety and efficacy will be evaluated. This duration is considered adequate to evaluate the safety and efficacy outcomes of Naxozol. Relevant discussion and the rationale for other parts of the study design are included in Section 4.2.

## Conclusion

This study is expected to provide the basis for the efficacy and safety of Naxozol in the treatment and prevention of gastrointestinal dysfunction. This study will be conducted in accordance with protocol, good clinical practice (GCP), and other local legislations and regulations.

# Purpose

The purpose of this study was to evaluate the safety and efficacy of the investigational product, the Naxozol tablet, in osteoarthritis patients over 50 years of age after 12 weeks of oral administration.

**Primary purpose**

- To prove non-inferiority of the gastroprotective effect of a 12-week administration of Naxozol in comparison to that of celecoxib in osteoarthritis patients, as evaluated by the Leeds Dyspepsia Questionnaire (LDQ).

**Secondary purpose**

- To compare the gastroprotective effect, as evaluated by the Gastrointestinal Symptom Rating Scale (GSRS), of 12 weeks of treatment with Naxozol or celecoxib in osteoarthritis patients.
- To compare the incidence rate of gastrointestinal (GI) adverse events as well as the drug discontinuation rate due to gastrointestinal (GI) adverse events during 12 weeks of treatment with Naxozol or celecoxib in osteoarthritis patients.
- To compare the pain improvement effect, as evaluated by the visual analogue scale (VAS), after 12 weeks of treatment with Naxozol or celecoxib in osteoarthritis patients.
- To compare the improvement in quality of life (QoL), as evaluated by EQ-5D, after 12 weeks of treatment with Naxozol or celecoxib in osteoarthritis patients.
- To compare patient compliance during 12 weeks of treatment with Naxozol or celecoxib in osteoarthritis patients.
- To compare overall safety during 12 weeks of treatment with Naxozol or celecoxib in osteoarthritis patients.

# Plan

## General study methodology and plan

This is a prospective, randomized, double-blind, double-placebo, active-controlled, two-arm, parallel, multicenter, phase 4 study. Subjects eligible for this study will be randomized on Day 1 into either the experimental or control groups, to receive either two tablets of Naxozol or 1 capsule of Celebrex per day, respectively, for 12 weeks.

#### Dosage and administration of the investigational product

Refer to Section 5.1.1 for dosage and administration of the investigational product. For drugs contraindicated for co-administration, warnings, and packaging and distribution of the investigational product, refer to Sections 5.3 and 5.4.

#### Primary outcome

The primary outcome of the study is to prove non-inferiority of the gastroprotective effect of a 12-week administration of Naxozol in comparison to that of celecoxib in osteoarthritis patients, as evaluated by the Leeds Dyspepsia Questionnaire (LDQ).

#### Secondary outcome

Secondary outcomes are listed in Section 7.3.2 and Section 7.3.3. Secondary outcomes evaluate protection against gastrointestinal dysfunction, pain improvement, quality of life improvement, compliance, and safety.

#### Subjects

This study is conducted on osteoarthritis patients who have agreed to participate in the study, meet inclusion criteria, and do not meet any of the exclusion criteria. The investigator must carefully review a potential subject’s eligibility at the time of enrolment and fully consider the purpose of this study, as well as the rights and health status of the subject. Individuals unable to sign the consent form voluntarily cannot be included in the study. In particular, vulnerable volunteers must be selected with great care and proven feasibility for participation. Inclusion criteria are listed in Section 4.3.

#### Process and evaluation of the study

The screening and following scheduled visits will be conducted on a minimum 106 volunteers from 10 clinical trial sites (subject to change) after written consent is obtained. Information regarding subject allocation (Section 4.4.2), subject drop-out (Section 4.5), and study termination (Section 4.9) is discussed in each respective section.

Refer to ‘Section 6. Study process and evaluation’ for other study procedures, observation categories, and evaluation categories related to this study.

All observations, including all laboratory tests, will be conducted at each clinical trial site according to the pre-specified schedule. At the end of the study, all subjects must physically visit the study sites.

For subjects who drop out early, tests and evaluations corresponding to Visit 3 must be conducted when possible. Subjects who have dropped out cannot be substituted (refer to Section 4.6). If there are continued adverse events, additional visits may be conducted (refer to Section 6.1).

#### Level and method of blinding

- Double-blind.
- This is a double-blind study. Both investigator and subject will be blinded to study group allocation. Hence, they will be blinded as to whether the subject will receive the experimental drug or the control drug. The blinding method and emergency unblinding will be discussed in Section 5.8 ‘Blinding’ and Section 5.9 ‘Emergency unblinding’.
- A placebo will be supplied for each of the experimental (Naxozol tablet) and control (Celebrex capsule) drugs, with the same formulation and packaging so that both investigator and subject cannot distinguish between the active and placebo drugs. This will ensure that the blinding of both investigator and subject to the administered drug is maintained.

#### Treatment-related design

- Multi-dose, parallel treatment group.
- This is a multi-dose, parallel treatment group study. To be selected as a subject of this study, patients must score greater than 40 mm on the pain VAS. The LDQ score on symptoms of dyspepsia associated with NSAID use is expected to decrease after 12 weeks of administration, and, if a cross-over design was applied, the subject’s baseline may not return to a value appropriate for selection criteria after the first round of treatment and before the second round. In addition, gastrointestinal adverse events associated with NSAID use, a secondary outcome of this study, may not improve during the washout period in a cross-over study. Therefore, a parallel design was deemed more appropriate than a cross-over design.

#### Control group

- Active control.
- This study will use the Celebrex capsule as an active control.

#### Subject allocation

Subject allocation is discussed in Section 4.4.2, and randomization is discussed in Section 7.2. After allocation, each subject is assigned a serial number that is used for their identification throughout the duration of the study. A serial number cannot be reused during the study. Stratification recruitment will not be conducted during the allocation. Subjects will be allocated to one of two groups in total: 53 in the experimental group and 53 in the control group.

#### Expected duration of the study

- The expected duration of the subject enrolment period is approximately 21 months.
- The expected duration of the study is approximately 12 weeks from the study initiation date.
- The planned treatment and evaluation period for each subject consists of one day of screening and washout (day of administration) and 12 weeks of treatment and follow-up.
- Each subject will undergo 1 screening visit, 1 randomization and investigational product distribution visit, and 1 follow-up visit.

The general structure of the study is as follows (Figure 1):

#### Figure 1. General structure of the study

| **V1**  **Screening / Washout**  **(D^-^14 - 1)** | **V2**  **Randomization /**  **Investigational product distribution**  **(D1)** | | **V3**  **(End of study)**  **(D84±7)** |
| --- | --- | --- | --- |
| **Screening/ Washout** | | **Drug administration and follow-up visit (12 weeks)** | |

## Review and discussion of the study design

This is a prospective, randomized, double-blind, double-placebo, active-controlled, two-arm, parallel, multicenter, phase 4 study.

## Review of study design

This is a prospective, randomized, double-blind, double-placebo, active-controlled, two-arm, parallel, multicenter, phase 4 study that aims to evaluate non-inferiority of the gastroprotective effect of a 12-week administration of Naxozol tablets in comparison to that of Celebrex capsules in osteoarthritis patients, as evaluated by the Leeds Dyspepsia Questionnaire (LDQ). To reduce investigator- or subject-induced bias in the evaluation of gastrointestinal symptoms, efficacy, or adverse events, a randomized design was used.

## Review of treatment duration

The total duration of treatment per subject is 12-14 weeks, including a screening period (maximum 14 days) and a treatment and evaluation period (12 weeks). The 12-week treatment period is deemed sufficient to observe the change in the LDQ score, the primary outcome.

In a prospective study that compared the symptomatic improvement of dyspepsia between placebo and itopride in functional dyspepsia patients, a change in the LDQ score was observed after 8 weeks of administration. (22) In another study that evaluated dyspepsia in rheumatoid arthritis or osteoarthritis patients using a severity of dyspepsia assessment (SODA) scale instead of the LDQ, symptoms of dyspepsia were evaluated after 12 weeks of treatment. (23)

In conclusion, the 12-week treatment period planned for this study was deemed sufficient to evaluate the efficacy of experimental drug.

## Dose selection and rationale

**Selected dose:** Either 2 Naxozol tablets per day or 1 control drug capsule per day will be administered orally for 12 weeks in order to observe the change in the LDQ score.

The doses used in this study, 1 tablet twice a day for the experimental drug and 1 capsule once a day for the control drug, are both locally approved dosages for osteoarthritis treatment.

## Review of the control drug selection

This is an active-controlled study that uses the Celebrex capsule as the control drug. Celebrex is a selective COX-2 inhibitor, its use resulting in fewer symptoms of dyspepsia associated with NSAID use than with the use of a non-selective NSAID. (11,16,17)

Therefore, the Celebrex capsule was deemed an appropriate control drug for a study regarding dyspepsia symptoms associated with NSAID use.

## Review of the study group allocation

This is a randomized study. In order to reduce bias from the investigator or subject during the safety and efficacy evaluation, this study was designed as a double-blind, randomized study. The randomized design will minimize the effect of baseline characteristics on the results of the study. (24) There will be no stratification during study group allocation.

## Inclusion of special populations as subjects

This study may include elderly subjects, but will not include children, pregnant or lactating women, or other special population groups. Advanced age is a risk factor for gastrointestinal ulceration and bleeding associated with NSAID use. This study will exclude elderly subjects who have other risk factors (including a history of peptic ulcers with or without complications, NSAID use other than low-dose aspirin, and / or anticoagulant or steroid use) through its inclusion / exclusion criteria and drugs contraindicated for co-administration during this study.

## Subject selection

This study will be conducted on osteoarthritis patients aged 50 years or over with a pain VAS score of 40 mm or more.

## Inclusion criteria

- 1. Subjects of Korean nationality who voluntarily decide to participate in the study and provide written consent after receiving and understanding a detailed description and explanation of the study.
  2. Men and women aged 50 years or older at the time of consent.
  3. Persons who are available for follow-up throughout the study duration and are able to perform due tasks for the study, such as reading and filling out EQ-5D and VAS surveys.
  4. Persons with a confirmed medical history of physically symptomatic osteoarthritis who score 40 mm or greater on the pain VAS performed at V2.
  5. Persons deemed appropriate for the study based on the screening results (NB: subjects can be included at the discretion of the investigator even if laboratory test results are outside the reference ranges).

## Exclusion criteria

1. Persons who are participating in a clinical study or have participated in a clinical study within the last 30 days (participation is based on the date of final investigational product administration).
2. Persons with a history of abuse of alcohol or other drugs within 6 months prior to screening.
3. Persons who drink 21 standard drinks or more per week (generally, 55 mL of soju is equated to 200 mL of beer or 30 mL of spirits).
4. Persons with a history of peptic ulcer disease with complications (bleeding, perforation, penetration, and gastric outlet obstruction) within the last 5 years.
5. Persons with a history of peptic ulcer disease without complications within 6 months prior to screening visit or persons with active peptic ulcer disease.
6. Persons who have been infected with helicobacter but did not undergo eradication therapy.
7. Persons with a known history of gastroesophageal reflux disease.
8. Persons with a confirmed diagnosis or clinical suspicion of the following arthropathies that may affect safety and efficacy evaluation: septic arthritis, inflammatory arthropathy such as rheumatoid arthritis, gout, recurrent pseudopain, Paget’s disease, fracture, ochronosis, acromegaly, hemochromatosis, Wilson’s disease, primary osteomalacia, and Ehlers Danlos Syndrome and other genetic collagen disorders.
9. Persons with surgery planned during the study period.
10. Persons with a history of gastrointestinal cancer.
11. Persons with a GI disorder due to a malfunction in drug absorption.
12. Persons with gastrointestinal bleeding, cerebrovascular bleeding, other bleeding disorders, or severe hematological disorders.
13. Persons with moderate to severe hepatic impairment (Child Pugh Class II or higher).
14. Persons with a history of severe heart failure or coronary artery bypass graft (CABG).
15. Persons with severe renal impairment (CrCl <30 mL / min).
16. Persons with severe hypertension (SBP ≥160 mmHg or DBP ≥100 mmHg) refractory to treatment.
17. Persons with a history of allergy to components of the investigational product.
18. Persons with hypersensitivity or allergy to other NSAIDs or PPIs.
19. Subjects who received contraindicated drug or therapy within a pre-specified period prior to study participation (Section 5.3.2).
20. Patients who are receiving contraindicated co-administration drug (Section 5.3.3).
21. Patients who have a history of surgery on the osteoarthritic joint within the past 1 year.
22. Women who are pregnant or breastfeeding, or women of childbearing age who are not using appropriate contraception (condom, diaphragm, intrauterine contraceptive device, or hormonal contraceptive drug, used from 21 days prior to investigational product administration, at least, or male vasectomy); this clause does not apply to menopausal women.
23. Persons deemed inappropriate for study enrollment by the investigator due to clinically relevant medical or psychiatric conditions.
    1. **Subject allocation/investigational product administration**

Subjects who meet the inclusion / exclusion criteria and have given written consent before study participation will be randomly allocated to each study group to receive the investigational products (refer to Section 7.2).

## Assignment of screening and subject numbers

A screening code number (S + site initial + XXX) will be assigned to each volunteer who has provided written consent to participate in the study in the order of their screening site visit. The screening code will start from S + site initial + 001 and will be assigned as a serial number. Once assigned, a screening code cannot be assigned to another subject, and each subject will receive only one screening code. Subjects participating in this study who successfully complete screening tests will undergo randomization to experimental or control groups on Day 1 and will receive a subject code number (R + site initial + XXX). The subject codes will start from RX001 at each site and will have a specific pattern. For example, in ‘RB006’, ‘R’ means randomized, ‘B’ is the site initial, and the three-digit number, ‘006’, means that this subject is the 6^th^ patient to be randomized at site B. Each subject code assigned to a subject will be used as the subject identification code until study termination. Once assigned, a subject code cannot be assigned to another subject. Each subject will receive only one subject code.

## Study group allocation

At the screening visit, a screening code will be assigned to each subject after written consent and successful completion of the screening tests. Subjects who fulfill the inclusion criteria will be assigned to a treatment at Visit 2. The subjects will be allocated to experimental or control groups, and the investigational products will be administered. After allocation, each subject will be assigned a subject code, generated by the sponsor, contract research organization, or persons entrusted by them for computer-based code generation. The assigned subject code will be recorded on the case report form, and the subject will be identified using age and the subject identification code for the remainder of the study. The subject code cannot be re-used during the study.

This study is a parallel-group study. The number of subjects allocated to the experimental and control groups is as follows:

#### Table 1. Treatment groups and number of subjects per group

|  | Dose | Number of subjects | Administered drug |
| --- | --- | --- | --- |
| Experimental | 1 tablet, twice a day | 53 | Naxozol tablet |
| Control | 1 capsule, once daily | 53 | Celebrex capsule |

## Subject withdrawal criteria

Withdrawal of a subject from the study, temporary discontinuation of investigational product administration, early termination of the study, or closure of a study site may occur due to personal, medical, or administrative reasons as listed below:

## Study withdrawal

A subject can withdraw from the study at any time and is not obliged to reveal the reason. A subject must be withdrawn from the study if one of the situations below arises:

- - Withdrawal of consent by the subject or the subject’s legal proxy.
  - The investigator’s assessment that continued participation in the study is harmful to the subject.
  - Adverse events too serious to continue the study (CTCAE grade 4 or 5) or difficulty with study participation as per the investigator’s assessment.
  - Serious protocol violation as per the sponsor or investigator’s assessment.
  - Unfit to continue in the study as per the sponsor or investigator’s assessment.
  - A subject’s wish to withdraw from the study for other reasons.

In cases of serious protocol violation as listed below, the corresponding subject must be excluded from the analysis (PP excepted):

- - Need for cessation of study participation due to the possibility of biasing the efficacy or safety evaluation of the experimental drug, or the violation of inclusion / exclusion criteria that can affect the safety of a subject, as per the investigator or sponsor’s assessment.
  - Notification by a subject of the use of another drug or treatment for the study’s target disease during the study period, and the investigator’s conclusion that the subject has no intention of ceasing the use of such drug or treatment.
  - The use or administration of drugs contraindicated for co-administration with the investigational product, as stated in Section [5.3.3](#_bookmark68).
  - Subjects who did not undergo washout for specified drugs before randomization.
  - Incorrect randomization.
  - Less than 80% compliance rate.

If the follow-up of a withdrawn subject is not possible, the investigator must make an effort to confirm the address and health status of subject. If a subject is withdrawn from the study, all efforts must be made to record, in full detail, the reason and situation leading to their withdrawal. If a subject withdraws early, the evaluation and treatment corresponding to Visit 3 must be conducted, when possible in addition to an appropriate assessment to determine the reason for early withdrawal, including any adverse reactions to the investigational products (refer to Section 6.1). The reason for withdrawal must be recorded in case report form for all cases.

## Discontinuation of investigational product use

Administration of the investigational product must be discontinued if any of the below situations arise during the study:

- - Fulfillment of exclusion criteria that can affect the safety of a subject and necessitate discontinuation of the investigational product administration, as per the clinical assessment by the investigator or sponsor.
  - Adverse events for which temporary or permanent discontinuation of the investigational products is necessary, as per the assessment of the investigator or subject.
  - Administration of drugs contraindicated for co-administration that require discontinuation of the investigational products, as stated in Section 5.3.3.
  - Serious non-compliance to administration of the investigational product.

The investigator must ensure that the administration of the investigational products is as planned. If investigational product use is discontinued by a subject, all observations necessary and possible must be made and recorded, as in the case of study withdrawal. In all cases of discontinuation of the investigational product, the reason, situation, and duration must be recorded in case report form.

## Substitution of subject

This study does not allow for the substitution of a withdrawn subject.

## Addition of new dosage groups

This study does not allow for additional dosage groups.

## Early termination of study

The study can be terminated early in the following cases (in which there is new information regarding negative effects of the investigational products on the risk / benefit ratio for the subjects):

- - Evidence of lack of efficacy.
  - New and important drug-related adverse events or an unexpectedly high incidence rate or severity of one or more previously known adverse events.
  - Other safety-related reasons.
  - An inability to medically or ethically justify continuation of the study, as per the sponsor’s assessment.
  - The rate of subject enrolment at a study site is deemed to hinder the enrolment of the required number of subjects for the study.
  - The cessation of sale or supply of the investigational products.

If this study is terminated early, a government regulatory authority or clinical study review committee will be notified according to the relevant regulations. The study can be suspended or terminated by a request from the government regulatory authority.

## Termination of study

In this study, Visit 3 is defined as the “end of study (trial) visit’, and subjects who have completed Visit 3 are defined as completed subjects. If the following situations occur, this study will not be deemed completed:

- - There are remaining subjects who are still due to receive investigational products.
  - The pre-specified visit schedule at a study site has not been completed.

If a subject misses a visit or is lost to follow-up despite reasonable requests for a visit, the corresponding subject will be deemed to have terminated the study participation. No further evaluation will be conducted after study termination.

## Other considerations

Subjects must limit any external factors that may affect laboratory test results or induce interactions with the investigational products (alcohol, excessive exercise, etc.).

#### Diet

Subjects must be fasted from 10 pm the night before the laboratory test is due to be conducted.

#### Alcohol

Subjects should abstain from a weekly alcohol intake of 21 standard drinks (3 drinks / day) during the study period and from any alcohol intake for 72 hours prior to the laboratory tests.

#### Exercise

Excessive exercise should be avoided the day before the laboratory tests, including the screening tests.

# Investigational product and other study drugs

“Investigational medicinal product” includes the experimental drug and the control drug.

## Investigational product

## Experimental drug

## Product name / code

Naxozol tablet.

## Pharmaceutical form

Rectangular, pale yellow, film-coated tablet.

## Qualitative and quantitative composition

Naproxen (USP) 500 mg, and esomeprazole strontium tetrahydrate 24.60 mg (as esomeprazole 20 mg), per tablet.

## Storage conditions

Sealed container, room temperature (1 - 30℃); shelf life: 24 months from manufacture date.

## Experimental drug placebo

Rectangular, pale yellow, film-coated tablet that does not contain the active ingredients but has the same pharmaceutical form as the experimental drug.

## Control drug

## Product name/code

Celebrex capsule.

## Pharmaceutical form

White capsule with two yellow bands containing white to pale yellow powder.

## Qualitative and quantitative composition

Celecoxib 200 mg per capsule.

## Storage conditions

Sealed container, room temperature (15 - 30℃).

## 5.1.4. Control drug placebo

White capsule with two yellow bands that does not contain the active ingredient but does contain white to pale yellow powder and has the same pharmaceutical form as the experimental drug.

## Expected dosage and administration

## Expected dosage and administration of the experimental drug

* Adult: 1 tablet twice daily (naproxen 500 mg / esomeprazole 20 mg) orally. During this study, 1 tablet of the experimental drug and 1 control drug placebo capsule will be administered concomitantly 30-60 minutes before a meal. This drug should not be split, chewed, or crushed, and should be taken whole with water. Administration 30-60 minutes before food is recommended.

- - Effect on laboratory values:

1. This drug may interfere with platelet aggregation and increase bleeding time. Caution must be taken when measuring bleeding time.
2. This drug may affect the measurement of 17-ketogenic steroids and should be temporarily discontinued 72 hours before measurement.
3. Naproxen may affect urinalysis of 5-hydroxyindoleacetic acid (5-HIAA).

## Expected dosage and administration of the control drug

* During this study, 1 capsule of the control drug and 1 experimental drug placebo tablet should be administered 30-60 minutes before a meal. The minimum recommended dose should be adjusted for each patient. This drug can be administered irrespective of food times.

1. Osteoarthritis (degenerative arthritis): 200 mg celecoxib, once daily.
2. Rheumatoid arthritis: 200 mg celecoxib, once daily.
3. Ankylosing spondylitis: 200 mg celecoxib, once daily. If no effect is seen within 6 weeks, the dose may be increased to 400 mg/day. If no effect is seen at 400 mg / day within 6 weeks, another treatment should be considered.
4. Acute pain and primary menstrual pain: the initial recommended dose is 400 mg celecoxib. An additional 200 mg may be given on the first day, if needed. From the second day of administration, 200 mg per administration, once a day, is given as a recommended dose when needed.
5. Hepatic impairment: the dose should be reduced to approximately 50% of the daily recommended dose in patients with moderate hepatic impairment (Child-Pugh class II).
   - Effect on laboratory values:
6. Patients on long-term NSAID therapy must undergo a regular complete blood count, physical examination, and blood chemistry test. If abnormal liver function or renal function persists or worsens, this drug must be discontinued.
7. As with other NSAIDs, in clinical studies BUN levels were elevated more frequently in patients on celecoxib than in those given placebo, though without confirmed clinical significance.

## Other drugs used in the study

## Concomitant pharmacological therapy and treatment

## Supplementary medication

If dyspepsia due to the investigational product is severe, the following supplementary medication is allowed for the subject:

- - - - - Almagate (Hanmi Almagate 500 mg tablet); a maximum of 6 tablets / day and 30 - 50 tablets in total per person.

For the above medication, the dose and duration of administration must be recorded on the case report form.

- - - - - Criteria for administration of the supplementary medication:

For the entire duration of the study, if the symptoms of dyspepsia become unacceptable, a maximum of 6 Almagate (Hanmi Almagate 500 mg) tablets over 24 hours per subject will be allowed for additional gastrointestinal protective therapy. For this, an adequate amount of supplementary medication should be distributed to the randomized subjects. Subjects should be educated not to exceed 6 Almagate (Hanmi Almagate 500 mg tablet) tablets over 24 hours. The amount of supplementary medication used must be recorded in the subject file, and the remaining amount should be returned for confirmation of the amount used. The above criteria must be conveyed to the subjects by the investigator.

## Rescue medication

The subjects are allowed to take the following rescue medications if pain control is difficult with the investigational products:

 Acetaminophen (Hanmi Susphen ER 650 mg tablet); a maximum of 3 g / day and 30 - 50 tablets per person.

For the above drug, the amount and duration used should be recorded on a case report form.

 Administration criteria for the rescue medication:

Subjects who experience an unacceptable degree of pain during the study period can be given acetaminophen 650 mg for additional pain control. For this, subjects must be given the appropriate amount of rescue medication after randomization. Subjects must be educated not to take more than 3 g / day of acetaminophen 650 mg. The amount of rescue medication used should be recorded in the subject file, and the remaining amount must be returned at the end of the study to confirm the total amount used. The above information must be given to the subjects by the investigator.

## Drugs or therapies contraindicated prior to study participation

Subjects who received the following medications for the periods listed below cannot participate in the study:

1. NSAIDs (LDA [≤325 mg / day low dose aspirin] excluded) within 7 days prior to administration of the investigational product.
2. Gastroprotective agents (including H2 blockers, PPIs, misoprostol, etc.) within 7 days prior to administration of the investigational product.
3. IV or oral corticosteroids (oral corticosteroids corresponding to ≤7.5 mg / day prednisone, topical ointments, eyedrops, and nasal agents are excluded) within 7 days prior to investigational product administration.
4. Intraarticular or intramuscular corticosteroids or hyaluronic acid within 8 weeks prior to administration of the investigational product.
5. Glucosamine and / or chondroitin sulfate within 7 days prior to administration of the investigational product.
6. Lithium within 7 days prior to administration of the investigational product.
7. Antiretrovirals (atazanavir, atazanavir sulfate) within 7 days prior to administration of the investigational product.

## Drugs or therapies contraindicated during study participation

Drugs other than investigational products, especially one of the drugs listed below, should not be administered during the study period, on principle:

1. NSAIDs (LDA [<325 mg/day low dose aspirin] excluded) other than investigational products.
2. Other gastroprotective drugs (H2 blockers, PPIs, misoprostol, etc.).
3. IV or oral corticosteroids (oral corticosteroids corresponding to ≤7.5 mg / day prednisone, topical ointments, eyedrops, nasal agents excluded).
4. Intraarticular or intramuscular corticosteroids or hyaluronic acid.
5. Glucosamine and / or chondroitin sulfate.
6. Lithium.
7. Antiretrovirals (atazanavir, atazanavir sulfate).

All drugs deemed necessary by the investigator, as per the investigator’s medical opinion, can be given. However, if a drug administered without an investigator’s assessment is deemed to have the capacity to influence the safety and efficacy evaluation of this study, the corresponding subject will be excluded from PP analysis. All drugs administered, and their dosage, route of administration, and reason for administration, must be recorded in a case report form signed by the investigator.

## Precautions regarding the experimental drug

## Adverse events associated with the experimental drug

1. A comparison of the safety data for this drug and of that for each of naproxen and esomeprazole, the active ingredients in this drug, indicated that there was no novel safety information.
2. Adverse events associated with this drug in a clinical study have been classified as shown below, according to frequency and System Organ Class (SOC). Frequency is defined as follows: very common (>1/10), common (>1/100, <1/10), uncommon (>1/1,000, <1/100), rare (>1/10,000, <1/1,000), very rare (<1/10,000), and unknown (cannot be measured in usable data).

|  | Very common | Common | Uncommon | Rare |
| --- | --- | --- | --- | --- |
| Infection |  |  | Infection | Diverticulitis |
| Hematological or lymphatic disorder |  |  |  | Eosinophilia, leukopenia |
| Immune disorder |  |  |  | Hypersensitivity |
| Metabolic and nutritional disorder |  |  | Decreased appetite | Fluid retention, hyperkalemia, hyperuricemia |
| Psychiatric disorder |  |  | Anxiety, depression, insomnia | Confusion, abnormal dreams |
| Neurological disorder |  | Dizziness, headache, dysgeusia | Cognitive disorder, syncope | Somnolence, tremor |
| Ear and labyrinth disorder |  |  | Tinnitus, vertigo |  |
| Cardiovascular disorder |  |  | Arrhythmia, tachycardia | Myocardial infarction, tachycardia |
| Vascular disorder |  | Hypertension |  |  |
| Respiratory, chest, and mediastinal disorder |  |  | Asthma, bronchospasm, dyspnea |  |
| Gastrointestinal disorder | Dyspepsia | Abdominal pain, constipation, diarrhea, esophagitis, flatulence, gastroduodenal ulcer*, gastritis, nausea, vomiting | Gastrointestinal bleeding, oral mucositis | Glossitis, hematemesis, rectal bleeding |
| Skin and subcutaneous tissue disorder |  | Rash | Dermatitis, hyperhidrosis, pruritis, hives | Alopecia, ecchymosis |
| Musculoskeletal and connective tissue disorder |  | Arthralgia | Myalgia |  |
| Renal and urological disorder |  |  |  | Proteinuria, renal failure |
| Reproductive and breast disorder |  |  |  | Menstrual disorder |
| Systemic disorder |  | Edema | Lethargy, tiredness, fever |  |
| Laboratory disorder |  |  | Abnormal liver function, elevated serum creatinine |  |

*: Confirmed by planned general gastroscopy.

1. Adverse events shown in clinical and post-marketing studies of naproxen are as follows:
2. Hypersensitivity: symptoms of shock (dyspnea, hypotension, bradycardia, diaphoresis, edema, etc), PIE syndrome (pulmonary infiltration with eosinophilia: fever, wheeze, cough, sputum), anaphylactic reaction, hives, rash, and pruritis.
3. Gastrointestinal: hematemesis, peptic ulcers, perforation, gastric discomfort, gastric pain, abdominal pain, nausea, vomiting, decreased appetite, dyspepsia, heartburn, diarrhea, constipation, stomatitis, distension, malena, thirst, esophagitis, gastritis, glossitis, belching, flatulence, coffee-ground vomit, pancreatitis, colitis, exacerbation of inflammatory bowel disease (ulcerative colitis, Crohn’s disease), and ulcerative stomatitis.
4. Hematological: agranulocytosis, neutrophilia, granulocytopenia, pancytopenia, leukopenia, thrombocytopenia, aplastic anemia, hemolytic anemia, platelet dysfunction (prolongation of the bleeding time), and lymphadenitis.
5. Hepatic: GOT and GPT elevation, jaundice, hepatitis, liver failure, and cholestatis.
6. Renal: renal dysfunction, glomerular nephritis, hematuria, interstitial nephritis, renal syndrome, renal papillary necrosis, proteinuria, renal failure, and oliguria / polyuria.
7. Central nervous system:

- Somnolence and cognitive dysfunction; rarely dizziness, headache, paresthesia, decreased libido, fatigue, depression, dream abnormalities, inability to concentrate, disorientation, insomnia, myalgia, muscle weakness, agitation, anxiety, confusion, hallucination, nervousness, lethargy, and myasthenia gravis.

- Aseptic meningitis (discontinue the drug and consult physicians if the following symptoms occur: severe headache, nausea, vomiting, neck stiffness, fever, and / or an altered level of consciousness).

1. Respiratory: dyspnea, asthma, bronchospasm, eosinophilic pneumonitis, pneumonia, pulmonary edema, and decreased respiration.
2. Dermatological: pruritis, ecchymosis, rash, eczema, sweating, purpura, photosensitivity, alopecia, redness, toxic epidermal necrolysis (TEN), erythema multiforme, erythema nodosum, fixed drug eruption, lichen sclerosis, porphyria cutanea tarda, exfoliative dermatitis, and bullous skin reaction.
3. Sensory: tinnitus, hearing disorder, and vision disorder.
4. Metabolic and nutritional: edema, fluid retention, hyperglycemia, hypoglycemia, hyperkalemia, and hyperuricemia.
5. Infection: diverticulitis, infection, and sepsis.
6. Other: angioneurotic edema, palpitations, congestive heart failure, infertility, menstrual disorder, chills, fever, vasculitis, and serum creatinine elevation.
7. Adverse events shown in clinical and post-marketing studies of enteric coated esomeprazole are as follows (no dose relationship was seen):

|  | Common | Uncommon | Rare | Very rare | Not known |
| --- | --- | --- | --- | --- | --- |
| Blood and lymphatic system disorders |  |  | Leukopenia, thrombocytopenia | Agranulocytosis, pancytopenia |  |
| Immune system disorders |  |  | Hypersensitivity reactions e.g. fever, angioedema and anaphylactic reaction/shock |  |  |
| Metabolism and nutritional disorders |  | Peripheral edema | Hyponatremia | Hypomagnesemia |  |
| Psychiatric disorders |  | Insomnia | Agitation, confusion, depression | Aggression, hallucinations |  |
| Nervous system disorders | Headache | Dizziness, paresthesia, somnolence | Taste disturbance |  |  |
| Eye disorders |  |  | Blurred vision |  |  |
| Ear and labyrinths disorder |  | Vertigo |  |  |  |
| Respiratory, thoracic, and mediastinal disorders |  |  | Bronchospasm |  |  |
| Gastrointestinal disorders | Abdominal pain, diarrhea, flatulence, nausea / vomiting, constipation | Dry mouth | Stomatitis, gastrointestinal candidiasis | Microscopic colitis | *C. difficile* diarrhea |
| Hepatobiliary disorders |  | Increased liver enzymes | Hepatitis with or without jaundice | Hepatic failure, hepatic encephalopathy in patients with preexisting liver disease |  |
| Skin and subcutaneous tissue disorders |  | Dermatitis, pruritus, urticaria, rash | Alopecia, photosensitivity | Erythema multiforme, Stevens-Johnson syndrome, toxic epidermal necrolysis (TEN) (partially fatal) |  |
| Musculoskeletal and connective tissue disorders |  |  | Arthralgia, myalgia | Muscular weakness |  |
| Renal and urinary disorders |  |  |  | Interstitial nephritis |  |
| Reproductive and breast disorders |  |  |  | Gynecomastia |  |
| General and administration site disorders |  |  | Malaise, increased sweating |  |  |

## Contraindications for the experimental drug

The following are contraindications for this drug:

1. Known hypersensitivity to naproxen, esomeprazole, any component of this drug, or substituted benzimidazoles.
2. A history of asthma, rhinitis, nasal polyps, urticaria, or other allergic-type reactions after taking aspirin or other NSAIDs, including COX-2 inhibitors (fatal and severe anaphylactic reaction has rarely been reported in such patients after NSAID use).
3. Active peptic ulcer disease.
4. Gastrointestinal bleeding, cerebrovascular bleeding, other bleeding disorders, or a severe hematological disorder.
5. Severe hepatic dysfunction (e.g. Child-Pugh class C).
6. Severe cardiac failure.
7. Severe renal failure (CrCl <30 mL / min).
8. Severe hypertension.
9. Use during the peri-operative period of coronary artery bypass graft (CABG) surgery.
10. Use during the last 3 months of pregnancy.
11. Atazanavir or nelfinavir use.

## Warnings for use of the experimental drug

Warnings for use of the experimental drug are as follows:

1. If a patient who regularly drinks 3 or more standard drinks / day requires this drug or another antipyretic analgesic agent, a physician or pharmacist must be consulted. Such a person may be at an increased risk of gastrointestinal bleeding.
2. Cardiovascular risk: Use of NSAIDs, including this drug, may increase the risk of major cardiovascular events, myocardial infarction, and stroke, all of which can be fatal. This risk can increase with increasing duration of administration, and may be increased in patients with pre-existing cardiovascular disease or risk factors. The physician and patient must both carefully monitor for symptoms of such cardiovascular events, even without a history of cardiovascular disease. The patient must be aware of signs and / or symptoms of major cardiovascular toxicity and the appropriate response to take should such symptoms occur.
3. Gastrointestinal risk: Use of NSAIDs, including this drug, can increase the risk of gastrointestinal adverse events, including gastrointestinal bleeding, ulcers, or perforation, all of which can be fatal. This drug significantly reduced the incidence of GI ulcers compared to naproxen alone, but ulcers and their complications may still occur. Such adverse events can occur without warning symptoms during treatment. Elderly patients may be at an increased risk of such serious GI adverse events. A longer duration of treatment can increase the risk of GI adverse events, but short-term administration does not rule out such risks. Patients must be carefully monitored for signs or symptoms of GI ulcers or bleeding while on this drug. If serious GI adverse events are suspected, immediately perform additional assessments and provide treatment. It may be necessary to discontinue NSAID use until there is a complete resolution of serious GI adverse events. In high-risk groups, alternative treatments that are not related to NSAIDs should be considered.

## Precautions for the use of the experimental drug

#### Administration with caution

Administer cautiously to:

1. Patients with a history of peptic ulcers.
2. Patients with hematological disorders or a history of hematological disorders.
3. Patients with bleeding diathesis (which may decrease platelet function).
4. Patients with liver dysfunction or a history of liver dysfunction.
5. Patients with renal dysfunction, a history of renal dysfunction, or decreased renal blood flow.
6. Patients with cardiac dysfunction.
7. Patients with hypertension.
8. Patients with hypersensitivity.
9. Patients with bronchogenic asthma.
10. Patients with induced porphyria, systemic lupus erythematosus (SLE), or mixed connective tissue disease (MCTD).
11. Elderly patients.

#### General precautions

1. Carefully consider the potential benefits and risks of this drug and other treatment options before deciding to use this drug. When administering this drug, adequately monitor progress and use the lowest effective dose for an appropriate amount of time.
2. It must be noted that NSAID therapy is a symptomatic, rather than a curative, treatment.
3. Long-term (especially 1 year or longer) therapy with this drug must be monitored regularly by clinical tests (urine analysis, complete blood count, chemistry profile, liver function test, eye test, etc.). If abnormalities are noted, appropriate management, including dose reduction or temporary discontinuation, must be implemented. If clinical signs and / or systemic manifestations (e.g. eosinophilia, rash, etc.) consistent with liver disease or renal disease develop, or abnormal liver function tests or renal function tests persist or worsen, this drug should be discontinued.
4. Patients must be monitored sufficiently for any adverse events. Excessive hypothermia, lethargy, or cool periphery can occur. Therefore, the patient’s condition must be carefully noted, especially in elderly patients or patients with a wasting disease. Debilitated patients should be given the lowest dose possible and monitored for incidence of adverse events.
5. Based on the pharmacological properties of this drug, any signs and symptoms of fever or inflammation may be muted, delaying the diagnosis of infectious complication under conditions of pain or non-infection.
6. Gastrointestinal effects:
7. NSAIDs should be prescribed with extreme caution in patients with a prior history of ulcerative disease or gastrointestinal bleeding. Patients with a prior history of peptic ulcers and / or gastrointestinal bleeding who use NSAIDs have a greater than 10-fold increased risk of developing a GI bleed than patients with neither of these risk factors. Other factors that increase the risk of GI bleeding in patients treated with NSAIDs include concomitant use of oral corticosteroids or anticoagulants, a longer duration of NSAID therapy, smoking, use of alcohol, older age, and poor general health status. Most spontaneous reports of fatal GI events are in elderly or debilitated patients and therefore special care should be taken in treating this population. Co-administration with misoprostol or a proton pump inhibitor must be considered in such patients.
8. If GI bleeding or ulcers occur during administration of this drug, this drug must be discontinued.
9. NSAIDs should be given with care to patients with a history of gastrointestinal disease (ulcerative colitis or Crohn's disease) as these conditions may be exacerbated.
10. In the presence of any alarm symptom (e.g. significant unintentional weight loss, recurrent vomiting, dysphagia, hematemesis or melaena) and when a gastric ulcer is suspected or present, malignancy should be excluded as treatment with esomeprazole magnesium may alleviate symptoms and delay diagnosis.
11. Treatment with proton pump inhibitors may slightly increase the risk of gastrointestinal infections such as *Salmonella* and *Campylobacter.*
12. Esomeprazole might reduce the absorption of vitamin B12 (cyanocobalamin) due to hypo- or achlorhydria. This should be considered in patients on long-term therapy with a risk of reduced vitamin B12 absorption or storage.

## Drug Interactions

1. Antiretrovirals:
   1. Atazanavir: Co-administration of omeprazole (40 mg once daily) with atazanavir 300 mg / ritonavir 100 mg to healthy volunteers resulted in a substantial reduction in atazanavir exposure (approximately 75% decrease in the AUC, Cmax, and Cmin). Increasing the atazanavir dose to 400 mg did not compensate for the impact of omeprazole on atazanavir exposure. Therefore, atazanavir must not be co-administered with this drug.
   2. Nelfinavir: Co-administration of omeprazole (40 mg once daily) with nelfinavir reduced the mean nelfinavir AUC, Cmax, and Cmin by 36 - 39%, and the mean AUC, Cmax, and Cmin for the pharmacologically active metabolite, M8, by 75-92%. Therefore, nelfinavir must not be co-administered with this drug.
   3. Saquinavir: Co-administration of omeprazole (40 mg once daily) with saquinavir can increase the risk of adverse events by increasing the serum concentration of saquinavir. Therefore, necessary precautions, such as dose reduction according to a patient’s individual needs, must be taken.
2. Other NSAIDs including selective cyclooxygenase-2 inhibitors: Co-administration with naproxen derivatives is not recommended, as it can increase risk of adverse events such as gastrointestinal ulcers or bleeding.
3. Hydantoin antiepileptics, sulfonamides, and sulphonylurea hypoglycemic agents: Co-administration with these drugs can increase their activity. Therefore, adequate monitoring and caution must be exercised when administering these drugs.
4. Propranolol and other beta-blockers: Naproxen and other NSAIDs can reduce the antihypertensive effect of propranolol and other beta-blockers.
5. ACE inhibitors (e.g. captopril):
   1. NSAIDs have been reported to reduce the antihypertensive effect of ACE inhibitors. Such interactions must be considered when co-administering naproxen and ACE inhibitors.
   2. Co-administration of naproxen with ACE inhibitors (captopril) can cause renal dysfunction and therefore they must be administered with care.
6. Loop and thiazide diuretics:
   1. Clinical studies, as well as post-marketing observations, have shown that NSAIDs can reduce the natriuretic effect of furosemide and thiazides in some patients. This response has been attributed to the inhibition of renal prostaglandin synthesis. During concomitant therapy with NSAIDs, the patient should be observed closely for signs of renal failure.
   2. This drug must be administered with care when co-administering with loop diuretics (furosemide or piretanide) and thiazide diuretics, since it can reduce their efficacy.
7. Probenecid: Probenecid given concurrently extends the half-life of naproxen in plasma by increasing its concentration. Therefore, cautious administration, such as dose reduction, is recommended.
8. Aspirin: There is no consistent evidence that concomitant use of aspirin with NSAIDs can decrease the risk of serious cardiovascular thromboembolic events. As with other NSAIDs, this drug, when concomitantly used with aspirin, can increase the risk of serious GI adverse events. Therefore, combination of the two drugs is not generally recommended.
9. Lithium: NSAID usage has caused an elevation of plasma lithium levels and a reduction in renal lithium clearance. These effects have been attributed to the inhibition of renal prostaglandin synthesis by the NSAID. Thus, when NSAIDs and lithium are administered concurrently, subjects should be observed carefully for signs of lithium toxicity and doses should be reduced when necessary.
10. Methotrexate: When given together with proton pump inhibitors, methotrexate levels have been reported to increase in some patients. Concomitant administration of methotrexate with NSAIDs may reduce the tubular secretion of methotrexate and enhance its toxicity. Therefore, this drug should not be used concomitantly with the high-dose methotrexate used for anti-cancer therapy. Co-administration with low-dose methotrexate must be given with care.
11. Coumarin-type anticoagulants (warfarin, etc.):
    1. Warfarin and NSAIDs can exert a synergetic effect on gastrointestinal bleeding. Therefore, patients on both drugs concomitantly may be at an increased risk of severe gastrointestinal bleeding compared to patients on each drug alone.
    2. When administered with this drug, the activity of the coumarin-type anticoagulant may be increased. Therefore, sufficient observation must be made before administration.
12. Zidovudine: This drug can inhibit the metabolism of zidovudine and increase its serum concentration. Therefore, caution, such as dose reduction, should be taken when administering this drug with zidovudine.
13. Nuquinolone-type antibiotic (enoxacin, etc.): Caution must be exercised, since co-administration with this drug can cause seizures.
14. Cyclosporin and tacrolimus: Co-administration of NSAIDs and cyclosporin or tacrolimus can cause renal toxicity.
15. Selective serotonin reuptake inhibitor (SSRI): NSAIDs, including selective COX-2 inhibitors, can increase the risk of GI bleeding when co-administered with SSRIS.
16. Corticosteroids: Concomitant use of corticosteroids and NSAIDs, including selective COX-2 inhibitors, increases the risk of GI bleeding.
17. Cholestyramine: Concomitant use with NSAIDs, including naproxen, may delay absorption.
18. Cardiac glycosides (e.g. digoxin): When administered concomitantly with NSAIDs, the plasma concentration of cardiac glycosides can be increased. In healthy subjects, concomitant use of digoxin and omeprazole (20 mg / day) increased the bioavailability of digoxin by up to 30%.
19. Clopidogrel: Concomitant use of omeprazole and clopidogrel decreases exposure to the active metabolite of clopidogrel and attenuates its inhibitory effect on platelet aggregation. Therefore, co-administration with esomeprazole is not recommended.
20. Drugs dependent on gastric pH for absorption: During administration of this drug, absorption of drugs dependent on gastric pH for absorption may increase or decrease due to a decrease in gastric pH. As with other inhibitors of acid secretion, or antacids, this drug may reduce the absorption of ketoconazole or itraconazole.
21. CYP2C19, CYP3A4 inhibitor / inducer and substrates:

Esomeprazole is metabolized via CYP2C19 and CYP3A4 and inhibits CYP2C19, the main metabolic enzyme.

1. The plasma concentration of drugs metabolized by CYP2C19, such as diazepam, citalopram, imipramine, clomipramine, phenytoin, and warfarin, can be decreased when used with this drug. Co-administration of 30 mg esomeprazole and diazepam, a CYP2C19 substrate, resulted in a 45% decrease in the clearance of diazepam. However, concomitant administration of 40 mg esomeprazole resulted in a 13% increase in trough plasma levels of phenytoin in epileptic patients. Monitor the plasma concentration of phenytoin when starting or discontinuing this drug.
2. Omeprazole (400 mg once daily) increased the Cmax and AUCτ of voriconazole (a CYP2C19 substrate) by 15% and 41%, respectively.
3. Concomitant administration of esomeprazole and clarithromycin, a CYP3A4 inhibitor (500 mg twice daily), resulted in a 2-fold increase in the AUC of esomeprazole.
4. Inducers of CYP2C19 or CYP3A4 (e.g. rifampicin and St. John’s Wort) can decrease the plasma concentration of esomeprazole by increasing esomeprazole metabolism.
5. Cilostazol: Omeprazole, given in doses of 40 mg daily for one week to 20 healthy subjects in a cross-over study, increased the Cmax and AUC of cilostazol by 18% and 26%, respectively. The Cmax and AUC of one of its active metabolites, 3,4-dihydrocilostazol, which has 4-7 times the activity of cilostazol, were increased by 29% and 69%, respectively.

## Special Populations

1. Pregnancy:
   1. There are no adequate and well-controlled studies of this drug in pregnant women. In animal studies, administration of naproxen during the perinatal period was associated with delayed delivery, an increased frequency of difficult delivery, and decreased fetal survival. Therefore, this drug should not be used in late pregnancy.
   2. In a study of rats in late pregnancy, constriction of the fetal ductus arteriosus was reported. As with other NSAIDs, the administration of this drug in late pregnancy may lead to early closure of the fetal ductus arteriosis. Therefore, the use of this drug should be avoided in pregnant women.
   3. The safety of this drug in pregnant women has not been established. Therefore, in women who are potentially pregnant, this drug should only be used when benefits are considered to outweigh the risk.
2. Breastfeeding:

Naproxen contained in this drug is transferred to breast milk, causing inhibition of prostaglandin synthesis. It should not be administered during lactation.

1. Pediatric population:

Administration in children under 18 is not recommended, as its safety and efficacy is not established in this population.

1. Elderly:

Generally, advanced age is associated with a higher incidence of NSAID-related GI ulcers and bleeding. Therefore, either the lowest possible dose of this drug should be administered in this population, with particular attention to adverse events, or an alternative option should be considered.

## Precautions regarding the control drug

## Adverse Events associated with the control drug

1. In placebo- or active-controlled clinical trials of Celebrex, the control drug, the discontinuation rate due to adverse events was 7.1% in the Celebrex group and 6.1% in the placebo group. The most common adverse events that led to discontinuation of this drug were dyspepsia and abdominal pain. The following adverse events, regardless of a causative relationship with Celebrex, have been reported in adults who have been administered this drug:
   1. Gastrointestinal: dyspepsia (8.8%), diarrhea (5.6%), irritable bowel syndrome, and increased frequency of bowel movement; occasional abdominal pain, upper abdominal pain, flatulence, nausea, constipation, diverticulitis, dysphagia, belching, esophagitis, gastroenteritis, gastrointestinal ulcer, gastroesophageal reflux, gastric discomfort, gastrointestinal dysfunction, hemorrhoids, hiatal hernia, hematochezia, dry mouth, stomatitis, tenesmus, dental disease, vomiting, exacerbation of gastroenteritis, and angular cheilitis; rarely intestinal obstruction, intestinal perforation, gastrointestinal bleeding, colitis with bleeding, esophageal perforation, pancreatitis, ileus alveolar osteitis after dental extraction, malena, glossitis, aphthous stomatitis, bullae of oral mucosa, oral numbness, and oral pain.
   2. Central and peripheral nervous system: headache (15.8%); occasional dizziness, leg cramps, hypertonia, hypoesthesia, migraine, neuropathic pain, neuropathy, paresthesia, and stroke; rarely aseptic meningitis, ataxia, suicide, anosmia, and ageusia.
   3. Systemic: occasional back pain, exacerbation of allergy, allergic reaction, lethargy, chest pain, fluid retention / peripheral edema, general edema, face edema, fatigue, malaise, fever, facial redness, influenza syndrome, pain, and peripheral pain; rarely sepsis, sudden death, anaphylactoid reaction, and angioedema.
   4. Psychiatric: occasional insomnia, decreased appetite, anxiety, increased appetite, depression, neurosis, somnolence, fatal intracranial bleeding, delusion, hallucination, and epilepsy exacerbation.
   5. Respiratory: upper respiratory infection (8.1%) and sinusitis (5.0%); occasional pharyngitis, rhinitis, bronchitis, bronchospasm, aggravated bronchospasm, coughing, dyspnea, laryngitis, and pneumonia; rarely epistaxis and dysphonia.
   6. Skin and appendages: bullous dermatitis and ecchymosis; occasional rash, alopecia, dermatitis, nail disease, photosensitivity reactions, pruritus, erythematous rash, maculopapular rash, skin disorders, dry skin, increased sweating, urticaria, erythema multiforme, exfoliative dermatitis, Stevens-Johnson syndrome, toxic epidermal necrolysis (Lyell syndrome), eczema, lipoma, and allergic dermatitis; rarely petechiae, dandruff, and drug rash with eosinophilia and systemic symptom (DRESS).
   7. Cardiovascular: arrhythmia, ventricular hypertrophy, malignant hypertension, hypertension, angina, unstable angina, coronary artery disease, myocardial infarction, heart failure, congestive heart failure, palpitations, tachycardia, stroke, aortic regurgitation, hematoma, and sinus bradycardia; rarely syncope, ventricular fibrillation, pulmonary embolism, cerebrovascular spasm, peripheral gangrene, thrombophlebitis, phlebitis, arrhythmic embolism, and cerebral hemorrhage.
   8. Infection: upper respiratory infection, erysipelas, wound infection, and gingival infection; occasional herpes simplex, herpes zoster, bacterial infection, fungal infection, soft tissue infection, viral infection, moniliasis, genital moniliasis, and otitis media.
   9. Reproductive: breast tenderness and menopausal symptoms; occasional breast fibromatosis, thoracic neoplasm (female), chest pain (female), menstrual pain, menstrual disorder, cervical bleeding, vaginitis, prostate disorder, and ovarian cysts.
   10. Ear: occasional hearing loss, ear pain, tinnitus, and hearing difficulties.
   11. Hepatobiliary: occasional liver dysfunction, ALT elevation, AST elevation, γ-GTP elevation, ALP increase, and urine urobilinogen positivity; rarely cholelithiasis, hepatitis, jaundice, liver failure, fulminant hepatitis, and liver necrosis.
   12. Metabolic and nutritional: increased sodium concentration and increased potassium concentration, occasional BUN increase, CPK increase, diabetes, hypercholesterolemia, hyperglycemia, NPN increase, creatinine increase, alkaline phosphatase increase, weight gain, decreased appetite, and LDH increase; rarely hypoglycemia and hyponatremia.
   13. Musculoskeletal: ganglionic pain; occasional arthralgia, arthrosis, skeletal disorder, myalgia, neck tension, fasciitis, tendonitis, myositis, leg spasm, fracture, and tendon tear; rarely back pain and muscle spasm.
   14. Hematological: decreased hematocrit and increased hemoglobin; occasional ecchymosis, epistaxis, thrombocytosis, and anemia; rarely thrombocytopenia, agranulocytosis, aplastic anemia, pancytopenia, and leucopenia.
   15. Administration site: cellulitis, contact dermatitis, injection site reaction, and skin nodules.
   16. Other organs: occasional dysgeusia.
   17. Urinary: occasional albuminuria, cystitis, hematuria, difficulty in micturition, oliguria, renal stones, urinary incontinence, and urinary tract infection.
   18. Eye: occasional blurred vision, cataracts, conjunctivitis, eye pain, and glaucoma; rarely ocular bleeding, and retinal arterial or venous occlusion.
   19. Renal: increased creatinine and β2-microglobulin; occasional NAG increase and urinary occult blood positivity; rarely renal dysfunction, acute renal failure, and interstitial nephritis.
2. Local post-marketing survey results:

In a 6-year post-marketing study in Korea, 338 adverse events in 305 of 5,648 cases (5.40%) were reported, regardless of causative relationship to this drug. Dyspepsia was the most frequent adverse event, with 155 cases (2.74%), followed by facial edema (54 cases, 0.96%), abdominal pain (33 cases, 0.58%), nausea (13 cases, 0.23%), peripheral edema and rash (each 12 cases, 0.21%), diarrhea (10 cases, 0.18%), and insomnia (7 cases, 0.12%). Other reported adverse events included headache and dizziness (each 5 cases, 0.09%); 2 cases (0.04%) of each of constipation, gastric ulcer, urticaria, and anemia; and 1 case (0.02%) of each of thirst, ulcerative stomatitis, gastritis, allergy, back pain, chest pain, hypercholesterolemia, diabetes, weight gain, fracture, arthralgia, RA factor positivity, herpes zoster, pneumonia, dyspnea, ALT elevation, and hearing difficulty.

1. Overseas post-marketing survey results:

Adverse events reported in overseas post-marketing surveys include cholestasis, cholestatic hepatitis, jaundice, renal syndrome, minimal change disease (MCD), acute generalized exanthematous pustulosis (AGEP), and decreased female reproductivity.

## Contraindications for the control drug

1. Patients with hypersensitivity or a history of hypersensitivity to this drug or its components.
2. Patients with an allergic reaction to sulfonamides.
3. Patients with a history of asthma, acute rhinitis, nasal polyps, angioedema, urticaria, or allergic reaction to aspirin or other NSAIDs (including COX-2 inhibitors).
4. Patients with severe hepatic impairment.
5. Patients with severe renal impairment (CrCl <30 mL / min).
6. Patients with active peptic ulcer disease or GI bleeding.
7. Patients with inflammatory bowel disease, such as Crohn’s disease or ulcerative colitis.
8. Patients with congestive heart failure (NYHA II-IV).
9. Patients with confirmed ischemic heart disease, peripheral arterial disease, and / or cerebrovascular disease.
10. Pregnant women, or women of childbearing age.
11. Lactating women.
12. Patients being treated for post-carotid arterial bypass graft (CABG) pain.
13. Patients with hyperkalemia.
14. This drug contains lactose. Therefore, patients with genetic conditions such as galactose intolerance, Lapp lactase deficiency, or glucose-galactose malabsorption should not be given this drug.

## Warnings for the use of the control drug

Warnings for use of the control drug are as follows:

1. If a person who regularly drinks more than 3 standard drinks per day requires this drug or another antipyretic analgesic drug, a physician or a pharmacist must be consulted. Such a person is at an increased risk of gastrointestinal bleeding.
2. Cardiovascular risk: NSAIDs, including this drug, may cause an increased risk of serious adverse cardiovascular thrombotic events, myocardial infarction, and stroke, all of which can be fatal. This risk may be increased with increasing duration of treatment, and may be higher in patients with cardiovascular disease or cardiovascular risk factors. Physicians and patients should remain alert for the development of such events, even in the absence of previous cardiovascular symptoms. Patients should be informed about the signs and / or symptoms of serious cardiovascular toxicity and the steps to take if they occur.
3. Gastrointestinal risk: NSAIDs, including this drug, can cause serious gastrointestinal adverse events including bleeding, ulceration, and perforation of the stomach, small intestine or large intestine, all of which can be fatal. Although this drug significantly reduced the risk of peptic ulcers compared to naproxen alone, adverse events, including ulceration and related complications, still occur. These events can occur at any time, with or without warning symptoms, during treatment with NSAIDs. The risk of serious gastrointestinal adverse events may be higher in the elderly. A longer duration of treatment may increase the risk of serious gastrointestinal adverse events, but a shorter treatment duration does not exempt patients from this risk. Patients must be carefully monitored for signs and symptoms of gastrointestinal ulcers or bleeding. If serious gastrointestinal adverse events are suspected, additional evaluation and treatment must be immediately given. It may be necessary to discontinue NSAID therapy until all serious gastrointestinal adverse events have been ruled out. In high risk groups, an alternative, non-NSAID therapy must be considered.

## Precautions for the use of the control drug

#### Administration with caution

Administer cautiously to:

1. Patients with current or a history of hepatic dysfunction.
2. Patients with current or a history of renal dysfunction (CrCl <60 mL / min).
3. Asthmatic patients.
4. Patients with current or a history of heart failure.
5. Patients with current or a history of hypertension.
6. Patients with current or a history of fluid retention or edema.
7. Patients on diuretics or ACE inhibitors.
8. Patients at risk of hypovolemia.
9. Patients with dehydration.
10. Elderly patients.
11. Patients with a history of peptic ulcers or gastrointestinal bleeding.
12. Patients with risk factors (hypertension, hyperlipidemia, diabetes, or smoking) for cardiovascular events (myocardial infarction or stroke), cardiovascular disease, or a history of cardiovascular disease.
13. Patients with poor metabolism via CYP2C9.
14. Patients with coagulopathy or patients on anticoagulants.
15. Patients planning for pregnancy (this drug may damage female reproductive ability).
16. Diabetic patients.

#### General precautions

1. Carefully consider the potential benefits and risks of this drug and other treatment options before deciding to use it. Since cardiovascular risk increases with increasing dosage and exposure to this drug, use the lowest effective dose for the shortest duration possible. In osteoarthritis patients, in particular, reassess regularly.
2. Gastrointestinal adverse events: NSAIDs should be prescribed with extreme caution in patients with a prior history of ulcerative disease or gastrointestinal bleeding. Patients with a prior history of peptic ulcer disease and / or gastrointestinal bleeding who use NSAIDs have a greater than 10-fold increased risk of developing a GI bleed than patients who have neither of these risk factors. Other factors that increase the risk of GI bleeding in patients treated with NSAIDs include concomitant use of oral corticosteroids or anticoagulants, a longer duration of NSAID therapy, smoking, use of alcohol, older age, and poor general health. Most spontaneous reports of fatal GI events are in elderly or debilitated patients and therefore special care should be taken in treating this population.
3. Hypertension: As with all NSAIDs, this drug can lead to the onset of hypertension or the worsening of pre-existing hypertension, either of which may contribute to the increased incidence of cardiovascular events. Patients taking thiazides or loop diuretics may have an impaired response to these therapies when taking NSAIDs. NSAIDs, including this drug, should be used with caution in patients with hypertension. Blood pressure should be monitored closely during the initiation of therapy with this drug and throughout the course of the therapy.
4. Congestive heart failure and edema: Fluid retention and edema have been observed in some patients taking NSAIDs, including this drug. The inhibition of prostaglandin synthesis may lead to worsening of renal function and fluid retention. Therefore, this drug must be administered with caution in patients with heart failure, left ventricular dysfunction, hypertension, edema, or fluid retention. This drug must also be administered with caution in patients on diuretics or patients at risk of blood volume depletion.
5. Long-term administration of NSAIDs has resulted in renal papillary necrosis and other renal injury. Since prostaglandins are important in maintaining renal blood flow, particular care must be taken in patients with heart failure, renal failure, or hepatic failure, in patients on diuretics, ACE inhibitors, or angiotensin II blockers, and in elderly patients. Discontinuation of this drug usually leads to recovery to the pre-administration state.
6. Advanced renal disease: No information is available from controlled clinical studies regarding the use of this drug in patients with advanced renal disease. Therefore, treatment with this drug is not recommended in patients with advanced renal disease. If this drug must be used, close monitoring of the patient's renal function is advisable.
7. NSAIDs, including this drug, may lead to elevated liver enzymes. These laboratory abnormalities may progress, may remain unchanged, or may be transient with continuing therapy. Rare, and sometimes fatal, cases of severe hepatic reactions, including jaundice and fulminant hepatitis, liver necrosis and hepatic failure have been reported with NSAIDs, including this drug. A patient with symptoms and / or signs suggesting liver dysfunction, or in whom an abnormal liver test has occurred, should be monitored carefully for evidence of the development of a more severe hepatic reaction while on therapy with this drug. If clinical signs and symptoms consistent with liver disease develop, or if systemic manifestations occur (e.g. eosinophilia, rash, etc.), use of this drug should be discontinued.
8. Patients on long-term treatment with this drug should have their hemoglobin or hematocrit checked if they exhibit any signs or symptoms of anemia or blood loss. This drug does not generally affect platelet counts, prothrombin time (PT), or partial thromboplastin time (PTT), and does not inhibit platelet aggregation at the indicated dosages.
9. Patients on long-term NSAID treatment should have a CBC and their chemistry profile checked periodically. If systemic clinical symptoms (e.g. eosinophilia, rash) associated with hepatic or renal pathology, or abnormal liver or renal function tests, persist or worsen, use of this drug must be discontinued.
10. Anaphylactoid reactions: As with NSAIDs in general, anaphylactoid reactions have occurred in patients without known prior exposure to this drug. This complex symptom typically occurs in asthmatic patients who experience rhinitis with or without nasal polyps, or who exhibit severe, potentially fatal bronchospasm after taking aspirin or other NSAIDs. Emergency help should be sought in cases in which an anaphylactoid reaction occurs.
11. Skin reaction: This drug is a sulfonamide and can cause serious skin adverse events such as exfoliative dermatitis, Stevens-Johnson syndrome (SJS), and toxic epidermal necrolysis (TEN), which can be fatal. These serious events can occur without warning and in patients without prior known sulfa allergy. In most cases, such adverse events occur within 1 month of initiation of therapy with this drug. Patients should be informed about the signs and symptoms of serious skin manifestations and use of the drug should be discontinued at the first appearance of bullae, fever, skin rash or any other sign of hypersensitivity.
12. Patients with asthma may have aspirin-sensitive asthma. The use of aspirin in patients with aspirin-sensitive asthma has been associated with severe bronchospasm, which can be fatal. Since cross reactivity, including bronchospasm, between aspirin and other nonsteroidal anti-inflammatory drugs has been reported in such aspirin-sensitive patients, this drug should not be administered to patients with this form of aspirin sensitivity and should be used with caution in patients with preexisting asthma.
13. This drug cannot be expected to substitute for corticosteroids or to treat corticosteroid insufficiency. Abrupt discontinuation of corticosteroids may lead to exacerbation of corticosteroid-responsive illness. Patients on prolonged corticosteroid therapy should have their therapy tapered slowly if a decision is made to discontinue corticosteroids.
14. Based on pharmacological properties of this drug, signs and symptoms of fever or inflammation may be subdued, delaying the diagnosis of infectious complication under conditions of pain or non-infection.
15. If a patient shows signs of severe dehydration, fluid replacement must be given before administration of this drug, and patient must be monitored carefully.
16. This drug can be used on a short-term basis (within 1 week) for alleviation of acute pain (perioperative or post-extraction pain) in adults.
17. Two large, controlled, clinical trials of a different COX-2 selective NSAID for the treatment of pain in the first 10-14 days following CABG surgery found an increased incidence of myocardial infarction and stroke.
18. Clinical tests (urine analysis, blood test, renal function test, liver function test, electrocardiocram, fecal occult blood test, etc.) must be conducted regularly or as needed. If abnormal result is found, appropriate management including temporary or permanent discontinuation of this drug must be implemented.
19. This drug should not be co-administered with NSAIDs other than low-dose aspirin (325 mg/day or less).
20. Patients who experience dizziness or somnolence after taking this drug should avoid driving or operating heavy machinery.
21. This drug does not exert any effect on platelet function and should not be used as a substitute for aspirin in preventing cardiovascular events. Patients receiving antiplatelet therapy should not discontinue it when given this drug.
22. It must be noted that NSAID therapy is a symptomatic treatment rather than curative treatment.
23. Cross-sensitivity: a patient with sensitivity to an NSAID may exhibit sensitivity to other NSAIDs.
24. Patients with autoimmune disease (e.g. systemic lupus erythematosus (SLE) and mixed connective tissue disease (MCTD) patients) may be at an increased risk of asceptic meningitis when given this drug.
25. Administration of this drug to diabetic patients or concomitant administration of this drug with agents that increase serum potassium concentration may cause hyperkalemia. Therefore, in such cases, regular monitoring of potassium level is necessary.
26. Temporary infertility has been reported in female patients on long-term NSAID therapy.

#### Drug Interactions

1. This drug is a CYP2C9 inhibitor. Caution must be taken when coadministering this drug with other drugs metabolised through CYP2C9.
2. Concomitant administration of fluconazole at 200 mg once daily resulted in a two-fold increase in celecoxib plasma concentration. This increase is due to the inhibition of celecoxib metabolism via P450 2C9 by fluconazole. This drug should be introduced at the lowest recommended dose in patients receiving fluconazole. Plasma concentration of this drug and Fluvastatin may increase when the two are used concomitantly. Combination of this drug with CYP2C9 inducers, such as rifampicin, carbamazepine, and barbiturates, may decrease the plasma concentration of this drug.
3. According to an in vitro study, celecoxib is an inhibitor of CYP2D6 and may interact with medicinal products which are metabolised by CYP2D6. When administered concomitantly, this drug may increase the plasma concentrations of CYP2D6 substratied, such as antidepressants (tricyclics and SSRIs), neuroleptics, anti-arrhythmic medicinal products, and dextromethorphan. Dose reduction of such drugs may be necessary when starting concomitant administration with celecoxib. Dose increase of such drugs may be necessary when stopping concomitant administration with celecoxib.
4. In vitro study showed that this drug partially inhibits metabolism mediated by CYP 2C19. Though clinical relevance of this finding is not known, drugs metabolized by CYP2C19 include diazepam, citalopram, and imipramine.
5. ACE inhibitors or angiotensin II receptor blockers: NSAIDs may reduce the effect of anti-hypertensive medicinal products including ACE-inhibitors and angiotensin II receptor antagonists. Therefore, concomitant administration of celecoxib with ACE inhibitor or angiotensin II receptor blocker should be conducted with such interactions in mind. In patients with renal impairment (e.g., dehydrated or elderly patients), concomitant administration of NSAID including this drug with ACE inhibitor or angiotensin II receptor blocker may generally increase the risk of reversible acute renal failure. Therefore, such combination must be given with care, especially in elderly patients. Adequate hydration and regular renal function monitoring must be provided after starting such combination therapy.
6. Diuretics: Clinical studies, as well as post-marketing observations, have shown that NSAIDs can reduce the natriuretic effect of furosemide and thiazides in some patients. This response has been attributed to inhibition of renal prostaglandin synthesis. Patients must be carefully monitored for signs of renal failure while on combination therapy with the above drugs and NSAID.
7. This drug can be used with low-dose aspirin (325 mg/day or less). However, concomitant administration of aspirin with this drug increases the rate of GI adverse events, including ulceration or other complications, compared to use of this drug alone Because of its lack of platelet effects, this drug is not a substitute for aspirin for cardiovascular prophylaxis
8. Lithium: NSAID can increase the plasma concentration of lithium and renal clearance of lithium by inhibiting prostaglandin synthesis in the kidneys. Therefore, patients on NSAID and lithium combination therapy must be monitored carefully for signs of lithium toxicity.
9. Methotrexate: NSAIDs may reduce the tubular excretion of methotrexate leading to increased fatal hematological toxicity of methotrexate. Therefore, this drug should not be co-administered with high-dose methotrexate used for cancer treatment (greater than 15 mg/week), and co-administration of this drug with low-dose methotrexate must be carefully considered. Co-administration of celecoxib with methotrexate (dose for rheumatoid arthritis treatment) in rheumatoid arthritis patient did not exert a significant effect. However, appropriate monitoring must be exercised for methotrexate-associated toxicity during co-administration.
10. Coumarin-type anticoagulant (e.g. warfarin): warfarin and NSAID can have a synergetic effect on gastrointestinal bleeding. Therefore, patients on both drugs concomitantly may be at an increased risk of gastrointestinal bleeding. In a study that evaluates the effect of this drug on anticoagulatory activity of warfarin by administering 2-5 mg of warfarin in health subjects, there was no effect on anticoagulatory activity as evaluated by prothrombin time. However, according to post-marketing survey, significant bleeding associated with increased prothrombin time that can potentially be fatal has been reported in patients who received concomitant therapy with warfarin and this drug, especially in the elderly.
11. Co-administration of NSAID with cyclosporin or tacrolimus may increase renal toxicity of cyclosporin or tacrolimus. Therefore, when co-administering the above drugs, renal function must be monitored.
12. Administration of this drug with high fat diet delayed maximum plasma concentration by 1-2 hours and increased AUC by 10-20%.
13. Co-administration of this drug with antacids containing aluminum or magnesium resulted in 37% decrease in maximum plasma concentration and 10% decrease in total absortion. Patient must be monitoring for signs of renal failure during co-administration.
14. This drug must not be co-administered with NSAIDs other than low-dose aspirin (325 mg/day or less), as this may increase the risk of adverse events.
15. Corticosteroids: there is an increased risk of gastrointestinal adverse events (e.g., ulcer, bleeding), expecially in elderly (65 years old or more).

#### Special Populations

1. Pregnancy and breastfeeding
   1. In rat models, this drug is not known to delay delivery. However, effect of this drug on labor and delivery is not known.
   2. In developmental study on rabbit embryo and fetus, oral administration of 150 mg/kg/day or more (Equivalent to two times the AUC0-24 in humans with 200 mg twice daily administration) was associated with rare increase in fetal deformities such as rib fusion and sternal segment fusion and interventricular septal defect. In one out of two developmental studies on rat embryo and fetus, oral administration of 30 mg/kg/day or more (Equivalent to six times the AUC0-24 in humans with 200 mg twice daily administration) was associated with volume dependent increase in fetal diaphragmatic hernia. Also, administration of 50 mg/kg/day or more in rats (Equivalent to six times the AUC0-24 in humans with 200 mg twice daily administration) has been shown to result in increased pre- and post-implantation loss and embryo-foetal lethality. This toxicity was not observed when pregnancy was initiated 2 weeks after drug discontinuation.
   3. There are no clinical data on ths administration of this drug in pregnant women. Animal studies (rabbit and rat) showed reproductive toxicity including deformities, but no potential risk in human pregnancy is known. As this drug, like other prostaglandin synthesis inhibitors, may cause uterine incompetence or early closure of fetal ductus arteriosus, this drug should not be administered to pregnant women or women capable of becoming pregnant. If pregnancy is confirmed during administration of this drug, this drug must be discontinued.
   4. Mechanism of action of NSAIDs may hinder or delay folicular rupture, which may cause reversible infertility in certain women. Therefore, discontinuation of NSAIDs including this drug must be considered in women who are reproductively challenged or undergoing infertility tests.
   5. Inhibition of prostaglandin synthesis may adversely affect the pregnancy and/or the embryo/foetal development. Data from epidemiological studies suggest an increased risk of miscarriage after use of a prostaglandin synthesis inhibitor in early pregnancy. In animals, administration of a prostaglandin synthesis inhibitor has been shown to result in increased implantation failure.
   6. This drug is excreted in human milk at similar concentration as plasma. According to limited data on 1 study subject, this drug may also be transferred to human breastmilk. Many drugs may be transferred to human breastmilk and possibly cause severe adverse events. Therefore, either breastfeeding or drug administration must be discontinued considering the importance of drug administration in pregnant women.
2. Pediatric population
   1. The safety and efficacy of VIMOVO in children aged 0 to 18 years has not been established.
3. Elderly
   1. Older people are at an increased risk of renal, hepatic, and cardiac impairment. Administration fo this drug to elderly require adequate observation.
   2. According to previous clinical studies, there was no significant difference in efficacy between elderly and younger subjects. Studies that compare renal function (GFR, BUN, creatinine) and platelet function (bleeding time, platelet aggregation) did now show a difference between elderly and younger subjects. However, similarly to other NSAIDs including selective COX-2 inhibitors, spontaneous post-marketing adverse events such as fatal gastrointestinal adverse events and acute renal failure have been reported more frequently in elderly subjects.
   3. Dose adjustment is generally not required in the elderly. However, this drug must be initiated at minimum dosage in patients under 50 kg.

## Packaging and labelling

The investigational product will be supplied by the sponsor and will be boxed. The investigational product must be managed at study sites by the clinical study pharmacist or other qualified personnel. The experimental drug, Naxozol (in tablet form), and the comparator, Celebrex (in capsule form), will both be packaged and supplied in a box.

The following information will be specified on one panel per box in black, according to the Enforcement Rule of Medicinal Product Safety, Article 69 (6):

- - An indication that the investigational product is “for use in the clinical study only”.
  - The designated identification code.
  - The product code or generic name of the main active ingredient.
  - The manufacture code and use-by or re-test date.
  - Storage instructions.
  - The business name and address of the approval authority for the study protocol.
  - An indication that the investigational product “cannot be used for purposes other than the clinical study”.

For primary labeling of the direct product container, only the following information will be specified due to limited space: an indication that the investigational product is only for use in the clinical study, the business name of the authority approving the study protocol, the subject registration number, the product code or generic name of the active ingredient, the re-test date, and the manufacture date.

## Supply, handling, and storage

#### Supply

The experimental drug and the comparator will be supplied by Sung Hwan Moon (Severance Hospital, Department of Orthopedics).

#### Handling

Both the experimental drug and the comparator must be managed at the study sites by the clinical trial pharmacist or other qualified personnel.

#### Storage

The investigational products must be stored in sealed containers.

Use-by date:

- - - Experimental drug: written separately
    - Comparator: written separately

#### Storage and Handling Precautions

1. Store out of reach of children.
2. Storing in an alternative container may cause accidents or compromise the quality of the product. This should therefore be avoided.

## Distribution of the Investigational Product

The investigator is responsible for all aspects of investigational product distribution, including maintenance of the distribution records for the investigational product.

- Upon receipt of the investigational product, the investigator or investigator’s proxy must confirm receipt by signing (including initials) and dating the document provided by the sponsor or sponsor’s proxy. The document must then be returned to the investigational product supplier. A copy should be stored in the investigator’s file.
- If necessary, the recipient of the investigational product must receive and keep a log of the storage temperature during transport.
- The specifics of the investigational product distribution must be accurately recorded in the investigator or sponsor’s study drug receipt / shipment form. At the monitoring visit, the monitoring officer must be able to check the accuracy of such details.

The investigator must also keep the records detailed below:

- A document that confirms the administration of the investigational product to the subjects according to the protocol and its amendments.
- A document that confirms that there is appropriate management of the supplied investigational product and no discrepancy between shipment and receipt.
- If necessary, a document that confirms the appropriate storage of the investigational product (e.g. temperature).

Unused investigational product should not be discarded or used for purposes other than this clinical trial. Unused investigational product supplied to a subject must not be re-supplied to other subjects.

## Treatment compliance

The number of investigational products administered during the study will be recorded, and this number will be used to evaluate treatment compliance.

## Blinding

- Confirmation of the inability to differentiate between the investigational products:

This study applies double-blinding to which of the experimental drug or the comparator is being administered. The investigational products for this study (experimental drug: Naxozol tablet; comparator: Celebrex capsule) differ in color and packaging, so this study is a double-placebo study that uses a placebo for the experimental drug that is equal in form, shape, and packaging to the experimental drug, and a different placebo for the comparator that is equal in form, shape, and packaging to the comparator, ensuring that the double-blinding of the investigator and subjects will be maintained during the study period. Neither the investigator nor the subject can be aware of whether the experimental drug or the comparator was administered to the subject until the termination of the study.

- Preparation and maintenance of the randomization code and unblinding envelope:

The randomization code will be created by the sponsor or a proxy randomization code generator designated by the sponsor. The generated randomization code will be sent to the person in charge of packaging the experimental drug and comparator for Sung Hwan Moon (Department of Orthopedics, Severance Hospital). The unblinding envelope will be provided to the investigator in case emergency unblinding is necessary. The unblinding envelope will be safely sealed with the study arms for the study subjects until the time of unblinding. After the termination of the study, a monitoring agent will confirm that the unblinding envelope remains sealed.

- Collection of the investigational product:

The blind must be maintained until the time of unblinding. For this, the investigational products must be sealed and stored appropriately.

- Unblinding of the double-blind:

Neither the investigator nor the subject should know whether the experimental drug or the comparator was administered. After the termination of the study, the sponsor will open the code to the investigator for data analysis. The randomization code should not be accessed until uncoding, according to appropriate process after the termination of study. However, unblinding for the entire subject group or for individual subjects is possible for the evaluation of safety and efficacy by the investigator.

## Emergency unblinding

The unblinding envelope provided to the investigator should only be opened in emergency situations. If unblinding is needed, the investigator must inform the sponsor before unblinding the subject or subjects. If the randomization is unblinded, the sponsor must be informed immediately over the phone, and the reason and date of the unblinding must be recorded in a separate document. The opened unblinding envelope must be dated and signed by the investigator.

## Management of overdose

The experimental drug has not previously been administered to humans. If overdose is observed, the investigator must admit the subject for monitoring of adverse events and appropriate symptomatic treatment.

- Experimental drug:

1. Overdose of this drug has not been reported during clinical studies. No severe toxicity was seen after administering this drug to 12 patients at ≤2400 mg / day for ≤10 days.
2. Symptoms of NSAID overdose usually include malaise, somnolence, nausea, vomiting, and upper abdominal pain. These symptoms can be treated with supportive therapy. Gastrointestinal bleeding may occur, and rarely also hypertension, acute renal failure, respiratory depression, and coma.
3. Symptomatic and supportive therapy is standard, and there is no specific antidote.
4. Symptom onset within 4 hours of use or after very excessive use may be managed with induced vomiting, activated charcoal (60-100 g for adults, 1-2 g / kg for children), and osmotic laxatives.
5. Hemodialysis, forced urination, alkalization of urine, and hemoperfusion are not useful due to the high protein binding rate of celecoxib.

- Comparator:

1. Overdose of this drug has not been reported during clinical studies. No severe toxicity was seen after administering this drug to 12 patients at ≤2400 mg/day for ≤10 days.
2. Symptoms of NSAID overdose usually include malaise, somnolence, nausea, vomiting, and upper abdominal pain. These symptoms can be treated with supportive therapy. Gastrointestinal bleeding may occur, and rarely also hypertension, acute renal failure, respiratory depression, and coma.
3. Symptomatic and supportive therapy is standard, and there is no specific antidote.
4. Symptom onset within 4 hours of use or after very excessive use may be managed with induced vomiting, activated charcoal (60-100 g for adults, 1-2 g / kg for children), and osmotic laxatives.
5. Hemodialysis, forced urination, alkalization of urine, and hemoperfusion are not useful due to the high protein binding rate of celecoxib.

## Evaluation and treatment of subjects after study termination

Subjects will receive standard therapy according to the institution, the generally accepted therapeutic guideline, and the individual medical need of the subject for any adverse events occurring during the study period.

# Study procedure and evaluation

The study procedure and evaluations will be conducted as detailed below:

## Evaluation schedule

A general summary of the study procedure is shown in the study flow chart (Appendix 1). All visits must be conducted according to the pre-determined schedule. The study duration for each subject is 12-14 weeks and consists of the following evaluations:

- - Screening and washout period: 0 - 2 weeks (Day -14 - Day 1).
  - Treatment & F/U period: 12 weeks (Day 1 - Day 84).

The visit schedule during the study consists of 3 visits, including 1 pre-treatment screening visit. Randomization and study drug distribution will be conducted at visit 2, and the last visit will be conducted 12 weeks after the first day of the investigational product administration.

Additional visits may be conducted if adverse events occur.

#### Visit 1: Screening visit (Day -14 - Day 1)

At visit 1, the investigator will obtain consent from all subjects and evaluate whether they meet all the inclusion criteria and do not meet any of the exclusion criteria. If deemed suitable for this study, the investigator will conduct the following procedures and evaluations for each subject:

- - Obtain signed consent.
  - Assign screening number.
  - Document demographic data.
  - Document item data.
  - Physical examination (including weight measurement).
  - Vital signs (blood pressure, pulse rate, respiration rate).
  - Take blood sample.
  - Pregnancy test (for women of childbearing potential).
  - Confirm medication history.
  - Check pre-treatment adverse event.
  - Instruct for washout if the subject is on contraindicated medication.
  - (If applies) book visit 2 on Day 1 within 14 days of visit 1.
  - Fill out the case report form.

#### Visit 2: Randomization visit (Day 1)

Visit 2 will be conducted on Day 1. The investigator will conduct the following procedures and evaluations for each subject:

- - Reconfirm the fulfillment of inclusion / exclusion criteria.
  - Conduct the pain VAS evaluation.
  - Conduct the LDQ evaluation.
  - Randomization.
  - Record all new adverse events and / or changes in pre-existing adverse events.
  - Record all co-administered drugs and / or changes in pre-existing co-administered drugs.
  - Distribute the GSRS and EQ-5D surveys and ask the subjects to answer them.
  - Distribute the investigational products.
  - Explain how to use the ancillary and rescue drugs, and how to fill out the subject log.
  - Distribute the ancillary and rescue drugs.
  - Confirm subject log completion and remind to bring the log at visit 3.
  - Book visit 3: the visit should be 84 days after visit 2 (Day 84 - 87).
  - Fill out the case report form.

#### Visit 3 (Day 84 - 87)

The following procedures and evaluations will be conducted:

- - Record all new adverse events and / or changes in pre-existing adverse events.
  - Record all co-administered drugs and / or changes in pre-existing co-administered drugs.
  - Conduct the pain VAS evaluation.
  - Conduct the LDQ evaluation.
  - Distribute the GSRS and EQ-5D surveys and ask the subjects to answer them.
  - Physical examination.
  - Vital signs (blood pressure, pulse rate, respiratory rate).
  - Blood sample collection.
  - Collect the unused investigational products, ancillary drugs, and rescue drugs.
  - Collect the completed subject log.
  - Complete the case report form.

#### Early termination visit

During this visit, the following procedures and evaluations will be conducted, as per visit 3:

- - Record the reason for early termination.
  - Record all new adverse events and / or changes in pre-existing adverse events.
  - Record all co-administered drugs and / or changes in pre-existing co-administered drugs.
  - Conduct the pain VAS evaluation.
  - Conduct the LDQ evaluation.
  - Distribute the GSRS and EQ-5D surveys and ask the subjects to answer them.
  - Physical examination.
  - Vital signs (blood pressure, pulse rate, respiratory rate).
  - Blood sample collection.
  - Collect the unused investigational products, ancillary drugs, and rescue drugs.
  - Collect the completed subject log.
  - Complete the case report form.

#### Additional visit

Additional visits may be conducted, if required, for the follow-up evaluation of adverse events, etc. The evaluation criteria for the additional visits will be subject to the investigator’s judgement, but may include the following:

- - Record all new adverse events and / or changes in pre-existing adverse events.
  - Record all co-administered drugs and / or changes in pre-existing co-administered drugs.
  - Physical examination.
  - Vital signs (blood pressure, pulse rate, respiratory rate).
  - Blood sample collection.
  - Complete the case report form.

## Demographic information and other baseline evaluations

Demographic and other baseline information, including the following variables, will be evaluated:

- - Demographic information, such as age and gender.
  - Information regarding health status, such as current and past medical history.
  - Past and present medication history (including co-administration of low dose aspirin).
  - Participation in other clinical trials (within 30 days of screening).
  - Weight.
  - Surveys related to efficacy evaluation (LDQ, GSRS, pain VAS, and EQ-5D).

## Safety evaluation

The safety of the investigational products will be evaluated by recording, reporting, and analyzing the subjects’ baseline comorbidities, adverse events, physical examination results (including vital signs), and laboratory test results. The severity of the adverse events that may occur after the administration of the investigational products will be evaluated using the CTCAE (Common Terminology Criteria for Adverse Events).

After consent has been given, a comprehensive evaluation of drug toxicity, including the adverse events experienced by the subjects, will be conducted throughout the duration of the study. All personnel involved in the study must report all adverse events reported by the subjects and recognized by the investigator (refer to section 6.3.1.2, “Reporting and evaluation of adverse events”). Refer to Sections 6.3.1.4 and 6.3.1.5 for the deadline for adverse events reporting.

## Adverse events

## Definition of adverse events

#### Adverse events

Adverse events include all harmful and unintended signs (including laboratory test results), symptoms, or diseases occurring in subjects who have received the investigational products. The adverse events need not be causally related to the investigational products. In cases of surgical or diagnostic treatment, the causative condition or disease, rather than the treatment itself, will be considered as an adverse event. In cases of death, the cause of the death will be considered an adverse event, and the death will be considered an outcome of the said adverse event.

The following items are not included as adverse events:

- - The disease or disorder being investigated by this study.
  - An asymptomatic overdose of the investigational products or co-administered drugs.
  - Medical or surgical treatment (the causative condition for the treatment is considered an adverse event).
  - A disease or condition existing before, or found at the start of, the study that does not worsen during the study period.
  - Cases in which no undesirable medical events occurred (e.g. hospitalization for pre-scheduled surgery for a pre-existing condition that did not worsen after administration of the investigational product).

#### Causal relationship between the drug and adverse events

Evaluation of an association between the administration of the investigational product and adverse events (definitely related, probably related, possibly related, unlikely to be related, not related, not assessable) will be performed clinically, based on all information obtainable at the time of the case report completion.

#### Definitely related

An association between the investigational product and an adverse event is clear and fulfills all of the criteria for ‘Probably related’ in addition to the criteria below:

- - Recurrence at resumption of administration (if applicable).
  - The adverse event is consistent with known information regarding the investigational product or drugs of the same class as the investigational product.

#### Probably related

This category includes clinical events such as laboratory abnormalities that are not associated with comorbid diseases, other drugs, and chemicals that all have logical temporal continuity with investigational product administration, lead to clinically relevant results after investigational product discontinuation, and fulfill the following criteria:

- - Evidence of investigational product administration.
  - Logical temporal order between investigational product administration and the adverse event incidence.
  - The adverse event can be explained more logically by exposure to the investigational product than by other possible reasons.
  - A disappearance or alleviation of the adverse event after investigational product discontinuation or dose reduction.

#### Possibly related

Clinical events such as laboratory test abnormalities that can be explained by co-morbid diseases, other drugs, or chemicals, but which also show logical temporal continuity with investigational product administration (although not accompanied by information on drug discontinuation) and fulfill the following criteria:

- - Evidence of investigational product administration.
  - Logical temporal order between investigational product administration and the adverse event incidence.
  - An adverse event that is equally likely to be due to the investigational product as to other possible causes.
  - An adverse event that disappears or is alleviated by investigational product discontinuation or dose reduction.

#### Unlikely to be related

Generally, the cases listed below are considered unlikely to be related:

- - Evidence of investigational product administration.
  - A higher possibility that an adverse event is due to reasons other than investigational product administration.
  - Negative or dubious results after investigational product discontinuation or dose reduction (if applicable).
  - Negative or dubious results after investigational product re-administration.

1. **Not related**
   - The subject failed to receive the investigational product.
   - No logical temporal order between investigational product administration and the adverse event incidence.
   - The presence of other evidence that can clearly explain the reason for the adverse event incidence (e.g. bleeding from a surgical site).
   - Not likely (e.g. the patient collided with a motorcycle, but there is not enough proof that the investigational product causes enough loss of proprioception to cause the event, or a cancer that is found days after investigational product administration).

#### Not assessable

There is insufficient or paradoxical information that cannot be supplemented or confirmed.

The following points must be considered in order to evaluate the association between the investigational drug and any adverse events:

- - Temporal continuity after drug administration: An adverse event must occur after investigational product administration. The time interval after investigational product administration to the onset of the adverse event must be evaluated based on the clinical condition of the event.
  - Recovery after drug discontinuation and / or recurrence after re-administration: the reaction of the subject after drug discontinuation and drug re-administration must be evaluated (re-administration must be considered from the point of view of normal clinical steps for the event in question).
  - Baseline, co-existing, and co-morbid disease: reports on each adverse event must be evaluated based on the characteristics and progression of the disease being treated and other diseases.
  - A co-administered drug or treatment: other drugs or treatments administered to the subject may be associated with the event in question and must also be evaluated.
  - A reaction or trend already known to be related to this class of drug: clinical / pre-clinical.
  - Exposure to physical and / or psychological stress: exposure to stress can lead to a change in adverse events and provides a logical and more accurate reason for an adverse event.
  - The pharmacological and pharmacokinetic properties of the drug: the pharmacokinetics of the investigational product (absorption, distribution, metabolism, excretion) must be considered in conjunction with a subject’s medication history.

#### Laboratory abnormalities and other findings outside of the reference range

All laboratory tests and results must be recorded on the case report form. The cases listed below must be treated as adverse events and recorded and reported separately on the adverse event report form within the case report form.

- - Laboratory values that lead to study discontinuation.
  - Clinical symptoms.
  - Subject requires treatment.
  - Other cases deemed clinically relevant by the investigator: clinically significant laboratory abnormalities or other measured values that are found after investigational product administration or that have existed at study commencement, but have been exacerbated after study commencement, are considered to be adverse events or serious adverse events. However, clinically important findings which existed at study commencement, but which were not exacerbated after investigational product administration are not considered adverse events or serious adverse events.

#### Serious adverse events

Serious adverse events (SAEs) refer to incidences of medical events that were not intentional at an arbitrary dose of the investigational product, and include the following cases:

- - Death.
  - A life-threatening situation (a “life-threatening situation” is defined as a case in which a subject is at risk of death during an adverse event; it is not a situation which can theoretically lead to death if the adverse event worsens).
  - A need for hospitalization or prolongation of hospitalization: this does not include hospitalization for treatment related to the protocol or for normal clinical treatment. It refers to official hospitalization and excludes emergency room visits (however, a stay exceeding 24 hours is considered hospitalization). It does not include hospitalization pre-scheduled before study commencement, hospitalization for a pre-existing condition that does not worsen during the study period, or hospitalization for aesthetic purposes (e.g. cosmetic surgery). Hospitalization or prolongation of hospitalization at the subject’s will, and not due to medical reasons, is not considered an SAE.
  - Permanent or serious disability or dysfunction.
  - Fetal deformity or abnormality.
  - Other medically important adverse events: a medically important event can qualify as an SAE even if it does not cause death, is not life-threatening, or does not require hospitalization. Such cases include those that may endanger the subject, according to appropriate medical evaluation, or that require medical or surgical treatment to prevent the above SAE. Examples include allergic bronchospasm that requires at-home or emergency intensive treatment, blood dyscrasia that does not merit hospitalization, convulsions, and drug dependency or misuse. Suspected infection via the investigational product will also be treated as an SAE. All such cases must be reported swiftly, as detailed in Section 6.3.1.4.

#### Reporting and evaluation of adverse events

At each visit, the subject will be asked about their health status. During the adverse event reporting period of the study, all undesirable status changes reported by the subject or observed by the investigator will be recorded as adverse events. All information regarding all adverse events experienced by a subject during the adverse event reporting period (stated below) will be recorded consistently, completely, accurately, and continuously in case report form. SAEs will be recorded additionally in the SAE report form, as per Section 6.3.1.1.4, and reported separately. For each adverse event, the description, duration (including date of occurrence and date of resolution: the timing must also be known if the timing of the adverse event incidence relative to the investigational product administration time is also important), seriousness, association with the study treatment, other suspected causes, treatment given, other treatments (including investigational product dose adjustment and discontinuation), and progress will be reported. SAEs must be strictly identified during the study period and recorded and reported according to the appropriate category.

#### Definition of the adverse event reporting period

The adverse event and SAE reporting period commences on the date of subject enrollment (the date that the consent form is signed) and continues until the end of the follow-up period after treatment. If a continuing adverse event is observed at the visit at the end of the study, the subject will be monitored until the adverse event improves or stabilizes.

#### Reporting process for SAEs

The investigator will inform the sponsor (or proxy) and the pharmacovigilance personnel for the investigational product distributor (Sung Hwan Moon, Department of Orthopedics, Severance Hospital) immediately (within 24 hours of becoming aware of an adverse event), by fax or email, of all new SAEs that occur during this period.

The pharmacovigilance personnel for Sung Hwan Moon (Department of Orthopedics, Severance Hospital) can be contacted as follows:

**Email:** [**drugsafety@hanmi.co.kr**](mailto:drugsafety@hanmi.co.kr)

#### Tel: 02-410-0446

**Fax: 050-2260-0479**

An SAE that has already been reported must also be followed up using the same process and deadline as for a new adverse event.

All SAE reports will be filled out by the investigator, in accordance with the SAE report form, and processed.

The information on an SAE report form must always concur with the appropriate section of the case report form. If the investigator / reporter is asked for follow-up information (i.e. additional information, progress, final evaluation, and / or the relevant record by request) or receives the sponsor’s query regarding the SAE, it must be handled within the same deadline as the first report. This is crucial to allow the sponsor to evaluate the SAE and meet the legal processing deadline for prompt safety reporting.

Under certain exceptional circumstances the sponsor may contact the investigator directly to clarify or discuss a particularly urgent situation, but follow-up observation is normally requested by the monitoring agent in charge of the study.

#### Safety reporting to government authorities, the institutional review board (IRB), and the investigator

If the investigator reports an SAE (in particular, death) in a subject to the IRB, the report must follow the SAE reporting criteria of the said IRB. According to the ICH GCP, the sponsor must notify the investigator if “a case that may negatively affect a subject’s ability to undergo the study or changes the IRB approval status on the continuation of the study” occurs. In particular, according to the relevant regulations, the sponsor must inform the investigator of any suspected unexpected serious adverse reaction (SUSAR). The investigator must store safety-related reports within the investigator’s site file. Safety reporting must comply with local regulations for safety reporting to the investigator. If mandated by law, the sponsor can directly report to the IRB, and such a report will be documented. If not mandated by law, the investigator must directly report the safety information supplied by the sponsor to the IRB, and copies of all communication records regarding this issue must be kept within the investigator’s site file. The sponsor must report safety-related issues to the health authorities, according to the relevant law and regulations. For local studies, SUSAR reports must be conducted according to the Pharmaceutical Affairs Act. According to this Act, any SUSAR that is fatal or life-threatening must be reported to the MFDS within 7 days of the sponsor becoming aware of it, and, in such a case, the sponsor must report the details of the SUSAR within 8 days of the first report date. All other SUSARs must be reported within 15 days of the sponsor being made aware of them.

#### Monitoring of a subject’s adverse event

The investigator must monitor and follow up a subject who has an adverse event that may be associated with the investigational product during the study period until disappearance of the said adverse event or confirmation of final progress, unless follow-up becomes impossible. The investigator must do due diligence to confirm the information regarding the adverse event and must document such information. The investigator is responsible for appropriate additional treatment and follow-up observation of the adverse event. The sponsor must actively follow up adverse events occurring during the study period and collect all relevant information. However, for SAEs, such measures must continue until the resolution of the SAE or confirmation of final progress, while for all other adverse events, such measures will only continue until database lock.

## Exposure of pregnant women

A pregnancy that is deemed by the investigator to be associated with the study treatment (as a result of drug interaction with contraceptives) will be considered an adverse event. However, all pregnancies that occur during the period defined in 6.4.1.3 must be recorded in the adverse event section of the case report form. This applies to both the female subjects and the female partners of male subjects. The investigator must promptly inform the sponsor within 24 hours of becoming aware of the pregnancy using the attached pregnancy report form, in accordance with the process stated in Section 6.4.1.4.

The investigator must actively follow up and record the progress of the pregnancy, even if the subject is removed from the study, and must report the results to the sponsor. If the progress is abnormal and the abnormality persists within the subject or child / fetus, this must be reported using the SAE report form. All abnormal progress related to the pregnancy must be promptly reported by the methods defined in Section 6.3.1.4, and normal progression of the pregnancy must be reported as soon as possible, within 45 days of delivery.

Subjects who become pregnant during the study period must discontinue the investigational product. The sponsor must be notified without delay, and the subject must follow the pre-specified process.

## Laboratory tests

Before the investigational products are delivered to each site, the reference ranges for laboratory values must be conveyed to the sponsor or the sponsor’s proxy. If the reference range changes during the study period, the sponsor or sponsor’s proxy should be notified. The investigator must prevent errors by confirming the identity of the samples collected for laboratory tests.

#### Table 2. List of Laboratory Safety Tests

| **Hematology** | **Serum Chemistry** |
| --- | --- |
| Hemoglobin (Hgb) | Alanine aminotransferase (ALT) |
| Hematocrit (Hct) | Aspartate aminotransferase (AST) |
| RBC count | Blood urea nitrogen (BUN) |
| Platelet | Creatinine |
| WBC with differential count | Glucose |
|  | Total bilirubin |

## Vital signs, physical examination, and other evaluations

A general physical examination will be conducted during the screening and final visits. Vital signs include blood pressure, pulse rate, respiratory rate, and body temperature. The physical examination and vital signs results will be recorded in the case report forms.

## Efficacy evaluation

## Primary evaluation

This study will use the Leeds Dyspepsia Questionnaire (LDQ) as its primary evaluation method,.

The LDQ is a method proven to evaluate the severity and treatment response of eight symptoms of gastrointestinal disorders. (25)

The 8 symptoms evaluated are:

1. Indigestion (pain in the upper abdomen).
2. Heartburn (a burning feeling behind the breast bone)
3. Food or drink stuck behind the breast bone
4. Regurgitation (an acid taste coming up into the mouth from the stomach)
5. Burping or belching
6. Nausea
7. Vomiting
8. Excessive fullness

Each of these symptoms is rated on the Likert scale from very mild, to mild, moderate, severe, and very severe.

## Secondary evaluation

As secondary evaluation endpoints, the GSRS, pain VAS, and EQ-5D will be used.

- The GSRS (Gastrointestinal Symptom Rating Scale) consists of 15 short answer questions that can indicate a high correlation with gastroesophageal reflux disease, functional dyspepsia, and irritable bowel disease. (26, 27, 28)
- The VAS (visual analogue scale) will be used to evaluate the osteoarthritic pain experienced by the subjects. The VAS is a simple method that is widely used for pain evaluation in osteoarthritis patients. It has proven correlation with the survey-based Likert scale. (29)
- EQ-5D is a widely-used tool for the measurement of quality of life and health status. It includes the five categories of mobility, self-care, usual activities, pain / discomfort, and anxiety / depression. Each item is measured using the VAS, indicating subjective health status on a thermometer-shaped scale, as well as by multiple choice questions that evaluate health status as one of the following three stages: “no problem”, “some problems”, and “extreme problems”.

The study will also evaluate the incidence rate of gastrointestinal adverse events and the discontinuation rate due to gastrointestinal adverse events.

As an ancillary method to evaluate the pain control of the experimental drug and comparator, this study will also evaluate ancillary and rescue drug use after 8 weeks of oral administration of the study drugs, the average number of days of use, the average amount used per day (total amount used / days used) and the average amount of use during the study period (total amount used / study duration).

This study will also include compliance analysis. Compliance is defined as ‘(the actual number of administrations) / (the planned number of administrations)*100’, and patients with a compliance rate of 80% or above will be included in the PP (per-protocol) group analysis.

## 6.5. Other evaluations

Pharmacological genomic studies, etc. are not included.

# Statistical analysis plan

## Determination of subject number

**Target number of subjects**: 106

The target number of subjects for each of the experimental and comparator groups of this study is 42. Considering a drop-out rate of 20%, this study will enroll 53 subjects per treatment arm (106 in total).

#### Rationale:

The purpose of this study is to show the non-inferiority of the gastroprotective effect of Naxozol in osteoarthritis patients compared to that of celecoxib, as evaluated by the average change in the LDQ (Leeds Dyspepsia Questionnaire) score from baseline after 12 weeks of oral administration.

In previous studies that evaluated the symptomatic improvement of gastrointestinal disorders with a Likert scale such as the LDQ, 0.40 was used as the minimal clinically important difference (MCID) in the degree of improvement.(30, 31, 32) Hence, the non-inferiority margin for this study was also set at 0.4. The LDQ consists of a 5-step Likert scale.

In a study by Holtmann et al. on the efficacy of Itopride in the improvement of functional dyspepsia, the standard deviation of the average change in the LDQ was 0.63 - 0.65. In this study, the standard deviation (σ) was set at 0.65 for a more conservative approach. (22)

With a power of 80% and a one-sided α of 0.025, the number of subjects per treatment arm, as estimated by the sample size derivation formula below, was 42. Considering a drop-out rate of 20%, this study will enroll 53 subjects per treatment arm (106 in total).

𝑛experimental

2

= 𝑛control =

[2𝜎2][𝑍_𝛼_

δ2

### + 𝑍_𝛽_]

[2 ∗ 0.652][1.96 + 0.842]2

= 0.42 ≅ 42

σ= standard deviation of change in LDQ

δ = non-inferiority margin

## Randomization

#### Screening number

All subjects will receive their own screening number regardless of randomization at the screening visit.

#### Randomization code generation

The randomization code will be generated for the subjects by the sponsor or sponsor’s proxy to evaluate the safety and efficacy of the investigational product. Randomization will be conducted before study commencement. The Permuted Block Randomization Method will be applied for each study site.

Allocation to each treatment arm is double-blinded, hence the investigator and subject cannot know which arm the subject is allocated to. The allocation proportion between the experimental drug and the comparator is 1:1.

#### Subject allocation and randomization code maintenance

Subjects who fulfill the inclusion / exclusion criteria will be assigned randomization numbers in the order of study enrollment. Each subject will be assigned to a treatment arm according to the randomization number and will receive the appropriate investigational product.

The investigator and the subject cannot know whether the subject received the experimental drug or the comparator before study termination. After study termination, the sponsor will open the code to the investigator for data analysis.

## Evaluation endpoints

## Primary evaluation endpoint

The primary evaluation endpoint is the average change in the LDQ from baseline after 12 weeks of oral administration of the investigational product.

Before investigational product administration, the total LDQ score will be categorized into three groups (No / Very Mild: 0-8; Mild / Moderate: 9-24; Severe / Very Severe: 25-40), and the average change in the LDQ from baseline will be calculated for each group.

## Secondary evaluation endpoint

- Average LDQ after 12 weeks of oral administration of the investigational product.
- Average change in the GSRS from baseline after 12 weeks of oral administration of the investigational product.
- Incidence rate of GI adverse events (dyspepsia, diarrhea, nausea, abdominal pain, heartburn) after 12 weeks of oral administration of the investigational product.
- Discontinuation rate due to GI adverse events after 12 weeks of oral administration of the investigational product.
- Average change in the pain VAS from baseline after 12 weeks of oral administration of the investigational product.
- Average change in the average EQ-5D score from baseline after 12 weeks of oral administration of the investigational product.
- Drug compliance after 12 weeks of oral administration of the investigational product.
- Ancillary and rescue drug use after 12 weeks of oral administration of the investigational product: average number of days used, average daily amount used (total amount used / total number of days used), and average amount of use during study participation (total amount used / study duration).
- Incidence, frequency, and characteristics (clinical presentation, severity, result, etc.) of adverse events after 12 weeks of oral administration of the investigational product.
- Results of physical examination, vital signs, and laboratory tests after 12 weeks of oral administration of the investigational product.

## Other evaluation endpoints

N/A

## Definition of analysis sets

#### Target population

This study will be conducted on osteoarthritis patients of ≥50 years who can perform normal physical activity during the study period, with a pain VAS of 40 mm or more.

**Safety evaluation set: A population in which at least one safety follow-up observation is made after randomization and investigational product administration.**

**Full Analysis (FA) set: A population in which at least one efficacy evaluation is performed after randomization and investigational product administration.**

**Per-Protocol (PP) set: A population in which at least one efficacy evaluation is performed after randomization and investigational product administration, and in which there is no major protocol violation (violation of inclusion / exclusion criteria, violation of compliance). A compliance rate of 80% is used as the reference.**

## Statistical analysis plan

## General considerations

Efficacy data obtained from the subjects in this study will be analyzed in the FA and PP sets. Safety data will be analyzed in the Safety evaluation set. Considering that this is a non-inferiority study, the primary efficacy endpoint will be analyzed in both the FA and PP sets, according to ICH guidelines.

All statistical sets, excluding the primary efficacy endpoint, will be subjected to two-sided tests with a significance level of 5%.

Missing data will be replaced using a multiple imputation method. No interim analysis will be performed.

Co-administered drugs will be recorded in ATC terminology, and adverse events and medical history will be standardized to SOC (system organ class) and PT (preferred term) using a Medical Dictionary (MedDRA or WHOART).

## Primary outcome analysis

Average change in LDQ from baseline after 12 weeks of oral administration of the investigational product: descriptive statistics (sample size, mean, standard deviation, median, min, max) will be presented for LDQ change after 12 weeks of investigational product administration in each treatment arm. To prove non-inferiority of the experimental drug, the one-sided 97.5% confidence interval for the between-group difference in LDQ change will be calculated. If the 97.5% upper confidence limit is smaller than the non-inferiority margin of 0.40, the null hypothesis is rejected, and non-inferiority is proven. Additionally, before investigational product administration, the total LDQ score will be categorized into three groups (No / Very Mild: 0-8; Mild / Moderate: 9-24; Severe / Very Severe: 25-40) for further analysis.

## Secondary outcome analysis

- The average LDQ after 12 weeks of oral administration of the investigational product: descriptive statistics (sample size, mean, standard deviation, median, min, max) will be presented for the LDQ score after 12 weeks of investigational product administration. The between-group difference will be tested for significance using the independent two-sample t-test or the Wilcoxon rank sum test.
- The average change in the GSRS from baseline after 12 weeks of oral administration of the investigational product: descriptive statistics (sample size, mean, standard deviation, median, min, max) will be presented for GSRS measured before and after 12 weeks of investigational product administration, and the change in GSRS from baseline after 12 weeks of oral administration of the investigational product in each treatment arm. The change within each arm will be tested for significance using the paired t-test or the Wilcoxon signed rank test. The between-group difference at each time point and in the amount of change will be tested for significance using the independent two-sample t-test or the Wilcoxon rank sum test.
- The incidence rate of GI adverse events (dyspepsia, diarrhea, nausea, abdominal pain, heartburn) after 12 weeks of oral administration of the investigational product: the number and proportion of the subjects who experience a GI adverse event during the 12 weeks of investigational product administration, and the frequency of these events, will be presented for each treatment arm. The between-group difference in proportion will be tested for significance using the Chi-square test or Fisher’s exact test.
- The discontinuation rate due to GI adverse events after 12 weeks of oral administration of the investigational product: the number and proportion of subjects who discontinue drug administration due to GI adverse events that occur during the 12 weeks of investigational product administration will be presented for each treatment arm. The between-group difference in proportion will be tested for significance using the Chi-square test or Fisher’s exact test.
- The average change in pain VAS from baseline after 12 weeks of oral administration of the investigational product: descriptive statistics (sample size, mean, standard deviation, median, min, max) for pain VAS before and after 12 weeks of investigational product administration, and the change in pain VAS from baseline after 12 weeks of oral administration of investigational product in each treatment arm, will be presented. The change within each arm will be tested for significance using the paired t-test or the Wilcoxon signed rank test. The between-group difference at each time point and in the amount of change will be tested for significance using the independent two-sample t-test or the Wilcoxon rank sum test.
- The average change in the average EQ-5D score from baseline after 12 weeks of oral administration of the investigational product: descriptive statistics (sample size, mean, standard deviation, median, min, max) for EQ-5D before and after 12 weeks of investigational product administration and the change in EQ-5D from baseline in each treatment arm will be presented. The change within each arm will be tested for significance using the paired t-test or the Wilcoxon signed rank test. The between-group difference at each time point and in the amount of change will be tested for significance using the independent two-sample t-test or the Wilcoxon rank sum test.
- Drug compliance after 12 weeks of oral administration of the investigational product: compliance during the 12 weeks of investigational product administration is defined by ‘actual number of administrations / predicted number of administrations *100’, and descriptive statistics (sample size, mean, standard deviation, median, min, max) will be presented for each treatment arm. The between-group difference in compliance will be tested for significance using the independent two-sample t-test or the Wilcoxon rank sum test. The actual number of administrations is determined based on the number of returned investigational products. Evident reasons, such as loss or damage, will be accounted for.
- Ancillary and rescue drug use after 12 weeks of oral administration of the investigational product, the average number of days used, the average daily amount used (total amount used / total number of days used), and the average amount of use during study participation (total amount used / study duration): the number and proportion of subjects who use ancillary or rescue drugs during the 12 weeks of investigational product administration are presented for each treatment arm. The between-group difference will be tested for significance using the Chi-square test or Fisher’s exact test. Descriptive statistics (sample size, mean, standard deviation, median, min, max) will be presented for the average number of days of ancillary or rescue drug use, the average amount used per day (total amount used / total number of days used), and the average amount used during the study period (total amount used / duration of study participation) in each treatment arm. The between-group difference in the average will be tested for significance using the independent two-sample t-test or the Wilcoxon rank sum test.
- The incidence, frequency, and characteristics (clinical presentation, severity, result, etc.) of adverse events after 12 weeks of oral administration of the investigational product: the number and proportion of subjects that experienced all adverse events, SAEs, and adverse events leading to study discontinuation during the 12 weeks of investigational product administration, as well as all adverse events, SAEs, and adverse events leading to study discontinuation that are related to the investigational product are presented for each treatment arm. The between-group difference in proportion will be tested for significance using the Chi-square test or Fisher’s exact test. The number of all adverse events and adverse events associated with investigational product, the severity and seriousness of these events, the treatment, and the results of the events will also be presented. The number and proportion of subjects, as well as the number of events for all adverse events and adverse events associated with the investigational product, as per MedDRA or WHOART SOC, and the PT, will also be presented for each treatment arm.
- Physical examination: a distribution chart that includes the frequency and proportion of normal and abnormal physical examination findings before and after the 12 weeks of investigational product administration will be presented. The significance of the change in each treatment arm will be tested using McNemar’s test or McNemar’s exact test, and the between-group difference in change will be tested for significance using the GEE (Generalized Estimating Equation).
- Vital signs: descriptive statistics (sample size, mean, standard deviation, median min, max) will be presented for vital signs before and after the 12 weeks of investigational product administration and for the change in vital signs after the 12 weeks of investigational product administration. The change in each treatment arm will be tested for significance using the paired t-test or the Wilcoxon signed rank test. The between-group difference at each time point and in the amount of change will be tested for significance using the independent two-sample t-test or the Wilcoxon rank sum test.
- Laboratory tests: a distribution chart that includes the frequency and proportion of normal and laboratory test findings before and after the 12 weeks of investigational product administration will be presented. The significance of the changes in each treatment arm will be tested using McNemar’s test or McNemar’s exact test, and the between-group difference in change will be tested for significance using the GEE (Generalized Estimating Equation). For continuous data, such as hematological or blood chemistry test results, descriptive statistics (sample size, mean, standard deviation, median, min, max) for values before and after the 12 weeks of investigational product administration, and the change in values after the 12 weeks of investigational product administration, will be presented. The change in each treatment arm will be tested for significance using the paired t-test or the Wilcoxon signed rank test. The between-group difference at each time point and in the amount of change will be tested for significance using the independent two-sample t-test or the Wilcoxon rank sum test.

## Demographic information

Evaluation of the demographic and other baseline data, including clinical medical history, will be performed on the safety evaluation set. Sample size, mean, standard deviation, median, minimum and maximum values for continuous data, and frequency and proportion for categorical data, will be presented. For comparison between the treatment arms, the independent two-sample t-test or the Wilcoxon rank sum test for continuous data, and the Chi-square test or Fisher’s exact test for categorical data, will be used to evaluate statistical significance.

## 7.6. Interim analysis

Interim analysis will not be performed.

# Ethico-legal aspect of the trial

## Responsibilities of the investigator

The investigator will be responsible for conducting this study at the study site with which they are affiliated. The investigator will ensure that the study is conducted according to the protocol, the ethical principles as per the Declaration of Helsinki, the GCP regulations of ICH (ICH Topic E6, 1996), and the local regulations. The investigator must particularly make sure that only the subjects who provide informed consent are included in the study.

## Subject information and consent

Written consent for study participation is an unconditional and absolute prerequisite for a subject’s participation in the study. Before a subject is accepted onto the study, written consent by the subject or the subject’s parents or legal guardian is required. Before consent is obtained, the investigator must provide the appropriate information to the subject or subject’s legal guardian. The subject information sheet, used for obtaining a subject’s consent, must be in the subject’s mother tongue and prepared by the sponsor in accordance with ICH GCP (ICH Topic E6, 1996). The investigator must also orally explain all aspects of the study, apart from the information given in the written subject information sheet, in layman’s terms (i.e. in language that is easy for non-medical personnel to understand).

After the explanation, the investigator and subject must individually sign and date the subject consent form.

The signed and dated consent forms must be kept at the investigator’s study site and stored safely for easy access for monitoring, check-up, and audits. A copy of the signed and dated consent form, along with a copy of the subject information sheet, must be provided to the subject before study participation.

If important new information arises that can affect the subject’s consent, the sponsor must revise the subject consent form and the subject information sheet and resubmit both documents to the IRB for review and approval. The revised information, after approval, must be provided to each subject, and each subject must sign and date the revised consent form after the investigator has explained the changes from the previous version.

## Subject identification and confidentiality

Once consent is obtained, each subject will be assigned a unique identification number. This number will be used for the duration of the study, as well as during data processing.

Subject information collected during the study period will also be stored using this number. Only the investigator may associate the subject with the subject information regarding this study, using the identification list stored at the study site. Subject confidentiality must be strictly maintained during any review of the original data for document verification by a monitoring agent, during a check-up, or during an audit by health authorities.

The law on information protection and privacy protection covers all stages of obtaining, relaying, processing, and storing a subject’s personal information. The subject must be clearly informed of, and give consent to, information processing methods as per the local legislation. Sensitive information gathered in this study, including information regarding the health of the subjects, will be stored for up to three years after study termination, and thereafter will be destroyed appropriately.

## Subject compensation and insurance

All subjects participating in this study will be protected by insurance appropriate for the local situation. The compensation policy, in case of damage due to this study, will be approved by the IRB.

## Institutional review board (IRB)

Before commencement of the study at each site, the protocol, the case report form, and all documents provided to the subjects, including the subject information sheet, must be submitted to the IRB for approval.

The approval letter from the IRB must be kept in the investigator site file for the relevant study site, and a copy must be kept in the clinical trial basic document file of the sponsor or proxy organization. The study must not begin before IRB approval. Once approval is obtained, a document stating the date of approval, the review board member list, and the voter list must be conveyed. The approval document must clearly state the study, the protocol version, the subject information sheet, and the consent version. If possible, meeting minutes must also be conveyed. Changes to the study must also be submitted to the IRB before implementation (refer to Section 9.5). The relevant safety information during the study period must be submitted to the IRB, in accordance with the regulations and policies.

## Government authorities

In accordance with local law, the relevant regulatory authorities must be notified of the study, and this protocol and other relevant documents (product information for the investigational products, the subject information sheet, and the subject consent form) must be submitted.

# Clinical Trial Management

## Management of case report forms

The purpose of case report forms is to collect necessary information, in accordance with the study protocol, in a manner that is complete, accurate, easily recognized, and time-saving. Information on case report forms must correspond with information on evidence documents.

The information collected during this study must be recorded on case report forms, and, if applicable, adverse event safety report forms, and conveyed to either the sponsor or the sponsor’s proxy. The conveyed information will be processed, evaluated, and stored anonymously in accordance with information protection regulations.

The investigator must confirm that the case report forms and other related documents have been relayed to the sponsor or relevant proxy anonymously.

Data must be recorded in full in the case report forms in an easily legible manner in black or blue ballpoint pen that can be used for official documentation. All changes or amendments must be made and countersigned by the investigator, with the date of the change / amendment recorded. Errors must be documented legibly and must not be corrected using correction tapes. The investigator must record the reason for any change / amendment in important documentation item.

Blank spaces for information omitted from the case report form must be struck out to prevent unnecessary queries.

As these documents are essential to clinical trials, case report forms must be managed so that they are adequate for submission for auditing purposes by government authorities.

## Evidence and subject files

The investigator must preserve all paper or electronic files related to the subjects participating in this study (e.g. medical files and original copies of medical records). These files include demographic data and medical records of the subjects and must be recorded as completely as possible. In particular, the following information must be discernable from these files:

- - Name of subject.
  - Birth date.
  - Gender.
  - Demographic information.
  - Past and present medical history.
  - Past and present medication history (including changes during study period).
  - Trial identification information.
  - Date of study enrollment (date of written consent).
  - Subject number during study period.
  - Dates of site visits.
  - Medical tests, as pre-defined by the protocol, or other clinical findings.
  - All adverse events.
  - Study end date.
  - If applicable, the date of discontinuation of the study or the investigational products, and the reason for discontinuation.

This subject file will be used for the personal identification of each subject.

All documents, including printed results of values measured by an automated device, radiological data, electrocardiogram records, and laboratory test results, will be kept within the file. These documents should at least contain the subject number and information on the date of treatment. If possible, data must be directly printed from the device used for the evaluation or measurement. Data that cannot be printed from an automated device will be recorded in person. If necessary, any medical evaluation based on these records must also be recorded, as well as the investigator’s signature and the date.

Some information can be directly recorded in the case report forms (or survey or logs), rather than in the original medical files, if the original file (paper or electronic) that corresponds to that information does not exist. In this case, the information in the case report forms serves as evidence. If the information in the case report form serves as evidence, the protocol must clearly and completely state what type of information belongs to such a case. If the information in the case report form is not explicitly stated as evidence, it must be stated in the subject’s medical file.

## Storage of the investigator file and data

The investigator will be provided with the investigator site file at the time of study commencement. This file will include all documents necessary to conduct this study and must be regularly updated to maintain a complete record until study termination. This file must be accessible to the monitoring agent for review, and readily available for the sponsor’s review and audit by the health authorities. In accordance with the Enforcement Rule for Medicinal Product Safety Article 30 Clause 12, the file must be kept for three years after study termination. The stored document will include the subject identification list and subject consent. If storage of the investigator site file becomes impossible at the respective site, the investigator must alert the sponsor to this. The investigator will protect the personal information of the subjects in accordance with the local laws and regulations. The personal information for each subject will be processed anonymously using coded numbers, such that age, gender, and other personal information cannot be recognized. Identifiable data will be stored with limited access to ensure confidentiality of the records. Study data will be viewed and studied anonymously for research purposes only and can be published if necessary. In the event that the data are published, the personal information of the subjects will be kept confidential.

All original files related to the subjects (medical records) must be stored for the longer of the duration mandated by either the law regarding the clinical trial site (hospital, research organization, or clinic) or the ICH clinical trial management regulation (GCP). Medical records must not be destroyed before written consent is obtained from the sponsor.

## Monitoring quality control, government audit

This study will be monitored according to the Korean Food and Drug Administration and Clinical Trial Management Protocol by ICH (ICH topic E6, 1996, and KGCP). Monitoring will be conducted by a monitoring agent designated by the sponsor. The monitoring agent for each study site will regularly visit the site. The sponsor or sponsor’s proxy, as well as the health authorities, must be allowed to audit all documents related to the study, as well as other data from the study sites, including the investigator site file, the completed case report forms, the investigational products, medical records / subject files, etc.

The protocol, information acquisition process for each step, data handling and management, and the clinical trial result report are subject to quality assurance to monitor whether the relevant laws are being upheld. An audit may be conducted at a random time point during or after the study period in order to ensure the validity and verity of the information related to this study.

## Change of protocol

If the protocol is to be changed, the change should be documented on documents related to protocol change (plan of change). For major changes (e.g. changes to a significant portion or important changes), the documentation must be provided to the related parties and to the IRB for approval. The major changes can only be implemented after approval.

Minor protocol changes, including administrative changes, will be documented and stored with the sponsor and at study sites, and will be submitted to the IRB or the health authority according to the relevant policies.

All changes that may impact a subject’s consent must be applied after renewed consent from the subjects (refer to Section 8.2).

## Principles of clinical study reports and data presentation

## Clinical study report

The clinical study report will be drawn up after study termination, based on the principal investigator’s advice, etc. and in accordance with the ICH E3 guideline.

## Data presentation

The first presentation regarding this study will include analysis of the primary endpoint based on data collected from all study sites. The investigator or investigator’s proxy should notify the sponsor if there is any plan to publish or present data related to this study. All data to be published or presented (as an abstract, press release, oral presentation, etc.) should be sent to the sponsor for review (either the entirety or part of a draft). The sponsor will not object to, or deny, any presentation, but reserves the right to delay the timing of the presentation for protection of intellectual property or based on their commercialization strategy.

# References

| 1. | AstraZeneca. Vimovo prescribing Information; 2012. |
| --- | --- |
| 2. | NIH. Daily Med. current medication information. [Online]. [cited 2014 6. Available from:  [http://dailymed.nlm.nih.gov/dailymed/lookup.cfm?setid=e2e18e10-6f02-4ccc-842d-](http://dailymed.nlm.nih.gov/dailymed/lookup.cfm?setid=e2e18e10-6f02-4ccc-842d-1962a3838b74) [1962a3838b74.](http://dailymed.nlm.nih.gov/dailymed/lookup.cfm?setid=e2e18e10-6f02-4ccc-842d-1962a3838b74) |
| 3. | Dugowson CE, Gnanashanmugam P. Nonsteroidal Anti-Inﬂammatory Drugs. In Phys Med Rehabil Clin N Am; 2006. p. 347-354. |
| 4. | Shin JM, Sachs G. Pharmacology of Proton Pump Inhibitors. In Curr Gastroenterol Rep;  2008. p. 528-534. |
| 5. | Hanmi Pharmaceutical. An open-label, randomised, cross-over, single-dose study to compare the safety and pharmacokinetic characteristics of HCP1004 and Vimovo tablet 500/20 mg in healthy Korean adult male volunteers. (Protocol No. HM-ESNP-102); 2013. |
| 6. | Bang CS, Baik GH. Complications and Management of Peptic Ulcer Disease. In The Korean Journal of Helicobacter and Upper Gastrointestinal Research; 2014. p. 18-23. |
| 7. | Ramakrishnan K, Salinas R. Peptic ulcer disease. In Am. Family Physician; 2007. p. 1005-1012. |
| 8. | Milosavljevic T, Kostić-Milosavljević M, Jovanović I, Krstić M. Complications of peptic ulcer disease. In Digestive Diseases; 2011. p. 491-493. |
| 9. | Martinez J, Mattu A. Abdominal pain in the elderly. In Emergency Medicine Clin. North Am.; 2006. p. 371-388. |
| 10. | Lanza F, Chan FKL, Quigley EMM, Gast. PPCoACo. Guidelines for Prevention of NSAID-Related Ulcer Complications. In Am J Gastroenterol; 2009. p. 728-738. |
| 11. | Patrignani P, Tacconelli S, Bruno A, Sostres C, Lanas A. Managing the Adverse Effects of Nonsteroidal Anti-inflammatory Drugs. In Expert Review of Clinical Pharmacology; 2011. p. 605-621. |
| 12. | Lanas A, Hirschowitz B. Toxicity of NSAIDs in the stomach and duodenum. In Eur. J. Gastroenterol. Hepatol.; 1999. p. 375-381. |
| 13. | Hawkey C, Karrasch J, Szczepanski L, al. Omeprazole compared with misoprostol for ulcers associated with nonsteroidal antiinflammatory drugs. Omeprazole versus Misoprostol for NSAID-Induced Ulcer Management. In N. Engl. J. Med.; 1998. p. 727-734. |
| 14. | Yeomans N, Tulassay Z, Juhasz L, al. A comparison of omeprazole with ranitidine for ulcers associated with nonsteroidal antiinflammatory drugs. Acid Suppression Trial: |

|  | Ranitidine versus Omeprazole for NSAID-associated Ulcer Treatment (ASTRONAUT) study group. In N. Engl. J. Med. 338; 1998. p. 719-726. |
| --- | --- |
| 15. | Yeomans N, Lanas A, Talley N, al. Prevalence and incidence of gastroduodenal ulcers during treatment with vascular protective doses of aspirin. In Aliment Pharmacol. Ther.; 2005. p. 795-801. |
| 16. | Watson D, Harper S, Zhao P, al. Gastrointestinal tolerability of the selective cyclooxygenase-2 (COX-2) inhibitor rofecoxib compared with nonselective COX-1 and COX-2 inhibitors in osteoarthritis. In Arch. Intern. Med.; 2000. p. 2998-3003. |
| 17. | Moore R, Derry S, Makinson G, McQuay H. Tolerability and adverse events in clinical trials of celecoxib in osteoarthritis and rheumatoid arthritis: systematic review and meta-analysis of information from company clinical trial reports. In Arthritis Res. Ther.; 2005. p. R644–R665. |
| 18. | Hawkey C, Jones R, Yeomans N, al. Efficacy of esomeprazole for resolution of symptoms of heartburn and acid regurgitation in continuous users of non-steroidal anti-inflammatory drugs. In Aliment Pharmacol. Ther; 2007. p. 813-821. |
| 19. | Scheiman J, Yeomans N, Talley N, al. Prevention of ulcers by esomeprazole in at-risk patients using non-selective NSAIDs and COX-2 inhibitors. In Am. J. Gastroenterol.; 2006. p. 701-710. |
| 20. | Roberts DN, Miner PB. Safety aspects and rational use of a naproxen + esomeprazole combination in the treatment of rheumatoid disease. In Drug, Healthcare and Patient Safety; 2011. p. 1-8. |
| 21. | Sostek M, Fort J, Estborn L, Vikman K. Long-term safety of naproxen and esomeprazole magnesium fixed-dose combination: phase III study in patients at risk for NSAID-associated gastric ulcers. In Curr Med Res Opin; 2011. p. 847-854. |
| 22. | Holtmann G, Talley NJ, Liebregts T, Adam B, Parow C. A Placebo-Controlled Trial of Itopride in Functional Dyspepsia. In N Engl J Med; 2006. p. 832-840. |
| 23. | Rabeneck L, Goldstein J, Vu A, Mayne T, Rublee D. Valdecoxib is associated with improved dyspepsia-related health compared with nonspecific NSAIDs in patients with osteoarthritis or rheumatoid arthritis. In Am J Gastroenterol.; 2005. p. 1043-1050. |
| 24. | INTERNATIONAL CONFERENCE ON HARMONISATION OF TECHNICAL REQUIREMENTS FOR REGISTRATION OF PHARMACEUTICALS FOR HUMAN USE. CHOICE OF CONTROL GROUP AND RELATED ISSUES IN CLINICAL TRIALS (E10). ICH; 2000. |
| 25. | Moayyedi P, Duffett S, Braunholtz D, Mason S, Richards DG, Dowell AC, et al. The Leeds Dyspepsia Questionnaire: a valid tool for measuring the presence and severity of |

|  | dyspepsia. In Alimentary Pharmacol. Ther.; 1998. p. 1257-1262. |
| --- | --- |
| 26. | Svedlund J, Sjodin I, Dotevall G. GSRS - a clinical ration scale for gastrointestinal symptoms in patients with irritable bowel syndrome and peptic ulcer disease. In Dig Dis Sci.; 1988. p. 129-134. |
| 27. | Revicki D, Wood M, Wiklund I, al. Reliability and validity of the Gastrointestinal Symptom Rating Scale in patients with gastroesophageal reflux disease. In Qual Life Res; 1998. p. 75-83. |
| 28. | Mones J, Adan A, Segu J, Lopez J, Artes M, Guerrero T. Quality of life in funtional dyspepsia. In Dig. Dis. Sci.; 2002. p. 20-26. |
| 29. | Bolognese J, Schnitzer T, Ehrich E. Response relationship of VAS and Likert scales in osteoarthritis efficacy measurement. In Osteoarthritis Cartilage; 2003. p. 499-507. |
| 30. | Hawkey C, Talley NJ, Yeomans ND, Jones R, Sung JJ, Långström G, et al. Improvements with Esomeprazole in Patients with Upper Gastrointestinal Symptoms Taking Non-Steroidal Antiinflammatory Drugs, Including Selective COX-2 Inhibitors. In Am J Gastroenterol; 2005. p. 1028-1036. |
| 31. | Junghard O, Wiklund I. What is a clinically relevant difference in patient-reported outcomes in the treatment of reflux disease? In Clinical Ther; 2003. p. d42-d45. |
| 32. | Tally N, Fullerton S, Junghard O, Wiklund I. Quality of life in patients with endoscopy-negative heartburn: reliability and sensitivity of disease-specific instruments. In AJG; 2001. p. 1998-2004. |

# Appendices

## Appendix 1. Study schedule

| Schedule | Screening & Washout period | | Treatment & Follow-up Period* | |
| --- | --- | --- | --- | --- |
|  | Screening1 | Randomization | | End of Study |
| Weeks | -2 - 0 | 0 | | 12 |
| Day of visit | -14 - 1 | 1 | | 84-87 |
| Visit category | V1 | V2 | | V3 |
| Consent |  |  | |  |
| Screening number assignment |  |  | |  |
| Inclusion / exclusion criteria |  |  | |  |
| Demographic information / medical history / medication history |  |  | |  |
| Physical examination 2 | (including weight) |  | |  |
| Vital signs3 |  |  | |  |
| X-ray (lesion)4 |  |  | |  |
| Pain VAS5 | - |  | |  |
| Randomization |  |  | |  |
| Blood test |  |  | |  |
| Blood chemistry test 6 |  |  | |  |
| Hematological test 7 |  |  | |  |
| Pregnancy test8 |  |  | |  |
| Drug administration |  |  | |  |
| Distribution of study drug / ancillary drug / rescue drug |  |  | |  |
| Collection of study drug / ancillary drug / rescue drug |  |  | |  |
| LDQ, GSRS, EQ-5D |  |  | |  |
| Distribution of subject log |  |  | |  |
| Collection of subject log |  |  | |  |
| Confirmation of co-administered drugs |  |  | |  |
| Adverse event monitoring |  |  | |  |

1. If additional washout is not needed, screening and randomization can be conducted on Day 1.
2. Height (history), weight (measured). Weight measured only at visit 1.
3. Blood pressure, pulse rate, respiration rate.
4. Not conducted if there is an X-ray result of the lesion taken within 6 months.
5. Conducted only at visit 2 and not at visit 1.
6. ALT, AST, BUN, creatinine, glucose, total bilirubin.
7. CBC (hemoglobin, hematocrit, RBC count, WBC with differential count, platelet count).
8. Conducted only in women of childbearing potential.

- **Additional visit**: Additional visits may be conducted if follow-up observation of adverse events is needed.

## Appendix 2. Investigator survey

#### (Leeds Dyspepsia Questionnaire, LDQ)

| 1 | Over the last FOUR WEEKS have you had any **indigestion** (a pain in the upper abdomen) (see picture)?  YES [ ] NO [ ]  *If the answer is no please go to question 2.*  How severe has your indigestion been over the last FOUR WEEKS?  Very mild [ ] Mild [ ] Moderate [ ] Severe [ ]  Very severe [ ] | 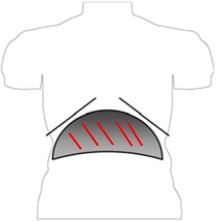 |
| --- | --- | --- |
|  |  | epigastric pain, discomfort, or burning.  * Note the difference in location from #2! |
| 2 | Over the last FOUR WEEKS have you experienced heartburn (a burning feeling behind the breast bone) (see picture)?  YES [ ] NO [ ]  *If the answer is no please go to question 3.*  How severe has your heartburn been over the last FOUR WEEKS?  Very mild [ ] Mild [ ] Moderate [ ] Severe [ ]  Very severe [ ] | 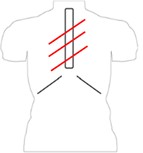 |
|  |  | Chest burning, discomfort, or pain. |
| 3 | Over the last FOUR WEEKS has **food or drink ever stuck behind your breast bone as it went down**?  YES [ ] NO [ ]  *If the answer is no please go to question 4.*  How severe has your symptom been over the last FOUR WEEKS?  Very mild [ ] Mild [ ] Moderate [ ] Severe [ ]  Very severe [ ] | Chest discomfort or pain when swallowing, odynophagia (ddx) or dysphagia. |
| 4 | Over the last FOUR WEEKS have you experienced any **regurgitation** (an acid taste coming up into your mouth from your stomach)?  YES [ ] NO [ ]  *If the answer is no please go to question 5.*  How severe has your regurgitation been over the last FOUR WEEKS?  Very mild [ ] Mild [ ] Moderate [ ] Severe [ ]  Very severe [ ] | Reflux of gastric contents (reflux) (ddx) belching, vomiting, or rumination. |

| 5 | Over the last FOUR WEEKS have you noticed excessive **burping or belching**?  YES [ ] NO [ ]  *If the answer is no please go to question 6.*  How severe has your belching been over the last FOUR WEEKS?  Very mild [ ] Mild [ ] Moderate [ ] Severe [ ]  Very severe [ ] | Belching, oral expulsion of gas only. |
| --- | --- | --- |
| 6 | Over the last FOUR WEEKS have you experienced any **nausea** (a feeling of sickness without actually being sick)?  YES [ ] NO [ ]  *If the answer is no please go to question 7.*  How severe has your nausea been over the last FOUR WEEKS?  Very mild [ ]  Mild [ ] Moderate [ ]  Severe [ ]  Very severe [ ] | Nausea, retching. |
| 7 | Over the last FOUR WEEKS have you experienced any **vomiting**?  YES [ ] NO [ ]  *If the answer is no please go to question 8.*  How severe has your vomiting been over the last FOUR WEEKS?  Very mild [ ] Mild [ ] Moderate [ ] Severe [ ]  Very severe [ ] | Accompanied by nausea or retching (ddx) reflux, rumination, belching. |
| 8 | Over the last FOUR WEEKS have you noticed an  **excessive feeling of fullness after eating**?  YES [ ] NO [ ]  *If the answer is no please go to question 9.*  How severe has your fullness been over the last FOUR WEEKS?  Very mild [ ] Mild [ ] Moderate [ ] Severe [ ]  Very severe [ ] | Post-prandial fullness, incorrect sensation of fullness. |
| 9 | Which, if any, of these symptoms has been the most troublesome to you over the last FOUR WEEKS?  TICK ONE BOX ONLY   1. Heartburn [ ] 2. Regurgitation [ ] 3. Indigestion [ ] 4. Belching [ ] 5. Nausea [ ] 6. Vomiting [ ] 7. Excessive fullness [ ] 8. None of these has troubled me [ ] |  |

## Appendix 3. Subject survey

| S |  |  |  |  |  |
| --- | --- | --- | --- | --- | --- |
|  |  | - |  |  |  |
|  |  |  |  |  |  |

| **Protocol number** | **Naxozol_P4_1** | | | | | | | | | | | | |
| --- | --- | --- | --- | --- | --- | --- | --- | --- | --- | --- | --- | --- | --- |
| **Screening number** |  | | | | | | | | | | | | |
| **Randomization number** |  | | | | | | | | | | | | |
|  |  | **R** |  | | |  | |  | |  | |  |  |
|  |  |  |  |  |  | - | |  |  |  |  |  |  |
|  |  |  |  | | |  | |  | |  | |  |  |
| **Subject initials** |  | | | | | | | | | | | | |
|  |  | | |  | | |  | |  | |  | | |
|  |  |  |  |  | | |  | |  | |  |  |  |
| **Date of visit** | **Visit 2** | | | | YY MM DD | | | | | | | | |
|  | **Visit 3** | | | |  |  |  |  |  |  |  |  |  |
| **Investigator** |  | | | | | | | | | | | | |

- - Thank you very much for participating in our clinical trial.
  - The information provided on this form will not be used for purposes other than this study.
  - Please fill out the form as accurately and credibly as possible.
  - Please return this form after completion.
  - Thank you again for participating in this study.

**Pain (VAS)**

| Protocol number | Visit | Screening number | | | | Randomization number | | | | Initials |
| --- | --- | --- | --- | --- | --- | --- | --- | --- | --- | --- |
| Naxozol_P4_1 |  | S |  |  |  | R |  |  |  |  |

Please indicate your pain level by drawing a line across the straight line below (VAS):

**(Pain VAS)**

| **No pain** |  | **Worst pain imaginable** |
| --- | --- | --- |
|  |  |  |

#### Gastrointestinal Symptom Rating Scale (GSRS)

Please read the following and check the number that corresponds to your experience over the past 3 months.

- 1.
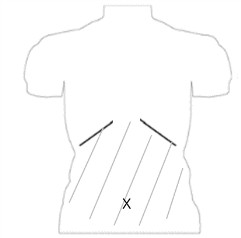
**Abdominal pain**

This means pain in the entire area of the abdomen (below the ribs, not in the chest).

[0]. No pain, or momentary pain.

[1]. Pain or discomfort that occurs intermittently and hinders activity.

[2]. Prolonged pain or discomfort that required alleviation and hinders many social activities.

[3]. Severe and debilitating pain that affects all social activities.

#### Heartburn


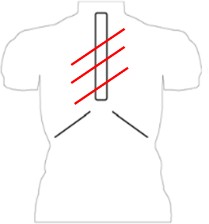
This means burning pain in the esophagus area (post-sternal).

[0]. No burning, or momentary burning.

[1]. Short-term discomfort that occurs intermittently.

[2]. Prolonged discomfort that occurs often and requires alleviation.

[3]. Prolonged discomfort that is temporarily alleviated by antacids.

#### Acid regurgitation

This means unpleasant reflux of acidic fluid from the stomach towards the chest.

[0]. No regurgitation, or momentary regurgitation.

[1]. Bothersome regurgitation that occurs intermittently.

[2]. Regurgitation that occurs 1-2 times a day and requires alleviation.

[3]. Regurgitation that occurs many times a day and is only temporarily and ineffectively alleviated by antacids.

#### Burning sensation in the epigastrium


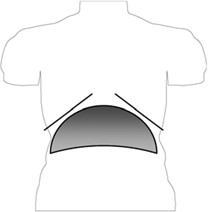
This means a burning sensation in the middle of the upper abdomen.

[0]. No burning sensation, or momentary sensation.

[1]. Short-term discomfort that occurs intermittently; no antacids or food needed between meals.

[2]. Prolonged discomfort that occurs frequently and requires food or antacids between meals.

[3]. Prolonged discomfort that often requires food or antacids between meals.

#### Nausea and vomiting

[0]. No nausea.

[1]. Short-term nausea that occurs intermittently.

[2]. Prolonged nausea that occurs frequently without vomiting.

[3]. Continuous nausea with frequent vomiting.

#### Borborygmus

This means a gurgling sound in the abdomen.

[0]. No borborygmus or intermittent borborygmi.

[1]. Bothersome, short-term borborygmi that occur intermittently.

[2]. Prolonged, frequent borborygmi that are inhibited by movement and do not hinder social activities.

[3]. Continuous borborygmi that severely hinder social activities.

#### Abdominal distension

This means fullness, a feeling of indigestion, and flatulence.

[0]. No distension, or momentary distension.

[1]. Short-term discomfort that occurs intermittently.

[2]. Prolonged discomfort that occurs frequently and can be increased by tight-fitting clothes.

[3]. Continuous discomfort that severely hinders social activities.

#### Eructation

This is expulsion of gas through the mouth without rumination, which is regurgitation of gastric contents, and vomiting.

[0]. No eructation, or momentary eructation.

[1]. Bothersome eructation that occurs intermittently.

[2]. Frequent eructation that hinders some social activities.

[3]. Frequent eructation that severely hinders social activities.

#### (EQ-5D)

Please read the following and check the number that best corresponds to your status.

1. **Exercise**
2. I have no issues with walking.
3. I have some issues with walking.
4. .I need to sit / lie down all the time.

#### Self-care

1. I have no issues with bathing or dressing.
2. I have some issues with bathing or dressing.
3. I cannot bathe or dress by myself.

#### Daily activities (work, study, housework, family, leisure)

1. I have no issues with daily activities.
2. I have some issues with daily activities.
3. I cannot perform daily activities.

#### Pain / discomfort

1. I do not have pain or discomfort.
2. I have moderate pain or discomfort.
3. I have severe pain or discomfort.

#### Anxiety / depression

1. I am neither anxious nor depressed.
2. I am somewhat anxious or depressed.
3. I am severely anxious or depressed.

####
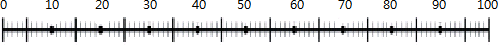
How would you rate your overall health status?

Very bad Very good

#### Thank you for taking the time to answer the questions above.

## Appendix 4. Subject log

| Protocol number | Screening number | | | | Randomization number | | | | Subject initials | | | | |
| --- | --- | --- | --- | --- | --- | --- | --- | --- | --- | --- | --- | --- | --- |
| Naxozol_P4_1 | S |  |  |  | R |  |  |  |  |  |  |  |  |

If you have taken the ancillary drug (Hanmi Almagate 500 mg tablet) or the rescue drug (Hanmi Susphen ER 650 mg tablet), please fill in the form below.

| **Date administered**  (e.g.: 2014 July 10  = **1 4 0 7 1 0**  Please enter one number per box). | | | | | | | **ⓛ Ancillary drug** | **② Rescue drug** |
| --- | --- | --- | --- | --- | --- | --- | --- | --- |
|  |  |  |  |  |  |  | **Hanmi Almagate 500 mg tablet**  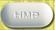 | **Susphen ER 650 mg tablet**  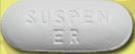 |
|  |  |  |  |  |  |  | Take if gastrointestinal dysfunction is severe beyond toleration. Do not exceed 6 tablets per day. | Take if pain is severe beyond toleration. Do not exceed 4 tablets per day. |
| 1 |  |  |  |  |  |  | Tablets | Tablets |
|  | Y | Y | M | M | D | D |  |  |
| 2 |  |  |  |  |  |  | Tablets | Tablets |
|  | YY MM DD | | | | | |  |  |
| 3 |  |  |  |  |  |  | Tablets | Tablets |
|  | YY MM DD | | | | | |  |  |
| 4 |  |  |  |  |  |  | Tablets | Tablets |
|  | YY MM DD | | | | | |  |  |
| 5 |  |  |  |  |  |  | Tablets | Tablets |
|  | YY MM DD | | | | | |  |  |
| 6 |  |  |  |  |  |  | Tablets | Tablets |
|  | YY MM DD | | | | | |  |  |
| 7 |  |  |  |  |  |  | Tablets | Tablets |
|  | YY MM DD | | | | | |  |  |
| 8 |  |  |  |  |  |  | Tablets | Tablets |
|  | YY MM DD | | | | | |  |  |
| 9 |  |  |  |  |  |  | Tablets | Tablets |
|  | YY MM DD | | | | | |  |  |
| 10 |  |  |  |  |  |  | Tablets | Tablets |
|  | YY MM DD | | | | | |  |  |

## Appendix 5. Signature form

## Sponsor’s signature

Signature:

Protocol approval

Protocol number: Naxozol_P4_1

Version number: Version 1.2

I hereby confirm that this protocol has been approved.

| Study Principal Investigator | |
| --- | --- |
| (Signature: above) |  |
| Name, degree | Sung Hwan Moon, MD, PhD |
| Position | Professor |
| Institution | Department of Orthopedics, Severance Hospital |
| Address | 50-1 Yonsei-ro, Seodaemun-gu, Seoul |

## Principal Investigator’s Signature

Signature:

Protocol number: Naxozol_P4_1

Version number: Version 1.2

I hereby confirm that I will conduct this study according to this protocol, clinical trial management policies, and other relevant laws.

Principal Investigator

| (Signature: above) |  | Date |
| --- | --- | --- |
| Name, degree |  |  |
| Position |  |  |
| Institution |  |  |
| Address |  |  |

## Appendix 6. Compensation clause

**Compensation clause**

1. Principles of compensation
   1. Physical damage to the subject, including death, will be compensated.
   2. The subject will be compensated for the necessary medical costs, within the compensation insurance limit, if damage related to the study occurs.
   3. The subject will be compensated for the necessary medical costs if damage occurs during treatment of any adverse events associated with this study.
2. Principles of non-compensation
   1. Damage due to adverse events associated with drugs not related to the investigational products.
   2. Compensation for failure to provide efficacy or benefit for the indications of the investigational products.
   3. Damage incurred by violation of the protocol agreed upon by both parties.
   4. Damage incurred by the carelessness of the subject or subject’s guardian (e.g. a car accident due to the subject’s carelessness).
   5. Loss that has been amplified by the subject either intentionally or by their serious fault.
   6. Damage due to a clear error or neglect of duty by the investigator.
3. Criteria for compensation evaluation
   1. The level of compensation must be appropriate for the nature, degree, and duration of the damage, and must correspond to the usual compensation for similar damage as mandated by Korean legislation.
   2. If there is a disagreement between the subject’s proxy and the sponsor on the level of compensation, expert consultation that can be accepted by both parties is necessary.

The sponsor will ensure, based on the aforementioned clauses, that the subjects are not disadvantaged in any way by their participation in this study. If a problem occurs due to this study, the sponsor will be responsible according to the compensation clause.

October 20, 2014

Clinical trial sponsor: Sung Hwan Moon, Professor, Department of Orthopedics, Severance Hospital.

# 12. Attachments: Documents Controlled & Filed Separately

## Study sites, principal investigator / sub-investigator / managing pharmacist and sponsor / contract organization

Attached separately (Attachment 1).

- List of clinical trial sites.
- List of names and positions of principal investigators.
- List of names and positions of sub-investigators.
- List of names of managing pharmacists.
- Contact information for the sponsor.
- Contact information for the contract research organization.

## Explanation and Consent Form for Subject Consent

Attached separately (Attachment 2).
