# Supplementary material for: A comparative study of the efficacy of NAXOZOL compared to celecoxib in patients with osteoarthritis
Source: PLoS One. 2020 Jan 27;15(1):e0226184. doi: 10.1371/journal.pone.0226184 (PMC6984721; doi:10.1371/journal.pone.0226184)
Supplement: S2 File — (DOCX) [file pone.0226184.s009.docx]

**임상시험 계획서**

| 임상시험 계획서 번호 | Naxozol_P4_1 |
| --- | --- |
| 임상시험 제목 | 골관절염 환자를 대상으로 낙소졸과 Celecoxib의 위장 장애 보호 효과와 통증 개선 효과를 비교하기 위한 전향적, 무작위 배정, 이중 눈가림, 이중 위약, 활성 대조, 다기관, 중재 연구 |
| 임상시험 단계 | 제 4상 |
| 의뢰자 | 문성환 (세브란스병원 정형외과) |
| 임상시험 계획서 | Version 1.2 |

**목 차**

[임상시험 요약 (synopsis) 6](#_Toc400369612)

[1. 의뢰자, 시험자 및 임상시험 행정 관련 정보 12](#_Toc400369613)

[1.1. 임상시험 제목 12](#_Toc400369614)

[1.2. 임상시험 의뢰자 및 주소 12](#_Toc400369615)

[1.3. 임상시험 실시기관·책임자·담당자·관리자 12](#_Toc400369616)

[1.3.1. 임상시험 실시기관 12](#_Toc400369617)

[1.3.2. 임상시험 책임자 및 담당자의 성명 및 직명 12](#_Toc400369618)

[1.3.2.1. 임상시험 책임자 및 담당자의 성명 및 직명 12](#_Toc400369619)

[1.3.2.2. 임상시험용 의약품 관리 약사 성명 12](#_Toc400369620)

[1.4. 자료 안전성 모니터링 위원회 12](#_Toc400369621)

[2. 시험 배경 및 근거 13](#_Toc400369622)

[2.1. 물질명 및 물리화학적 성상 요약 13](#_Toc400369623)

[2.2. 비임상 대사, 약동학, ADME 연구 결과 요약 13](#_Toc400369624)

[2.3. 약물 작용 기전 및 생물학적 표적 기관에 대한 요약 14](#_Toc400369625)

[2.4. 임상 약리학 연구 결과 요약 14](#_Toc400369626)

[2.5. 임상시험 대상군 특성 요약 14](#_Toc400369627)

[2.6. 임상시험 대상군에 대한 임상시험용 의약품 투약 근거 15](#_Toc400369628)

[2.7. 임상시험용 의약품의 유효성과 안전성 요약 16](#_Toc400369629)

[2.8. 임상시험용 의약품의 사용 경험 16](#_Toc400369630)

[2.9. 투여에 따른 위험 편익 고찰 16](#_Toc400369631)

[2.10. 임상시험 디자인 고찰 17](#_Toc400369632)

[2.11. 결론 17](#_Toc400369633)

[3. 임상시험 목적 18](#_Toc400369634)

[4. 임상시험계획 19](#_Toc400369635)

[4.1. 임상시험 전반적인 방법 및 계획 19](#_Toc400369636)

[4.2. 임상시험 디자인에 대한 검토 논의 21](#_Toc400369637)

[4.2.1. 임상시험 디자인에 대한 검토 22](#_Toc400369638)

[4.2.2. 치료 기간에 대한 검토 22](#_Toc400369639)

[4.2.3. 용량 선정 및 선정 근거 22](#_Toc400369640)

[4.2.4. 대조군 선정에 대한 검토 23](#_Toc400369641)

[4.2.5. 시험군 배정에 대한 검토 23](#_Toc400369642)

[4.2.6. 특수 인구 집단 시험대상자의 포함 23](#_Toc400369643)

[4.3. 임상시험 대상군의 선정 23](#_Toc400369644)

[4.3.1. 선정 기준 23](#_Toc400369645)

[4.3.2. 제외 기준 24](#_Toc400369646)

[4.4. 시험대상자 배정/ 임상시험용 의약품 투약 25](#_Toc400369647)

[4.4.1. 스크리닝 번호 및 시험대상자 번호 부여 25](#_Toc400369648)

[4.4.2. 시험군 배정 26](#_Toc400369649)

[4.5. 시험대상자 탈락 기준 26](#_Toc400369650)

[4.5.1. 임상시험 탈락 26](#_Toc400369651)

[4.5.2. 임상시험용 의약품 투약의 중단 27](#_Toc400369652)

[4.6. 시험대상자의 대체 28](#_Toc400369653)

[4.7. 새로운 용량군 추가 28](#_Toc400369654)

[4.8. 임상시험의 조기 종료 28](#_Toc400369655)

[4.9. 임상시험의 종료 29](#_Toc400369656)

[4.10. 기타 고려 사항 29](#_Toc400369657)

[5. 임상시험용 의약품 및 임상시험에 사용되는 기타 의약품 30](#_Toc400369658)

[5.1. 임상시험에 사용되는 의약품 30](#_Toc400369659)

[5.1.1. 시험약 30](#_Toc400369660)

[5.1.1.1. 제품명/코드명 30](#_Toc400369661)

[5.1.1.2. 성상 30](#_Toc400369662)

[5.1.1.3. 성분 및 함량 30](#_Toc400369663)

[5.1.1.4. 보관 조건 30](#_Toc400369664)

[5.1.2. 시험약의 위약 30](#_Toc400369665)

[5.1.3. 대조약 30](#_Toc400369666)

[5.1.3.1. 제품명/코드명 30](#_Toc400369667)

[5.1.3.2. 성상 30](#_Toc400369668)

[5.1.3.3. 성분 및 함량 30](#_Toc400369669)

[5.1.3.4. 보관조건 30](#_Toc400369670)

[5.1.4. 대조약의 위약 30](#_Toc400369671)

[5.2. 예측 용법 용량 31](#_Toc400369672)

[5.2.1. 시험약의 예측 용법 용량 31](#_Toc400369673)

[5.2.2. 대조약의 예측 용법 용량 31](#_Toc400369674)

[5.3. 임상시험에 사용되는 기타 의약품 32](#_Toc400369675)

[5.3.1. 병용 약물 및 치료 32](#_Toc400369676)

[5.3.1.1. 보조약물 32](#_Toc400369677)

[5.3.1.2. 구제약물 32](#_Toc400369678)

[5.3.2. 임상시험 참여 전 금기 의약품 또는 치료 33](#_Toc400369679)

[5.3.3. 임상시험 중 사용이 금지된 의약품 및 치료 33](#_Toc400369680)

[5.3.4. 시험약의 사용 상 주의 사항 34](#_Toc400369681)

[5.3.4.1. 시험약의 이상 반응 Adverse Events 34](#_Toc400369682)

[5.3.4.2. 시험약의 금기 사항 Contraindications 36](#_Toc400369683)

[5.3.4.3. 시험약의 경고 사항 37](#_Toc400369684)

[5.3.4.4. 시험약의 주의 사항 Precautions 38](#_Toc400369685)

[5.3.4.5. 약물 상호 작용 Drug Interactions 39](#_Toc400369686)

[5.3.4.6. 특수 인구 집단 42](#_Toc400369687)

[5.3.5. 대조약의 사용 상 주의 사항 43](#_Toc400369688)

[5.3.5.1. 대조약의 이상반응 Adverse Events 43](#_Toc400369689)

[5.3.5.2. 대조약의 금기사항 Contraindications 45](#_Toc400369690)

[5.3.5.3. 대조약의 경고사항 46](#_Toc400369691)

[5.3.5.4. 대조약의 주의사항 46](#_Toc400369692)

[5.4. 포장 및 라벨링 53](#_Toc400369693)

[5.5. 공급, 취급 및 보관 54](#_Toc400369694)

[5.6. 임상시험용의약품의 교부 54](#_Toc400369695)

[5.7. 치료 순응도 55](#_Toc400369696)

[5.8. 눈가림법 55](#_Toc400369697)

[5.9. 응급 눈가림 해제 56](#_Toc400369698)

[5.10. 과다 투여에 대한 처치 56](#_Toc400369699)

[5.11. 시험 종료 후 시험대상자의 진료 및 치료 57](#_Toc400369700)

[6. 임상시험 절차 및 평가 58](#_Toc400369701)

[6.1. 평가 일정 58](#_Toc400369702)

[6.2. 인구학적정보 및 기타 기저값 평가 60](#_Toc400369703)

[6.3. 안전성 평가 60](#_Toc400369704)

[6.3.1. 이상반응 61](#_Toc400369705)

[6.3.1.1. 이상반응 정의 61](#_Toc400369706)

[6.3.2. 임부에 대한 노출 67](#_Toc400369707)

[6.3.3. 임상검사실 검사 평가 67](#_Toc400369708)

[6.3.4. 활력징후, 신체 검사 및 기타 평가 68](#_Toc400369709)

[6.4. 유효성 평가 68](#_Toc400369710)

[6.4.1. 일차 평가 68](#_Toc400369711)

[6.4.2. 이차 평가 68](#_Toc400369712)

[6.5. 기타 평가 69](#_Toc400369713)

[7. 통계분석 계획 70](#_Toc400369714)

[7.1. 시험대상자 수 산정 70](#_Toc400369715)

[7.2. 무작위 배정 71](#_Toc400369717)

[7.3. 평가 변수 71](#_Toc400369718)

[7.3.1. 일차 평가 변수 71](#_Toc400369719)

[7.3.2. 이차 평가 변수 71](#_Toc400369721)

[7.3.3. 기타 평가 변수 72](#_Toc400369732)

[7.4. 분석군의 정의 72](#_Toc400369733)

[7.5. 통계 분석 계획 73](#_Toc400369734)

[7.5.1. 일반적 고려 사항 73](#_Toc400369735)

[7.5.2. 일차 평가 변수 분석 73](#_Toc400369736)

[7.5.3. 이차 평가 변수 분석 73](#_Toc400369739)

[7.5.4. 인구학적 정보 77](#_Toc400369740)

[7.6. 중간 분석 77](#_Toc400369743)

[8. 임상시험의 윤리적 법률적 측면 78](#_Toc400369744)

[8.1. 시험자의 책임 78](#_Toc400369745)

[8.2. 시험대상자 정보 및 동의서 78](#_Toc400369746)

[8.3. 시험대상자 식별과 비밀 유지 78](#_Toc400369747)

[8.4. 시험대상자에 대한 보상 및 보험 79](#_Toc400369748)

[8.5. 임상시험 심사위원회 79](#_Toc400369749)

[8.6. 정부 기관 79](#_Toc400369750)

[9. 임상시험 관리 80](#_Toc400369751)

[9.1. 증례기록서 관리 80](#_Toc400369752)

[9.2. 근거 자료와 시험대상자 관련 파일 80](#_Toc400369753)

[9.3. 시험자 파일과 자료의 보관 81](#_Toc400369754)

[9.4. 모니터링 품질 보증, 정부 기관의 실사 82](#_Toc400369755)

[9.5. 임상시험 계획서의 변경 82](#_Toc400369756)

[9.6. 임상시험 보고와 자료 발표에 관한 원칙 83](#_Toc400369757)

[9.6.1. 임상시험 보고서 83](#_Toc400369758)

[9.6.2. 자료의 발표 83](#_Toc400369759)

[10. References 84](#_Toc400369760)

[11. Appendices 부록 87](#_Toc400369761)

[Appendix 1. 임상시험 진행 일정표 87](#_Toc400369762)

[Appendix 2. 시험자 설문지 88](#_Toc400369763)

[Appendix 3. 시험대상자 설문지 90](#_Toc400369763)0

[Appendix 4. 시험대상자 일지 94](#_Toc400369764)4

[Appendix 5. 서명양식 95](#_Toc400369765)5

[의뢰자 서명 95](#_Toc400369766)5

[시험책임자 서명 96](#_Toc400369767)6

[Appendix 6. 피해자 보상 규약 97](#_Toc400369768)7

[12. Attachments별첨: Documents Controlled & Filed Seperately 별도 관리되는 문서](#_Toc400369769) 98

[임상시험 실시기관, 시험책임자/담당자/관리약사 및 의뢰자/수탁기관](#_Toc400369770)(별첨1)

[시험대상자 동의를 위한 설명문 및 동의서](#_Toc400369771)(별첨2)

**용어 및 약어**

| ACE | Angiotensin Converting Enzyme |
| --- | --- |
| ACG | American College of Gastroenterology |
| AGEP | Actue Generalized Exanthematous Pustulosis |
| ALB | Albumin |
| ALP | Alkaline Phosphatase |
| ALT | Alanine Aminotransferase |
| AST | Aspartate Aminotransferase |
| BUN | Blood Urea Nitrogen |
| CABG | Coronary Artery Bypass Graft |
| CBC | Complete Blood Count |
| COX | Cyclo-Oxygenase |
| CPK | Creatine Phosphkinase |
| CRC | Clinical Research Coordinator |
| CRF | Case Report Form |
| CRO | Contract Research Organization |
| CTCAE | Common Terminology Criteria for Adverse Events |
| CYP | Cytochrome |
| DBP | Diastolic Blood Pressure |
| DRESS | Drug Rash with Eosinophilia and Systemic Symptom |
| DSMB | Data and Safety Monitoring Board |
| ECL | Enterochromaffin-Like |
| ELISA | Enzyme-Linked Immunosorbent Assay |
| FAS | Full Analysis Set |
| GCP | Good Clinical Practice |
| GFR | Glomerular Filtration Rate |
| γ-GT | Gamma Glutamyltransferase |
| GSRS | Gastrointestinal Symptom Rating Scale |
| Hb | Hemoglobulin |
| HCV | Hepatitis C Virus |
| HR | Heart Rate |
| ICH | International Conference on Harmonisation of Technical Requirements for Registration of Pharmaceuticals for Human Use |
| IND | Investigational New Drug |
| ITT | Intent-To-Treat |
| KGCP | Korean Good Clinical Practice |
| LDA | Low Dose Aspirin |
| LDH | Lactate Dehydrogenase |
| LDQ | Leeds Digestion Questionnaire |
| MCD | Minimal Change Disease |
| MCID | Minimal Clinically Important Difference |
| MCTD | Mixed Connective Tissue Disease |
| MedDRA | Medical Dictionary for Regulatory Activities |
| MRI | Magnetic Resonance Imaging |
| NPN | Non-Protein Nitrogen |
| PLT | Platelet |
| PR | Purse Rate |
| PPI | Proton Pump Inhibitor |
| PTT | Partial Thromboplastin Time |
| PT | Preferred Term |
| RBC | Red Blood Cell |
| SAE | Serious Adverse Event |
| SLE | Systemic Lupus Erythematosus |
| SOC | System Organ Class |
| SODA | Severity of Dyspepsia Assessment |
| SUSAR | Suspected Unexpected Serious Adverse Reaction |
| TEN | Toxic Epidermal Necrolysis |
| UNL | Upper Normal Limit |
| VAS | Visual Analogue Scale |
| WBC | White Blood Cell |
| WHO | World Health Organization |
| WHOART | WHO Adverse Reactions Terminology |

# 임상시험 요약 (synopsis)

| Trial Title | 골관절염 환자를 대상으로 낙소졸과 Celecoxib의 위장 장애 보호 효과와 통증 개선 효과를 비교하기 위한 전향적, 무작위 배정, 이중 눈가림, 이중 위약, 활성 대조, 다기관, 중재 연구  A Prospective, Randomized, Double-blinded, Double-dummy, Active-contolled, Multi-center, Interventional Study to Compare Gastro-protective Effect and Pain Relief Effect of Naxozol Compared to Celecoxib in Patients with Osteoarthritis | | |
| --- | --- | --- | --- |
| Trial Number | Naxozol_P4_1 | Version | 1 |
| Sponsor | 문성환 (세브란스병원 정형외과) | | |
| Phase | 제 4상 (연구자 주도 임상시험) | | |
| IND | 해당  해당 안됨 | | |
| 임상시험 실시기관/국가 | 10개 기관/대한민국 | | |
| 임상시험 예상기간 | 임상시험 개시일로부터 약 24개월 | | |
| 시험 목적 | **1차 목적**   - 골관절염 환자를 대상으로 낙소졸 투여 12주 후 LDQ (Leeds Dyspepsia Questionnaire)로 평가되는 위장 장애 보호 효과가 Celecoxib 투약군에 비해 비열등함을 입증한다. | | |
|  | **2차 목적** | | |
|  | - 골관절염 환자를 대상으로 낙소졸 및 Celecoxib 투여 12주 후 GSRS (Gastrointestinal Symptom Rating Scale)로 평가되는 위장 장애 보호 효과를 비교한다. - 골관절염 환자를 대상으로 낙소졸 및 Celecoxib 투여 12주 후 GI (Gatrointestinal) 부작용 발생률 및 GI 부작용으로 인한 약물 중단률을 비교한다. - 골관절염 환자를 대상으로 낙소졸 및 Celecoxib 투여 12주 후 통증 VAS (Visual Analogue Scale)로 평가되는 통증 개선 효과를 비교한다. - 골관절염 환자를 대상으로 낙소졸 및 Celecoxib 투여 12주 후 EQ-5D로 평가되는 삶의 질 개선 효과를 비교한다. - 골관절염 환자를 대상으로 낙소졸 및 Celecoxib 투여 12주 후 환자 복용 순응도를 비교한다. - 골관절염 환자를 대상으로 낙소졸 및 Celecoxib 투여 12주 후 전반적인 안전성을 비교한다. | | |
| 임상시험 디자인 계획 | - 전향적 (Prospective), 무작위 배정 (Randomized), 이중 눈가림 (Double-blinded), 이중 위약 (Double-dummy), 활성 대조 (Active-controlled), 2개 치료군 평행 (Two-arm Parallel), 다기관 (Multi-center) | | |
| 계획된 연구 참여 시험대상자 수 | - 시험군 42명, 대조군 42명 목표, 20%의 중도 탈락율 고려하여 시험군 53명, 대조군 53명, 2개 투여군에 총 106명 이상이 참여할 예정 | | |
| 방문 일정 및 평가 | - Screening & washout period: ~ 14일간 (day -14~day 1 [week -2~0]) - Treatment and F/U period: 12주간 (day 1 ~ day 84 [week 12])   **평가를 위한 방문 일정**   - Visit 1: screening & washout, day -14~day 1 - Visit 2: randomization, day 1 - Visit 3: end of study, 무작위 배정일 기준 day 84±7   **평가 일정**   1. **안전성 및 내약성 평가**   이상반응, 신체 검진, 활력징후, 임상검사실 검사 (혈액학 검사, 혈액화학 검사), 병용약물   - 이상반응: V1-V3 매 방문 시 - 병용약물: V1-V3 매 방문 시 - 활력징후 (vital sign; V/S): V1와 V3 방문 시 - 신체 검진: V1과 V3 방문 시 - 임상검사실 검사:   1. ① 혈액학 검사: V1과 V3 방문 시   2. ② 혈액화학 검사: V1과 V3 방문 시 | | |
| 선정 기준 | 1. 본 임상시험에 대하여 자세한 설명을 듣고 이해한 후, 자의로 참여를 결정하고, 서면 동의한 한국인 2. 동의서 작성 당시 연령이 만 50세 이상의 남녀 3. 임상시험 동안 추적이 가능하고, EQ-5D, VAS 설문지 등을 읽고 작성할 수 있는 등, 임상시험에 요구되는 사항을 준수할 수 있는 자 4. 병력을 통해 신체의 증상이 있는 골관절염이 확인된 환자로서, 방문 2에서 실시한 통증 VAS 검사 상 40mm 이상 5. 스크리닝 결과, 본 연구에 적합하다고 판단된 자 (단, 임상검사실 검사 결과가 참고 범위를 벗어나도 시험자의 판단에 따라 참여할 수 있음) | | |
| 제외 기준 | 1. 현재 임상시험에 참여 중이거나 이전 임상시험 참여 후 30일이 경과하지 않은 자 (참여는 임상시험용 의약품의 최종 투약일을 기준으로 함) 2. 스크리닝 전 6개월 기준으로 알코올 및 기타 약물 남용 기왕력자 3. 일주일 기준 21잔 이상 (일반적으로 소주잔: 55mL; 맥주잔: 200mL; 양주잔: 30mL로 동일하게 취급)의 술을 마시는 경우 4. 최근 5년 이내 합병증 (출혈 [Bleeding], 장천공 [Perforation], 관통을 통한 장누공 [Penetration], 위 유문부 폐쇄 [Gastric Outlet Obstruction])을 동반한 소화성 궤양 병력 환자 5. 스크리닝 방문 전 6개월 이내 합병증을 동반하지 않은 소화성 궤양 환자 또는 현 병력 상 활동성 소화성 궤양 환자 6. 기왕에 알려진 헬리코박터 감염 환자로서 제균 치료를 시행 받지 않은 자 7. 기왕에 알려진 위식도 역류증 환자 8. 안전성 및 유효성 평가에 영향을 줄 수 있는 다음의 관절 질환이 병력 상 확진되었거나 임상 소견 상 의심되는 환자: 패혈성 관절염, 류마티스 관절염과 같은 염증성 관절 질환, 통풍, 재발성 가성 통증, 파제트병, 관절 골절, 조직흑변증, 말단거대증, 혈색소증, 윌슨병, 원발성 골연골증, 과운동 증후군 (Ehlers Danlos Syndrome) 및 기타 콜라겐 유전자 이상 질환 등 9. 임상시험 기간 동안 수술이 예정되어 있는 환자 10. 소화기관의 암 병력을 가진 환자 11. 약물 흡수 장애로 인한 GI disorder 환자 12. 위장관 출혈, 뇌혈관 출혈, 기타 출혈 질환 환자, 심한 혈액 이상 환자 13. 중등증 또는 중증 간장애 환자 (Child Pough Class II 이상) 14. 중증 심부전 환자 및 관상동맥 우회로술 (CABG) 병력 환자 15. 중증 신장애 환자 (CrCl <30mL/min) 16. 치료에도 불구하고 조절되지 않는 심한 고혈압 환자 (SBP ≥160mmHg 또는 DBP ≥100mmHg ) 17. 본 임상시험용 의약품을 구성하고 있는 성분에 대해 알레르기의 기왕력이 있는 경우 18. 기타 NSAIDs나 PPI에 과민증, 알러지가 있는 환자 19. 임상시험 참여 전 금기 의약품 또는 치료를 받고 정해진 기간이 경과하지 않은 시험대상자 (Section 5.3.2 참조) 20. 병용금기 의약품을 투여 중인 환자 (Section 5.3.3 참조) 21. 최근 1년 이내의 골관절염의 해당 관절 수술이력을 가진 환자 22. 임부, 수유부 또는 적절한 피임법 (임상시험용 의약품 투여 21일 전부터 콘돔, 격막, 자궁내 피임 장치, 호르몬 피임 제제 복용 등을 사용하거나 남성 파트너가 정관 절제술을 받은 경우)을 실시하지 않는 가임 여성 (폐경기 여성은 피임법 적용 해당 안됨) 23. 기타 임상적으로 유의한 의학적 또는 정신 의학적 소견으로 시험자가 부적합하다고 판단한 자 | | |
| 시험약 | - 낙소졸 정: 복합제 (Naproxen 500mg+Esomeprazole 20mg)   용법 용량: 1일 2회, 1회 1정. 쪼개거나 씹거나 부수지 말고 식전 30분~60분에 복용  *시험군 : 아침 식전 30분 ~ 60분에 낙소졸 1정, 저녁 식전 30분 ~ 60분에 낙소졸 1정과 쎄레브렉스 위약1캡슐 복용 | | |
| 대조약 | - 쎄레브렉스 캡슐: Celecoxib 200mg   용법: 용량: 1일 1회, 1회 1캡슐. 쪼개거나 씹거나 부수지 말고 식전30분~60분에 복용 (허가 기재 사항: 1일 1회, 1회 1정. 식사와 관계없이 복용)  * 대조군 : 아침 식전 30분 ~ 60분에 낙소졸 위약1정, 저녁 식전 30분 ~ 60분에 낙소졸 위약 1정과 쎄레브렉스 1캡슐 복용 | | |
| 시험대상자당 계획된 임상시험기간 | 시험대상자 당 총 12주 | | |
| 일차 평가 변수 | - 임상시험용 의약품 12주 경구 투여 후 LDQ (Leeds Dyspepsia Questionnaire)의 기저치 대비 평균 변화량 | | |
| 이차 평가 변수 | - 임상시험용 의약품 12주 경구 투여 후 LDQ (Leeds Dyspepsia Questionnaire)의 평균 - 임상시험용 의약품 12주 경구 투여 후 GSRS (Gastrointestinal Symptom Rating Scale)의 기저치 대비 평균 변화량 - 임상시험용 의약품 12주 경구 투여 후 GI 부작용 (Dyspepsia, Diarrhoea, Nausea, Abdominal Pain, Heartburn) 발생률 - 임상시험용 의약품 12주 경구 투여 후 GI 부작용으로 인한 약물 중단률 - 임상시험용 의약품 12주 경구 투여 후 통증 VAS (Visual Analogue Scale)의 기저치 대비 평균 변화량 - 임상시험용 의약품 12주 경구 투여 후 EQ-5D 평균 점수의 기저치 대비 평균 변화량 - 임상시험용 의약품 12주 경구 투여 후 복용 순응도 - 임상시험용 의약품 12주 경구 투여 후 보충 및 구제 약물 사용 여부, 사용한 평균 일수, 하루 당 평균 사용량 (총 사용량/총 사용 일수) 및 임상시험 참여 기간 중 평균 사용량 (총 사용량/임상시험 참여 기간) - 임상시험용 의약품 12주 경구 투여 후 이상반응 발생 여부 및 빈도와 특성 (양상, 중대성, 결과 등) - 임상시험용 의약품 12주 경구 투여 후 신체 검진, 활력징후, 임상검사실 검사 소견 | | |
| 자료 분석 | - 분석군의 정의   **안전성 평가군:** 무작위 배정 후 임상시험용 의약품을 투여 받고 한차례라도 안전성 관련 추적 관찰이 이루어진 시험대상자 집단을 의미한다.  **FAS 평가군:** 무작위 배정 후 임상시험용 의약품을 투여 받고 유효성 평가가 한 차례라도 이루어진 시험대상자 집단을 의미한다.  **PP 평가군:** 무작위 배정 후 임상시험용 의약품을 투여 받고 유효성평가가 한 차례라도 이루어진 시험대상자 집단 중 중대한 계획서 위반이 없는 집단을 의미한다.   - 통계 분석   비열등성 실험이므로, ICH 권고사항에 따라 FA 군 (Modified ITT)과 PP 군 모두를 주 분석군으로 한다.  임상시험 중 결측치가 발생한 경우, Multiple Imputation 방법을 적용하여 결측치를 대체한다.  시험약의 비열등성을 증명하기 위해 두 투여군 간의 기저치 대비 12주째 LDQ 변화량 차이에 대해 97.5% 단측 검정 신뢰구간 (one-sided 97.5% confidence interval)을 구한다. 이 신뢰구간의 상한선 (97.5% upper confidence limit)이 미리 명시된 비열등성 마진인 0.40보다 작으면 귀무가설을 기각하여 비열등성이 증명된다.  비열등성 평가를 제외한 모든 통계분석은 유의수준 (α) 0.05하에서 양측검정 (Two-sided)을 원칙으로 한다.  중간 분석은 실시하지 않는다.  기술계량으로 연속형 변수의 경우 평균, 표준편차, 중앙값, 최소값과 최대값, 범주형 변수의 경우 빈도와 비율 제시를 기본 원칙으로 하고, 필요 시 발생 빈도를 제시한다.  인구학적 그리고 기타 기저 자료, 안전성, 유효성 자료는 각 측정 시점에서 혹은 기저치로부터의 변화를 기술통계량으로 요약하며 투여군 별로 제시한다. 추가적으로 신체 검진과 임상검사실 검사 결과의 정상/비정상 변화에 대해 분할표를 투여군 별로 제시한다.  병용약물은 ATC 용어로 기록하며, 이상반응는 Medical Dictionary (MedDRA 또는 WHOART)를 이용하여 SOC (System Organ Class) 및 PT (Preferred Term)로 표준화한다. | | |

# 의뢰자, 시험자 및 임상시험 행정 관련 정보

## 임상시험 제목

골관절염 환자를 대상으로 낙소졸과 Celecoxib의 위장 장애 보호 효과와 통증 개선 효과를 비교하기 위한 전향적, 무작위 배정, 이중 눈가림, 이중 위약, 활성 대조, 다기관, 중재 연구

## 임상시험 의뢰자 및 주소

| 의뢰자: | 문성환 MD, PhD. (세브란스병원 정형외과) |
| --- | --- |
| 주소 | 서울특별시 서대문구 연세로 50-1 |

## 임상시험 실시기관·책임자·담당자·관리자

### 임상시험 실시기관

본 임상시험은 한국 내 10개 기관에서 진행될 예정이다 (참여기관명은 12 별첨 참조).

임상시험 실시기관의 목록은 임상시험 계획서와 별도 문서로서 관리될 것이다.

### 임상시험 책임자 및 담당자의 성명 및 직명

### 임상시험 책임자 및 담당자의 성명 및 직명

참여기관 시험 책임자의 서명 및 서명 양식은 Appendix 5에 수록하였다.

참여기관 시험 책임자 성명 및 직명은 별도 문서로서 관리될 것이다 (12 별첨 참조)

참여기관 공동 연구자 및 담당자의 성명 및 직명은 별도 문서로서 관리될 것이다 (12 별첨 참조).

### 임상시험용 의약품 관리 약사 성명

참여기관 임상시험용 의약품 관리 약사의 성명에 대한 목록은 별도 문서로서 관리될 것이다. (12 별첨 참조)

## 자료 안전성 모니터링 위원회

본 임상시험에서는 자료 안전성 모니터링 위원회가 설치되지 않을 예정이다.

# 시험 배경 및 근거

## 물질명 및 물리화학적 성상 요약

제품명: 낙소졸 정

활성물질명: 나프록센 (USP) 500mg과 에스오메프라졸스트론튬사수화물 24.60mg (에스오메프라졸로서 20mg)

성상: 미황색 장방형의 필름코팅정제

요약: 낙소졸 정은 나프록센 500mg과 에스오메프라졸스트론튬사수화물 24.60mg 을 포함하고 있으며, 겉은 속방형 제제 에소메졸이 둘러싸고, 속은 장용코팅된 나프록센이 포함된 복합제이다. 낙소졸 정에 포함된 유효 성분은 각각 다음과 같다.

**나프록센:**

Chemical name: (S)-6-methoxy-α-methyl-2-naphthaleneacetic acid

Molecular formula: C_14_H_14_O_3_

Molecular weight: 230.26

**
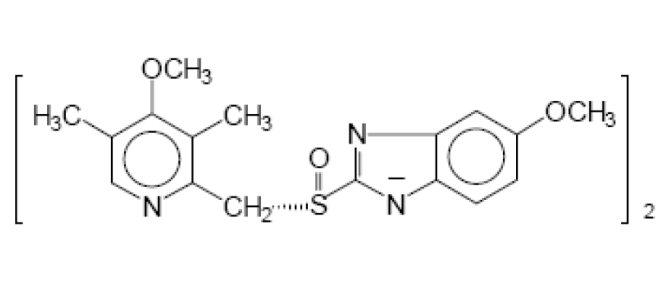
에스오메프라졸 스트론튬사수화물:**

Sr^2+^ • 4H_2_O

Chemical name: bis (5-methoxy-2-[(S)-[(4-methoxy-3,5-dimethyl-2-pyridinyl)methyl] sulfinyl]-1H-benzimidazole-1-yl) strontium tetrahydrate

Molecular formula: C34H36N6O6S2Sr. 4H2O

Molecular weight: 848.50

## 비임상 대사, 약동학, ADME 연구 결과 요약

나프록센 성분의 경우 오랜 기간 동안 사용되어 온 NSAID (non-steroidal anti-inflammatoy drug) 계열 약물로서 독성에 대해 잘 알려져 있다. 랫트를 대상으로 한 나프록센 장기 투여 발암성 시험에서 발암성이 관찰되지 않았다. (1)

Esomeprazole은 omeprazole의 enantiomer이며, omeprazole을 이용한 랫트 대상의 발암성 연구 결과 gastric ECL 세포의 hyperplasia가 보고된 바 있다. 생쥐를 이용한 발암성 연구 결과에서는 omeprazole 투여로 인한 종양 발생 차이가 관찰되지 않았으나 확정적인 결과는 아니었다.

Esomeprazole strontium을 이용한 돌연변이 시험 결과는 음성이었다. (2)

## 약물 작용 기전 및 생물학적 표적 기관에 대한 요약

낙소졸 정은 나프록센과 에스오메프라졸로 구성되어 있다. 나프록센 (naproxen)은 타 non-steroidal anti-inflammatoy drug (NSAID)과 마찬가지로 주로 cyclooxygenase-2 (COX- 2) 효소 활성을 저해하여 arachidonic acid로 부터의 prostaglandin과 thromboxane 합성을 감소시켜서 항염증, 진통, 해열 작용을 하는 의약품이다. (3)

에스오메프라졸은 benzimidazole의 일종으로 위 parietal 세포벽에 존재하는 H+, K+-ATPase 효소를 억제함으로써 위산 분비를 억제하는 proton pump 저해제이며, 타 proton pump 저해제에 비해 더 오랫동안 위장내 pH를 높게 유지할 수 있는 의약품으로 알려져 있다 (pH>4 유지 시간: 타 proton pump inhibitor 12 시간 가량; esomeprazol: 16.8 시간 가량). (4)

## 임상 약리학 연구 결과 요약

낙소졸 정을 대상으로 임상 약리학 연구를 실시한 결과, 공복 상태에서 단회 경구 투여 시 대조약인 비모보 정과 비교하였을 때, 안전성에 차이를 보이지 않았고 유사하게 양호한 내약성이 관찰되었다. (5)

약동학적 평가 결과, C_max_ 및 AUC_last_의 기하평균비와 90% 신뢰구간은 naproxen에서 각각 0.99 (0.94~1.06), 1.00 (0.98~1.01)이었으며, esomeprazole에서 각각 0.99 (0.82~1.18), 1.04 (0.91~1.18)로서, 일반적으로 생물학적 동등성을 인정하는 범위인 0.8 – 1.25에 포함되는 값으로서, 유사한 정도의 체내 노출 (systemic exposure)을 나타내었으며, 결과적으로 naproxen과 esomeprazole 각각 시험약과 대조약에서 약동학적 특성의 동등함이 확인되었다. (5)

## 임상시험 대상군 특성 요약

본 임상시험은 만 50세 이상의 한국인 남녀 골관절염으로 인해 NSAID 치료가 필요한 환자를 대상으로 시험대상자를 모집할 예정이다.

골관절염은 본 임상시험용 의약품의 적응증 대상 질환이므로 본 임상시험용 의약품을 투여 받은 환자들은 치료 효과 측면에서 해택을 받을 수 있는 적절한 대상 질환군 선택으로 판단되었다.

상부 위장관 출혈은 소화성 궤양 환자의 15∼20%에서 발생하는 것으로 알려져 있으며, 소화성 궤양 합병증 중에 가장 흔한 것으로 보고되고 있다. (6,7,8) 특히, 장 천공이나 위 유문부 폐쇄 등의 합병증 빈도가 감소하는 데 비해 오히려 위장관 출혈의 합병증은 증가하는 것으로 보고되고 있는데, 이는 고령의 환자에서 아스피린을 포함한 NSAID의 사용 증가로 인한 것으로 추정되고 있으며, (6,8) NSAID를 복용하는 고령의 환자군의 20%에서 무증상 궤양으로부터 출혈이 발생하는 것으로 조사되어 문진 및 진찰 시 주의가 요구된다. (6,9) 또한 NSAID 등 약제 사용의 증가는 위궤양 천공을 증가시키고 있는 것으로 조사되었는데, 개방성 천공과 그로 인한 세균성 복막염의 발생은 응급 수술의 적응증이기도 하다. (6)

따라서 NSAID를 복용 중인 환자, 특히 소화성 궤양 발생의 위험 인자-소화성 궤양의 과거력, 65세 이상의 고령, 고용량 NSAID 투여, 스테로이드 병용, 항응고제 병용, 저용량 아스피린을 포함한 NSAID의 중복 투여를 가진 환자의 경우, 소화성 궤양 예방을 위한 조치로서 NSAID와 위장관 보호 약물 (PPI, misoprostol 등)의 병용을 권고하고 있다. (10)

소화성 궤양 발생의 위험성이 매우 높은 환자군 (high risk group)의 경우, NSAID와 위장관 보호제를 병용 (예: 낙소졸 정) 하는 대신, 환자에 대한 위장관 보호 수준을 더욱 높여 대안을 모색하거나 COX-2 저해제와 위장관 보호제를 병용하도록 권고하고 있다. (10) 본 임상시험에서는 선정/제외 기준을 통해 high risk group 환자를 제외한 골관절염 환자들로 시험대상자군이 선정되도록 하였다.

## 임상시험 대상군에 대한 임상시험용 의약품 투약 근거

NSAID 투여와 관련된 dyspepsia 증상은 매우 흔하며, 약 25~50%의 약물 복용자에서 발생하는 것으로 보고되고 있다. (11,12,13,14)

그러나 erosive gastritis가 내시경에서 확인된 복용자의 40%는 dyspepsia 증상이 관찰되지 않았으며, dyspepsia 증상이 있는 복용자의 50%는 정상 위장 점막 소견이 관찰된 만큼 dyspepsia 증상이 위장 점막의 손상을 예측하지는 않는 것으로 알려져 있다. (11,13,14,15)

비선택적인 NSAID에 비해 COX-2 선택적 억제제를 사용할 경우 NSAID 투여와 관련된 dyspepsia 증상 발생이 감소하는 것으로 보고되고 있다. (11,16,17)

또한 비선택적 NSAID 투여로 인한 dyspepsia 증상은 PPI 등의 사용으로 감소시킬 수 있으며, ranitidine, misoprostol 등에 비해 PPI인 omeprazole이나 시험약인 esomeprazole dyspepsia 증상 개선에 더욱 효과적인 것으로 보고되고 있다. (13,14,18,19)

따라서 본 임상시험 참여 골관절염 환자들을 대상으로 본 임상시험용 의약품인 낙소졸 정이나 COX-2 저해제를 투여하는 것은 시험대상자에 대해 위장 기능 장애 치료 관련 유용성과 안전성이 충분히 고려된 임상시험이 될 것으로 판단하였다.

## 임상시험용 의약품의 유효성과 안전성 요약

본 임상시험용 의약품인 낙소졸 정을 대상으로 임상시험을 실시하였으며, 이상반응 발현 현황을 포함한 안전성에 대한 정보는 Section 5.3.4.1에 기재하였다.

본 임상시험용 의약품과 마찬가지로 Naproxen과 에스오메프라졸이 함께 포함된 복합제 임상시험에서도 두 성분의 병용 투여가 naproxene 단독 투여에 비해 위장관 출혈 발생을 감소시키며 상부 위장관 이상반응이나 십이지장 궤양으로 인한 약물 투여의 중단을 감소시키는 것으로 보고된 바 있다. (20)

또한 두 약제의 병용이 장기 안전성 측면에서도 양호함이, 12개월 간의 장기 투여 임상시험 결과에서 확인된 바 있다. (21)

## 임상시험용 의약품의 사용 경험

본 임상시험의 시험약인 낙소졸 정은 2013년 8월 품목 승인된 이래 본 임상시험의 적응증인 골관절염을 포함하여 근골격근계 질환 등에 사용되어 왔다.

## 투여에 따른 위험 편익 고찰

본 임상시험은 낙소졸 정의 NSAID 투여가 필요한 골관절염 환자 대상의 제 4상 임상시험이다.

본 임상시험용 의약품인 낙소졸 정는 NSAID 계열 의약품인 naproxen과 PPI 계열 의약품인 에소메프라졸이 포함되어 있는 서방형 제제이다.

골관절염 환자의 통증을 조절하기 위한 NSAID의 임상적 유용성은 기왕의 사실로서, NSAID 계열인 나프록센 성분은 골관절염 환자의 통증 개선을 위해서, PPI 계열인 에스오메프라졸 성분은 NSAID 투여에 따른 위장관 출혈이나 dyspepsia 증상 치료를 위해서 널리 사용되어 온 비교적 안전한 의약품들로 알려져 있다.

미국 소화기학회 (ACG)의 가이드라인에서도, 위장관 출혈에 대해 moderate risk를 가진 환자들의 경우, NSAID 투여 시 PPI 제제를 병용하도록 권고하고 있다. (10)

더욱이 본 임상시험을 실시함에 있어 임상시험용 의약품의 금기 사항들에 대해 선정/제외 기준에 이미 반영하였으므로, 기왕에 알려진 위험군은 본 임상시험 참여에서 배제될 것이다.

결론적으로 적절한 시험대상자군 선정과 이상반응 발생 여부에 대한 추적 평가가 선행된다면 골관절염 환자를 대상으로 시험약인 낙소졸 정을 12주 투여하는 것이 시험대상자의 위해 발생 가능성을 크게 증가시키지는 않을 것으로 판단하였고, 시험대상자의 이상반응 발생 여부와 활력징후 측정을 포함하여 면밀한 관찰이 병행된다면 용량 범위에서 12주 경구 투여의 안전성은 양호할 것으로 예상되었다.

## 임상시험 디자인 고찰

전향적 (Prospective), 무작위 배정 (Randomized), 이중 눈가림 (Double-blinded), 이중 위약 (Double-dummy), 활성 대조 (Active-controlled), 2개 치료군 평행 (Two-arm Parallel), 다기관 (Multi-center) 임상시험이다.

시험대상자 별 치료 및 관찰 기간은 12주이다.

각 투여군 별로 12주의 투여가 완료된 후 안전성과 유효성을 관찰할 것이다.

본 임상시험에서 설정된 투여 및 관찰 기간은 낙소졸의 안전성 및 유효성 변수를 평가하는데 적절할 것으로 판단된다. 이에 관하여 본 임상시험 계획서 Section 4. 2에서 논의하고 있다.

기타 임상시험 디자인의 근거 등에 대해서도 Section 4.2에서 언급하였다.

## 결론

결과적으로 본 임상시험을 바탕으로 낙소졸 정 투여가 위장 기능 장애 치료 및 예방에 미치는 효력 관련 정보와 더불어 기타 안전성 정보를 제공받을 수 있게 되기를 기대한다.

본 임상시험은 임상시험 계획서와 의약품 임상시험 관리기준 (GCP), 기타 국내 관련 법규를 준수하여 시행될 예정이다.

# 임상시험 목적

본 임상시험의 목적은 임상시험용 의약품 낙소졸 정을 만 50세 이상의 골관절염 환자에 12주 경구 투여 후 안전성과 유효성을 평가하는데 있다.

**일차 시험 목적**

- 골관절염 환자를 대상으로 낙소졸 투여 12주 후 LDQ (Leeds Dyspepsia Questionnaire)로 평가되는 위장 장애 보호 효과가 Celecoxib 투약군에 비해 비열등함을 입증한다.

**이차 시험 목적**

- 골관절염 환자를 대상으로 낙소졸 및 Celecoxib 투여 12주 후 GSRS (Gastrointestinal Symptom Rating Scale)로 평가되는 위장 장애 보호 효과를 비교한다.
- 골관절염 환자를 대상으로 낙소졸 및 Celecoxib 투여 12주 후 GI (Gatrointestinal) 부작용 발생률 및 GI 부작용으로 인한 약물 중단률을 비교한다.
- 골관절염 환자를 대상으로 낙소졸 및 Celecoxib 투여 12주 후 통증 VAS (Visual Analogue Scale)로 평가되는 통증 개선 효과를 비교한다.
- 골관절염 환자를 대상으로 낙소졸 및 Celecoxib 투여 12주 후 EQ-5D로 평가되는 삶의 질 개선 효과를 비교한다.
- 골관절염 환자를 대상으로 낙소졸 및 Celecoxib 투여 12주 후 환자 복용 순응도를 비교한다.
- 골관절염 환자를 대상으로 낙소졸 및 Celecoxib 투여 12주 후 전반적인 안전성을 비교한다.

# 임상시험계획

## 임상시험 전반적인 방법 및 계획

전향적 (Prospective), 무작위 배정 (Randomized), 이중 눈가림 (Double-blinded), 이중 위약 (Double-dummy), 활성 대조 (Active-controlled), 2개 치료군 평행 (Two-arm Parallel), 다기관 (Multi-center) 디자인에 의하여 진행되는 4상 임상시험으로, 스크리닝 시, 본 임상시험에 적합한 것으로 평가된 시험대상자는 day 1에 시험군과 대조군에 무작위 배정되어 시험약인 일일 2정의 낙소졸 정이나 대조약인 쎄레브렉스 1캡슐을 12주간 매일 경구 투여받는 임상시험이다.

**임상시험용 의약품 및 용법 용량**

임상시험용 의약품 및 용법 용량에 대해서는 Section 5.1.1를 참조하기 바란다. 기타 임상시험용 의약품의 병용금지 의약품, 주의 사항, 의약품의 포장 및 교부 등 자세한 내용에 대해서는 Section 5.3및 Section 5.4를 참조하기 바란다.

**일차 평가 변수**

골관절염 환자를 대상으로 낙소졸 투여 12주 후 LDQ (Leeds Dyspepsia Questionnaire)로 평가되는 위장 장애 보호 효과가 Celecoxib 투약군에 비해 비열등함을 입증한다.

**이차 평가 변수**

이차 평가 변수에 대해서는 Section 7.3.2와 Section 7.3.3에서 기술하였다. 이차 평가 변수는 위장 장애 보호 효과, 통증 개선 효과, 삶의 질 개선 효과, 복용 순응도, 안전성 평가 등을 위한 변수들로 구성되어 있다.

**시험대상자**

본 임상시험 참여에 동의하고 시험대상자 선정 기준에 합당하고 제외 기준에 해당되지 않는 골관절염 환자를 대상으로 한다. 시험자는 시험대상자 등록 시 합당 여부를 신중히 검토하고, 본 연구의 목적 및 시험대상자의 권리 및 건강 상태에 대해 충분히 고려하여야 한다. 자발적으로 동의서에 서명할 수 없는 시험대상자는 본 연구의 대상자로 선정되지 않는다. 특히, 취약한 환경에 있는 시험대상자 (vulnerable volunteers)는 시험대상자 선정 시 세심한 주의를 기울여야 하여 참여 이유에 대한 타당성이 입증되어야 한다.

시험대상자의 선정 기준에 대해서는 Section 4.3에서 기술하였다.

**임상시험 절차 및 평가**

본 임상시험은 국내 10개 (변경 가능) 임상시험 기관으로부터 106명 이상의 자원자를 대상으로 서면 동의서 확보 후 스크리닝 방문과 이후의 일정을 시행한다.

시험대상자 시험군 배정 (Section 4.4.2), 시험대상자의 탈락 (Section 4.5), 시험대상자의 시험 종료 (Section 4.9) 등은 각각 해당 항목에서 논의하였다.

본 임상시험과 관련된 기타 임상시험 절차와, 관찰 항목 및 평가 항목에 대해서는 Section 6 임상시험 절차 및 평가를 참고하기 바란다.

모든 임상검사실 검사 등을 포함한 모든 관찰 항목들은 각기 정해진 일정에 따라 임상시험 실시기관에서 시행될 것이다.

임상시험 참여 종료 방문 (end of study)은 모든 시험대상자를 대상으로 시험기관에 실제 방문을 실시하여야 한다.

중도 탈락되는 시험대상자의 경우 모든 가능한 시험대상자들을 대상으로 방문3에 상응하는 검사 및 평가를 실시한다. 탈락한 시험대상자는 대체될 수 없다 (Section 4.6 참조).

이상반응이 지속될 경우 추가 방문이 실시될 수 있다 (Section 6.1 참조).

**눈가림 수준과 방식**

- 이중 눈가림
- 본 임상시험은 이중 눈가림 임상시험으로 시험자와 시험대상자는 배정받은 투여군에 대해서는 눈가림되므로, 시험약 또는 대조약을 투여 받을 지에 대해서는 눈가림될 것이다. 눈가림법 및 응급 눈가림 해제에 대해서는 Section 5.8눈가림법 항목과 Section 5.9 응급 눈가림 해제 항목을 참조바란다.

사용되는 시험약 (낙소졸 정)과 대조약 (쎄레브렉스 캡슐)은 각각에 대한 위약을 함께 공급하되, 시험자나 시험대상자가 각각의 약물과 해당 위약 간의 차이를 식별하지 못하도록 동일한 성상, 포장을 시행하여 시험자와 시험대상자에 대해 투약된 약물에 대한 눈가림을 유지하도록 한다.

**치료 관련 디자인**

- 다회 투여, 평행 치료군
- 본 임상시험은 다회 투여, 평행 치료군 임상시험이다. 본 임상시험의 시험대상자로 선정되기 위해서는 통증 VAS 평가 상 40mm 이상이어야 한다. 또한 NSAID 복용으로 인해 발생한 dyspepsia 증상에 대한 LDQ 척도 점수가 12주 투여 후 감소할 것으로 판단되며, 교차 설계를 적용할 경우 첫 치료 적용 후 두 번째 치료 적용 전 시험대상자의 기저치가 다시 선정 기준에 해당하는 수준으로 복귀될 수 있는 지 여부가 확실하지 않다. 또한 이차 평가 변수인 NSAID 사용으로 인한 위장관 이상반응의 경우 교차 설계 시 설정되는 washout 기간 중 개선되기 어려울 것으로 판단되었다. 따라서 교차 설계보다 평행 임상시험이 더 적절할 것으로 판단하였다.

**대조군**

- 활성 대조군
- 본 임상시험에서는 쎄레브렉스 캡슐 투여군을 대조군으로 사용할 예정이다.

**시험대상자 배정**

시험군 배정에 대해서는 Section 4.4.2에서 기술하였으며, 무작위 배정 과정에 대해서는 Section 7.2에 기술하였다. 배정 후에는 각 시험대상자에게 고유의 시험대상자 번호가 부여되어 남은 연구 참여 기간 중 시험대상자 식별을 위해 사용된다. 시험대상자 번호는 임상시험 중 재사용될 수 없다.

배정 과정 중 층화 모집은 시행하지 않는다.

시험대상자는 총 2개 투여군에 시험군 53명, 대조군 53명이 무작위 배정될 예정이다.

**예정된 임상시험 기간**

- 예정된 시험대상자 모집기간은 약 21개월 예정이다.
- 예정된 전체 임상시험 기간은 임상시험 개시일로부터 약 12주이다.
- 각각의 시험대상자에 대한 예정된 치료 및 평가 기간은 1일 (투약 당일)의 스크리닝 및 washout 기간과 약 12주의 투약 및 추적 관찰 기간으로 구성되어 있다.
- 각 시험대상자는 1번의 스크리닝 방문과 무작위 배정 및 임상시험용 의약품 교부가 이루어지는 1번의 방문 및 1번으로 이루어진 추적 방문을 시행한다.

임상시험의 전반적인 구성은 다음과 같다 (그림 1).

그림 1 임상시험의 전반적인 구성

| **V1**  **스크리닝/ Washout**  **(D-14~1)** | | | **V2**  **무작위배정/시험약 교부**  **(D1)** | | | | | | | |  | | | |  |  |  | | |  | | **V3**  **(시험종료)**  **(D84±7)** | | | | | | |  |
| --- | --- | --- | --- | --- | --- | --- | --- | --- | --- | --- | --- | --- | --- | --- | --- | --- | --- | --- | --- | --- | --- | --- | --- | --- | --- | --- | --- | --- | --- |
|  |  |  | |  |  | |  | | |  | |  |  |  | | | |  |  | |  | |  |  | | |  |  | |
|  | **스크리닝/ Washout** | | | **투약 및 추적 관찰 방문 기간 (12주)** | | | | | | | | | | | | | | | | | | | | | | | |  |  |
|  |  | | |  |  |  | |  |  | | | | | | | | | | | | |  | | |  |  | |  | |

## 임상시험 디자인에 대한 검토 논의

전향적 (Prospective), 무작위 배정 (Randomized), 이중 눈가림 (Double-blinded), 이중 위약 (Double-dummy), 활성 대조 (Active-controlled), 2개 치료군 평행 (Two-arm Parallel), 다기관 (Multi-center) 임상시험으로서, 골관절염 환자 대상의 제 4상 임상시험이다.

### 임상시험 디자인에 대한 검토

본 임상시험은 전향적 (Prospective), 무작위 배정 (Randomized), 이중 눈가림 (Double-blinded), 이중 위약 (Double-dummy), 활성 대조 (Active-controlled), 2개 치료군 평행 (Two-arm Parallel), 다기관 (Multi-center) 임상시험으로, 시험약인 낙소졸 정을 12주 투여 후 LDQ를 이용 dyspepsia 증상의 변화 정도를 동일 기간 투여된 대조약인 쎄레브렉스 캡슐 대비 비열등성을 평가하는 것이 목적이다. 본 임상시험에서 dyspepsia 증상을 포함하여 위장관 증상의 발현, 유효성 및 이상반응 발생 현황 평가 시 시험자나 시험대상자로부터 기인하는 편견 (bias)을 줄이고자 이중 눈가림, 무작위 배정 디자인을 적용하였다.

### 치료 기간에 대한 검토

시험대상자 별 전체 치료 기간은 12주~14주로서, 스크리닝 기간 (14일 이내), 12주의 치료 기간 및 평가 기간으로 구성된다.

12주의 치료 기간은 일차 평가 변수인 LDQ 척도 점수 변화를 관찰하기에 충분한 기간으로 판단된다.

Functional dyspepsia 환자를 대상으로 위약과 Itopride 투여 간의 dyspepsia 증상 개선 정도를 비교한 선행 연구에서, 이들 약물을 8주간 투여 후 LDQ 척도 점수 변화를 관찰하였다. (22)

또한 LDQ 대신 SODA (severity of dyspepsia assessment)를 이용하여 NSAIDs를 투여받은 류마티즘 관절염 또는 골관절염 환자들에서 dyspepsia를 평가한 임상시험의 경우 12주간의 투여 후 dyspepsia 증상에 대해 평가한 바 있다. (23)

결론적으로 본 임상시험에서 계획된 12주간 투여는 시험약 투여 효과를 보는데 부족하지 않을 것으로 판단되었다.

### 용량 선정 및 선정 근거

**선정 용량:** 12주의 투여 후 LDQ 척도 상 점수 변화를 관찰하는 용량으로서, 낙소졸 정 2정/일 또는 대조약 1캡슐/일을 매일 경구 투여할 것이다.

본 임상시험에서 사용될 용량은 시험약 1일 2회 1정씩, 2정/일, 대조약 1일 1회 1캡슐씩, 1캡슐/일로서, 시험약과 대조약 모두 골관절염에 대해 국내 승인되어 있는 용법 용량이다.

### 대조군 선정에 대한 검토

본 임상시험은 활성 대조 임상시험이다. 대조약으로는 쎄레브렉스 캡슐이 사용될 것이다.

쎄레브렉스 캡슐은 선택적COX-2 저해제이며, 비선택적인 NSAID에 비해 선택적 COX-2 억제제를 사용할 경우 NSAID 투여와 관련된 dyspepsia 증상 발생이 감소하는 것으로 보고되고 있다. (11,16,17)

따라서 쎄레브렉스 캡슐은 NSAID 투여와 관련된 dyspepsia 증상 관련 연구에 적절한 대조약으로 판단되었다.

### 시험군 배정에 대한 검토

본 임상시험은 무작위 배정 임상시험이다. 안전성과 유효성 평가 시 시험자나 시험대상자로부터 기인하는 편견 (bias)을 줄이고자 이중 눈가림, 무작위 배정 디자인을 적용하였다.

또한 본 임상시험은 무작위 배정 방식으로 진행하여 기저값이 연구 결과에 미치는 영향을 최소화할 수 있을 것으로 판단된다. (24)

또한 시험군 배정 과정 중 층화를 실시하지는 않을 것이다.

### 특수 인구 집단 시험대상자의 포함

본 임상시험에는 노인은 포함될 수 있으나, 소아, 임산부, 수유부 등의 기타 특수 인구 집단 시험대상자는 포함되지 않을 것이다.

고연령은 NSAID 투여로 인한 위장관의 궤양 및 출혈 위험 인자 중 하나이다. 본 임상시험에서는 선정/제외 기준과 임상시험 중 병용금기 의약품 설정 등을 통해 기타 위험 인자 (합병증을 동반 또는 동반하지 않는 소화성 궤양 병력자, 저용량 아스피린을 제외한 타 NSAID 병용 투여의 금지, 항응혈제 및 스테로이드 제제 사용의 금지)를 지닌 노인 시험대상자의 참여를 제한할 것이다.

## 임상시험 대상군의 선정

본 임상시험은 만 50세 이상의 통증VAS 평가 상 40mm 이상인 골관절염 환자를 대상으로 실시할 예정이다.

### 선정 기준

1. 본 임상시험에 대하여 자세한 설명을 듣고 이해한 후, 자의로 참여를 결정하고, 서면 동의한 한국인
2. 동의서 작성 당시 연령이 만 50세 이상의 남녀
3. 임상시험 동안 추적이 가능하고, EQ-5D, VAS 설문지 등을 읽고 작성할 수 있는 등, 임상시험에 요구되는 사항을 준수할 수 있는 자
4. 병력을 통해 신체의 증상이 있는 골관절염이 확인된 환자로서, 방문 2에서 실시한 통증 VAS 검사 상 40mm 이상
5. 스크리닝 결과, 본 연구에 적합하다고 판단된 자 (단, 임상검사실 검사 결과가 참고 범위를 벗어나도 시험자의 판단에 따라 참여할 수 있음)

### 제외 기준

1. 현재 임상시험에 참여 중이거나 이전 임상시험 참여 후 30일이 경과하지 않은 자 (참여는 임상시험용 의약품의 최종 투약일을 기준으로 함)
2. 스크리닝 전 6개월 기준으로 알코올 및 기타 약물 남용 기왕력자
3. 일주일 기준 21잔 이상 (일반적으로 소주잔: 55mL; 맥주잔: 200mL; 양주잔: 30mL로 동일하게 취급)의 술을 마시는 경우
4. 최근 5년 이내 합병증 (출혈 [Bleeding], 장천공 [Perforation], 관통을 통한 장누공 [Penetration], 위 유문부 폐쇄 [Gastric Outlet Obstruction])을 동반한 소화성 궤양 병력 환자
5. 스크리닝 방문 전 6개월 이내 합병증을 동반하지 않은 소화성 궤양 환자 또는 현 병력 상 활동성 소화성 궤양 환자
6. 기왕에 알려진 헬리코박터 감염 환자로서 제균 치료를 시행 받지 않은 자
7. 기왕에 알려진 위식도 역류증 환자
8. 안전성 및 유효성 평가에 영향을 줄 수 있는 다음의 관절 질환이 병력 상 확진되었거나 임상 소견 상 의심되는 환자: 패혈성 관절염, 류마티스 관절염과 같은 염증성 관절 질환, 통풍, 재발성 가성 통증, 파제트병, 관절 골절, 조직흑변증, 말단거대증, 혈색소증, 윌슨병, 원발성 골연골증, 과운동 증후군 (Ehlers Danlos Syndrome) 및 기타 콜라겐 유전자 이상 질환 등
9. 임상시험 기간 동안 수술이 예정되어 있는 환자
10. 소화기관의 암 병력을 가진 환자
11. 약물 흡수 장애로 인한 GI disorder 환자
12. 위장관 출혈, 뇌혈관 출혈, 기타 출혈 질환 환자, 심한 혈액 이상 환자
13. 중등증 또는 중증 간장애 환자 (Child Pough Class II 이상)
14. 중증 심부전 환자 및 관상동맥 우회로술 (CABG) 병력 환자
15. 중증 신장애 환자 (CrCl <30mL/min)
16. 치료에도 불구하고 조절되지 않는 심한 고혈압 환자 (SBP ≥160mmHg 또는 DBP ≥100mmHg )
17. 본 임상시험용 의약품을 구성하고 있는 성분에 대해 알레르기의 기왕력이 있는 경우
18. 기타 NSAIDs나 PPI에 과민증, 알러지가 있는 환자
19. 임상시험 참여 전 금기 의약품 또는 치료를 받고 정해진 기간이 경과하지 않은 시험대상자 (Section 5.3.2 참조)
20. 병용금기 의약품을 투여 중인 환자 (Section 5.3.3 참조)
21. 최근 1년 이내의 골관절염의 해당 관절 수술이력을 가진 환자
22. 임부, 수유부 또는 적절한 피임법 (임상시험용 의약품 투여 21일 전부터 콘돔, 격막, 자궁내 피임 장치, 호르몬 피임 제제 복용 등을 사용하거나 남성 파트너가 정관 절제술을 받은 경우)을 실시하지 않는 가임 여성 (폐경기 여성은 피임법 적용 해당 안됨)
23. 기타 임상적으로 유의한 의학적 또는 정신 의학적 소견으로 시험자가 부적합하다고 판단한 자

## 시험대상자 배정/ 임상시험용 의약품 투약

임상시험 참여 이전 서면 동의서 획득을 포함한 선정/제외 기준에 충족된 시험대상자들만이 각 투여군 별로 무작위 배정되어 임상시험용 의약품을 투여 받을 것이다 (Section 7.2참조).

### 스크리닝 번호 및 시험대상자 번호 부여

임상시험에 참여하고자 서면 동의한 자원자를 대상으로, 스크리닝 시 시험기관을 방문한 순서대로 스크리닝 번호 (S+기관이니셜+XXX)를 부여한다. 이 때 스크리닝 번호는 S+기관이니셜+001부터 시작하며 일련번호 형태로 부여한다. 한 번 부여한 스크리닝 번호는 다른 자원자에게 부여할 수 없으며, 모든 자원자는 오직 하나의 스크리닝 번호를 받을 수 있다. 본 임상시험에 참여하는 시험대상자 중 스크리닝 검사를 통과한 시험대상자를 대상으로 day 1에 무작위 배정을 시행하여 시험대상자 번호 (R+ 기관이니셜 +XXX)를 부여한다. 시험대상자 번호는 각 기관별로 RX001부터 시작하며 일정한 규칙을 갖는다. 예를 들어, ‘RB006’에서 ‘R’은 무작위 배정 (Randomized)을, ‘B’는 기관이니셜을, 이후의 세 자리 숫자 ‘006’은 B 기관에서 본 임상시험에 6번째로 무작위 배정된 시험대상자를 의미한다. 각 시험대상자에게 부여된 시험대상자 번호는 임상시험이 끝날 때까지 시험대상자를 인식하는 시험대상자 식별 코드 (subject identification code)로 사용되며, 한 번 부여된 시험대상자 번호는 다른 시험대상자에게 부여할 수 없으며, 모든 시험대상자는 오직 하나의 시험대상자 번호를 받을 수 있다.

### 시험군 배정

스크리닝 방문 시, 서면 동의를 획득하고 스크리닝 검사에 통과한 이후 임상시험 관련 처치가 시작되기 이전에 각 시험대상자 별로 고유의 시험대상자 번호가 부여될 것이다. 선정 기준에 해당되는 시험대상자들은 방문 2에서 치료에 배정될 예정이다. 부여된 시험대상자 번호는 증례기록서에 기록될 예정이다. 배정된 시험대상자는 임상시험의 나머지 기간 동안 만 나이, 시험대상자 번호를 이용해 식별될 것이다.

배정 코드 생성은 의뢰자 또는 임상시험 위탁기관이나 이들로부터 컴퓨터를 이용한 코드 생성을 위임 받은 자에 의해 시행될 예정이다. 배정을 통해 시험군이나 대조군에 배정된 시험대상자들은 임상시험용 의약품을 투여 받게 된다.

배정 후에는 각 시험대상자에게 고유의 시험대상자 번호가 부여되어 남은 연구 참여 기간 중 시험대상자 식별을 위해 사용된다. 시험대상자 번호는 임상시험 중 재사용될 수 없다.

본 임상시험은 평행군 임상시험이다. 본 임상시험의 시험군과 대조군에 배정될 시험대상자 수는 다음과 같다.

표 1 시험군 및 군 별 시험대상자 수

|  | 용량 | 시험대상자 수 | 투여 약제 |
| --- | --- | --- | --- |
| 시험군 | 1회 1정; 2정/일 | 53명 | 낙소졸 정 |
| 대조군 | 1회 1캡슐; 1캡슐/일 | 53명 | 쎄레브렉스 캡슐 |

## 시험대상자 탈락 기준

시험대상자의 개인적 상황, 의학적 또는 행정적인 사유에 의하여 임상시험으로부터 시험대상자의 탈락, 임상시험약 투여 중지, 임상시험의 조기 종료/임상시험 기관 폐쇄가 시행될 수 있으며 그 원인은 아래와 같다.

### 임상시험 탈락

시험대상자가 원할 시에는 언제든 임상시험의 참여를 중단할 수 있으며, 그 이유를 밝힐 의무가 없다.

아래와 같은 사유가 발생하면 임상시험에서 탈락되어야 한다.

- 시험대상자 본인 또는 법적 대리인이 동의 철회하는 경우
- 임상시험에의 지속적인 참여가 시험대상자에게 해가 된다고 시험자가 판단하는 경우
- 임상시험을 지속하기에는 너무 심각한 이상 반응 (CTCAE기준 Grade4 또는5에 해당하거나, 시험자가 판단했을 때, 시험대상자의 연구 참여에 어려움이 있다고 예상되는 경우)
- 중대한 임상시험 계획서 위반이라고 의뢰자 또는 시험자가 판단하는 경우
- 기타 의뢰자 또는 시험자의 판단에 의해 임상시험 진행이 적합하지 못하다고 판단되는 경우
- 시험대상자가 기타 이유 등으로 중단하기를 원하는 경우

중대한 임상시험 계획서 위반의 경우, 해당 시험대상자를 분석에서 탈락 처리 (PP제외)함을 원칙으로 하며 해당 사항은 다음과 같다.

- 시험약의 유효성 또는 안전성 평가를 심하게 왜곡시킬 수 있거나, 임상적인 판단 상 시험대상자의 안전에 영향을 줄 수 있는 선정/제외 기준 위반에 해당되어 임상시험 참여 중지가 필요하다고 시험자 또는 의뢰자가 판단하는 경우
- 시험대상자가 임상시험 기간 동안 임상시험 대상 질환에 대한 약물이나 외과적인 다른 치료법을 계속 사용하는 것으로 시험대상자가 알려오고 이 치료법들을 중단할 의사가 없다고 시험자가 판단하는 경우
- Section 5.3.3에서 임상시험용 의약품 투여를 중지하도록 규정하고 있는 병용금지 의약품을 복용 또는 투여한 경우
- 무작위배정 전 사전에 규정된 약물에 대한 Washout을 실시하지 않은 시험대상자
- 무작위 배정 오류인 경우
- 복용 순응도가 80% 미만인 경우

시험자는 탈락된 시험대상자에 대해 추적 조사를 할 수 없는 경우, 시험대상자의 거주지와 건강 상태를 확인하기 위한 노력을 해야 한다.

만약 어떤 시험대상자가 임상시험에서 탈락된다면 탈락 사유와 상황에 대하여 가능한 완벽하게 완료하여 기록해 두기 위한 모든 노력을 기울여야 한다.

시험대상자가 조기 중단을 하는 경우, 방문 3에 해당하는 평가 및 처치가 시행되어야 하며, 임상시험용 의약품에 대한 이상반응 여부를 포함하여 조기 중단의 원인을 찾기 위한 적절한 평가를 가능한 한 실시하여야 한다 (Section 6.1 참조). 모든 경우에 대하여 그 탈락 사유를 증례기록서에 기록해야 한다.

### 임상시험용 의약품 투약의 중단

아래와 같은 사유가 발생하게 되면 임상시험 도중에 임상시험용 의약품 투여를 중단하여야 한다.

- 임상적인 판단 상 시험대상자의 안전에 영향을 줄 수 있는 제외 기준에 해당되고 임상시험용 의약품 투여 중지가 필요하다고 시험자 또는 의뢰자가 판단하는 경우
- 임상시험용 의약품 투여를 일시적 또는 지속적으로 중지하는 것이 필요하다고, 시험자 또는 시험대상자가 판단하는 경우의 이상반응 발생
- Section 5.3.3에서 임상시험용 의약품 투여를 중지하기로 규정되어 있는 병용금지 의약품을 복용 또는 투여한 경우
- 임상시험용 의약품에 대한 심각한 불순응

시험자는 시험대상자가 계획에 따라 투여를 하도록 최선을 다해야 한다. 시험대상자가 임상시험용 의약품의 투여를 중단하는 경우, 임상시험 참여 중단과 마찬가지로, 가능한 한 모든 필요한 항목들에 대해 관찰 하고 이를 기록을 하여야 한다.

모든 경우에 있어서 임상시험용 의약품 투여를 중단하게된 사유와 상황 및 기간에 대해 증례기록서에 기재하여야 한다.

## 시험대상자의 대체

본 임상시험에서는 중도 탈락한 시험대상자를 대체하지 않는다.

## 새로운 용량군 추가

본 임상시험에서는 새로운 용량군을 추가하지 않을 것이다.

## 임상시험의 조기 종료

아래와 같은 사유에 의해 임상시험이 조기에 종료될 수 있다.

시험대상자에 대한 이점/위험성 비율에 관하여 부정적인 방향으로 임상시험용 의약품과 관련된 새로운 정보가 획득된 경우로 다음과 같은 사유들이 해당한다.

- 효과없다는 증거 획득
- 새롭고 중요한 이상약물반응 발생 또는 기왕의 이상반응 발생율과 심각성이 예상하지 못한 정도로 과도한 경우
- 기타 안전성 상의 이유
- 본 임상시험을 지속하는 것이 의학적 윤리적 관점에서 정당하지 않다고 의뢰자가 판단하는 경우
- 해당 임상시험 실시기관에서의 시험대상자 등록 속도가 본 임상시험을 위하여 필요한 시험대상자 수의 등록에 차질을 줄 것으로 판단되는 경우
- 임상시험용 의약품의 판매 또는 공급이 중단된 경우

본 임상시험이 조기에 중단될 경우 관련 규정에 의거하여 정부 규제 기관이나 임상시험 심사위원회에 통보될 것이다.

정부 규제 기관의 요청이 있을 경우 임상시험은 중단되거나 정지될 수 있다.

## 임상시험의 종료

본 임상시험에서 방문 3을 “시험 종료 방문 end of study (trial) visit” 으로 규정하며 방문 3을 완료된 시험대상자들을 완료된 시험대상자로 규정한다.

아래의 경우가 발생하게 되면 본 임상시험은 종료되지 않은 것으로 판단한다.

- 임상시험용 의약품을 투여 예정인 시험대상자가 남아 있는 경우
- 시험 기관에서 임상시험에서 규정된 방문 일정이 완료되지 않은 경우

단, 합리적 수준의 방문 요청을 하였음에도 불구하고 방문을 시행하지 않거나 연락이 되지 않는 경우 (lost to follow up)에는 해당 시험대상자의 임상 참여가 종료된 것으로 판단할 것이다.

임상시험 종료 후에는 추가적인 평가가 시행되지 않을 것이다.

## 기타 고려 사항

임상검사실 검사에 영향을 주거나 임상시험용 의약품과의 상호작용이 발생 할 수 있는 외인적 요인 (음주, 심한 운동 등)을 절제한다.

**식사**

임상검사실 검사가 예정된 날짜의 하루 전 밤 10시 이후 금식해야 한다.

**음주**

임상시험 기간 도중 주당 평균 21잔 (하루 3잔) 이상의 음주는 절제한다. 또한, 임상검사실 검사 전 72시간 동안은 음주를 피한다.

**운동**

스크리닝 검사를 포함하여 임상검사실 검사 전일에는 심한 운동은 금한다.

# 임상시험용 의약품 및 임상시험에 사용되는 기타 의약품

“임상시험용 의약품 Investigational Medicinal Product”은 시험약과 대조약을 포함한다.

## 임상시험에 사용되는 의약품

### 시험약

### 제품명/코드명

제품명/코드명: 낙소졸 정

### 성상

미황색 장방형의 필름코팅정제

### 성분 및 함량

나프록센 (USP) 500mg과 에스오메프라졸스트론튬사수화물 24.60mg (에스오메프라졸로서 20mg)

### 보관 조건

기밀용기, 실온(1~30℃) 보관

사용(유효) 기간: 제조일로부터 24개월

### 시험약의 위약

주성분을 함유하지 않은 시험약과 성상이 동일한 (미황색 장방형의 필름코팅정제) 제제

### 대조약

### 제품명/코드명

코드명/제품명: 쎄레브렉스 캡슐

### 성상

흰색내지 미황색의 분말이 충진된 상하 황색줄이 있는 흰색의 캡슐제

### 성분 및 함량

1캡슐 중 Celecoxib 200mg

### 보관조건

밀폐 용기, 실온 (15~30°C) 보관

### 대조약의 위약

주성분을 함유하지 않은 대조약과 성상이 동일한 (흰색내지 미황색의 분말이 충진된 상하 황색줄이 있는 흰색의 캡슐제) 제제

## 예측 용법 용량

### 시험약의 예측 용법 용량

*본 임상시험 도중 시험약 1정과 대조약의 위약 1캡슐을 식전 30분~60분에 동시에 복용

성인: 1일 2회, 1회 1정 (나프록센 500mg/에스오메프라졸 20mg)씩 경구 투여한다.

이 약은 쪼개거나 씹거나 부수지 말고 물과 함께 그대로 삼켜서 복용한다. 식전 30분~60분 전에 복용하는 것을 권장한다.

- 임상검사치에 대한 영향

1. 혈소판의 응집을 억제하고 출혈 시간을 연장시킬 수 있으므로 출혈 시간을 측정할 때는 주의한다.
2. 17-케토제닉 스테로이드의 측정에 영향을 주므로 측정 72 시간 전에 이 약의 투여를 중지한다.
3. 나프록센은 5-히드록시 인돌초산 (5HIAA)의 뇨 중 분석에 영향을 미칠 수 있다.

### 대조약의 예측 용법 용량

*본 임상시험 도중 대조약 1캡슐과 시험약의 위약 1정을 식전 30분~60분에 동시에 복용

이 약의 최소 권장량은 환자에 따라 조절되어야 하며, 식사와 관계없이 투여할 수 있다.

1. 골관절염 (퇴행관절염): 세레콕시브로서 200 mg을 1일 1회 복용한다.
2. 류마티스관절염: 세레콕시브로서 200 mg을 1일 1회 복용한다.
3. 강직척추염 (AS): 세레콕시브로서 200 mg으로 1일 1회 투여한다. 6 주 후에 효과가 관찰되지 않으면, 1일 400 mg까지 투여할 수도 있다. 1일 400 mg 투여한지 6 주 후에 효과가 관찰되지 않으면, 다른 치료 방법을 고려해야 한다.
4. 급성 통증 및 원발월경통: 초기 권장 투여량은 세레콕시브로서 400 mg이며 필요시 투여 첫날에 200 mg을 추가로 투여한다. 투여 둘째 날부터는 필요시 권장량으로서 1회 200 mg씩 1일 1회 투여한다.
5. 간장애 환자: 중등도의 간장애 (Child-Pugh Class II) 환자에 대해서는 용량을 1일 권장량의 약 50%로 감소시켜야 한다.

- 임상검사치에 대한 영향

1. 비스테로이드성 소염 진통제를 장기간 투여하는 환자는 정기적으로 전혈구 검사 (CBC) 및 이화학적 검사를 실시해야 한다. 비정상적인 간기능 검사 또는 신기능 검사 결과가 지속되거나 악화되면, 이 약의 투여를 중단해야 한다.
2. 다른 비스테로이드성 소염 진통제와 마찬가지로, 임상시험에서 BUN 상승이 위약보다 더 빈번하게 관찰되었으나, 임상적인 유의성은 확인되지 않았다.

## 임상시험에 사용되는 기타 의약품

### 병용 약물 및 치료

### 보조약물

임상시험용 의약품으로 위장 장애 (dyspepsia) 가 심할 경우 시험대상자가 사용할 수 있도록 허용된 보조 약물 (supplementary medication)은 다음과 같다.

- Almagate (한미 알마게이트 500mg정) 최대 6정/일, 30~50알/1인

상기 투여 허용된 의약품의 경우, 사용 용량, 투여 기간 등을 증례기록서 상에 기록해야 한다.

- 보조약물의 투여 기준

전체 임상시험 기간 동안 위장 장애 (dyspepsia) 증상의 강도가 허용 불가능한 수준이 된 시험대상자들에게는 Almagate (한미 알마게이트 500mg 정)를 24시간 당 최대 6정까지 추가 위장 장애 보호 요법을 시작하도록 허용한다. 이를 위해, 무작위배정이 끝난 후 시험대상자에게 적절한 분량의 구제약물을 공급하여야 한다. 시험대상자에게 Almagate (한미 알마게이트 500mg 정)를 24시간 당 최대 6정을 초과하여 투여하지 않도록 지도해야 한다. 보조약물 사용량은 시험대상자 일지에 기재 되어야 하고, 종료 시점에 반납 받아 사용량이 확인되어야 한다. 이상의 기준을 시험자는 시험대상자에게 교육하여야 한다.

### 구제약물

임상시험용 의약품으로 통증 조절이 어려울 경우 본 시험대상자 사용할 수 있도록 허용된 진통제 (rescue medication)는 다음과 같다.

- Acetaminophen (한미 써스펜ER 650mg 정) 최대 3g/일, 30~50알/1인

상기 투여 허용된 의약품의 경우, 사용 용량, 투여 기간 등을 증례기록서 상에 기록해야 한다.

- 구제약물의 투여 기준

전체 임상시험 기간 동안 통증의 강도가 허용 불가능한 수준이 된 시험대상자들에게는 아세트아미노펜 650mg으로 추가 진통제 요법을 시작하도록 허용한다. 이를 위해, 무작위배정이 끝난 후 시 시험대상자에게 적절한 분량의 구제약물을 공급하여야 한다. 시험대상자에게 아세트아미노펜 650mg을 1일 3g을 초과하여 투여하지 않도록 지도해야 한다. 구제약물 사용량은 시험대상자 일지에 기재 되어야 하고, 종료 시점에 반납 받아 사용량이 확인되어야 한다. 이상의 기준을 시험자는 시험대상자에게 교육하여야 한다.

### 임상시험 참여 전 금기 의약품 또는 치료

다음과 같은 의약품을 투여 받고 아래 기술한 기간이 경과하지 않은 시험대상자는 본 임상시험에 참여할 수 없다.

1. 임상시험용 의약품 투여 전 7일 이내의 NSAIDs (LDA [low dose aspirin, ≤325mg/day]는 해당 없음)
2. 임상시험용 의약품 투여 전 7일 이내의 gastroprotective 제제 (H2 blocker, PPI, misoprostol 등 기타 위장 보호 약물)
3. 임상시험용 의약품 투여 전 7일 이내의 정맥 또는 경구 투여 corticosteroid (prednisone 기준 ≤7.5mg/day 용량인 경구용 corticosteroid 제제, 피부용 연고제, 안점안액, 흡입 [inhalated], 비강내 [nasal]는 해당 없음)
4. 임상시험용 의약품 투어 전 8주 이내에 투여된 관절내 또는 근육내 corticosteroid 제제 또는 hyaluronic acid
5. 임상시험용 의약품 투여 전 7일 이내의 glucosamine and/or chondroitin sulfate
6. 임상시험용 의약품 투여 전 7일 이내의 lithium
7. 임상시험용 의약품 투여 전 7일 이내의 항레트로바이러스 제제 (atazanavir, atazanavir sulfate)

### 임상시험 중 사용이 금지된 의약품 및 치료

시험기간 중 시험약 이외의 약물은 투여하지 않는 것을 원칙으로 한다.

특히 다음과 같은 의약품은 투여되어서는 안된다.

1. 임상시험용 의약품을 제외한 NSAIDs (LDA (low dose aspirin, ≤325mg/day)는 해당 없음)
2. 타 gastroprotective drug (H2 blocker, PPI, misoprostol 등 기타 위장 보호 약물)
3. 정맥 또는 경구 투여 corticosteroid (prednisone 기준 ≤7.5mg/day 용량인 경구용 corticosteroid 제제, 피부용 연고제, 안점안액, 흡입 [inhalated], 비강내 [nasal]는 해당 없음)
4. 관절내 또는 근육내 corticosteroid 제제 또는 hyaluronic acid
5. glucosamine and/or chondroitin sulfate
6. lithium
7. 항레트로바이러스 제제 (atazanavir, atazanavir sulfate)

시험자가 의학적 견지에 따라 의약품 투여가 필요하다고 판단할 경우 모든 의약품은 투약될 수 있다. 그러나 시험자의 판단 없이 투약한 약물이 본 임상시험의 안전성 및 유효성 평가에 영향을 줄 수 있다고 예상되는 경우 이 시험대상자는 PP 군 분석에서 제외된다.

투여한 모든 약물명, 용량, 투여 경로 및 투여 사유를 반드시 증례기록서에 기재하고 시험자가 서명한다.

### 시험약의 사용 상 주의 사항

### 시험약의 이상 반응 Adverse Events

1. 본 약의 투여군 (N=1157)과 이 약의 주성분인 나프록센과 에스오메프라졸 각각의 안전성 자료를 비교한 결과, 새롭게 추가된 안전성 정보는 없었다.
2. 임상시험에서 이 약 투여시 발현된 이상반응을 빈도와 발현기관 (SOC, System Organ Class)에 따라 다음과 같이 분류하였다. 빈도는 다음과 같이 정의된다. 매우 흔하게 (>1/10), 흔하게 (>1/100, <1/10), 흔하지 않게 (>1/1,000, <1/100), 드물게 (>1/10,000, <1/1,000), 매우 드물게 (<1/10,000), 알려지지 않음 (이용 가능한 자료에서 측정되지 않음)

|  | 매우 흔하게 | 흔하게 | 흔하지 않게 | 드물게 |
| --- | --- | --- | --- | --- |
| 감염 |  |  | 감염 | 게실염 |
| 혈액 및 림프계 장애 |  |  |  | 호산구증가, 백혈구 감소증 |
| 면역계 장애 |  |  |  | 과민 반응 |
| 대사 및 영양 장애 |  |  | 식욕장애 | 체액저류, 고칼륨혈증, 고요산혈증 |
| 정신신경계 장애 |  |  | 불안, 우울, 불면 | 정신 혼란, 비정상적인 꿈 |
| 신경계 장애 |  | 어지럼증, 두통, 미각 장애 | 지각 이상, 실신 | 졸림, 떨림 |
| 귀 및 미로 장애 |  |  | 이명, 현훈 |  |
| 심혈관계 장애 |  |  | 부정맥, 심계항진 | 심근경색, 빈맥 |
| 혈관 장애 |  | 고혈압 |  |  |
| 호흡기계, 흉부 및 종격동 장애 |  |  | 천식, 기관지 경련, 호흡 곤란 |  |
| 위장관 장애 | 소화 불량 | 복통, 변비, 설사, 식도염, 고창, 위/십이지장궤양*, 위염, 구역, 구토 | 위장관 출혈, 구내염 | 설염, 토혈, 직장 출혈 |
| 피부 및 피하조직 장애 |  | 피부 발진 | 피부염, 다한증, 가려움증, 두드러기 | 탈모, 반상 출혈 |
| 근골격계 및 연결조직 장애 |  | 관절통 | 근육통 |  |
| 신장 및 비뇨기계 장애 |  |  |  | 단백뇨, 신부전 |
| 생식계 및 유방 장애 |  |  |  | 월경 장애 |
| 전신 및 투여 부위 장애 |  | 부종 | 무기력, 피로, 발열 |  |
| 실험실적 검사 |  |  | 비정상적 간기능 검사 수치, 혈청크레아티닌 상승 |  |

*: 계획된 일반 내시경에 의해 확인됨.

1. 나프록센의 임상시험 및 시판 후 조사에서 이상반응은 다음과 같다.

과민증: 쇽증상 (호흡 곤란, 혈압 강하, 서맥, 식은땀, 부종 등), PIE 증후군 (호산구증가를 수반하는 폐침윤: 발열, 천명, 기침, 가래를 동반함), 아나필락시양 반응, 두드러기, 발진, 가려움 등,

소화기계: 토혈, 위장 출혈, 소화성 궤양, 천공, 위부 불쾌감, 위통, 복통, 구역, 구토, 식욕 부진, 소화 불량, 가슴 쓰림, 설사, 변비, 구내염, 복부 팽만감, 흑변, 구갈, 식도염, 위염, 설염, 트림, 고창, 흑토증, 췌장염, 대장염, 염증성 장 질환 (궤양성 대장염, 크론병)의 악화, 직장 출혈, 궤양성 구내염

혈액: 무과립구증, 호산구 증다, 과립구 감소증, 범혈구 감소증, 백혈구 감소, 혈소판 감소, 재생 불량성 빈혈, 용혈성 빈혈, 혈소판 기능 저하 (출혈 시간의 연장), 임파선염

간장: GOT, GPT치의 상승, 황달, 간염, 간부전, 담즙울체

신장: 신장애, 사구체 신염, 혈뇨, 간질성 신염, 신증후군, 신장 유두 괴사, 단백뇨, 신부전, 핍뇨/다뇨

정신신경계

- 졸음, 인식 기능 장애 드물게 현기, 두통, 수족 저림, 정력 감퇴, 피로, 우울, 비정상적인 꿈, 집중 능력 상실, 지남력 상실, 불면, 근육통, 근쇠약, 동요, 불안, 정신 혼란, 환각, 신경과민. 무기력, 근무력증

- 무균성 수막염 (심한 두통, 구역, 구토, 불면, 목이 뻣뻣함, 발열 또는 의식 장애 등의 증상이 나타나면 즉시 투여를 중단하고 의사와 상의한다.)

호흡기계: 호흡 곤란, 천식, 기관지 경련, 호산구성 폐렴, 폐렴, 폐부종, 호흡 저하

피부: 가려움증, 반상 출혈, 발진, 습진, 발한, 자반, 광과민증, 탈모, 발적, 괴사성 용해, 다형성홍반, 스티븐스-존슨증후군 (피부점막안증후군), 두드러기, 표피 수포증, 결절성 홍반, 고정약물 발진, 편평태선, 만발성피부포르피린증, 탈락성 피부염, 농포성 반응

감각기계: 이명, 청각 장애, 시각 장애

대사 및 영양: 부종, 체액저류, 고혈당, 저혈당, 체중변화, 고칼륨 혈증, 고요산혈증

감염: 게실염, 감염, 패혈증

기타: 혈관신경부종, 심계항진, 울혈성심부전, 불임, 월경장애, 오한, 발열, 맥관염, 혈청크레아티닌 수치 상승

1. 에스오메프라졸 장용코팅제제의 임상시험 및/또는 시판 후 조사에서 보고된 이상반응은 다음과 같다. 용량 상관성은 없었다.

|  | 흔하게 | 흔하지 않게 | 드물게 | 매우 드물게 | 빈도 불명 |
| --- | --- | --- | --- | --- | --- |
| 혈액 및 림프계 장애 |  |  | 백혈구 감소증, 저혈소판증 | 무과립구증, 범혈구 감소증 |  |
| 면역계 장애 |  |  | 발열, 혈관부종, 아나필락시스 반응, 쇼크와 같은 과민 반응 |  |  |
| 대사 및 영양 장애 |  | 말초 부종 | 저나트륨혈증 | 저마그네슘혈증 |  |
| 정신신경계 장애 |  | 불면 | 동요, 정신 혼란, 우울 | 공격성, 환각 |  |
| 신경계 장애 | 두통 | 어지럼증(현기), 지각 이상, 졸음 | 미각 장애 |  |  |
| 시각 장애 |  |  | 시야 혼탁 |  |  |
| 귀 및 미로장애 |  | 현훈 |  |  |  |
| 호흡기계, 흉부 및 종격동 장애 |  |  | 기관지 경련 |  |  |
| 위장관계 장애 | 복통, 설사, 고창, 구역/구토, 변비 | 구강 건조 | 구내염, 위장관계 칸디다증 | 대장의 현미경적 염증 | 클로스트리듐  디피실레성 설사 |
| 간담도계 장애 |  | 간 효소 증가 | 황달을 동반하거나 동반하지 않는 간염 | 간부전, 기존 간질환 이 있는 환자에서 간성 뇌병증 |  |
| 피부 및 피하조직 장애 |  | 피부염, 소양증, 두드러기, 발진 | 탈모, 광감수성 | 다형성 홍반, 스티 븐스-존슨 증후군, 독성표피 괴사용해 (TEN, 일부 치명적임) |  |
| 근골격계 및 연결조직 장애 |  |  | 관절통, 근육통 | 근육 쇠약 |  |
| 신장 및 비뇨기계 장애 |  |  |  | 간질성 신염 |  |
| 생식계 및 유방 장애 |  |  |  | 여성형 유방 |  |
| 전신 및 투여 부위 장애 |  |  | 무력증, 발한 증가 |  |  |

### 시험약의 금기 사항 Contraindications

이 약의 금기 사항은 다음과 같다.

1. 나프록센, 에스오메프라졸, 치환기인 벤즈이미다졸 및 이 약의 구성 성분에 과민증이 있는 환자
2. 아스피린이나 기타 비스테로이드성 소염 진통제 (COX-2 저해제 포함)의 투여에 의하여 천식, 비염, 코의 용종, 두드러기, 알레르기 반응 또는 그 병력이 있는 환자 (이러한 환자에서 비스테로이드성 소염 진통제 투여 후 치명적인 중증의 아나필락시양 반응이 드물게 보고되었다.)
3. 활동성 소화성 궤양 환자
4. 위장관 출혈, 뇌혈관 출혈, 기타 출혈 질환 환자, 심한 혈액 이상 환자
5. 중증 간장애 환자 (예. Childs-Pugh 등급 C)
6. 중증의 심부전 환자
7. 중증의 신장애 환자 (크레아티닌클리어런스 30mL/min 미만)
8. 심한 고혈압 환자
9. 관상동맥 우회로술 (CABG) 전후에 발생하는 통증의 치료
10. 임신 말기 3개월 기간에 해당하는 임부
11. 아타자나비어, 넬피나비어 투여 환자

### 시험약의 경고 사항

시험약의 경고 사항은 다음과 같다.

1. 매일 세잔 이상 정기적으로 술을 마시는 사람이 이 약이나 다른 해열 진통제를 복용해야 할 경우 반드시 의사 또는 약사와 상의해야 한다. 이러한 사람이 이 약을 복용하면 위장 출혈이 유발될 수 있다.
2. 심혈관계 위험: 이 약을 포함한 비스테로이드성 소염 진통제는 중대한 심혈관계 혈전 반응, 심근경색증 및 뇌졸중의 위험을 증가시킬 수 있으며, 이는 치명적일 수 있다. 투여 기간에 따라 이러한 위험이 증가될 수 있다. 심혈관계 질환 또는 심혈관계 질환의 위험 인자가 있는 환자에서는 더 위험할 수도 있다.

의사와 환자는 이러한 심혈관계 증상의 발현에 대하여 신중히 모니터링하여야 하며, 이는 심혈관계 질환의 병력이 없는 경우에도 마찬가지로 적용된다.

환자는 중대한 심혈관계 독성의 징후 및/또는 증상 및 이러한 증상이 발현되는 경우 취할 조치에 대하여 사전에 알고 있어야 한다.

1. 위장관계 위험: 이 약을 포함한 비스테로이드성 소염 진통제는 위 또는 장관의 출혈, 궤양 및 천공을 포함한 중대한 위장관계 이상반응의 위험을 증가시킬 수 있으며, 이는 치명적일 수 있다. 나프록센 단독 투여에 비하여 이 약이 위궤양 발생을 유의하게 감소시켰으나, 궤양 및 그와 관련된 합병증은 여전히 발생된다. 이러한 이상반응은 투여 기간 동안에 경고 증상 없이 발생할 수 있다. 고령자는 중대한 위장관계 이상반응의 위험이 더 클 수 있다. 투여 기간이 길어질수록 중대한 위장관계 이상반응의 발생 가능성이 증가될 수 있으나 단기 투여시 이러한 위험이 완전히 배제되는 것은 아니다. 이 약을 투여하는 동안 위장관계 궤양 또는 출혈의 증상 및 징후에 대하여 신중히 모니터링 하여야 하며, 중증의 위장관계 이상반응이 의심되는 경우 즉시 추가적인 평가 및 치료를 실시하여야 한다. 비스테로이드성 소염 진통제를 중증의 위장관계 이상반응이 완전히 배제될 때까지 투여 중단하는 것도 치료법이 될 수 있다. 고위험군의 환자에게는 비스테로이드성 소염 진통제와 관련 없는 다른 대체 치료제를 고려하여야 한다.

### 시험약의 주의 사항 Precautions

##### 신중 투여

1. 소화성 궤양의 병력이 있는 환자
2. 혈액 이상 또는 그의 병력이 있는 환자
3. 출혈 경향이 있는 환자 (혈소판 기능저하가 나타날 수 있다.)
4. 간장애 또는 그의 병력이 있는 환자
5. 신장애 또는 그의 병력이 있는 환자 및 신혈류량이 저하된 환자
6. 심기능 장애 환자
7. 고혈압 환자
8. 과민증의 병력이 있는 환자
9. 기관지 천식 환자
10. 유도성 포르피린증 환자, 전신성홍반성루푸스 (SLE) 환자 및 혼합결합조직 질환(MCTD) 환자
11. 고령자

##### 일반적 주의사항

1. 이 약을 투여하기 전에 이 약 및 다른 대체 치료법의 잠재적인 위험성과 유익성을 고려해야 한다. 치료시 경과를 충분히 관찰하고 증상에 따라 이 약을 최소 용량 및 적절한 치료 기간으로 투여하여야 한다.
2. 소염 진통제에 의한 치료는 원인 요법이 아닌 대증 요법임에 유의한다.
3. 이 약을 장기간 (특히 1년 이상) 투여하는 환자의 경우 정기적으로 검사를 받아야 한다. 임상검사 (뇨 검사, 전혈구 검사 (CBC) 및 이화학적 검사 등 혈액 검사, 간기능 검사, 안과 검사 등)를 실시하고 이상이 있을 경우 감량, 휴약 등의 적절한 조치를 한다. 간질환 또는 신질환과 관련된 임상 증상이나 전신적인 징후 (예: 호산구 증가증, 발진)가 발현되거나 비정상적인 간기능 검사 또는 신기능 검사 결과가 지속되거나 악화되면, 이 약의 투여를 중단해야 한다.
4. 환자의 상태를 충분히 관찰하고 이상반응의 발현에 유의한다. 과도한 체온 강하, 허탈, 사지 냉각 등이 나타날 수 있으므로 특히 고령자 또는 소모성 질환 환자에게는 투여 후 환자의 상태에 충분히 주의한다. 허약자에게는 필요한 최소량으로 신중히 투여하고 이상반응의 발현에 특히 유의한다.
5. 이 약의 약리학적 특성상 염증의 다른 증상과 징후를 불현성화하여 통증성 및 비감염성 조건하에서 감염성 합병증의 진단을 지연시킬 수 있다.
6. 위장관계에 미치는 영향
7. 나프록센을 포함한 비스테로이드성 소염 진통제를 궤양성 질환 또는 위장관 출혈의 병력이 있는 환자에게 처방 시 극심한 주의를 기울여야 한다. 소화성 궤양 질환 및/또는 위장관 출혈의 병력이 있는 환자의 경우 이러한 위험인자가 없는 환자에 비해 비스테로이드성 소염 진통제 투여시 위장관 출혈의 발생 위험이 10배 이상 증가하였다. 위장관계 출혈을 증가시키는 다른 위험 인자로는 경구용 코르티코스테로이드, 항응고제의 병용, 비스테로이드성 소염 진통제의 장기 사용, 알콜 섭취, 고령, 허약한 건강 상태 등이 있다. 치명적인 위장관계 이상반응에 대한 자발적 보고의 대부분은 고령자 및 허약자에 대한 것이므로, 이러한 환자에게 이 약을 투여시 특별히 주의하여야 한다. 미소프로스톨 또는 프로톤펌프저해제와의 병용 투여는 이러한 환자에서 고려되어야 한다.
8. 이 약 투여 중 위장관의 출혈이나 궤양 발생시 투여를 중단한다.
9. 위장관계 질환 (궤양성 대장염, 크론병)의 병력이 있는 환자는 신체 상태가 악화될 수 있으므로 비스테로이드성 소염 진통제를 신중히 투여해야 한다.
10. 에스오메프라졸로 인해 악성 종양의 증상이 완화되거나 진단이 지연될 수 있으므로 악성 종양이 의심되는 경고 증상 (의도하지 않은 현저한 체중 감소, 재발성 구토, 삼킴곤란, 토혈이나 흑토증 등)이 있으면서 위궤양이 있거나 의심되는 경우, 검사를 실시하여 악성 종양 여부를 확인하여야 한다.
11. 프로톤펌프 저해제의 투여는 살모넬라 및 캄필로박터와 같은 위장관 감염의 위험을 약간 증가시킬 수 있다.
12. 에스오메프라졸은 저위산증 또는 무위산증으로 인한 비타민 B12 (시아노코발라민)의 흡수를 감소시킬 수 있으므로, 장기간 치료에서 비타민 B12 흡수를 감소시키는 위험 인자 또는 체내 저장이 감소된 환자는 충분한 주의가 요구된다.

### 약물 상호 작용 Drug Interactions

1. 항레트로바이러스제제:
2. 아타자나비어: 건강한 지원자에게 오메프라졸 (20mg 1일 1회 투여)과 아타자나비어 300mg/리토나비어 100mg을 병용 투여했을때 아타자나비어의 노출이 감소했다 (AUC, C_max_, C_min_이 약 75% 감소). 아타자나비어 400mg 1일 4회 투여는 오메프라졸의 아타나자비어 노출에 대한 영향을 상쇄시킬 수 없다. 따라서 이 약과의 병용 투여해서는 안된다.
3. 넬피나비어: 오메프라졸 (1일 40mg)의 병용 투여시 넬피나비어의 평균 AUC, C_max_, C_min_이 36~39%까지 감소했다. 활성대사체 M8 (히드록시-t-부틸아미드)의 평균 AUC, C_max_ 및 C_min_은 75-92%까지 감소했다. 따라서 이 약과의 병용 투여해서는 안된다.
4. 사퀴나비르: 오메프라졸 (1일 40mg)과 병용 투여시 사퀴나비르의 혈중 농도가 증가하여 이상반응 위험이 증가할 수 있으므로 환자의 개별 상태에 따라 감량하는 등 신중히 투여한다.
5. COX-2 선택적 억제제 포함한 다른 비스테로이드성 소염 진통제 (NSAIDs): 나프록센 유도체와 병용할 경우 위장관 궤양 및 출혈과 같은 이상반응의 위험이 증가할 수 있으므로 병용하지 않는다.
6. 히단토인계 항간질제, 설파제, 설포닐요소계 혈당 강하제: 이러한 약물과 병용 투여시 그 작용을 증강시킬 수 있으므로 충분히 관찰하고 신중히 투여한다.
7. 프로프라놀롤 및 다른 β-차단제: 이 약과의 병용 투여로 인해 항고혈압 효과가 감소될 수 있다.
8. ACE저해제 (캅토프릴 등)
9. 비스테로이드성 소염 진통제에 의해 ACE 저해제의 항고혈압 효과가 감소될 수 있다는 보고가 있으므로 이 약과 ACE 저해제를 병용 투여하는 경우 이러한 상호작용을 염두에 두어야 한다.
10. ACE 저해제 (캅토프릴)와의 병용에 의해, 신장애를 일으킬 수 있으므로, 병용하는 경우에는 신중히 투여한다.
11. 루프계 이뇨제 및 치아짓계 이뇨제
12. 임상시험 및 시판 후 조사 결과 이 약의 신장에서의 프로스타글란딘 합성 억제에 의해 일부 환자에서 푸로세미드 및 치아짓계 이뇨제의 나트륨뇨 배설 효과가 감소할 수 있음이 확인되었다. 이들 약물과 비스테로이드성 소염 진통제를 병용 투여하는 동안 신부전 징후를 면밀히 관찰해야 한다.
13. 이 약은 루프계 이뇨제 (푸로세미드, 피레타니드) 및 치아짓계 이뇨제의 작용을 감소시킬 수 있으므로 병용 투여하는 경우에는 신중히 투여한다.
14. 프로베네시드: 병용 투여시 이 약의 혈중 농도가 상승하여 혈중 반감기가 연장되므로 감량하는 등 신중히 투여한다.
15. 아스피린: 아스피린과의 병용이 비스테로이드성 소염 진통제의 사용과 관련된 중대한 심혈관계 혈전 반응의 위험을 감소시킬 수 있다는 일관된 증거는 없다. 다른 비스테로이드성 소염 진통제와 마찬가지로 이 약과 아스피린의 병용에 의해 중증의 위장관계 이상반응의 발생 위험이 증가될 수 있으므로 두 약물의 병용은 일반적으로 권장되지 않는다.
16. 리튬: 비스테로이드성 소염 진통제는 신장에서의 프로스타글란딘 합성 억제에 의해 혈청 리튬의 농도를 증가시키고 리튬의 신클리어런스를 감소시킬 수 있다. 비스테로이드성 소염 진통제와 리튬의 병용 투여 시 리튬 중독을 일으킬 수 있으므로, 병용하는 경우 리튬의 독성 징후를 주의깊게 관찰하고 감량하는 등 신중히 투여해야 한다.
17. 메토트렉세이트

프로톤펌프 억제제와 함께 투여될 때, 몇몇 환자에서 메토트렉세이트의 수치 증가가 보고되었다. 비스테로이드성 소염 진통제 (NSAIDs)와의 병용 투여로 신세뇨관에서 메토트렉세이트의 배설이 지연되어 치명적인 메토트렉세이트의 혈액학적 독성이 증가될 수 있으므로 항암요법으로 사용하는 고용량의 메토트렉세이트와는 병용 투여하지 않으며, 저용량의 메토트렉세이트와 병용 투여시 신중히 투여되어야 한다.

1. 쿠마린계 항응혈제 (와파린 등)
2. 위장관계 출혈에 대하여 와파린과 비스테로이드성 소염 진통제는 상승 작용을 나타낼 수 있으므로 두 약물을 함께 사용하는 환자는 단독으로 투여하는 경우에 비해 중증의 위장관계 출혈의 위험이 높아질 수 있다.
3. 쿠마린계 항응혈제와 병용 투여시 그 작용을 증강시킬 수 있으므로 충분히 관찰하고 신중히 투여한다.
4. 지도부딘: 지도부딘의 대사를 억제하고 혈중 농도를 상승시킬 수 있으므로, 병용하는 경우에는 감량하는 등 신중히 투여한다.
5. 뉴퀴놀론계 항생 물질 (에녹사신 등): 이 약과 병용에 의해 경련을 일으킬 수 있으므로 신중히 투여한다.
6. 사이클로스포린 및 타크로리무스: 비스테로이드성 소염 진통제와 사이클로스포린 혹은 타크로리무스가 병용 투여될 때 신독성의 위험이 있다.
7. 선택적 세로토닌 재흡수 억제제 (SSRIs): COX-2 선택적 저해제를 포함한 비스테로이드성 소염 진통제와 SSRIs의 병용 투여는 위장관 출혈의 위험을 증가시킨다.
8. 코르티코스테로이드: COX-2 선택적 저해제를 포함한 비스테로이드성 소염 진통제를 코르티코스테로이드와 병용 투여시 위장관 출혈의 위험이 증가한다.
9. 콜레스티라민: 나프록센을 포함한 비스테로이드성 소염 진통제와 병용 투여시 흡수를 지연시킬 수 있다.
10. 디곡신과 같은 강심배당체와의 병용 투여 시 비스테로이드성 소염 진통제는 혈장 강심배당체의 농도를 증가시킬 수 있다. 또한 건강한 환자에서 디곡신과 오메프라졸 (1일 20mg) 병용 투여시 디곡신의 생체이용률이 10%까지 (10명 중 2명에서 30%까지) 증가하였다.
11. 클로피도그렐: 오메프라졸과의 병용 투여시 클로피도그렐 활성대사체의 노출이 감소하고, 평균 혈소판 응집억제 효과도 감소하므로, 에스오메프라졸과의 병용 투여는 권장되지 않는다.
12. 위 산도에 의해 흡수에 영향을 받는 약물: 이 약 투여 중 위내 산도 감소로 인해 위 산도에 의해 흡수 기전이 영향을 받는 약물의 흡수가 증가 또는 감소될 수 있다. 다른 산 분비 억제제나 제산제와 마찬가지로 이 약 투여 중에는 케토코나졸과 이트라코나졸의 흡수가 감소될 수 있다.
13. CYP2C19, CYP3A4 저해제/유도제 및 기질

에스오메프라졸은 CYP2C19과 CYP3A4에 의해 대사된다. 그리고 주대사 효소인 CYP2C19를 저해한다.

1. 디아제팜, 시탈로프람, 이미프라민, 클로미프라민, 페니토인, 와파린 등과 같이 CYP2C19에 의해 대사되는 약물과 병용 시 이들 약물의 혈장 농도가 증가될 수 있으므로 이들 약물의 감량이 필요할 수 있다. CYP2C19 기질인 디아제팜과 이 약 30mg의 병용 투여로 디아제팜의 청소율이 45% 감소되었다 (간질 환자에게 페니토인과 에스오메프라졸 40mg의 병용 투여로 페니토인의 혈장 농도 최저치가 13%가 되었다. 이 약을 투여하기 시작하거나 중단할 때 페니토인의 혈장 농도를 모니터링하도록 한다).
2. 오메프라졸 (1일 1회 400mg 투여)은 보리코나졸 (CYP2C19 기질)의 C_max_와 AUC_τ_를 각각 15% 및 41% 증가시켰다.
3. 에스오메프라졸과 CYP3A4 저해제인 클래리스로마이신 (500mg 1일 2회 투여)을 동시에 투여하면 에스오메프라졸의 AUC가 2배로 증가된다.
4. CYP2C19 및/또는 CYP3A4 유도제로 알려진 약물 (예, 리팜피신, 세인트존스워트)은 에스오메프라졸 대사를 증가 시킴으로써 에스오메프라졸 혈중 농도를 감소시킬 수 있다.
5. 실로스타졸: 건강한 환자에게 오메프라졸 40mg을 투여한 교차시험에서, 실로스타졸 C_max_와 AUC는 각각 18%와 26% 증가했다. 활성 대사체들 중 3,4-디히드로실로스타졸은 C_max_와 AUC가 각각 29%와 69%까지 증가했다.

### 특수 인구 집단

1. 임부에 대한 투여
2. 이 약을 임부에게 투여한 잘 조절된 임상자료는 없다. 나프록센의 동물실험에서 주산기에 투여하여 분만 지연, 난산의 발생 빈도 증가 및 새끼의 생존율 감소가 보고되어 있으므로 임신 말기에는 이 약을 투여하지 않는다.
3. 임신 말기의 랫트에 투여한 실험에서 태자의 동맥관 수축이 보고된 바 있다. 다른 비스테로이드성 소염 진통제와 마찬가지로 임신 말기에 이 약을 투여 시 태아의 동맥관을 조기 폐쇄시킬 수 있으므로 이 약은 임부에게 투여를 피해야 한다.
4. 임부에 대한 안전성은 확립되어 있지 않으므로 임신하고 있을 가능성이 있는 부인에는 치료 상의 유익성이 위험성을 상회한다고 판단되는 경우에만 투여한다.
5. 수유부에 대한 투여
6. 이 약 중의 나프록센은 모유로의 이행이 일어나 신생아에서 프로스타글란딘 합성을 억제하는 이상반응을 일으키므로 수유부에는 투여하지 않는다.
7. 소아에 대한 투여
8. 18세 이하의 소아에 대한 안전성 및 유효성이 확립되어 있지 않으므로 투여를 권장하지 않는다.
9. 고령자에 대한 투여
10. 일반적으로 고령자는 소염 진통제에 의한 소화관의 궤양, 출혈 등의 발현이 높으므로 필요한 최소량으로 투여하고 이상반응의 발현에 특히 유의하는 등 환자의 상태를 관찰하면서 신중히 투여하거나, 다른 대체약 투여를 고려해야 한다.

### 대조약의 사용 상 주의 사항

### 대조약의 이상반응 Adverse Events

1. 대조약인 쎄레브렉스 캡슐의 임상시험에서 보고된 이상반응은 다음과 같다.
2. 위약 또는 활성 대조약과의 비교 임상시험에 의하면, 이상반응으로 인한 약물 투여 중단 비율은 이 약 투여군에서 7.1%, 위약군에서 6.1%이었다. 이 약 투여를 중단시킨 이상반응 중 가장 빈번한 것은 소화 불량과 복통이었다. 이 약과의 인과관계에 상관없이, 이 약의 투여후 성인에서 보고된 이상반응은 다음과 같다.
3. 위장관계: 소화 불량 (8.8%), 설사 (5.6%), 과민성 대장 증후군, 배변 횟수 증가, 때때로 복통, 상복부 통증, 고창, 구역, 변비, 게실염, 삼킴 곤란, 트림, 식도염, 위장염, 위장관 궤양, 위식도 역류, 위 불쾌감, 위장 장애, 치질, 열공성 헤르니아, 혈변, 구강 건조, 구내염, 뒤무직, 치아 질환, 구토, 위장관 염증의 악화, 구각미란, 드물게 장폐색증, 장관 천공, 위장관 출혈, 출혈성 대장염, 식도천공, 췌장염, 장패쇄증, 치아 발치후 치조골염, 장문합 궤양, 흑색변, 설염, 아프타성 구내염, 구강 점막 수포형성, 입의 감각이 둔해짐, 구강내 통증이 나타날 수 있다.
4. 중추 및 말초 신경계: 두통 (15.8%), 때때로 어지러움, 다리 경련, 긴장항진, 지각 감퇴, 편두통, 신경통, 신경병증, 지각 이상, 뇌경색, 드물게 무균성 수막염 운동실조, 자살, 후각 상실, 무미각증
5. 전신: 때때로 등통, 알레르기 악화, 알레르기 반응, 무력, 가슴 통증, 체액저류/말초성 부종, 전신성 부종, 얼굴 부종, 피로, 권태감, 열, 얼굴 홍조, 인플루엔자성 증후군, 통증, 말초성 통증, 드물게 패혈증, 급사, 아나필락시스모양 반응, 혈관 부종
6. 정신신경계: 때때로 불면증, 식욕 부진, 불안, 식욕 증가, 우울증, 신경 과민 반응, 졸음, 치명적 두개내 출혈, 착란, 환각, 간질 악화
7. 호흡기계: 상기도 감염 (8.1%), 부비강염 (5.0%), 때때로 인두염, 비염, 기관지염, 기관지 경련, 악화성 기관지 경련, 기침, 호흡 곤란, 후두염, 폐렴, 드물게 코피, 발성 장애
8. 피부 및 부속기관: 수포성 피부염, 반상 출혈, 때때로 발진, 탈모증, 피부염, 손톱 질환, 광민감 반응, 소양감, 홍반성 발진, 반점상 구진의 발진, 피부 질환, 피부 건조, 발한 증가, 두드러기, 다형홍반, 박탈 피부염, 피부점막안 증후군 (스티븐-존슨증후군), 독성표피괴사용해 (리엘증후군), 습진, 반상 출혈, 지방종, 알레르기 피부염, 드물게 점상 출혈, 비듬, 호산구 증가와 전신 증상을 동반한 약물 발진 (drug rash with eosinophilia and systemic symptom, DRESS 또는 과민 반응 증후군)
9. 심혈관계: 부정맥, 심실 비대, 때때로 악화성 고혈압, 고혈압, 협심증, 불안정 협심증, 관상동맥 장애, 심근경색증, 심부전, 울혈심부전, 심계항진, 빈맥, 뇌졸중, 대동맥반폐쇄부전증, 혈종, 동성서맥, 드물게 실신, 심실성 세동, 폐색전증, 뇌혈관 발작, 말초성 괴저, 혈전성 정맥염, 혈관염, 심부정맥 혈전증, 뇌출혈
10. 감염: 상기도감염, 단독(erysipelas), 상처감염, 치은감염, 때때로 단순포진, 대상포진, 박테리아성 감염, 진균성 감염, 연조직 감염, 바이러스성 감염, 모닐리아증, 생식기 모닐리아증, 중이염
11. 생식기계: 유방 압통, 폐경기 증상, 때때로 흉부 유선 섬유종증, 흉부신생물 (여성), 가슴 통증 (여성), 월경통, 월경 장애, 질출혈, 질염, 전립선 장애, 난소낭증
12. 귀: 때때로 청각 상실, 귀의 이상, 이통, 이명, 난청
13. 간 및 담도계: 때때로 간기능 이상, ALT 상승, AST 상승, γ-GTP 증가, ALP 증가, 요(尿) 우로빌리노겐 양성, 드물게 담석증, 간염, 황달, 간부전, 전격성 간염, 간괴사
14. 대사 및 영양: 혈중 나트륨 증가, 혈중 칼륨 증가, 때때로 BUN 증가, CPK 증가, 당뇨병, 고콜레스테롤혈증, 고혈당증, 저칼륨증, 고칼륨혈증, NPN 증가, 크레아티닌 증가, 알카리포스파타제 증가, 체중 증가, 식욕 부진, LDH 증가, 드물게 저혈당증, 저나트륨혈증
15. 골격근계: 신경절의 통증, 때때로 관절통, 관절증, 골격 이상, 근육통, 경부긴장증, 활막염, 건염, 근염, 다리 경련, 골절, 건파열, 드물게 등의 통증, 근육 경직
16. 혈액계: 헤마토크리트 감소, 헤모글로빈 증가, 때때로 반상 출혈, 코피, 혈소판혈병, 빈혈, 드물게 혈소판 감소증, 무과립구증, 재생 불량성 빈혈, 범혈구 감소증, 백혈구 감소증
17. 투여부위: 때때로 봉소염, 접촉 피부염, 주사 부위 반응, 피부소결절
18. 특수기관: 때때로 미각도착
19. 요로계: 때때로 알부민뇨, 방광염, 혈뇨, 배뇨 곤란, 빈뇨증, 신결석, 요실금, 요로 감염
20. 눈: 때때로 시야혼몽, 백내장, 결막염, 눈의 통증, 녹내장, 드물게 안구 출혈, 망막동맥 또는 정맥폐색
21. 신장: 혈중 크레아티닌 증가, β2-마이크로글로불린 증가, 때때로 NAG 증가, 요잠혈 양성, 요단백 양성, 드물게 신기능 장애, 급성 신부전, 간질성 신염
22. 국내 시판 후 조사 결과

국내에서 6년 동안 실시한 시판후 사용성적조사 결과, 약과의 인과관계에 상관없이 총5,648례 중 305례 (5.40%)에서 338건의 이상반응이 보고되었다. 소화불량이 155건 (2.74%)으로 가장 많았으며, 얼굴 부종 54건 (0.96%), 복통 33건 (0.58%), 구역 13건 (0.23%), 말초성 부종, 발진 각 12건 (0.21%), 설사 10건 (0.18%), 불면증 7건 (0.12%) 순으로 나타났다.

기타 이상반응으로 두통, 현기증이 각 5건 (0.09%) 보고되었으며, 변비, 위궤양, 두드러기, 빈혈이 각 2건 (0.04%), 구갈, 궤양성 구내염, 위염, 알레르기, 요통,

가슴 통증, 고지혈증, 당뇨, 체중 증가, 골절, 관절통, RA factor 양성, 대상 포진, 폐렴, 호흡 곤란, ALT 상승, 난청이 각 1건 (0,02%) 보고되었다.

1. 국외 시판후 조사 결과

담즙정체, 담즙정체간염, 황달, 신증후군, 미세변화신증후군 (minimal change disease, MCD), 급성전신발진농포증 (actue generalized exanthematous pustulosis, AGEP), 여성 생식 능력 감소

### 대조약의 금기사항 Contraindications

1. 이 약 및 이 약의 구성 성분에 대해 과민 반응 또는 그 병력이 있는 환자
2. 설폰아미드에 대해 알레르기 반응을 보이는 환자
3. 아스피린이나 다른 비스테로이드성 소염 진통제 (COX-2 억제제 포함)에 대하여 천식, 급성 비염, 비측 폴립, 혈관부종, 두드러기, 또는 알레르기 반응 병력이 있는 환자
4. 중증 간장애 환자
5. 중증 신장애 환자 (크레아티닌 청소율 <30 mL/min인 환자)
6. 활동성 소화성 궤양 또는 위장관 출혈 환자
7. 크론병 또는 궤양성 대장염과 같은 염증성 장 질환 환자
8. 울혈성 심부전 환자 (NYHA II - IV)
9. 확립된 허혈성 심장질환자, 말초성 동맥 질환 환자 및/또는 뇌혈관 질환 환자
10. 임부 또는 임신하고 있을 가능성이 있는 여성
11. 수유부
12. 관상동맥 우회로술 (CABG) 전후에 발생하는 통증의 치료
13. 고칼륨혈증 환자
14. 이 약은 유당을 함유하고 있으므로, 갈락토오스 불내성 (galactose intolerance), Lapp 유당분해효소 결핍증 (Lapp lactase deficiency) 또는 포도당-갈락토오스 흡수 장애 (glucose-galactose malabsorption) 등의 유전적인 문제가 있는 환자에게는 투여하면 안된다.

### 대조약의 경고사항

시험약의 경고 사항은 다음과 같다.

1. 매일 세잔 이상 정기적으로 술을 마시는 사람이 이 약이나 다른 해열 진통제를 복용해야 할 경우 반드시 의사 또는 약사와 상의해야 한다. 이러한 사람이 이 약을 복용하면 위장 출혈이 유발될 수 있다.
2. 심혈관계 위험: 이 약을 포함한 비스테로이드성 소염 진통제는 중대한 심혈관계 혈전 반응, 심근경색증 및 뇌졸중의 위험을 증가시킬 수 있으며, 이는 치명적일 수 있다. 투여 기간에 따라 이러한 위험이 증가될 수 있다. 심혈관계 질환 또는 심혈관계 질환의 위험 인자가 있는 환자에서는 더 위험할 수도 있다.

의사와 환자는 이러한 심혈관계 증상의 발현에 대하여 신중히 모니터링하여야 하며, 이는 심혈관계 질환의 병력이 없는 경우에도 마찬가지로 적용된다.

환자는 중대한 심혈관계 독성의 징후 및/또는 증상 및 이러한 증상이 발현되는 경우 취할 조치에 대하여 사전에 알고 있어야 한다.

1. 위장관계 위험: 이 약을 포함한 비스테로이드성 소염 진통제는 위 또는 장관의 출혈, 궤양 및 천공을 포함한 중대한 위장관계 이상반응의 위험을 증가시킬 수 있으며, 이는 치명적일 수 있다. 나프록센 단독 투여에 비하여 이 약이 위궤양 발생을 유의하게 감소시켰으나, 궤양 및 그와 관련된 합병증은 여전히 발생된다. 이러한 이상반응은 투여 기간 동안에 경고 증상 없이 발생할 수 있다. 고령자는 중대한 위장관계 이상반응의 위험이 더 클 수 있다. 투여 기간이 길어질수록 중대한 위장관계 이상반응의 발생 가능성이 증가될 수 있으나 단기 투여시 이러한 위험이 완전히 배제되는 것은 아니다. 이 약을 투여하는 동안 위장관계 궤양 또는 출혈의 증상 및 징후에 대하여 신중히 모니터링 하여야 하며, 중증의 위장관계 이상반응이 의심되는 경우 즉시 추가적인 평가 및 치료를 실시하여야 한다. 비스테로이드성 소염 진통제를 중증의 위장관계 이상반응이 완전히 배제될 때까지 투여 중단하는 것도 치료법이 될 수 있다. 고위험군의 환자에게는 비스테로이드성 소염 진통제와 관련 없는 다른 대체 치료제를 고려하여야 한다.

### 대조약의 주의사항

##### 신중 투여

1. 간장애 환자 또는 그 병력이 있는 환자
2. 신장애 환자 또는 그 병력이 있는 환자 (크레아티닌청소율<60mL/min인 환자)
3. 기관지 천식 환자
4. 심부전 환자 또는 그 병력이 있는 환자
5. 고혈압 환자 또는 그 병력이 있는 환자
6. 체액저류 환자 또는 부종의 병력이 있는 환자
7. 이뇨제나 ACE 저해제를 투여 중인 환자
8. 저혈량증의 위험이 있는 환자
9. 탈수 환자
10. 고령자
11. 소화성 궤양이나 위장관 출혈의 병력이 있는 환자
12. 심혈관계 이상반응 (심장 발작, 뇌졸중 등)에 대한 고도의 위험 인자를 가진 환자 (예: 고혈압, 고지혈증, 당뇨, 흡연 등), 심혈관계 질환 또는 그 병력이 있는 환자
13. CYP2C9에 의한 대사가 어려운 환자
14. 혈액 응고 장애가 있거나 항응고제를 투여받고 있는 환자
15. 임신을 계획하는 여성 (이 약을 복용시 여성 생식 능력이 손상될 수 있다.)
16. 당뇨병 환자

##### 일반적 주의사항

1. 이 약을 투여하기 전에 이 약 및 다른 대체 치료법의 잠재적인 위험성과 유익성을 고려해야 한다. 이 약의 복용량과 노출 기간이 증가할수록 심혈관계 위험도가 커지므로 가능한 최단 기간 동안 최소 유효 용량으로 투여한다. 특히 골관절염 (퇴행관절염) 환자의 경우에 주기적으로 재평가를 해야 한다.
2. 위장관계 이상반응: 이 약을 포함한 비스테로이드성 소염 진통제를 궤양성 질환이나 위장관 출혈의 병력이 있는 환자에게 처방시 극심한 주의를 기울여야 한다. 소화성 궤양 질환 및/또는 위장관 출혈의 병력이 있는 환자의 경우 이러한 위험 인자가 없는 환자에 비해 비스테로이드성 소염 진통제 투여시 위장관 출혈의 발생 위험이 10배 이상 증가하였다. 위장관계 출혈을 증가시키는 다른 위험인자로는 경구용 코르티코스테로이드 또는 항응고제 병용, 비스테로이드성 소염 진통제 또는 아스피린의 병용 알콜 섭취, 흡연, 고령, 허약한 건강 상태 등이 있다. 치명적인 위장관계 이상반응에 대한 자발적 보고의 대부분은 고령자 및 허약자에 대한 것이므로, 이러한 환자에게 이 약을 투여시 특별히 주의하여야 한다.
3. 고혈압: 이 약을 포함한 비스테로이드성 소염 진통제는 고혈압을 일으키거나, 기존의 고혈압을 악화시킬 수 있으며, 이로 인해 심혈관계 이상반응의 발생률이 증가될 수도 있다. 티아지드계 이뇨제 또는 루프계 이뇨제를 복용중인 환자가 비스테로이드성 소염 진통제 복용시 이들 요법에 대한 반응이 감소될 수 있다. 이 약을 포함한 비스테로이드성 소염 진통제는 고혈압 환자에서 신중히 투여해야 한다. 이 약의 투여 초기와 투여기간 동안에 혈압을 면밀히 모니터링해야 한다
4. 울혈심부전 및 부종: 이 약을 포함한 비스테로이드성 소염 진통제를 복용하는 일부 환자에서 체액 저류 및 부종이 관찰되었다. 프로스타글란딘 합성 저해로 신기능의 악화 및 체액저류가 유발될 수 있으므로 이 약은 심부전, 좌심실 기능 장애, 또는 고혈압의 병력이 있는 환자나 부종이나 체액저류가 있는 환자에서 신중히 투여해야 한다. 또한 이뇨제를 투여하고 있거나 다른 사유로 혈량 저하증의 위험이 있는 환자의 경우 또한 이 약 투여시 주의하여야 한다.
5. 비스테로이드성 소염 진통제를 장기간 복용시 신장유두괴사나 기타 신장 손상이 일어날 수 있다. 또한, 신혈류를 유지하는데 프로스타글란딘의 역할이 중요하므로 심부전 환자, 신부전 환자, 간부전 환자, 이뇨제, ACE 억제제 또는 안지오텐신II 길항제를 투여 중인 환자, 고령자 등에서는 특별한 주의가 필요하다. 투약을 중단하면 대부분 치료 전 상태로 회복된다.
6. 진행된 신질환: 진행된 신질환 환자에서 이 약 사용에 대한 통제된 임상 시험은 실시된 바 없다. 따라서, 진행된 신질환 환자에 대해서는 이 약의 투여가 권장되지 않는다. 이 약의 투여를 개시해야 한다면, 환자의 신장 기능에 대해서 면밀히 관찰해야 한다.
7. 이 약을 포함한 비스테로이드성 소염 진통제의 투여로 간기능 수치의 상승이 나타날 수 있다. 이러한 비정상적인 검사 수치는 치료가 지속됨에 따라 악화되거나 변화가 없거나 또는 일시적일 수 있다. 또한 이 약을 포함한 비스테로이드성 소염 진통제 투여로, 황달, 치명적 전격성 간염, 간괴사, 간부전 (일부는 치명적임)을 포함한 중증의 간 관련 이상반응이 드물게 보고되었다. 간기능 이상을 암시하는 증상 및/또는 징후가 있는 환자 또는 간기능 시험 결과 비정상인 환자에 있어서는 투여 기간 동안 주의깊게 간기능의 악화 여부를 관찰하고, 간질환과 관련된 임상 증상이나 전신적인 징후 (예, 호산구증가증, 발진)가 발현되는 경우에는 이 약의 투여를 중지한다.
8. 이 약의 장기간 투여로 빈혈이나 혈액 손실의 증상 또는 징후가 나타나는 경우에는 헤모글로빈치 또는 헤마토크리트치 검사를 해야 한다. 이 약은 일반적으로 혈소판 수치나 프로트롬빈 시간 (PT), 부분트롬보플라스틴시간 (PTT)에는 영향을 미치지 않으며 권장 용량에서 혈소판 응집을 억제하지 않는다.
9. 비스테로이드성 소염 진통제를 장기간 투여하는 환자는 정기적으로 전혈구 검사(CBC) 및 이화학적 검사를 실시해야 한다. 간질환 또는 신질환과 간련된 임상 증상이나 전신적인 징후 (예, 호산구증가증, 발진)가 발현되거나 비정상적인 간기능 검사 또는 신기능 검사 결과가 지속되거나 악화되면, 이 약의 투여를 중단해야 한다.
10. 아나필락시스모양 반응: 다른 비스테로이드성 소염 진통제와 마찬가지로 아낙필락시스모양 반응은 약물에 노출된 경험이 없는 환자에서도 일어날 수 있다. 이러한 복합증상은 아스피린이나 다른 비스테로이드성 소염 진통제 투여 후 비측 폴립을 동반하거나 동반하지 않거나 또는 잠재적으로 치명적인 중증의 기관지 경련을 나타내는 천식환자에게 전형적으로 발생한다. 이러한 아나필락시스모양 반응이 나타나는 경우 응급처치를 실시하여야 한다.
11. 피부 반응: 이 약은 설폰아미드 계열의 약물로, 박탈피부염, 피부점막안증후군 (스티븐스-존슨 증후군) 및 독성표피괴사용해 (리엘증후군) 같은 중대한 피부 이상반응을 일으킬 수 있으며, 이는 치명적일 수 있다. 이들 중대한 이상반응은 경고 증상 없이 발생할 수 있으며 설파제 알레르기의 병력이 없는 환자에서도 발생할 수 있다. 대부분의 경우 이러한 이상반응은 투여 초기 1개월 이내에 발생한다. 환자는 중대한 피부 발현 증상 및 증후에 대해 알고 있어야 하며 피부 발진, 점막병변 또는 물집, 발열, 가려움과 같은 과민 반응의 최초 증상 및 징후가 나타날 때 약물 투여를 중단해야 한다.
12. 천식 환자 중 일부는 아스피린에 민감하게 반응할 수 있다. 아스피린 민감성 천식 환자에게 아스피린을 사용하는 경우 치명적일 수 있는 중증의 기관지 경련과 관련될 수 있다. 이러한 아스피린 민감성 환자에게서 아스피린과 다른 비스테로이드성 소염 진통제 간의 기관지 경련을 포함하는 교차반응이 보고되었다. 그러므로 이 약은 이러한 아스피린 민감성 환자에게는 투여하지 않도록 하며, 천식 환자에게는 주의깊게 사용하여야 한다.
13. 이 약은 코르티코이드 제제를 대체하거나 코르티코이드 결핍증을 치료하기 위한 약물로 사용될 수 없다. 코르티코스테로이드의 갑작스러운 투여 중단은 코르티코스테로이드- 반응성 질환의 악화를 초래할 수 있다. 장기간 코르티코스테로이드를 복용해 온 환자에게 이 약을 투여하고자 할 경우에는 서서히 용량을 감소시켜야 한다.
14. 이 약의 약리학적 특성상 발열 및 염증의 다른 증상과 징후를 불현성화하여 통증성 및 비감염성 조건 하에서 감염성 합병증의 진단을 지연시킬 수 있다.
15. 심한 탈수 증상을 보이는 환자에 있어서는 수분을 공급한 후 약물 투여를 시작하며 주의깊게 관찰해야 한다.
16. 성인의 급성통증 완화 (수술후 또는 발치후 진통)의 경우 단기간 (1주일 이내)으로 사용한다.
17. 관상동맥 우회로술 (CABG) 직후 최초 10~14일까지의 통증 치료를 위하여 COX-2 선택성 비스테로이드성 소염 진통제를 투여한 큰 규모의 통제된 임상시험 두 건의 결과 심근경색과 뇌졸중의 발생률이 증가하였다.
18. 정기적 혹은 필요에 따라 임상 검사 (요검사, 혈액검사, 신장기능 검사, 간기능 검사, 심전도 검사 및 대변잠혈검사 등)를 실시해, 이상이 확인될 경우 휴약이나 투여 중지 등의 적절한 처치를 실시해야 한다.
19. 이 약은 저용량 아스피린 (1일 325mg 이하) 이외의 비스테로이드성 소염 진통제와 투여 용량에 관계없이 병용을 피해야 한다.
20. 이 약을 복용하고 어지러움, 졸음 등을 경험한 환자들은 운전이나 기계를 다루는 일은 피해야 한다.
21. 이 약은 혈소판에 대한 작용이 없으므로 심혈관계에 대한 예방 요법으로서 아스피린의 대체약물이 될 수 없다. 이 약을 투여받고 있더라도 항혈소판요법을 받고 있는 환자는 그 치료를 중지해서는 안된다.
22. 소염 진통제에 의한 치료는 원인요법이 아닌 대증요법임에 유의한다.
23. 교차민감도: 하나의 비스테로이드성 소염 진통제에 감수성을 가진 환자는 마찬가지로 다른 비스테로이드성 소염 진통제에도 감수성을 나타낼 수 있다.
24. 자가면역 질환 환자(예, 전신홍반루푸스 (SLE) 및 혼합결합조직병 (MCTD) 환자) 가 이 약을 복용시 무균수막염에 걸릴 위험성이 증가할 수 있다.
25. 이 약은 당뇨병 환자 또는 혈중 칼륨 농도를 증가시키는 약물과 병용 투여시 고칼륨혈증이 나타날 수 있으므로 이러한 경우 칼륨 수치의 정기적인 관찰이 필요하다.
26. 비스테로이드성 소염 진통제를 장기간 복용하는 여성에서 일시적인 불임이 보고되었다.
    - - 1. **약물 상호 작용 Drug Interactions**
27. 이 약은 주로 간에서 CYP 2C9에 의해 대사되므로, CYP 2C9를 억제하는 약물과 병용 투여할 경우에는 주의하여야 한다.
28. 플루코나졸 200 mg을 1일 1회 병용투여시 플루코나졸에 의해 이 약의 CYP 2C9을 통한 대사가 억제되어 이 약의 혈중 농도가 2배 증가되었으므로 이 약의 병용을 시작하는 경우 최저 권장량으로 투여한다. 플루바스타틴과 병용시 이 약 및 플루바스타틴의 혈중 농도가 증가할 가능성이 있다. 리팜피신, 카르바마제핀, 바르비투르계 약물 등 CYP 2C9의 유도제와의 병용은 이 약의 혈중 농도를 감소시킬 수 있다.
29. In vitro 연구에 의하면, 이 약은 CYP 2D6을 억제하는 것으로 밝혀졌으므로, CYP 2D6에 의해 대사되는 약물과 in vivo에서 상호작용을 일으킬 수도 있다. 이 약과 병용투여시 CYP 2D6의 기질인 항우울제(삼환계 및 SSRIs), 신경이완제, 항부정맥약물, 덱스트로메트로판 등의 혈중농도가 증가할 수 있다. 이 약의 병용투여를 시작하는 경우에는 이러한 약물의 용량 감소가, 이 약의 병용투여를 중단하는 경우에는 이러한 약물의 용량 증가가 필요할 수 있다.
30. In vitro 연구에서 이 약이 CYP 2C19에 의해 매개되는 대사를 일부 저해하는 것으로 확인되었다. 이러한 결과의 임상적 유의성은 알려지지 않았으나, CYP 2C19에 의해 대사되는 약물에는 디아제팜, 시탈로프람 및 이미프라민 등이 있다.
31. ACE 억제제 또는 안지오텐신II 수용체 길항제: 비스테로이드성 소염 진통제에 의해 ACE 억제제 또는 안지오텐신II 수용체 길항제의 항고혈압효과가 감소될 수 있으므로 이 약과 ACE 억제제 또는 안지오텐신II 수용체 길항제를 병용투여하는 경우 이러한 상호작용을 염두에 두어야 한다. 신기능이 저하된 환자(예: 탈수환자 또는 고령자)에게 이 약을 포함한 비스테로이드성 소염 진통제를 ACE 억제제나 안지오텐신 Ⅱ 수용체 길항제와 병용 투여하는 경우 일반적으로 가역적인 급성 신부전의 위험이 증가할 수 있다. 그러므로 특히 고령자에게는 이러한 병용투여시 주의하여야 한다. 환자에게 적절한 수분을 공급하여야 하며, 이러한 병용투여를 시작한 후 정기적으로 신기능을 모니터링 하여야 한다.
32. 이뇨제: 임상시험 및 시판후 조사 결과 이 약의 신장에서의 프로스타글란딘 합성 억제에 의해 일부 환자에서 푸로세미드 및 티아지드계 이뇨제의 나트륨뇨배설 효과가 감소할 수 있음이 확인되었다. 이들 약물과 비스테로이드성 소염 진통제를 병용투여하는 동안 신부전 징후를 면밀히 관찰해야 한다.
33. 아스피린과의 병용이 비스테로이드성 소염 진통제의 사용과 관련된 중대한 심혈관계 혈전반응의 위험을 감소시킬 수 있다는 일관된 증거는 없다. 저용량의 아스피린 (1일 325mg 이하)과 병용투여할 수 있으나, 이 약을 단독 투여하는 것보다 위장관계 이상반응 (위장관 궤양) 또는 다른 위장관계 합병증의 발생률이 높아지는 것이 보고되었다. 심혈관계에 대한 예방요법으로서 아스피린을 투여할 경우에, 이 약은 혈소판에 대한 작용을 나타내지 않으므로 아스피린의 대체 약물이 될 수 없다.
34. 리튬: 비스테로이드성 소염 진통제는 신장에서의 프로스타글란딘 합성 억제에 의해 혈청 리튬의 농도를 증가시키고 리튬의 신청소율을 감소시킬 수 있다. 따라서 비스테로이드성 소염 진통제와 리튬의 병용 투여시 리튬의 독성 징후를 주의깊게 관찰해야 한다.
35. 메토트렉세이트: 비스테로이드성 소염 진통제와의 병용투여로 신세뇨관에서 메토트렉세이트의 배설이 지연되어 치명적인 메토트렉세이트의 혈액학적 독성이 증가될 수 있으므로 항암 요법으로 사용하는 고용량 (15 mg/주 이상)의 메토트렉세이트와는 병용 투여하지 않으며, 저용량의 메토트렉세이트와 병용 투여 시 신중히 투여되어야 한다. 세레콕시브를 류마티스 관절염 환자에게 메토트렉세이트와 병용 투여 (류마티스 치료용량) 시 유의한 영향을 미치지 않았다. 그러나 두 약물을 병용 투여하는 경우 메토트렉세이트와 관련된 독성에 대한 적절한 모니터링을 고려하여야 한다.
36. 쿠마린계 항응고제 (와르파린 등): 위장관계 출혈에 대하여 와르파린과 비스테로이드성 소염 진통제는 상승작용을 나타낼 수 있으므로 두 약물을 함께 사용하는 환자는 단독으로 투여하는 경우에 비해 중증의 위장관계 출혈의 위험이 높아질 수 있다. 와르파린의 항응고 활성에 대한 이 약의 효과를 보기 위하여 건강한 시험대상자에게 와르파린 1일 2-5 mg을 투여한 시험에 의하면, 프로트롬빈 시간의 측정에 의한 항응고 활성에는 영향을 미치지 않았다. 그러나, 시판 후 조사에 의하면, 와르파린과 이 약을 병용 투여한 환자에서 치명적일 수도 있는, 프로트롬빈 시간의 연장과 관련된 중대한 출혈이 보고된 바 있으며, 특히 고령자에서 더 많이 보고되었다.
37. 비스테로이드성 소염 진통제와 시클로스포린 또는 타크로리무스와의 병용 투여에 의해 시클로스포린 또는 타크로리무스의 신독성이 증가할 수 있으므로 이들 약물과 병용투여시 신기능을 모니터링하여야 한다.
38. 이 약을 고지방 식이와 함께 복용한 경우 최고 혈장 농도가 약 1-2시간 지연되어 나타났으며, 총흡수도 (AUC)가 10-20% 증가하였다.
39. 이 약을 알루미늄이나 마그네슘을 함유한 제산제와 병용 투약한 경우 최고 혈중 농도의 37% 감소와 함께 총흡수도의 10% 감소가 나타났다. 병용 투여하는 동안 신부전 징후를 면밀히 관찰해야 한다.
40. 저용량의 아스피린 (1일 325mg 이하) 이외에 다른 비스테로이드성 소염 진통제 와 병용 투여할 경우 이상반응의 위험이 증가할 수 있으므로 병용 투여하지 않아야 한다.
41. 코르티코스테로이드: 위장관계의 이상반응 (예, 궤양, 출혈)이 증가할 위험이 있다. 특히, 고령자 (65세 이상)에게 위험이 있다.
    - - 1. **특수 인구 집단**
42. 임부 및 수유부에 대한 투여
43. 랫트에 대한 실험에서 이 약은 분만을 지연시키지 않는 것으로 보고되어 있다. 그러나 이 약이 임부의 분만 및 출산에 미치는 영향은 알려져 있지 않다.
44. 토끼의 배ㆍ태자 발달시험에서 150 mg/kg/day 이상 (사람에게 200 mg씩 1일 2회 투여 시 AUC_0-24_의 약 2배에 해당하는 용량) 경구 투여 시 태자의 늑골융합, 흉골분절융합 등의 태아 이상과 심실중격결손의 발현율이 드물게 증가하였다. 랫트의 배ㆍ태자 발달시험에서는 두 시험 중 하나에서 30 mg/kg/day 이상 (사람에게 200 mg씩 1일 2회 투여 시 AUC_0-24_의 약 6배에 해당하는 용량) 경구 투여 시 태자에서 횡경막 헤르니아의 용량 의존적 증가가 관찰되었다. 또한, 랫트의 50 mg/kg/day 이상 (사람에게 200 mg씩 1일 2회 투여 시 AUC_0-24_의 약 6배에 해당하는 용량) 투여 시 착상 전 손실, 착상 후 손실 및 태자의 생존율 감소를 유발하였다. 이와 같은 독성은 약물 투여를 중지하고 2주 후에 임신 시에는 관찰되지 않았다.
45. 이 약을 임부에게 투여한 임상 자료는 없다. 동물실험 (토끼 및 랫트)에서 이 약의 투여로 기형 발생을 포함한 생식 독성이 나타났으나, 사람의 임신에 있어서의 잠재적인 위험은 알려져 있지 않다. 또한 이 약은 프로스타글란딘 합성을 저해하는 다른 약물과 마찬가지로 임신 말기에 투여 시 자궁 무력증을 일으키거나 태아의 동맥관을 조기 폐쇄시킬 수 있으므로 이 약은 임부 또는 임신할 가능성이 있는 여성에게는 투여하지 않는다. 이 약을 투여하는 동안에 임신이 확인된 경우에는 이 약의 투여를 중지한다.
46. NSAIDs 계열 약물의 사용은 그 작용기전으로 인해 난포파열을 방해하거나 지연시킬 수 있으며, 이는 일부 여성에게 가역적 불임을 일으킬 수 있다. 따라서 임신이 어렵거나 불임 검사를 받고 있는 여성의 경우 이 약을 포함한 NSAIDs 계열 약물의 사용중단을 고려해야 한다.
47. 프로스타글란딘 합성 억제 작용은 임신에 불리한 영향을 끼칠 수 있다. 역학 조사 에서 임신초기 프로스타글란딘 합성을 억제하는 약물을 복용한 후 자연 유산의 위험이 증가하는 것으로 나타났다. 동물시험에서는 프로스타글란딘 합성을 억제하는 약물을 투여할 경우 착상 실패가 증가하는 것으로 나타났다.
48. 이 약은 혈장과 유사한 농도로 랫트의 모유로 배설된다. 1명의 시험대상자에 대한 제한적인 자료에 의하면, 이 약은 사람의 모유로도 이행되는 것으로 판단된다. 많은 약물들이 모유 중으로 이행될 뿐만 아니라 이행될 경우 영아에서 심각한 이상반응의 발생이 우려되므로, 수유부에 대한 약물 투여의 중요성을 고려하여 수유를 중단하거나 약물 투여를 중단해야 한다.
49. 소아에 대한 투여
50. 만 18세 이하의 소아 및 청소년에 대한 안전성과 유효성은 확립되지 않았다.
51. 고령자에 대한 투여
52. 고령자는 신기능, 간기능 및 특히 심기능이 저하되어 있을 가능성이 높으므로 고령자에게 이 약을 투여하는 경우 적절한 관찰이 요구된다.
53. 임상 시험에 의하면, 고령자와 젊은 시험대상자간의 효과는 유의한 차이가 나타나지 않았다. 신장 기능 (GFR, BUN, 크레아티닌) 및 혈소판 기능 (출혈 시간, 혈소판 응집 정도)을 비교한 임상 시험에서도, 고령자와 젊은 시험대상자 사이에는 차이가 없었다. 그러나, 선택적인 COX-2 억제제를 포함한 다른 비스테로이드성 소염 진통제와 마찬가지로, 치명적인 위장관 이상반응과 급성 신부전에 대한 시판후의 자발적인 이상반응은 고령자에서 더 많이 보고되었다.
54. 일반적으로 고령자에 대한 용량 조절은 요구되지 않으나, 체중이 50 kg 이하인 경우에는 최소량에서 투여를 시작한다.

## 포장 및 라벨링

임상시험용 의약품은 의뢰자에 의해 공급될 것이다. 임상시험용 의약품은 임상시험 약사/자격 있는 의약품 취급자에 의해 임상시험 실시기관에서 관리되어야 한다.

본 임상시험에 사용되는 임상시험용 의약품은 지함 별로 포장되어 공급될 예정이다. 시험약인 낙소졸 정이나 대조약인 쎄레브렉스 캡슐이 개별 지함 별로 포장되어 공급될 것이다.

임상시험용 의약품의 지함 별 기재 사항은 1개의 판넬 위에 검정색으로 의약품 등의 안전에 관한 규칙 69조 제 6항에 부합되게 작성될 예정이며, 기재 사항은 다음을 포함한다.

- “임상시험용”이라는 표시
- 의약품 배정 식별 번호
- 제품의 코드명 또는 주성분의 일반명
- 제조 번호 및 사용 (유효) 기한 또는 재검사 일자
- 저장 방법
- 임상시험 계획 승인자의 상호명과 주소
- "임상시험 외의 목적으로 사용할 수 없음"이라는 표시

직접용기 라벨의 경우에는 공간이 협소한 관계로 임상시험용이라는 표시, 임상시험 계획 승인자의 상호, 시험대상자 등록 번호, 제품의 코드명 또는 주성분의 일반명, 재검사 일자, 제조 번호만을 기재하도록 한다.

## 공급, 취급 및 보관

**공급**

시험약과 대조약은 문성환 (세브란스병원 정형외과)를 통해 공급된다

**취급**

시험약과 대조약은 임상시험 약사/자격 있는 의약품 취급자에 의해 임상시험 실시기관에서 관리되어야 한다.

**보관**

기밀용기에 보관한다.

사용 기간:

- 시험약: 별도 표기
- 대조약: 별도 표기

**보관 및 취급 상의 주의사항**

1. 어린이의 손이 닿지 않는 곳에 보관할 것
2. 다른 용기에 바꾸어 넣는 것은 사고 원인이 되거나 품질 유지 면에서 바람직하지 않으므로 이를 주의할 것.

## 임상시험용의약품의 교부

시험자는 임상시험 교부 관련 기록의 유지를 포함하여 임상시험용 의약품의 교부 전반에 대한 책임이 있다.

- 임상시험용 의약품을 수령 받게 되면 시험자 또는 그 대리인은 수령 내용에 대하여 확인하며, 이에 대해 서명 (이니셜 포함)과 확인 날짜를 의뢰자 또는 의뢰자의 업무 위임자의 양식 문서에 기록을 남기고 문서를 의약품 공급자에게 다시 돌려준다. 복사본은 임상시험 시험자 파일에 보관한다.
- 필요할 경우 임상시험용 의약품을 수령 받는 자는 운송 도중 보관 온도에 대한 log를 함께 전달 받고 이를 보관한다.
- 임상시험용 의약품의 교부 내용은 시험자 또는 의뢰자의 시험약 입출고 내역 양식에 정확히 기록한다. 모니터링 방문 시 모니터 요원은 수불 사항이 정확한지 여부를 점검할 수 있어야 한다.

시험자는 또한 아래와 같은 문서를 기록하여야 한다.

- 시험대상자에게 임상시험 연구 계획서와 변경 사항에 따라 임상시험용 의약품이 투여되었다는 확인 문서
- 공급된 임상시험용 의약품이 모두 수불상 차이 없이 적절히 관리되고 있다는 확인 문서
- 필요할 경우 임상시험용 의약품이 보관 조건 (온도 등)에 부합되게 보관되고 있다는 확인 문서

사용되지 않은 임상시험용 의약품은 폐기되거나 본 임상시험 이외의 목적으로 사용되어서는 안 된다. 시험대상자에게 교부되었던 임상시험용 의약품은 다른 시험대상자에게 재교부되어서는 안 된다.

## 치료 순응도

임상시험 중 투여된 임상시험용 의약품의 수는 기록에 남겨질 것이며, 투여된 임상시험용 의약품의 갯수를 셈으로써 임상시험용 의약품의 투약 순응도를 평가할 것이다.

## 눈가림법

- 임상시험용 의약품을 구별할 수 없음을 확인

본 임상시험에서는 시험약 (낙소졸 정)과 대조약 (쎄레브렉스 캡슐) 중 어느 것을 투여 받고 있는지에 대해서 이중 눈가림법이 적용된다. 본 임상시험에 사용되는 시험약 (낙소졸 정)과 대조약 (쎄레브렉스 캡슐)은 색깔과 포장이 다르나, 시험약에 대해 동일한 성상, 모양, 포장을 하고 있는 시험약에 대한 위약과 대조약에 대해 동일한 성상, 모양, 포장을 하고 있는 대조약에 대한 위약을 채택한 이중 위약 (double-dummy) 임상시험이다. 따라서 임상시험 기간 동안 평가자인 시험자와 시험대상자의 이중 눈가림이 유지될 것이다.

시험 종료 이전에 시험자와 시험대상자는 시험약을 투여 받았는지, 대조약을 투여 받았는지 알 수 없다.

- 무작위 배정 코드와 눈가림 봉투의 준비 및 유지

무작위 배정 코드는 의뢰자 또는 의뢰자의 업무를 위임 받은 무작위 배정 생성 담당자가 생성한다. 생성된 무작위 배정 코드는 문성환 (세브란스병원 정형외과)의 시험약과 대조약 포장 담당자에게 전달될 것이다. 응급 눈가림 해제가 필요할 경우 사용하기 위하여 눈가림 해제 봉투가 시험자에게 제공된다. 눈가림 해제 봉투는 시험대상자에 대한 시험군이 각각 봉인된 채 눈가림이 해제되는 시점까지 안전하게 보관된다. 시험 종료 후 모니터 요원이 눈가림 해제 봉투가 봉인된 채로 있는지 확인한다.

- 임상시험용 의약품의 수집

눈가림 해제 이전에는 눈가림 상태를 유지해야 한다. 이를 위해 잔여 임상시험용 의약품은 적절한 방법에 의하여 보관 및 봉인되어야 한다.

- 이중 눈가림 해제

시험 종료 이전에 시험자와 시험대상자는 시험약을 투여 받았는지, 대조약을 투여 받았는지 알 수 없다. 시험 종료 후에는 결과 분석을 위하여 의뢰자 측에서 시험자에게 코드를 공개하게 된다.

임상시험이 모두 종료되어 정해진 절차에 의해 무작위 배정 코드가 해제되지 않는 한 무작위 배정 코드에 접근할 수 없다.

단, 시험자의 안전성 및 유효성 평가를 위해서 전체 시험대상자 또는 시험대상자 개인 별 눈가림은 해제될 수 있다.

## 응급 눈가림 해제

시험자에게 제공된 눈가림 해제 봉투는 응급 상황에서만 개봉된다. 눈가림을 해제하여야 하는 경우에는 시험자는 시험대상자의 무작위 배정군 배정에 관한 눈가림을 해제하기 전에 의뢰자 측의 담당자에게 연락하기 위해 최대한 노력을 해야 한다. 시험대상자 무작위 배정군의 눈가림이 해제 되면, 즉시 전화로 의뢰자 측에 알려야 하며, 별도 문서에 그 이유와 해당 날짜를 기재한다. 개봉된 눈가림 해제 봉투는 시험자가 날짜를 기록하고 서명을 하여야 한다.

## 과다 투여에 대한 처치

현 시점까지 본 시험약이 인체내 투여된 경험이 없으므로, 만일 인체내 과량 투여가 발견되면 시험자는 시험대상자를 입원시키고 이상반응이 나타나는지 관찰하며, 적절한 증상 치료를 실시하여야 한다.

- 시험약

1. 임상시험 중에 이 약의 과량 투여는 보고된 바 없다. 12명 환자에 대하여 최대 2400 mg/day의 용량으로 최대 10일 동안 투여시 중증의 독성이 나타나지 않았다.
2. 비스테로이드성 소염 진통제의 과량 투여에 의한 증상은 일반적으로 무기력, 졸음, 구역, 구토, 상복부 통증 등이며, 보조적인 치료로서 회복 가능하다. 위장관 출혈도 일어날 수 있으며, 드물게 고혈압, 급성 신부전, 호흡 억제와 혼수 상태 등이 일어날 수 있다.
3. 보통 대증적이고 보조적인 치료를 실시하며 특정한 해독제는 없다.
4. 약물 복용한 지 4시간 이내에 증상이 나타나거나 매우 다량 복용한 경우에는 구토, 활성탄 (성인 60-100 g, 소아 kg당 1-2 g), 삼투압 설사를 이용할 수 있다.
5. 혈액 투석, 강제 이뇨, 요의 알칼리화, 혈액 관류 등의 치료는 세레콕시브의 높은 단백결합률 때문에 유용하지 않다.

- 대조약

1. 임상시험 중에 이 약의 과량 투여는 보고된 바 없다. 12명 환자에 대하여 최대 2400 mg/day의 용량으로 최대 10일동안 투여 시 중증의 독성이 나타나지 않았다.
2. 비스테로이드성 소염 진통제의 과량 투여에 의한 증상은 일반적으로 무기력, 졸음, 구역, 구토, 상복부 통증 등이며, 보조적인 치료로서 회복 가능하다. 위장관 출혈도 일어날 수 있으며, 드물게 고혈압, 급성 신부전, 호흡 억제와 혼수 상태 등이 일어날 수 있다.
3. 보통 대증적이고 보조적인 치료를 실시하며 특정한 해독제는 없다.
4. 약물 복용한 지 4시간 이내에 증상이 나타나거나 매우 다량 복용한 경우에는 구토, 활성탄 (성인 60-100 g, 소아 kg당 1-2 g), 삼투압 설사를 이용할 수 있다.
5. 혈액 투석, 강제 이뇨, 요의 알칼리화, 혈액관류 등의 치료는 세레콕시브의 높은 단백결합률 때문에 유용하지 않다.

## 시험 종료 후 시험대상자의 진료 및 치료

임상시험 기간 중 발생한 이상반응에 대하여 임상시험 실시기관의 치료 기준, 일반적으로 받아들여지는 의학적 치료 지침, 시험대상자 별로 원하는 의학적 필요성에 따라 보편적으로 행해지는 치료가 시험대상자에게 시행될 것이다.

# 임상시험 절차 및 평가

임상시험 절차와 평가는 아래와 같이 실시될 예정이다.

## 평가 일정

임상시험의 절차에 대한 전반적인 요약은 임상시험 흐름도를 통해 제시하였다 (Appendix I 참조). 모든 방문은 정해진 일정에 따라 이루어져야 한다. 시험대상자 별 임상시험 기간은 12주~14주이며, 다음과 같은 평가 일정으로 이루어져 있다.

이상반응이 발생할 경우 추가적인 방문이 실시될 수 있다.

- Screening and washout period: 0~2주 (day -14~day1)
- Treatment & F/U period: 12주 (day 1~day 84)

임상시험 기간 중 방문 일정은 1개의 치료 전 스크리닝 방문을 포함하여 모두 3개의 방문으로 이루어졌다. 2번째 방문에서 무작위 배정과 시험약 교부가 이루어지며, 마지막 방문은 임상시험용 의약품 투여일 기준 12주 후이다.

**방문 1 (Visit 1): 스크리닝 방문 (Day -14~Day1)**

방문 1당일에 시험자는 모든 시험대상자로부터 시험대상자 동의서를 확보한 이후, 시험대상자를 대상으로 모든 선정 기준을 만족시키는지 여부와 어떠한 제외 기준에도 해당되지 않는지 여부를 평가한다. 본 임상시험에 적합할 것으로 판단되면 시험자는 시험대상자에 대하여 아래와 같은 절차 및 평가를 시행한다.

- 시험대상자 동의서에 서명 받음
- 스크리닝 번호 부여
- 인구학적 자료 기록
- 현재 질환, 과거 병력, 타 임상시험 참여 여부, 음주 습관, 흡연력, 기타 선정/제외 기준 항목 조사 기록
- 신체 검진 (체중 측정 포함)
- 활력징후 (혈압, 맥박 수, 호흡수)
- 혈액 검체 수집을 위한 채혈
- 임신반응 검사 (가임기 여성만 해당)
- 약물력 확인
- 투여 전 이상반응 확인
- 병용금기 의약품을 복용 중일 경우 washout 함을 지시
- (해당할 경우) 방문 1 이후 최대 14일 이내 Day 1에 방문 2를 예약
- 증례기록서 작성

**방문 2 (Visit 2): 무작위 배정 방문 (Randomization Visit) (Day 1)**

Day 1에 방문 2를 실시한다. 시험자는 시험대상자에 대하여 아래와 같은 절차 및 평가를 시행한다.

- 선정/제외 기준 부합 여부를 재확인
- 통증 VAS 실시
- LDQ 평가 실시
- 무작위 배정
- 모든 새로운 이상반응 그리고/또는 기존의 이상반응의 변화를 기록
- 모든 새로운 병용약물 그리고/또는 기존의 병용약물의 변화를 기록
- GSRS 및 EQ-5D 설문지를 시험대상자에게 교부하고 설문지에 답하도록 함
- 임상시험용 의약품 교부
- 보충약제 또는 구제약물 복용법 및 시험대상자 일지 작성법을 설명
- 보충약제 또는 구제약물 교부
- 시험대상자 일지 작성 확인 및 방문 3에 일지 지참하도록 상기시킴
- 방문 2기준 84일 후 (day 84±7)에 방문 3을 예약
- 증례기록서 작성

**방문 3 (Day 84±7)**

다음과 같은 평가 및 치료가 시행된다.

- 모든 새로운 이상반응 그리고/또는 기존의 이상반응의 변화를 기록
- 모든 새로운 병용약물 그리고/또는 기존의 병용약물의 변화를 기록
- 통증 VAS 실시
- LDQ 평가 실시
- GSRS 및 EQ-5D 설문지를 시험대상자에게 교부하고 설문지에 답하도록 함
- 신체 검진
- 활력징후 (혈압, 맥박 수, 호흡수)
- 혈액 검체 수집을 위한 채혈 실시
- 교부된 임상시험용 의약품, 보충약제, 구제약물 중 사용안된 약물을 회수
- 작성된 시험대상자 일지 회수
- 증례기록서 작성

**조기 중단 방문**

이 방문 기간 중 방문 3에 준하여 아래와 같은 절차와 평가를 실시한다.

- 조기 중단 사유를 기록
- 모든 새로운 이상반응 그리고/또는 기존의 이상반응의 변화를 기록
- 모든 새로운 병용약물 그리고/또는 기존의 병용약물의 변화를 기록
- 통증 VAS 실시
- LDQ 평가 실시
- GSRS 및 EQ-5D 설문지를 시험대상자에게 교부하고 설문지에 답하도록 함
- 신체 검진
- 활력징후 (혈압, 맥박 수, 호흡 수)
- 혈액 검체 수집을 위한 채혈 실시
- 교부된 임상시험용 의약품, 보충약제, 구제약물 중 사용안된 약물을 회수
- 작성된 시험대상자 일지 회수
- 증례기록서 작성

**추가 방문**

이상반응의 추적 조사 등을 위해 추가 방문을 실시할 수 있다. 추가 방문의 평가 사항은 시험자의 판단에 따르지만 다음과 사항이 포함될 수 있다.

- 모든 새로운 이상반응 그리고/또는 기존의 이상반응의 변화를 기록
- 모든 새로운 병용약물 그리고/또는 기존의 병용약물의 변화를 기록
- 신체 검진
- 활력징후 (혈압, 맥박 수, 호흡 수)
- 혈액 검체 수집을 위한 채혈 실시
- 증례기록서 작성

## 인구학적정보 및 기타 기저값 평가

다음의 변수를 포함하여 인구학적 특성과 기타 기저 정보에 대해 평가한다.

- 연령, 성별 등 인구학적 정보
- 현병력과 과거력의 유무 등 건강 상태에 대한 정보
- 약물력 및 현 병용 약물 등 기타 약물 복용에 대한 사항 (low dose aspirin 병용 여부 포함)
- 타 임상시험 참여 여부 (스크리닝 기준 30일 이내)
- 체중
- 유효성 평가와 관련 있는 다음의 설문 평가 (LDQ, GSRS, 통증 VAS, EQ-5D)

## 안전성 평가

임상시험용 의약품의 안전성은 시험대상자의 기저 질환, 이상반응, 활력징후를 포함한 신체 검진 상 소견, 임상검사실 검사 결과를 기록, 보고, 분석함으로써 평가될 것이다.

임상시험약 투여 후 발생하는 이상반응의 중증도에 대하여 CTCAE (Common Terminology Criteria for Adverse Events)를 기준으로 평가한다.

동의서에 서명한 시점부터 이후 임상시험이 진행되는 동안 시험대상자가 경험한 이상반응 등 약물의 독성에 대하여 포괄적인 평가가 수행될 것이다. 임상시험에 관련된 자들은 시험자에 의해 파악되고 시험대상자가 보고한 모든 이상반응들을 보고하여야 한다 (Section 6.3.1.2, “이상반응 보고 및 평가방법” 참조).

이상반응 보고 시한은 Section 6.3.1.4와 6.3.1.5을 참조 바란다.

### 이상반응

### 이상반응 정의

##### 이상반응

이상반응은 임상시험용 의약품을 투여한 시험대상자에게 발생한 모든 유해하고 의도하지 않은 증후 (sign, 실험실 실험 결과의 이상 등을 포함한다.), 증상 (symptom) 또는 질병을 말하며, 해당 임상시험용 의약품과 반드시 인과관계를 가져야 하는 것은 아니다.

외과적 또는 진단을 위한 처치의 경우 처치 자체보다는 처치를 초래한 상태 또는 질환을 이상반응으로 취급할 것이다.

사망 예의 경우는 사망을 초래한 원인이 이상반응으로 취급될 것이며 사망은 이상반응으로 인한 경과 (outcome)로 취급될 것이다.

다음의 항목들은 이상반응에 포함되지 않는다.

- 현재 임상시험 대상인 질병이나 질환
- 임상시험용 의약품이나 병용약물을 과량 투여 했으나 증상이 없는 경우
- 의학적 처치 또는 외과적 처치: 상기 처치를 초래한 상황이 이상반응임
- 임상시험 시작 시에 존재하거나 발견된 기존의 질병 또는 상태가 임상시험용 의약품을 투여한 이후 악화되지 않은 경우
- 바람직하지 않은 의학적 사건이 발생한 것이 아닌 경우 (예를 들면 임상시험용 의약품 투여 이후 더 이상 악화되지 않은 기존 질환에 대한 예정된 수술을 위한 입원)

##### 약물과 이상반응와의 인과 관계

임상시험용 의약품 (investigational product)의 투여와 이상반응과의 관계에 대한 평가 (definitely related, probably related, possibly related, unlikely related, not related, not assessable)는 증례기록서를 완료한 시점에서 얻을 수 있는 모든 정보에 근거하여 임상적으로 판단한다.

1. **Definitely related (관련성이 명백함)**

임상시험용의약품과 이상반응 간의 관계가 명백하다고 생각될 경우이다. 연관이 있음 (Probably related)의 기준을 모두 만족하면서 다음의 기준을 모두 만족하는 경우이다.

- 재투여 시 다시 나타나는 경우 (실시된 경우)
- 이상반응이 사용할 임상시험용 의약품 또는 임상시험용 의약품과 동일계열의 의약품에 대해 이미 알려져 있는 정보와 일관된 양상을 보이는 경우

1. **Probably related (관련성이 많음)**

동반 질환 또는 다른 약물, 화학 물질과의 관련성은 없고 임상시험용 의약품의 투여와 합리적인 시간의 연속성이 있는 실험실적 검사 이상 수치와 같은 임상적 사건으로 임상시험용의약품 투여 중단 시 임상적으로 타당한 결과를 볼 수 있는 경우로, 다음의 사항을 모두 만족하는 경우이다.

- 임상시험용 의약품을 투여하였다는 증거가 있는 경우
- 임상시험용 의약품의 투여와 이상반응 발현의 시간적 순서가 타당한 경우
- 이상반응이 다른 가능성 있는 원인보다 임상시험용 의약품에 의해 더욱 개연성 있게 설명된다고 판단되는 경우.
- 임상시험용 의약품 투여를 중단하거나 감량하였을 때 이상반응이 사라지거나 약화되는 경우

1. **Possibly Related (관련성이 의심됨)**

동반 질환 또는 다른 약물, 화학 물질로도 설명할 수 있으나 임상시험용 의약품 투여와 합리적인 시간의 연속성이 있는 임상검사실 검사 이상 수치와 같은 임상적 사건으로 약물 투여 중단에 대한 정보는 없거나 확실치 않은 경우로 다음의 경우에 해당한다.

- 임상시험용 의약품을 투여하였다는 증거가 있는 경우
- 임상시험용 의약품의 투여와 이상반응 발현의 시간적 순서가 타당한 경우
- 이상반응이 다른 가능성 있는 원인들과 비슷한 수준으로 임상시험용 의약품에 기인한다고 판단되는 경우
- 임상시험용 의약품 투여를 중단하거나 감량하였을 때 이상반응이 사라지거나 약화되는 경우

1. **Unlikely related (관련성 적음)**

일반적으로 아래의 경우에 해당되는 경우 관련성 적음 (Unlikely related)이라고 판단한다.

- 임상시험용 의약품을 투여하였다는 증거가 있는 경우
- 이상반응에 대해 임상시험용 의약품 투여보다 다른 원인에 기인할 가능성이 더 큰 경우
- 임상시험용 의약품 투여를 중단하거나 감량했을 때 그 결과가 음성이거나 모호한 경우 (실시된 경우)
- 재투여 실시되었을 경우, 재투여 결과가 음성이거나 모호한 경우

1. **Not related (관련성이 없음)**

다음의 경우에 해당하는 경우

- 시험대상자가 임상시험용 의약품을 투여 받지 않은 경우
- 임상시험용 의약품의 투여와 이상반응 발현의 시간적 순서가 타당하지 않을 경우
- 이유를 설명할 수 있는 확실한 다른 근거가 있는 경우 (예: 수술 부위의 출혈).
- 그럴 듯 하지 않음 (예: 환자가 오토바이에 부딪혔지만 임상시험용 의약품이 사건을 유발할 만큼의 방위 감각 상실을 유발한다는 증거가 없는 경우, 임상시험용 의약품 투여 후 며칠 후에 발생한 암)

1. **Not Assessable (평가 불가능)**

불충분하거나 모순된 정보, 그리고 보충하거나 확인할 수 없는 경우이다.

약물과 이상반응과의 관련성을 평가하기 위하여 아래와 같은 사항들을 고려해야 한다.

- 약물 투여 후 시간적 연속성: 이상반응은 임상시험용 의약품 투여 후 발생해야 한다. 임상시험용 의약품 투여 후 이상반응 발생까지의 시간적 간격은 사건의 임상적 정황에 따라 평가되어야 한다.
- 약물 투여 중단 후 회복, 약물 재투여 후 재발: 약물 투여 중단 후의 시험대상자 반응과 약물 재투여 후의 시험대상자의 반응에 따라 평가한다. (약물 재투여는 문제가 되는 사건에 대한 일상적인 임상적 단계의 관점에서 고려한다.)
- 근본적, 수반성, 병발성 질병: 각각의 이상반응에 대한 보고는 치료받고 있는 질병과 시험대상자가 가지고 있는 다른 질병의 특징 및 진행 단계에 따라 평가한다.
- 병용 약물 및 치료법: 시험대상자가 복용하는 다른 약물 또는 치료법은 문제의 사건과 관련성이 있을 수 있으므로 이들에 대해서도 평가해야 한다.
- 이 계열 약물에 대한 알려진 반응 및 경향: 임상/ 전임상
- 신체적 그리고/또는 정신적 스트레스에의 노출: 스트레스에 대한 노출은 이상반응의 변화를 유발할 수 있으며 이상반응에 대하여 논리적이고 보다 정확한 이유를 제공한다.
- 약물의 약리학, 약동학 특징: 임상시험용 의약품의 약동학 (흡수, 분포, 대사, 배설)은 시험대상자의 약력학 측면과 같이 고려해야 한다.

##### 임상검사실 검사 상 이상 결과 및 기타 검사상 참고 범위외 소견

모든 실험실 검사치 및 측정 항목은 증례기록서에 기록을 하며, 다음의 경우 이상반응으로서 증례기록서의 이상반응 양식에 별도로 기록하고 보고되어야 한다.

- 시험대상자의 임상시험 참여를 중단케 하는 임상검사실 검사 측정값
- 임상적 증상이 있는 경우
- 치료를 필요로 하는 경우
- 기타 시험자에 의해 임상적으로 유의 하다고 판단되는 경우

임상시험용 의약품 투여 후 발견되거나 또는 임상시험 시작 (기준) 시에 존재하였으나 임상시험 시작 이후 악화된 임상적으로 유의한 임상검사실 검사 이상 소견이나 그 밖의 측정값도 이상반응이나 중대한 이상반응이 된다. 그러나 임상시험이 시작될 때에 존재하였거나 발견되었지만 임상시험용 의약품 투여 이후 악화되지 않은 임상적으로 중요한 이상 소견은 이상반응이나 중대한 이상반응이 되지 않는다.

##### 중대한 이상반응

중대한 이상반응 (SAE)은 의약품의 임의 용량에서 발생한 의도하지 않은 의학적 사안의 발생으로 다음과 같다:

- 사망한 경우
- 생명에 대한 위협이 발생한 경우

주의: “생명을 위협하는 경우”의 정의는 이상반응 발생 동안에 시험대상자가 사망할 위험에 도달한 경우를 말하며 이상반응이 악화될 경우 이론적으로 사망에 도달할 수 있는 상황을 이르는 것은 아님.

- 입원할 필요가 있거나 입원 기간을 연장할 필요가 있는 경우

시험계획서와 관련된 처치를 위하여 입원하거나 일상적인 임상 처치로 인해 생긴 입원은 포함하지 않는다. 또한 공식적으로 병원에 입원하는 경우를 말하며 응급실에 온 것은 포함되지 않는다 (단, 24시간이 지나면 입원으로 간주함). 또한 임상시험 참여 전에 계획된 입원이거나, 참여 전부터 있었던 증상이 악화되지는 않은 상태에서 치료하기 위한 입원도 포함되지 않는다. 또한 심미적인 이유 등에 의한 입원 (성형수술 등)은 해당하지 않는다. 그리고, 의학적 사유가 아닌 시험대상자 본인의 의지에 의해 입원 또는 입원 기간의 연장이 있을 경우는 중대한 이상반응으로 간주하지 않는다.

- 영구적이거나 중대한 장애 및 기능 저하를 가져온 경우
- 태아에게 기형 또는 이상이 발생한 경우
- 기타 의학적으로 중대한 이상반응의 경우

의학적으로 중요한 경우에는 사망을 초래하거나 생명을 위협하거나 입원할 필요가 있는 경우가 아니더라도 중대한 이상반응일 수 있다. 이 경우는 적절한 의학적 판단 상 시험대상자들이 위험에 처하게 되거나 상기한 중대한 이상반응을 예방하기 위해서 내과적 또는 외과적 처치가 필요하다고 판단되는 경우가 해당한다. 예컨대 집이나 응급실에서 집중 치료가 요구되는 알러지성 기관 수축 (allergic bronchospasm), 입원 치료의 대상은 아닌 혈액 이상 (blood dyscrasia)이나 경련 (convulsions), 약물 의존성이나 약물 남용의 경우 등이 해당한다.

보고되는 이유가 임상시험용 의약품을 통한 감염이 의심되는 경우 또한 중대한 이상반응으로 취급될 것이며, 이 경우 모두 Section 6.3.1.4에서 기술된 바에 따라 신속히 보고되어야 한다.

##### 이상반응 보고 및 평가 방법

매 방문 시마다 시험대상자는 건강 상태에 대한 질문을 받을 것이다. 임상시험의 이상반응 보고 기간 동안 시험대상자의 보고 또는 시험자의 발견을 통해 알게 된 시험대상자에게 발생한 모든 바람직하지 않은 모든 상태 변화는 이상반응으로서 기록될 것이다.

시험대상자가 이상반응 보고 기간 (아래 기술) 동안 경험한 모든 이상반응 관련 정보들에 대하여 완전하고 정확하고 일관성 있게 임상시험 내내 지속적으로 증례기록서에 기록을 할 것이다. 이상반응 중 중대한 이상반응에 해당하는 것들은 Section 6.3.1.1.4에 기술되어 있는 바에 따라 의뢰자의 중대한 이상반응 보고 양식 (SAE report form) 에 추가 기술하며 보고해야 한다.

각각의 이상반응에 대하여 이상반응에 대한 기술, 기간 (발병일 및 종결일 (임상시험용 의약품의 투약 시점 대비 이상반응이 발생한 시점에 대한 평가가 중요할 경우 “시간”에 대해서도 알아야 함)), 심각도, 임상시험 치료와의 관련성, 기타 의심되는 발생 요인, 시행된 치료와 기타 처치 (임상시험용 의약품의 용량 조정 및 투약 중지), 경과 등에 관하여 보고하여야 한다. 중대한 이상반응은 임상시험 기간 동안 철저히 식별되어야 하며 해당 구분에 따라 기록되고 보고되어야 한다.

##### 이상반응 보고 기간의 정의

이상반응과 중대한 이상반응 보고 개시 시점은 시험대상자가 임상시험에 참여한 날 (최초 동의서 양식에 서명한 날)부터이고, 치료 후 추적 관찰 기간까지 지속된다. 또한, 임상시험 종료 방문 시점에서 진행 중인 이상약물반응이 관찰될 경우 소실되거나 안정화 될 때까지 모니터링 하도록 한다.

##### 중대한 이상반응 보고 절차

기간 중 새롭게 발생한 모든 중대한 이상반응에 대하여 시험자는 즉시 (이상반응 인지 후 24시간 이내) fax나 e-mail 로 의뢰자(또는 이를 대리하는 자) 및 임상시험용 의약품 공급사인 문성환 (세브란스병원 정형외과)의 약물 안전성 관련 담당자에게 연락하여야 한다.

문성환 (세브란스병원 정형외과)의 약물안전성 관리 담당자의 연락처는 다음과 같다.

**이메일: drugsafety@hanmi.co.kr**

**전화:02-410-0446**

**Fax: 050-2260-0479**

이미 보고되었던 중대한 이상반응의 추적 정보 (follow-up) 역시 새롭게 발생한 이상반응과 동일한 절차와 기한에 따라 처리되어야 한다.

모든 중대한 이상반응 보고는 중대한 이상반응 보고 양식 (SAE report form)에 따라 시험자에 의해 작성된 후 처리될 것이다.

중대한 이상반응 보고 양식 (SAE report form)에 작성된 정보들은 증례기록서의 해당 section에 기록된 정보와 항상 일치하여야 한다.

시험자/보고자는 추적 관찰 정보 (즉, 추가 정보, 경과 및 최종 평가, 요청에 의한 관련 기록 제공)를 요청하거나 중대한 이상반응과 관련하여 의뢰자의 질문이 있을 경우 최초 보고와 동일한 시한 안에 관련 사항을 처리하여야 한다. 이는 의뢰자가 중대한 이상반응에 대해 적절히 평가하고 신속한 안전성 관련 보고 의무 규정과 관련하여 의뢰자가 법적 처리 기한을 엄수하기 위해 필수적이다.

일부 예외적인 상황에서는 의뢰자가 특별히 위급한 사항을 명확히 하거나 논의하기 위해 시험자를 직접 접촉할 수도 있으나, 추적 관찰은 통상적으로 임상시험 책임을 맡고 있는 모니터 요원에 의해 요청된다.

##### 정부기관, 임상시험 심사위원회, 시험자에게로의 안전성 보고

시험자는 자신의 시험대상자의 중대한 이상반응 (특히 사망의 경우)에 대하여 소속된 임상시험 심사위원회에 보고할 경우, 임상시험 실시기관 별로 규정하고 있는 중대한 이상반응 보고 요건을 따라야 한다. ICH GCP 규정에 따라 의뢰자는 “시험대상자의 임상시험 수행에 부정적인 영향을 줄수 있거나 임상시험 지속에 대한 임상시험 심사위원회의 승인 변경이 될 만한 사안”이 발생하게 되면 이를 시험자에게 통보하여야 한다. 특히 관련 규정에 따라 의뢰자는 중대하고 예상하지 못한 이상약물반응 (SUSAR)에 대해서 시험자에게 알려야 한다. 시험자는 안전성과 관련된 보고 내용을 시험자 파일내에 보관하여야 한다. 각 국가 별로 시험자에 대한 안전성 관련 보고 관련 법규를 고려하여 이에 부합되어야 한다. 법률 규정이 의무화하는 경우에 한하여 의뢰자는 관련 임상시험 심사 위원회에 직접 안전성 관련 보고를 할 수 있으며 이러한 통보 내용을 기록에 남길 것이다. 법률 규정이 의무화하지 않는 경우에는 시험자가 임상시험 심사위원회에 의뢰자가 제공한 안전성 관련 보고를 직접 보고하여야 하며, 이와 관련된 모든 의사 소통 내용 복사본을 임상 시험자 파일에 보관하여야 한다.

의뢰자는 관련 법률과 규정에 따라 보건 당국에 안전성 관련 사항을 보고하여야 한다.

국내에서 시행되는 임상시험의 경우, 약사법 시행 규칙에 따라 중대하고 예상하지 못한 이상약물반응 (SUSAR) 보고가 시행되어야 한다. 시행 규칙에 따르면 사망을 초래하거나 생명을 위협하는 중대하고 예상하지 못한 이상약물반응은 의뢰자가 해당 사실을 보고받거나 알게 된 날부터 7일안에 식품의약품안전처장에게 보고되어야 하며, 이 경우 의뢰자는 이상약물반응에 대한 상세한 정보를 최초 보고일부터 8일 이내에 추가로 보고하여야 한다. 그 밖의 중대하고 예상하지 못한 이상약물반응의 경우 의뢰자가 해당 사실을 보고 받거나 알게 된 날부터 15일 이내에 보고하여야 한다.

##### 이상반응 시험대상자의 모니터링

시험자는 임상시험 기간 중 발생하고 임상시험용 의약품과 연관이 있을 것으로 판단되는 이상반응을 보인 시험대상자에 대해 추적 조사가 불가능하게 되지 않는 한 이상반응이 사라지거나 최종적인 경과가 확인될 때까지 감시하고 추적 관찰하여야 한다. 시험자는 이상반응 정보를 파악하기 위해 적절한 노력을 해야 하며 이를 문서화 해야 한다. 이상반응에 대하여 적절한 추가적 치료와 추적 관찰을 하는 것은 시험자의 책임이다. 의뢰자는 임상시험 도중 발생한 이상반응에 대하여 적극적으로 추적 관찰하여야 하며 관련 정보를 수집하여야 한다. 그러나 중대한 이상반응의 경우 이들 이상반응이 사라지거나 최종적인 경과가 확인될 때까지 이러한 조치들이 지속되어야 하는 반면, 중대한 이상반응이 아닌 경우에는 데이터 베이스를 잠그는 (database lock) 시점까지만 조치들이 지속될 것이다.

### 임부에 대한 노출

본 임상시험에서 사용된 치료와 (피임제와 약물 상호 작용에 따른 결과) 연관이 있다고 시험자에 의하여 판단된 임신만이 이상반응로 간주될 것이다. 그러나 Section 6.4.1.3에서 정의된 기간 동안 임신이 된 것으로 추정되는 모든 임신은 증례기록서의 이상반응 페이지/section에 기록되어야 한다. 이는 여성 시험대상자와 남성 시험대상자의 여성 파트너 모두에게 해당한다. 시험자는 별도로 정해진 임신 보고 양식 (pregnancy report form)을 이용하여 신속하게 의뢰자에게 보고해야 하며, Section 6.4.1.4의 중대한 이상반응과 동일한 과정에 따라 진행되어야 한다.

시험자는 임신과 관련하여 설령 시험대상자가 중도 탈락되더라도 적극적으로 그 경과를 추적하고 기록하며 그 결과를 의뢰자에게 보고하여야 한다.

시험자는 임신 관련 결과에 대해 인지 후 24시간 이내에 임신 보고 양식 (pregnancy report form)을 이용하여 보고하여야 하며, 임신 관련 경과가 비정상일 경우로서 각각 시험대상자에게 비정상적 경과가 지속될 경우는 중대한 이상반응 보고 양식 (SAE report form)을 아동/태아에게 비정상적인 경과가 지속할 시에도 정해진 양식을 이용하여 보고하여야 한다.

모든 비정상적인 임신 관련 경과는 Section 6.3.1.4에서 정의하고 있는 신속한 절차에 따라 보고되어야 하며, 정상적인 임신과 관련된 경과는 출산 후 45일 이내의 가능한 한 이른 시점에 보고되어야 한다.

임상시험 기간 도중 임신을 하게 된 시험대상자는 임상시험용 의약품 투여가 중지되어야 한다. 의뢰자에게는 지체 없이 보고되어야 하며 시험대상자는 전술한 절차를 따라야 한다.

### 임상검사실 검사 평가

임상시험용 의약품을 기관 별로 전달하기 이전에 임상검사실 검사값에 대한 참고 범위 리스트가 의뢰자 또는 그 대리인에게 전달되어야 한다. 임상검사실 검사치의 참고 범위가 임상시험 도중 변경되게 되면, 변경된 참고 범위에 대한 정보는 의뢰자 또는 그 대리인에게 통보되어야 한다.

시험자는 임상검사실 검사를 위해 채취된 검체의 식별 관련 정보를 확인하여 오류가 발생하지 않도록 주의하여야 한다.

표 2 List of Safety Laboratory Tests

| **Hematology** |  | **Serum Chemistry** |
| --- | --- | --- |
| -Hemoglobin (Hgb) |  | -Alanine aminotransferase (ALT) |
| -Hematocrit (Hct) |  | -Aspartate aminotransferase (AST) |
| -RBC count |  | -Blood urea nitrogen (BUN) |
| -Platelet |  | -Creatinine |
| -WBC with differential count |  | -Glucose |
|  |  | -Total bilirubin |

### 활력징후, 신체 검사 및 기타 평가

스크리닝 방문과 시험 종료 방문에서 신체 전반에 걸친 신체 검진이 시행될 것이며, 활력징후는 혈압 및 맥박, 호흡수, 체온 측정을 포함한다. 측정된 신체 검진과 활력징후는 증례기록서에 기록될 것이다.

## 유효성 평가

### 일차 평가

본 임상시험에서는 일차 평가 항목으로서 Leeds Dyspepsia Questionnaire (LDQ)를 평가할 것이다.

LDQ는 8개의 위장 장애 증상에 대한 심각도 및 치료 반응에 대해 평가할 수 있도록 구성된 검증된 평가 척도이다. (25)

8개의 평가 대상 증상은 ① indigestion (a pain in the upper abdomen), ② heartburn (a burning feeling behind the breast bone), ③ sticks of food or drink behind the breast bone, ④ regurgitation (an acid taste coming up into the mouth from the stomach), ⑤ burping or belching, ⑥ nausea, ⑦ vomiting, ⑧ excessive fullness로서, 이들 증상에 대해 very mild, mild, moderate, severe, very severe의 5단계로 구분되어 답변하도록 되어 있는 likert scale 평가법이다.

### 이차 평가

본 임상시험의 이차 평가 항목으로서, GSRS (Gastrointestinal Symptom Rating Scale), 통증 VAS, EQ-5D 등이 사용될 계획이다.

GSRS의 경우, 위식도 역류 질환이나 기능성 소화 불량, 과민성 장 증후군에 대해 높은 상관성을 지닌 환자의 자기 기입식 설문으로 총 15개의 설문으로 구성된 평가법이다. (26,27,28)

시험대상자의 골관절염 관련 통증에 대해서는 VAS (visual analogue scale)를 사용할 것이다. VAS는 골관절염 환자에서의 통증 연구에서도 광범위하게 사용되는 간편한 평가법으로 설문을 이용한 likert scale 척도와 연관성이 높은 검증된 평가법이다. (29)

EQ-5D는 가장 보편적으로 사용되는 삶의 질 및 건강 수준 측정 도구 중 하나로서 삶의 영역 중 이동성 (mobility), 자기관리 (self-care), 일상생활 (usual activities), 통증/불편감 (pain/discomfort), 불안/우울 (anxiety/depression) 다섯 개 영역으로 “전혀 문제 없음”, “다소 문제 없음”, “많이 문제 있음”의 세 단계 중 하나로 건강 수준을 평가하는 객관식 문항과 1개의 온도계 형태의 주관적 건강 수준을 표시하는 visual analogue scale로 구성되어 있는 측정 도구이다.

또한 본 임상시험에서는 소화기계 이상반응 발생률, 소화기계 이상반응 발생으로 인한 임상시험 중단률을 등을 함께 평가할 예정이다.

시험약과 대조약에 의한 통증에 대한 조절 여부를 측정하기 위한 보조적인 방법 중 하나로서, 8주간 시험약 경구 투여 후 보충 및 구제약물 사용 여부, 사용한 평균 일수, 하루 당 평균 사용량 (총 사용량/총 사용 일수) 및 임상시험 참여기간 중 평균 사용량 (총 사용량/임상시험 참여 기간) 등을 평가할 것이다.

본 임상시험에서는 복용 순응도도 평가될 것이다.

복용 순응도는 ‘(실제 복용한 횟수)/(복용 예정 횟수)*100’으로 정의되며, 80% 이상 투여한 경우 PP 군 분석에 포함한다.

## 기타 평가

약물 유전체학 연구 등은 포함되지 않는다.

# 통계분석 계획

## 시험대상자 수 산정

**목표 시험대상자 수**: 106명

본 임상시험의 시험약과 대조약의 투여군 별 목표 시험대상자는 42이다. 중도 탈락률 20%를 고려할 때, 본 임상시험에서는 투여군 별로 53명, 총 106명의 시험대상자를 모집할 예정이다.

**근거:**

본 임상시험의 목적은 골관절염 환자를 대상으로 임상시험용 의약품 12주 경구 투여 후 LDQ (leeds dyspepsia questionnaire)의 기저치 대비 평균 변화량으로 평가되는 위장 장애 보호 효과에 대하여 시험약인 낙소졸 정이 대조약인 Celecoxib에 비해 임상적으로 비열등하다는 것을 보이기 위함이다.

소화기계 질환의 증상 및 개선 효과를 평가하고자 하는 선행 연구 등에 따르면 LDQ (leeds dyspepsia questionnaire)와 같이Likert scale로 구성된 설문을 통해 환자의 증상을 평가하는 경우, 개선도에 관한 ‘임상적으로 중요한 최소 차이’ (MCID, minimal clinically important difference)를 0.40로 제시하고 있는 바, (30,31,32) 본 연구의 비열등성 마진을 0.4로 설정하였다. LDQ (leeds dyspepsia questionnaire) 문항의 경우 5단계 Likert scale로 이루어져 있다.

Holtmann et al. 에 의해 수행된 Itopride의 functional dyspepsia 개선 효과에 대한 연구에 따르면 LDQ (Leeds dyspepsia questionnaire) 평균 변화량의 표준편차는 0.63 ~ 0.65으로, 보수적인 접근을 위해 σ = 0.65로 가정하였다. (22)

검정력 80% 그리고 단측 검정 유의수준 ($\alpha)$ 0.025 조건 하에서 아래 표본수 도출 공식을 이용하여 산출된 투여군 당 시험대상자 수는 42명이다. 중도 탈락율 20%를 고려하여 각 투여군 당 53명씩, 총 106명 이상의 시험대상자를 모집할 예정이다.

$$n_{시험군}= n_{대조군}= \frac{\left[ 2\sigma^{2} \right]\left[ {Z_{\alpha}+Z}_{\beta} \right]^{2}}{\delta^{2}}$$

$$= \frac{\left[ 2{*0.65}^{2} \right]\left[ 1.96+0.842 \right]^{2}}{{0.4}^{2}} \cong42$$

σ= LDQ 변화량의 표준편차

δ = 비열등성 마진

## 무작위 배정

**스크리닝 번호**

모든 시험대상자는 스크리닝 시에 무작위 배정과 상관없이 각각 고유의 스크리닝 번호를 부여받게 된다.

**무작위 배정 코드 생성**

시험약에 대한 안전성과 유효성을 평가하기 위한 시험대상자에 대해 무작위 배정 코드를 의뢰자 또는 의뢰자의 업무를 위임 받은 무작위 배정 코드 생성 담당자가 생성한다.

무작위 배정은 임상시험 시작 전에 실시한다. 각 임상시험 실시기관 별 Permuted Block Randomization Method를 적용한다.

각 투여군으로의 배정은 이중 눈가림 배정이므로, 시험자와 시험대상자는 어느 투여군에 배정되었는지 알 수 없다.

시험약과 대조약의 배정 비율은 1:1이다.

**시험대상자의 배정 및 무작위 배정 코드 유지**

선정/제외 기준에 합당한 시험대상자는 시험에 등록되는 순서대로 무작위 번호를 배정받는다. 각 시험대상자는 본인의 해당 무작위 배정 번호에 따라 투여군이 결정되어 임상시험용 의약품을 투여받는다.

시험 종료 이전에 시험자와 시험대상자는 시험약을 투여 받았는지, 대조약을 투여 받았는지 알 수 없다. 시험 종료 후에는 결과 분석을 위하여 의뢰자 측에서 시험자에게 코드를 공개하게 된다.

## 평가 변수

### 일차 평가 변수

- 임상시험용 의약품 12주 경구 투여 후 LDQ (leeds dyspepsia questionnaire)의 기저치 대비 평균 변화량

추가적으로, 임상시험용 의약품 투여 전 LDQ (leeds dyspepsia questionnaire)의 총점을 세 집단 (No/Very Mild: 0-8 점, Mild/Moderate: 9-24점, Severe/Very Severe: 25-40점)으로 구분하여 각 등급 별 LDQ (leeds dyspepsia questionnaire)의 기저치 대비 평균 변화량

### 이차 평가 변수

- 임상시험용 의약품 12주 경구 투여 후 LDQ (leeds dyspepsia questionnaire)의 평균
- 임상시험용 의약품 12주 경구 투여 후 GSRS (gastrointestinal symptom rating scale)의 기저치 대비 평균 변화량
- 임상시험용 의약품 12주 경구 투여 후 GI 부작용 (dyspepsia, diarrhoea, nausea, abdominal pain, heartburn) 발생률
- 임상시험용 의약품 12주 경구 투여 후 GI 부작용 (dyspepsia, diarrhoea, nausea, abdominal pain, heartburn)으로 인한 약물 중단률
- 임상시험용 의약품 12주 경구 투여 후 통증 VAS (visual analogue scale)의 기저치 대비 평균 변화량
- 임상시험용 의약품 12주 경구 투여 후 EQ-5D의 기저치 대비 평균 변화량
- 임상시험용 의약품 12주 경구 투여 후 복용 순응도
- 임상시험용 의약품 12주 경구 투여 후 보충 및 구제약물의 사용 여부, 사용한 평균 일수, 하루 당 평균 사용량 (총 사용량/총 사용 일수) 및 임상시험 참여 기간 중 평균 사용량 (총 사용량/임상시험 참여 기간)
- 임상시험용 의약품 12주 경구 투여 후 이상반응 발생 여부 및 빈도와 특성 (양상, 중대성, 결과 등)
- 임상시험용 의약품 12주 경구 투여 후 신체 검진, 활력징후, 임상검사실 검사 소견

### 기타 평가 변수

해당 사항 없음

## 분석군의 정의

**목표집단**

본 임상시험은 임상시험 기간 동안 일상적인 신체 활동을 할 수 있고 통증 VAS 검사 상 40mm 이상인 만 50세 이상의 골관절염 환자를 대상으로 할 것이다.

**안전성 평가군:** 무작위 배정 후 임상시험용 의약품을 투여 받고 한차례라도 안전성 관련 추적 관찰이 이루어진 시험대상자 집단을 의미한다.

**FA 평가군:** 무작위 배정 후 임상시험용 의약품을 투여 받고 유효성 평가가 한 차례라도 이루어진 시험대상자 집단을 의미한다.

**PP 평가군:** 무작위 배정 후 임상시험용 의약품을 투여 받고 유효성 평가가 한 차례라도 이루어진 시험대상자 집단 중 중대한 계획서 위반 (선정/제외 기준 위반, 복용 순응도 위반 등)이 없는 집단을 의미한다. 복용 순응도는 80%를 기준으로 한다.

## 통계 분석 계획

### 일반적 고려 사항

본 임상시험의 시험대상자로부터 얻어진 유효성 자료는FA 군과 PP 군 2가지 형태로 분석하고 안전성 자료는 Safety 군을 대상으로 분석한다.

일차 유효성 평가 변수에 대한 자료는 비열등성 시험임을 감안하여, ICH가이드라인에 근거하여 FA 군과 PP 군 모두를 주 분석군으로 한다.

일차 유효성 평가 변수를 제외한 모든 통계 검정은 유의수준 5%하에서 양측검정을 실시한다.

평가 항목에서 결측이 발생한 경우 multiple imputation 방법을 적용하여 결측치를 대치한다.

중간 분석은 실시하지 않는다.

병용약물은 ATC 용어로 기록하며, 이상반응 (AEs)과 병력은 Medical Dictionary (MedDRA 또는 WHOART)를 이용하여 SOC (system organ class) 및 PT (preferred term)로 표준화한다.

### 일차 평가 변수 분석

- 임상시험용 의약품 12주 경구 투여 후 LDQ (leeds dyspepsia questionnaire)의 기저치 대비 평균 변화량:

임상시험용 의약품 투여 전 대비 12주 투여 후 LDQ 변화량에 대하여 기술통계량 (시험대상자 수, 평균, 표준편차, 중앙값, 최소값, 최대값)을 투여군 별로 제시한다. 시험약의 비열등성을 증명하기 위해 LDQ 변화량의 투여군 차이값에 대한 97.5% 단측 검정 신뢰구간 (one-sided 97.5% confidence interval)을 산출한다. 이 신뢰구간의 상한선 (97.5% upper confidence limit)이 미리 명시한 비열등성 마진인 0.40 보다 작으면 귀무 가설을 기각하게 되어 비열등성이 증명된다.

또한 임상시험용 의약품 투여 전 LDQ (leeds dyspepsia questionnaire) 총점을 세 집단 (No/Very Mild: 0-8 점, Mild/Moderate: 9-24점, Severe/Very Severe: 25-40점)으로 구분하여 추가적인 분석을 시행한다.

### 이차 평가 변수 분석

- 임상시험용 의약품 12주 경구 투여 후 LDQ (leeds dyspepsia questionnaire)의 평균:

임상시험용 의약품 12주 투여 후 측정된 LDQ에 대하여 기술통계량 (시험대상자 수, 평균, 표준편차, 중앙값, 최소값, 최대값)을 투여군 별로 제시하고, 투여군 간 차이는 independent two-sample t-test 또는 Wilcoxon rank sum test를 이용하여 유의성 검정을 한다.

- 임상시험용 의약품 12주 경구 투여 후 GSRS (gastrointestinal symptom rating scale)의 기저치 대비 평균 변화량:

임상시험용 의약품 투여 전과 12주 투여 후 측정된 GSRS 그리고 투여 전 대비 12주 투여 후 GSRS 변화량에 대하여 기술통계량 (시험대상자 수, 평균, 표준편차, 중앙값, 최소값, 최대값)을 투여군 별로 제시한다. 각 투여군 내 변화량은 paired t-test 또는 Wilcoxon signed rank test 그리고 각 시점 별 그리고 변화량에 대한 투여군 간 차이는 independent two-sample t-test 또는 Wilcoxon rank sum test를 이용하여 유의성 검정을 한다.

- 임상시험용 의약품 12주 경구 투여 후 GI 부작용 (dyspepsia, diarrhoea, nausea, abdominal pain, heartburn) 발생율:

임상시험용 의약품 12주 투여 기간 동안 GI 부작용을 경험한 시험대상자 수, 비율 그리고 발생 빈도를 투여군 별로 제시한다. 투여군 간 비율 차이는 Chi-square test 또는 Fisher’s exact test를 이용하여 유의성을 검정한다.

- 임상시험용 의약품 12주 경구 투여 후 GI 부작용 (dyspepsia, diarrhoea, nausea, abdominal pain, heartburn)으로 인한 약물 중단률:

임상시험용 의약품 12주 투여 기간 동안 발생한 GI 부작용으로 약물을 중단한 시험대상자 수와 비율을 투여군 별로 제시한다. 투여군 간 비율 차이는 Chi-square test 또는 Fisher’s exact test를 이용하여 유의성을 검정한다.

- 임상시험용 의약품 12주 투여 후 통증 VAS (visual analogue scale)의 평균 변화량:

임상시험용 의약품 투여 전과 12주 투여 후 측정된 통증 VAS 그리고 투여 전 대비 12주 투여 후 통증 VAS 변화량에 대하여 기술통계량 (시험대상자 수, 평균, 표준편차, 중앙값, 최소값, 최대값)을 투여군 별로 제시한다. 각 투여군 내 변화량은 paired t-test 또는 Wilcoxon signed rank test 그리고 각 시점 별 그리고 변화량에 대한 투여군 간 차이는 independent two-sample t-test 또는 Wilcoxon rank sum test를 이용하여 유의성 검정을 한다.

- 임상시험용 의약품 12주 경구 투여 후 EQ-5D 평균 점수의 기저치 대비 평균 변화량:

임상시험용 의약품 투여 전과 12주 투여 후 측정된 EQ-5D 그리고 투여 전 대비 12주 투여 후 EQ-5D 변화량에 대하여 기술통계량 (시험대상자 수, 평균, 표준편차, 중앙값, 최소값, 최대값)을 투여군 별로 제시한다. 각 투여군 내 변화량은 paired t-test 또는 Wilcoxon signed rank test 그리고 각 시점 별 그리고 변화량에 대한 투여군 간 차이는 independent two-sample t-test 또는 Wilcoxon rank sum test를 이용하여 유의성 검정을 한다.

- 임상시험용 의약품 12주 경구 투여 후 복용 순응도:

임상시험용 의약품 12주 투여 기간 동안의 복용 순응도는 ‘(실제 복용한 횟수)/(복용 예정 횟수)*100’으로 정의되며, 투여군 간 기술통계량 (시험대상자 수, 평균, 표준편차, 중앙값, 최소값, 최대값)을 제시한다. 복용 순응도에 대한 투여군 간 차이는 independent two-sample t-test 또는 Wilcoxon rank sum test를 이용하여 유의성 검정을 한다.

실제 복용한 횟수는 시험대상자가 반환한 임상시험용 의약품의 수를 기반으로 결정한다. 단 분실 손상 등 원인이 분명한 경우는 이를 반영한다.

- 임상시험용 의약품 12주 경구 투여 후 보충 및 구제약물의 사용 여부, 사용한 평균 일수, 하루 당 평균 사용량 (총 사용량/총 사용 일수) 및 임상시험 참여 기간 중 평균 사용량 (총 사용량/임상시험 참여 기간):

임상시험용 의약품 12주 투여 기간 동안 보충 및 구제약물을 사용한 시험대상자 수 및 비율을 투여군 별로 제시하고, Chi-square test 또는 Fisher’s exact test를 이용하여 투여군 간 비율 차이에 대한 유의성을 검정한다.

또한 임상시험용 의약품 12주 투여 기간 동안 보충 및 구제약물을 사용한 평균 일수, 하루 당 평균 사용량 (총 사용량/총 사용 일수) 및 임상시험 참여 기간 중 평균 사용량 (총 사용량/임상시험 참여 기간)에 대하여 기술통계량 (시험대상자 수, 평균, 표준편차, 중앙값, 최소값, 최대값)을 투여군 별로 제시한다. 투여군 간 평균 차이에 대한 비교는 independent two-sample t-test 또는 Wilcoxon rank sum test를 이용하여 유의성 검정을 한다.

- 임상시험용 의약품 12주 경구 투여 후 이상반응 발생 여부 및 빈도와 특성 (양상, 중대성, 결과 등):

임상시험용 의약품 12주 투여 기간 동안 발생한 전체, 중대한, 그리고 중도 탈락의 원인이 된 이상반응과 임상시험용 의약품과 관련 있는 전체, 중대한 그리고 중도 탈락의 원인이 된 이상반응을 경험한 시험대상자 수와 비율을 투여군 별로 제시하고, Chi-square test 또는 Fisher’s exact test를 이용하여 투여군 간 비율 차이에 대한 유의성을 검정한다. 또한 전체 그리고 임상시험용 의약품과 관련있는 이상반응 그리고 이들의 중증도, 중대성, 취해진 조치 및 결과 등에 대해 발생 건수를 제시한다.

MedDRA 또는 WHOART의 SOC와 PT로 전체 그리고 임상시험용 의약품과 관련있는 이상반응에 대해 시험대상자 수, 비율 및 발생 건수를 투여군 별로 제시한다.

- 신체 검진:

신체 검사는 임상시험용 의약품 투여 전과 12주 투여 후 정상/비정상에 대해 빈도와 비율을 포함한 분할표를 제시하고, 각 투여군 내 변화의 유의성은 McNemar’s test 또는 McNemar’s exact test 그리고 투여군 간 변화 차이는 GEE (Generalized Estimating Equation)을 이용하여 유의성 검정을 한다.

- 활력징후:

활력징후는 임상시험용 의약품 투여 전과 12주 투여 후 그리고 투여 전 대비 12주 투여 후 대비 변화량에 대한 기술통계량 (시험대상자 수, 평균, 표준편차, 중앙값, 최소값, 최대값)을 제시한다. 각 투여군 내 변화량은 paired t-test 또는 Wilcoxon signed rank test 그리고 각 시점 별 그리고 변화량에 대한 투여군 간 차이는 independent two-sample t-test 또는 Wilcoxon rank sum test를 이용하여 유의성 검정을 한다.

- 실험실적 검사

실험실적 검사는 임상시험용 의약품 투여 전과 12주 투여 후 정상/비정상에 대한 빈도와 비율을 포함한 분할표를 제시하고, 각 투여군 내 변화는 McNemar’s test 또는 McNemar’s exact test 그리고 투여군 간 변화 차이는 GEE (Generalized Estimating Equation)을 이용하여 유의성 검정을 한다.

또한 혈액학 및 혈액화학 검사와 같은 연속형 자료의 경우 임상시험용 의약품 투여 전과 12주 투여 후 그리고 투여 전 대비 12주 투여 후 변화량에 대한 기술통계량 (시험대상자 수, 평균, 표준편차, 중앙값, 최소값, 최대값)을 제시한다. 각 투여군 내 변화량은 paired t-test 또는 Wilcoxon signed rank test 그리고 각 시점 별 그리고 변화량에 대한 투여군 간의 차이는 independent two-sample t-test 또는 Wilcoxon rank sum test를 이용하여 유의성 검정을 한다.

### 인구학적 정보

인구학적 그리고 임상 병력을 포함한 기타 기저 자료에 대한 평가는 안전성 평가군을 대상으로 하며, 연속형 자료일 경우는 시험대상자 수, 평균, 표준편차, 중앙값, 최소값, 최대값, 범주형 자료의 경우는 빈도와 비율을 제시한다. 투여군 간 비교를 위해서 연속형 자료에 대해서는 independent two-sample t-test 또는 Wilcoxon rank sum test, 범주형 자료에 대해서는 Chi-square test 또는 Fisher’s exact test를 이용하여 유의성을 검정한다.

## 중간 분석

중간 분석은 실시하지 않는다.

# 임상시험의 윤리적 법률적 측면

## 시험자의 책임

시험자의 책임하에 본인이 소속된 임상시험 실시기관에서 임상시험을 실시하도록 한다. 시험자는 임상시험 계획서와 헬싱키 선언에 따른 윤리적 원칙, ICH의 GCP 규정 (ICH Topic E6, 1996) 및 국내 관련 법규에 따라 임상시험이 수행되도록 한다. 시험자는 특히 동의를 취득한 시험대상자만을 임상시험에 포함시켜야 한다.

## 시험대상자 정보 및 동의서

임상시험 참여에 대한 서면 동의는 시험대상자가 임상시험에 참여하기 위한 무조건적이고 절대적인 선제 조건이다. 임상시험 관련 절차가 수행되기 이전에 시험대상자 부모나 법적 보호자의 서면 동의나 시험대상자 본인의 서면 동의가 선행되어야 한다.

시험대상자 동의가 획득되기 이전에 시험자는 시험대상자 또는 법적 대리인에게 적절한 정보를 제공하여야 한다. 시험대상자 동의를 얻기 위한 목적으로 사용되는 시험대상자 설명문은 자국어로 작성되고 ICH의 GCP (ICH Topic E6, 1996)에 준거하도록 의뢰자가 제작하여 준비해야 한다. 서면으로 작성된 시험대상자 설명문과 더불어 시험자는 임상시험의 모든 관련 사항에 대하여 구두로 설명하여야 한다. 일반인도 쉽고 완전히 이해할 수 있도록 평이한 언어로 설명하여야 한다.

시험자가 설명을 한 후 시험자와 시험대상자는 각각 별도의 서명일과 함께 시험대상자 동의서에 자필로 서명해야 한다.

서명과 서명일이 기재된 시험대상자 동의서는 시험자의 임상시험 실시기관에서 보존되어야 하며, 모니터링, 점검, 실사에 대비하여 언제든 열람될 수 있도록 시험자는 안전하게 보관하여야 한다. 임상시험 참여 전 서명과 서명일이 기재된 동의서의 사본 및 시험대상자에게 제공된 설명문 등의 사본은 시험대상자에게 제공되어야 한다

시험대상자의 동의에 영향을 줄 수 있는 중요하고 새로운 정보가 생기면 의뢰자는 시험대상자 동의서와 시험대상자 설명문을 갱신하여야 하며 임상시험 심사위원회에 다시 제출하여 검토와 승인을 받아야 한다. 승인을 받은 후 갱신된 정보는 각 시험대상자에게 제공되어 서명일 기재가 함께 된 서명을 획득하여야 한다. 시험자는 이전 version에서 변경된 내용에 대하여 설명할 것이다.

## 시험대상자 식별과 비밀 유지

임상시험에 참여 시 동의서가 획득되면 각 시험대상자 별로 시험대상자의 고유 식별 번호가 부여될 것이다. 이 식별 번호는 임상시험 기간뿐만 아니라 데이터 베이스 처리 과정에서도 이용될 것이다.

임상시험 도중 수집된 시험대상자 정보 역시 이 번호를 이용하여 저장될 것이며, 시험자만이 임상시험 실시기관에 보관된 식별 목록을 통하여 시험대상자와 시험대상자의 임상시험 관련 정보간에 연관 짓는 것이 가능하다. 모니터 요원에 의한 근거 문서 검증 과정, 점검, 또는 보건당국의 실사를 위하여 의료 관련 원본의 정보를 검토하는 중 획득된 시험대상자의 비밀은 철저히 유지되어야 한다.

정보 보호와 사생활 보호 관련 법률은 시험대상자 개인 정보의 취득, 전달, 처리, 보관 전 과정에 걸쳐 관여하고 있다. 국내 법률에 의거한 정보 처리 과정에 대하여 시험대상자에게 분명히 알리고 동의들 얻어야 한다. 본 연구에서 수집된 시험대상자의 건강 정보를 포함한 민감 정보는 본 연구의 종료 후 3년까지 보관되고 이후 적법한 절차에 따라 폐기될 것이다.

## 시험대상자에 대한 보상 및 보험

본 임상시험에 참여하는 모든 시험대상자들은 국내 실정에 적합한 수준에서 보험으로 보호된다. 본 임상시험에 의한 피해가 발생할 경우를 대비한 피해 보상에 대한 규약은 IRB의 승인을 받을 것이다.

## 임상시험 심사위원회

임상시험을 해당 실시기관에서 개시하기 이전에 임상시험 계획서와 증례기록서, 시험대상자 설명문을 비롯하여 시험대상자에게 제공되는 모든 문서는 승인을 받기 위하여 임상시험 심사위원회에 제출되어야 한다.

임상시험 심사위원회의 서면 승인서는 실시기관의 시험자 파일 안에 보관되어야 하며, 의뢰자나 업무를 위임 받은 기관의 임상시험 기본 문서 파일 안에 복사본을 보관하여야 한다.

의뢰자가 임상시험 심사위원회로부터 승인을 얻기 전에는 임상시험이 개시되어서는 안 된다. 임상시험 심사위원회로부터 승인을 받으면, 승인을 받은 날짜와 참석한 위원 명단, 투표에 참여한 위원 명단이 수록된 문서를 전달 받아야 한다. 문서로 된 승인서에는 해당 임상시험이 명백히 언급되고, 임상시험계획서 version, 시험대상자 설명문과 동의서 version이 명시 되어 있어야 한다. 가능하면 회의록도 전달받아야 한다.

임상시험의 변경 역시 해당 임상시험 심사위원회에 제출되어 승인 후 변경내용을 임상시험에 반영하여야 한다 (Section 9.5 참조). 법규와 규정에 준거하여 해당되는 안전성 관련 정보는 임상시험 기간 도중 임상시험 심사위원회에 제출되어야 한다.

## 정부 기관

본 임상시험 계획서와 기타 해당 문서 (즉, 임상시험용 의약품 제품 문서, 시험대상자 설명문, 시험대상자 동의서 양식) 등은 국내 법률에 준거하여 관련 규제 기관에 제출되고 통보되어야 한다.

# 임상시험 관리

## 증례기록서 관리

증례기록서란 완벽하고 정확하며, 알아보기 쉽고 시간을 절약할 수 있는 형태로 임상시험 계획서 상 필요한 정보들을 수집하기 위한 것이다. 증례기록서에 있는 정보들과 해당 근거 문서상 정보들은 일치하여야 한다.

본 임상시험의 수행 중 수집된 정보들은 증례기록서와 해당 할 경우에는 이상반응 안전성 보고 양식 (adverse event safety report form)에 기재되어야 하며, 의뢰자나 의뢰자로부터 해당 업무를 위임 받은 자에게 전달되어야 한다. 전달된 정보들은 정보 보호 규정을 준수하며 무기명 상태로 처리, 평가, 보존될 것이다.

시험자는 증례기록서와 기타 관련 문서들이 의뢰자나 해당 업무를 위임 받은 자에게 무기명의 형태로 전달되었음을 확인하여야 한다.

증례기록서는 빠짐 없고 읽기 쉽게 기재되어야 하며, 공식 문서 기재용으로 사용될 수 있는 검정색이나 청색 볼펜을 이용하여 작성되어야 한다. 모든 변경이나 수정 사항은 시험자에 의해 시행되어야 하고 변경/수정된 날짜와 서명 (countersign)을 해야 한다. 오류는 읽을 수 있는 형태로 남겨져야 하며 수정액 등을 이용하여 수정해서는 안된다. 시험자는 반드시 중요 기재 사항이 수정된 사유를 기록에 남겨야 한다.

증례기록서에서 누락된 정보나 진술란의 경우 입력란은 불필요한 쿼리 발생을 줄이기 위하여 취소선을 그어야 한다.

증례기록서는 임상시험에 필수적인 문서로 정부기관의 실사 시 제출할 수 있도록 적합하게 관리되어야 한다.

## 근거 자료와 시험대상자 관련 파일

시험자는 임상시험에 참여한 모든 시험대상자에 대하여 종이 또는 전자 문서 상 작성된 시험대상자 관련 파일들 (의료 관련 파일, 원본 의료 기록)을 보존해야 한다. 이 파일들에는 시험대상자의 인구학적 자료와 의료기록 등이 포함되며, 가능한 한 완벽히 기록되어 있어야 한다. 특히 이들 파일에서는 본 임상시험과 관련하여 다음의 정보들을 파악할 수 있어야 한다.

- 시험대상자의 성명
- 출생일
- 성별
- 인구학적 정보
- 과거 및 현 병력
- 과거 및 현재의 투여 약물 (임상시험 기간 중 변화 포함)
- 임상시험 관련 식별 정보 (trial identification)
- 시험대상자가 임상시험에 참여한 날짜 (즉 서면 동의한 날)
- 임상시험 중 시험대상자 번호
- 시험대상자가 임상시험 실시기관을 방문한 날
- 임상시험 계획서 상에 미리 정의된 의학적 검사 또는 기타 임상적 소견
- 시험대상자에서 발생한 모든 이상반응
- 시험대상자의 임상시험 참여 종료일
- 해당되는 경우, 임상시험이나 임상시험용 의약품으로부터 탈락한 날짜와 이유

이 시험대상자 파일을 이용하여 각 시험대상자 별 개인 식별을 한다.

자동화된 기기를 통해 측정된 결과의 출력 자료, 영상 자료, 심전도 기록, 임상검사실 검사값 목록 등, 근거 자료를 포함하는 모든 문서들은 반드시 파일에 보존되어야 한다. 이들 문서들은 적어도 시험대상자 번호와 처치가 시행된 날짜에 대한 정보를 포함하여야 한다. 가능한 한 평가 또는 측정에 사용된 기기 자체에서 직접 출력된 자료이어야 한다. 자동화된 기기에서 출력할 수 없는 정보는 직접 기재한다. 필요할 경우에는 이들 정보 기록에 대하여 행한 의학적 평가 역시 기록되어야 하며, 시험자에 의한 서명과 날짜가 기재되어야 한다.

일부 정보들은 의료 관련 원본 파일이 아니라 증례기록서 (또는 설문, 일지 등)에 직접 기재될 수 있으며, 이 경우 시험대상자의 증례기록서에 기재된 정보에 상응하는 원본 파일 (종이 또는 전자 문서 상)이 존재하지 않는다. 이럴 경우에는 증례기록서에 기재된 정보가 근거 자료에 해당한다. 증례기록서에 기재된 정보가 근거 자료에 해당하는 경우에 대해서는 임상시험 계획서에 분명히 알기 쉽고 완벽하게 어떤 정보가 이에 해당하는지 기재하여야 한다. 증례기록서 상의 정보 중 명쾌하게 근거 자료로 명시되지 않는 경우에는 시험대상자 관련 의료 파일에 기재되어 있어야 한다.

## 시험자 파일과 자료의 보관

시험자는 임상시험이 개시되는 시점에서 시험자 파일 (investigator site file)을 제공 받는다. 이 파일은 임상시험 수행에 필요한 모든 문서들을 포함하고 있으며 임상시험이 종료될 때까지 지속적으로 갱신되어 항상 완전한 상태를 유지할 것이다. 이 파일은 모니터 요원이 검토할 수 있도록 접근 가능하여야 하며 의뢰자의 점검과 보건 당국의 실사에 대해 준비되어 있어야 한다. 의약품 등의 안전에 관한 규칙 30조 12항에 준거하여 임상시험 종료 후 3년간 보존하여야 한다. 보관 문서 중에는 시험대상자 식별 목록과 시험대상자 동의서가 포함된다. 만일 임상시험 실시기관에서 시험자 기관 파일의 보관이 불가능해지면 시험자는 의뢰자에게 그 사실을 반드시 통보하여야 한다.

시험자는 국내 법 및 법률을 준수하여 시험대상자의 개인정보를 보호할 것이다. 본 임상시험 참여 시 시험대상자의 개인 정보는 나이, 성별, 개인의 신원을 파악할 수 없게 코드화한 번호를 이용하여 익명으로 처리하고, 식별 가능한 자료는 접근을 제한하여 보관되어, 기록에 대한 비밀보장이 유지될 것이다. 임상시험 결과에 대한 자료는 연구 목적에 의해서만 익명으로 열람되고 연구되며 필요한 경우 출판될 수 있으며, 출판되더라도 시험대상자의 개인정보는 비밀로 유지된다.

모든 시험대상자 관련 원본 파일 (의료 기록)은 반드시 임상시험 실시기관 (병원, 연구기관 또는 의원)에 관련 법률이나 ICH 임상시험 관리 규정 (GCP)에서 규정하는 기간 중 더 긴 기간 동안 보존되어야 하며, 의뢰자가 서면 동의 하기 전에는 의료 기록이 파기되어서는 안 된다.

## 모니터링 품질 보증, 정부 기관의 실사

본 임상시험은 한국 식품의약품안전처와 ICH의 임상시험 관리 규정 (ICH topic E6, 1996와 KGCP)에 따라 모니터링 될 것이다. 각 기관을 담당하고 있는 모니터 요원은 정기적으로 임상시험 실시기관을 방문한다. 모니터링은 의뢰자에 의해 지정된 모니터 요원에 의해 시행될 것이다.

보건당국 이외에도 의뢰자 또는 위임 받은 자는 임상시험과 관련된 모든 문서들과 시험자 (연구자) 파일(investigator site file), 작성된 증례기록서, 임상시험용 의약품, 시험대상자의 의료기록/파일 등을 포함하여 실시기관의 기타 자료들을 실사할 수 있도록 허용되어야 한다.

임상시험 계획서, 각 단계별 정보 취득 과정, 정보의 취급 관리, 임상시험 결과 보고서가 관계법령을 준수하였는지 여부를 확인하는 품질 보증 (quality assurance)의 대상에 해당한다. 임상시험 관련 정보들의 타당성과 진실성을 확보하기 위하여 임상시험 도중이나 종료 후 임의의 시점에 점검 (audit)이 실시될 수 있다.

## 임상시험 계획서의 변경

임상시험 계획서 변경을 하게 되면 임상시험 변경에 관련된 문건 (변경 계획서)에 작성해야 한다. 주요 변경 (상당 부분 변경, 중요한 변경)의 경우 변경 승인을 받기 위해서 관계 당국과 해당 임상시험 심사위원회에 제출하여야 한다. 승인을 받은 후에만 주요 변경 내용이 반영되어 시행될 수 있다.

행정 관련 변경을 포함하여 사소한 계획서 변경은 의뢰자와 임상시험 실시기관에 기록이 보관될 것이며, 해당 임상시험 심사위원회나 보건당국에는 관련 규정에 따라 제출한다.

시험대상자의 동의 여부에 영향을 줄 수 있는 모든 변경 사항에 대해서는 변경 사항에 대하여 시험대상자로부터 동의를 받은 후에 적용하도록 한다 (Section 8.2 참조).

## 임상시험 보고와 자료 발표에 관한 원칙

### 임상시험 보고서

임상시험 종료 후 임상시험 책임자의 조언 등을 바탕으로ICH E3 지침에 맞추어 임상시험 결과 보고서를 작성한다.

### 자료의 발표

본 임상시험에 관한 최초의 발표는 일차 평가 변수 분석을 포함한 것이 될 것이며, 모든 임상시험 실시기관으로부터 수집된 정보를 포함할 것이다.

시험자 또는 시험자를 대표한 자는 본 임상시험에 관련된 데이터를 출판 또는 발표할 계획이 있을 경우 의뢰자에게 사전에 알려야 한다. 모든 출판 및 발표 자료들 (논문 초록, 신문 기사, 구두 발표 등)은 제출하기 전의 적절한 시기에 발표 자료의 전체 또는 일부를 보내어 의뢰자가 검토할 수 있도록 해야 한다.

의뢰자는 임상시험 발표에 대해 반대하거나 거부하지는 않을 것이나, 지적 재산권 보호를 위하거나 사업화 전략에 따라 발표시기를 연기할 수 있는 권리가 있다.

# References

x

| 1. | AstraZeneca. Vimovo prescribing Information. ; 2012. |
| --- | --- |
| 2. | NIH. Daily Med. current medication information. [Online]. [cited 2014 6. Available from: <http://dailymed.nlm.nih.gov/dailymed/lookup.cfm?setid=e2e18e10-6f02-4ccc-842d-1962a3838b74>. |
| 3. | Dugowson CE, Gnanashanmugam P. Nonsteroidal Anti-Inﬂammatory Drugs. In Phys Med Rehabil Clin N Am; 2006. p. 347–354. |
| 4. | Shin JM, Sachs G. Pharmacology of Proton Pump Inhibitors. In Curr Gastroenterol Rep; 2008. p. 528–534. |
| 5. | 한미약품 (주). 건강한 한국인 성인 남성 자원자에서 HCP1004 와 비모보정 500/20mg 의 안전성과 약동학적 특성을 비교하기 위한 공개형, 무작위 배정, 교차, 단회 투여 임상시험. 임상시험보고서 (Protocol No. HM-ESNP-102). ; 2013. |
| 6. | Bang CS, Baik GH. Complications and Management of Peptic Ulcer Disease. In The Korean Journal of Helicobacter and Upper Gastrointestinal Research; 2014. p. 18-23. |
| 7. | Ramakrishnan K, Salinas R. Peptic ulcer disease. In Am. Family Physician; 2007. p. 1005-1012. |
| 8. | Milosavljevic T, Kostić-Milosavljević M, Jovanović I, Krstić M. Complications of peptic ulcer disease. In Digestive Diseases; 2011. p. 491-493. |
| 9. | Martinez J, Mattu A. Abdominal pain in the elderly. In Emergency Medicine Clin. North Am.; 2006. p. 371-388. |
| 10. | Lanza F, Chan FKL, Quigley EMM, Gast. PPCoACo. Guidelines for Prevention of NSAID-Related Ulcer Complications. In Am J Gastroenterol; 2009. p. 728 – 738. |
| 11. | Patrignani P, Tacconelli S, Bruno A, Sostres C, Lanas A. Managing the Adverse Effects of Nonsteroidal Anti-inflammatory Drugs. In Expert Review of Clinical Pharmacology; 2011. p. 605-621. |
| 12. | Lanas A, Hirschowitz B. Toxicity of NSAIDs in the stomach and duodenum. In Eur. J. Gastroenterol. Hepatol.; 1999. p. 375–381. |
| 13. | Hawkey C, Karrasch J, Szczepanski L, al. e. Omeprazole compared with misoprostol for ulcers associated with nonsteroidal antiinflammatory drugs. Omeprazole versus Misoprostol for NSAID-Induced Ulcer Management. In N. Engl. J. Med.; 1998. p. 727–734. |
| 14. | Yeomans N, Tulassay Z, Juhasz L, al. e. A comparison of omeprazole with ranitidine for ulcers associated with nonsteroidal antiinflammatory drugs. Acid Suppression Trial: Ranitidine versus Omeprazole for NSAID-associated Ulcer Treatment (ASTRONAUT) study group. In N. Engl. J. Med. 338; 1998. p. 719–726. |
| 15. | Yeomans N, Lanas A, Talley N, al. e. Prevalence and incidence of gastroduodenal ulcers during treatment with vascular protective doses of aspirin. In Aliment Pharmacol. Ther.; 2005. p. 795–801. |
| 16. | Watson D, Harper S, Zhao P, al. e. Gastrointestinal tolerability of the selective cyclooxygenase-2 (COX-2) inhibitor rofecoxib compared with nonselective COX-1 and COX-2 inhibitors in osteoarthritis. In Arch. Intern. Med.; 2000. p. 2998–3003. |
| 17. | Moore R, Derry S, Makinson G, McQuay H. Tolerability and adverse events in clinical trials of celecoxib in osteoarthritis and rheumatoid arthritis: systematic review and meta-analysis of information from company clinical trial reports. In Arthritis Res. Ther.; 2005. p. R644–R665. |
| 18. | Hawkey C, Jones R, Yeomans N, al. e. Efficacy of esomeprazole for resolution of symptoms of heartburn and acid regurgitation in continuous users of non-steroidal anti-inflammatory drugs. In Aliment Pharmacol. Ther; 2007. p. 813–821. |
| 19. | Scheiman J, Yeomans N, Talley N, al. e. Prevention of ulcers by esomeprazole in at-risk patients using non-selective NSAIDs and COX-2 inhibitors. In Am. J. Gastroenterol.; 2006. p. 701–710. |
| 20. | Roberts DN, Miner PB. Safety aspects and rational use of a naproxen + esomeprazole combination in the treatment of rheumatoid disease. In Drug, Healthcare and Patient Safety; 2011. p. 1–8. |
| 21. | Sostek M, Fort J, Estborn L, Vikman K. Long-term safety of naproxen and esomeprazole magnesium fixed-dose combination: phase III study in patients at risk for NSAID-associated gastric ulcers. In Curr Med Res Opin; 2011. p. 847-854. |
| 22. | Holtmann G, Talley NJ, Liebregts T, Adam B, Parow C. A Placebo-Controlled Trial of Itopride in Functional Dyspepsia. In N Engl J Med; 2006. p. 832-840. |
| 23. | Rabeneck L, Goldstein J, Vu A, Mayne T, Rublee D. Valdecoxib is associated with improved dyspepsia-related health compared with nonspecific NSAIDs in patients with osteoarthritis or rheumatoid arthritis. In Am J Gastroenterol.; 2005. p. 1043-1050. |
| 24. | INTERNATIONAL CONFERENCE ON HARMONISATION OF TECHNICAL REQUIREMENTS FOR REGISTRATION OF PHARMACEUTICALS FOR HUMAN USE. CHOICE OF CONTROL GROUP AND RELATED ISSUES IN CLINICAL TRIALS (E10). ICH; 2000. |
| 25. | Moayyedi P, Duffett S, Braunholtz D, Mason S, Richards DG, Dowell AC, et al. The Leeds Dyspepsia Questionnaire: a valid tool for measuring the presence and severity of dyspepsia. In Alimentary Pharmacol. Ther.; 1998. p. 1257-1262. |
| 26. | Svedlund J, Sjodin I, Dotevall G. GSRS-a clinical ration scale for gastrointestinal symptoms in patients with irritable bowel syndrome and peptic ulcer disease. In Dig Dis Sci.; 1988. p. 129-134. |
| 27. | Revicki D, Wood M, Wiklund I, al. e. Reliability and validity of the Gastrointestinal Symptom Rating Scale in patients with gastroesophareal reflux disease. In Qual Life Res; 1998. p. 75-83. |
| 28. | Mones J, Adan A, Segu J, Lopez J, Artes M, Guerrero T. Quality of life in funtional dyspepsia. In Dig. Dis. Sci.; 2002. p. 20-26. |
| 29. | Bolognese J, Schnitzer T, Ehrich E. Response relationship of VAS and Likert scales in osteoarthritis efficacy measurement. In Osteoarthritis Cartilage; 2003. p. 499-507. |
| 30. | Hawkey , Talley NJ, Yeomans ND, Jones , Sung JJ, Långström , et al. Improvements with Esomeprazole in Patients with Upper Gastrointestinal Symptoms Taking Non-Steroidal Antiinflammatory Drugs, Including Selective COX-2 InhibitorsImprovements with Esomeprazole in Patients with Upper Gastrointestinal Symptoms. In Am J Gastroenterol; 2005. p. 1028-1036. |
| 31. | Junghard O, Wiklund I. What is a clinical relevant difference in patient-reported outcomes in the treatment of reflex disease? In Clinical Ther; 2003. p. d42-d45. |
| 32. | Tally N, Fullerton S, Junghard O, Wiklund I. Quality of life in patients with endoscopy-negative heartburn: reliability and sensitivity of disease-specific instruments. In AJG; 2001. p. 1998-2004. |

x

x

x

# Appendices 부록

### Appendix 1. 임상시험 진행 일정표

| 일정 | Screening & Washout period | | Treatment & Follow-up Period* | |
| --- | --- | --- | --- | --- |
|  | Screening^1^ | Randomization | | End of Sudy |
| Weeks | -2~0 | 0 | | 12 |
| 방문일 | -14~1 | 1 | | 84±7 |
| 방문  항목 | V1 | V2 | | V3 |
| 동의서 취득 |  |  | |  |
| 스크리닝 번호 부여 |  |  | |  |
| 선정/제외 기준 |  |  | |  |
| 인구학적 조사/병력/약물투여력 |  |  | |  |
| 신체 검진^2^ | (체중 포함) |  | |  |
| 활력징후^3^ |  |  | |  |
| X-ray (병변부위)^4^ |  |  | |  |
| 통증 VAS^5^ |  |  | |  |
| 무작위 배정 |  |  | |  |
| 채혈 일정 |  |  | |  |
| 혈액화학 검사**^6^** |  |  | |  |
| 혈액학 검사**^7^** |  |  | |  |
| 임신반응 검사**^8^** |  |  | |  |
| 약물투여 관련 사항 |  |  | |  |
| 시험약/보충약/구제약 교부 |  |  | |  |
| 시험약/보충약/구제약 회수 |  |  | |  |
| LDQ, GSRS, EQ-5D |  |  | |  |
| 시험대상자 일지 교부 |  |  | |  |
| 시험대상자 일지 회수 |  |  | |  |
| 병용약물 확인 |  |  | |  |
| 이상반응 모니터링 |  |  | |  |

1. Washout이 별도로 필요하지 않은 경우 Day 1당일에 스크리닝과 무작위배정을 실시할 수 있다.
2. 키(문진), 체중(실측치). 체중은 방문 1에서만 측정.
3. 혈압, 맥박 수, 호흡수
4. 6개월 이내 병변 부위 X-ray 결과가 있을 경우 미시행.
5. 방문 1에서는 시행 안하며 방문 2에서 시행
6. ALT, AST, BUN, creatinine, Glucose, Total Bilirubin
7. CBC (hemoglobin, hematocrit, RBC count, , WBC with differential count, platelet count)
8. 가임 여성의 경우에만 시행

* **추가적 방문**: 이상반응의 추적 관찰이 필요하면 추가적인 방문이 시행될 수 있다

### Appendix 2. 시험자 설문지

**(Leeds Dyspepsia Questionnaire, LDQ)**

| 1 | Over the last FOUR WEEKS have you had any **indigestion** (a pain in the upper abdomen) (see picture)?  YES [ ] NO [ ]  *If the answer is no please go to question 2.*  How severe has your indigestion been over the last FOUR WEEKS?  Very mild [ ]  Mild [ ]  Moderate [ ]  Severe [ ]  Very severe [ ] | 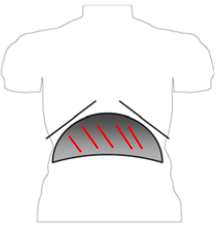 |
| --- | --- | --- |
|  |  | epigastric pain or discomfort,  상복부 통증, 불편감, 쓰림, 작열감.  * 2번 문항과 위치가 다름을 주의! |
| 2 | Over the past FOUR WEEKS have you ever experienced heartburn (a burning feeling behind the breast bone) (see picture)?  YES [ ] NO [ ]  *If the answer is no please go to question 3.*  How severe has your heartburn been over the last FOUR WEEKS?  Very mild [ ]  Mild [ ]  Moderate [ ]  Severe [ ]  Very severe [ ] | 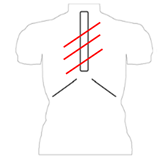 |
|  |  | 가슴 쓰림감, 흉부 작열감, 가슴 불편감,  가슴 통증 |
| 3 | Over the past FOUR WEEKS has **food or drink ever stuck behind your breast bone as it went down**?  YES [ ] NO [ ]  *If the answer is no please go to question 4.*  How severe has your symptom been over the last FOUR WEEKS?  Very mild [ ]  Mild [ ]  Moderate [ ]  Severe [ ]  Very severe [ ] | 연하 시 발생하는 흉부 불편감 및 통증 odynophagia (연하통)  ddx) dysphagia (연하곤란, 삼킴곤란) |
| 4 | Over the last FOUR WEEKS have you experienced any **regurgitation** (an acid taste coming up into your mouth from your stomach)?  YES [ ] NO [ ]  *If the answer is no please go to question 5.*  How severe has your regurgitation been over the last FOUR WEEKS?  Very mild [ ]  Mild [ ]  Moderate [ ]  Severe [ ]  Very severe [ ] | 위 내용물(gastric content)의 역류(reflux)  ddx) 트림(belching), 구토, 반추(rumination) |
| 5 | Over the last FOUR WEEKS have you noticed excessive **burping or belching**?  YES [ ] NO [ ]  *If the answer is no please go to question 6.*  How severe has your belching been over the last FOUR WEEKS?  Very mild [ ]  Mild [ ]  Moderate [ ]  Severe [ ]  Very severe [ ] | 트림, 가스만 입으로 배출 |
| 6 | Over the last FOUR WEEKS have you experienced any **nausea** (a feeling of sickness without actually being sick?)?  YES [ ] NO [ ]  *If the answer is no please go to question 7.*  How severe has your nausea been over the last FOUR WEEKS?  Very mild [ ]  Mild [ ]  Moderate [ ]  Severe [ ]  Very severe [ ] | 구역, 오심, 메스꺼움 |
| 7 | Over the last FOUR WEEKS have you experienced any **vomiting**?  YES [ ] NO [ ]  *If the answer is no please go to question 8.*  How severe has your vomiting been over the last FOUR WEEKS?  Very mild [ ]  Mild [ ]  Moderate [ ]  Severe [ ]  Very severe [ ] | 메스꺼움, 구역질(retching)을 동반함  ddx) 역류(reflux), 반추(rumination), 트림(belching) |
| 8 | Over the last FOUR WEEKS have you noticed an **excessive feeling of fullness after eating**?  YES [ ] NO [ ]  *If the answer is no please go to question 9.*  How severe has your fullness been over the last FOUR WEEKS?  Very mild [ ]  Mild [ ]  Moderate [ ]  Severe [ ]  Very severe [ ] | 식후 복부 팽만감, 헛배부름 |
| 9 | Which, if any, of these symptoms has been the most troublesome to you in the last FOUR WEEKS? TICK ONE BOX ONLY   1. Heartburn [ ] 2. Regurgitation [ ] 3. Indigestion [ ] 4. Belching [ ] 5. Nausea [ ] 6. Vomiting [ ] 7. Excessive fullness [ ] 8. None of these have troubled me [ ] |  |

### Appendix 3. 시험대상자 설문지

| **임상시험계획서 번호** | **Naxozol_P4_1** | |
| --- | --- | --- |
| **스크리닝 번호** | \|  \|  \|  \|  \|  \|  \| \| --- \| --- \| --- \| --- \| --- \| --- \| \| S \|  \| - \|  \|  \|  \| \|  \|  \|  \|  \|  \|  \| | |
| **무작위배정 번호** | \|  \|  \|  \|  \|  \|  \| \| --- \| --- \| --- \| --- \| --- \| --- \| \| **R** \|  \| - \|  \|  \|  \| \|  \|  \|  \|  \|  \|  \| | |
| **시험대상자 이니셜** | \|  \|  \|  \| \| --- \| --- \| --- \| \|  \|  \|  \| \|  \|  \|  \| | |
| **방문일** | **□ 방문2** | \|  \|  \|  \|  \|  \|  \| \| --- \| --- \| --- \| --- \| --- \| --- \| \| 년 \| 년 \| 월 \| 월 \| 일 \| 일 \| |
|  | **□ 방문3** |  |
| **시험담당자** |  | |

- 저희 임상시험에 참여해 주셔서 대단히 감사합니다.
- 작성해 주신 내용은 본 임상시험 이외의 목적으로는 전혀 사용되지 않습니다.
- 요령을 숙지 하시여 정확하고 신뢰할 수 있는 정보가 되도록 작성 부탁 드립니다.
- 작성 후에는 반드시 반환해 주셔야 합니다.
- 본 임상시험에 참여 해 주신 점 다시 한번 깊은 감사의 말씀을 드립니다.

| 시험번호 | 방문 | 스크리닝번호 | 무작위배정번호 | 이니셜 |
| --- | --- | --- | --- | --- |
|  |  |  |  |  |
| Naxozol_P4_1 |  | \| S \|  \|  \|  \| \| --- \| --- \| --- \| --- \| | \| R \|  \|  \|  \| \| --- \| --- \| --- \| --- \| | \|  \|  \|  \| \| --- \| --- \| --- \| |

**(통증 VAS)**

귀하의 통증 정도를 직선위에 선을 그어 표기해 주십시오 (VAS).

| **통증**  **없음** |  | **상상할 수 있는 가장 극심한 통증** |
| --- | --- | --- |
|  |  |  |

**(Gastrointestinal Symptom Rating Scale, GSRS)**

최근 3개월간, 해당하는 번호에 **∨** 표시하세요


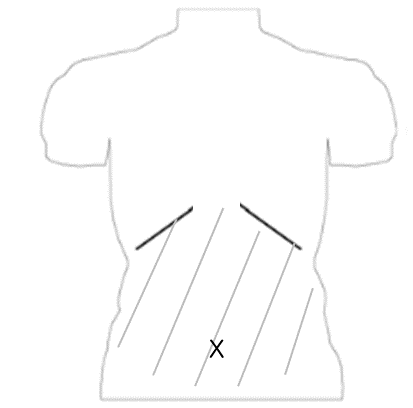


**1) 복 통 (abdominal pain)**

 가슴쪽(흉골하)이 아닌 복부 전반의 통증을 의미합니다.

⓪ 없거나 순간적인 통증

① 활동을 방해하는 수시로 나타나는 결림이나 통증

② 완화를 필요로 하고 많은 사회 활동을 방해하는 연장된 성가신 결림이나 통증

③ 모든 사회 활동에 영향을 주는 심하고 무력하게 하는 통증


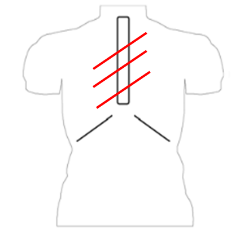


**2) 가슴쓰림 (heartburn)**

식도 부위(가슴뼈 뒤쪽)에 타는 듯한 느낌 및 통증을 의미합니다.

⓪ 없거나 순간적인 속쓰림

① 수시로 나타나는 짧은 동안의 불편

② 빈발하는 연장된 불편함의 상태; 완화의 요구

③ 단지 제산제에 의해 순간적으로 완화되는 지속적인 불편

**3) 산 역류(acid regurgitation)**

위장의 신물이 위장에서부터 가슴쪽으로 불쾌하게 올라오는 것을 의미합니다.

⓪ 없거나 순간적인 역류

① 가끔 나타나는 성가신 역류

② 하루에 1회내지 2회의 역류 ; 완화의 필요

③ 하루에 수차례 나타나는 역류 ; 제산제에 의해 단지 일시적이고 미약한 완화

**
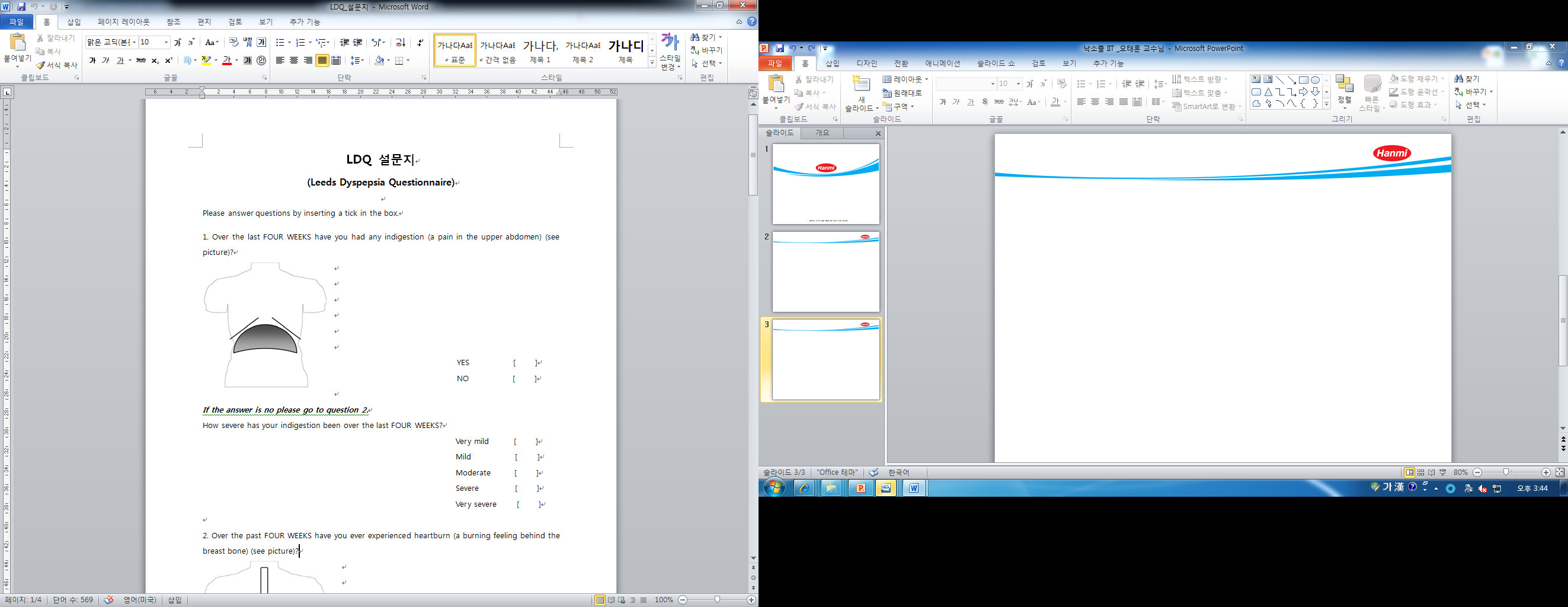
4) 상복부의 타는 느낌(sucking sensation in the epigastrium)**

윗배 중앙에 타는 듯한 느낌을 의미합니다.

⓪ 없거나 순간적인 핥는 느낌

① 수시로 나타나는 짧은 기간의 불편함; 식사 사이에 음식, 제산제가 필요하지 않음.

② 빈발하는 연장된 불편함의 상태; 식사 사이에 음식, 제산제를 필요로 함

③ 지속적인 불편 때문에 식사 사이에 음식, 제산제를 자주 필요로 함

**5) 오심과 구토(nausea and vomiting)**

오심이란 메스꺼움을 의미하며, 구토란 토하는 것을 의미합니다.

⓪ 오심 없음

① 수시로 나타나는 짧은 시간 동안의 상태

② 빈발하고 연장된 오심; 구토는 없음

③ 지속적인 오심; 빈발하는 구토

**6) 창자가스소리(Borborygmus)**

배에서 꾸럭꾸럭하는 소리가 나는 것을 의미합니다.

⓪ 없거나 순간적인 창자가스소리

① 수시로 나타나고 성가신 짧은 시간의 창자가스소리

② 사회적 일에 손상 없이 움직임에 의해 억제되는 빈발하고 연장된 창자가스소리

③ 사회적 일에 심각하게 방해하는 계속되는 창자가스소리

**7) 복부팽만(Abdominal distention)**

복부 전반에 팽만감 및 더부룩함, 헛배부름을 의미합니다.

⓪ 없거나 순간적인 팽창

① 수시로 나타나는 짧은 기간의 불편함

② 꼭 맞는 의류에 의해 억제될 수 있는 빈발하고 연장된 상태

③ 사회적 일을 심하게 방해하는 지속적인 불편

**8) 트림(eructation)**

위 내용물이 입으로 올라오는 반추, 구토와 달리 주로 가스만 입으로 배출 됩니다.

⓪ 없거나 순간적인 트림

① 수시로 나타나는 성가신 트림

② 몇몇 사회 활동을 방해하는 빈발하는 상태

③ 사회적 일을 심각하게 방해하는 빈발하는 상태

**(EQ-5D)**

다음 글을 읽어보시고, 각 번호의 문항 중 자신의 상태를 가장 잘 나타냈다고 생각되는 문항을 하나를 골라 해당하는 번호에 **∨** 표시하세요.

**1. 운동능력**

① 나는 걷는데 지장이 없다

② 나는 걷는데 다소 문제가 있다

③ 나는 종일 누워있어야 한다

**2. 자기관리**

① 나는 목욕을 하거나 옷을 입는데 지장이 없다

② 나는 혼자 목욕을 하거나 옷을 입는데 다소 지장이 있다

③ 나는 혼자 목욕을 하거나 옷을 입을 수 없다

**3. 일상활동(일, 공부, 가사일, 가족 또는 여가 활동)**

① 나는 일상 활동을 하는데 지장이 없다

② 나는 일상 활동을 하는데 다소 지장이 있다

③ 나는 일상 활동을 할 수 없다

**4. 통증/불편감**

① 나는 통증이나 불편감이 없다

② 나는 다소 통증이나 불편감이 있다

③ 나는 매우 심한 통증이나 불편감이 있다

**5. 불안/우울**

① 나는 불안하거나 우울하지 않다

② 나는 다소 불안하거나 우울하다

③ 나는 매우 심하게 불안하거나 우울하다

**6. 귀하의 전반적인 건강상태를 평가하신다면 다음 중 어디에 해당합니까?**

매우 나쁨 매우 좋음

**긴 시간 동안 질문에 성실히 응답해 주신 것에 감사 드립니다.**

### Appendix 4. 시험대상자 일지

| 시험번호 | | 스크리닝번호 | 무작위배정번호 | 시험대상자 이니셜 |
| --- | --- | --- | --- | --- |
|  |  |  |  |  |
| Naxozol_P4_1 | | \| S \|  \|  \|  \| \| --- \| --- \| --- \| --- \| | \| R \|  \|  \|  \| \| --- \| --- \| --- \| --- \| | \|  \|  \|  \| \| --- \| --- \| --- \| |

보조약물 (한미 알마게이트500mg 정 ) 이나 구제약 (한미 써스펜 ER 650mg 정)을 복용하셨을 경우, 아래 사항을 모두 기재해 주십시오.

(페이지 ______)

| 약드신날짜  (예: 2014년 7월10일 → **1 4 0 7 1 0** 한 칸에 한 자씩 기입해 주십시오). | | **ⓛ보충약** | **②구제약** |
| --- | --- | --- | --- |
|  |  | **한미 알마게이트500mg 정**  **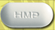** | **써스펜 이알 서방정 650mg**  **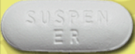** |
|  |  | 위장장애가 참을 수 없을 정도로 심한 경우 추가로 복용하시고, 하루 **최대 6정**을 넘게 드시지 마십시오. | 통증이 참을 수 없을 정도로 심한 경우에 추가로 복용하시고, 하루 **최대 4정**을 넘게 드시지 마십시오. |
| 1 | \|  \|  \|  \|  \|  \|  \| \| --- \| --- \| --- \| --- \| --- \| --- \| \| 년 \| 년 \| 월 \| 월 \| 일 \| 일 \| | \|  \| 정 \| \| --- \| --- \| | \|  \| 정 \| \| --- \| --- \| |
| 2 | \|  \|  \|  \|  \|  \|  \| \| --- \| --- \| --- \| --- \| --- \| --- \| \| 년 \| 년 \| 월 \| 월 \| 일 \| 일 \| | \|  \| 정 \| \| --- \| --- \| | \|  \| 정 \| \| --- \| --- \| |
| 3 | \|  \|  \|  \|  \|  \|  \| \| --- \| --- \| --- \| --- \| --- \| --- \| \| 년 \| 년 \| 월 \| 월 \| 일 \| 일 \| | \|  \| 정 \| \| --- \| --- \| | \|  \| 정 \| \| --- \| --- \| |
| 4 | \|  \|  \|  \|  \|  \|  \| \| --- \| --- \| --- \| --- \| --- \| --- \| \| 년 \| 년 \| 월 \| 월 \| 일 \| 일 \| | \|  \| 정 \| \| --- \| --- \| | \|  \| 정 \| \| --- \| --- \| |
| 5 | \|  \|  \|  \|  \|  \|  \| \| --- \| --- \| --- \| --- \| --- \| --- \| \| 년 \| 년 \| 월 \| 월 \| 일 \| 일 \| | \|  \| 정 \| \| --- \| --- \| | \|  \| 정 \| \| --- \| --- \| |
| 6 | \|  \|  \|  \|  \|  \|  \| \| --- \| --- \| --- \| --- \| --- \| --- \| \| 년 \| 년 \| 월 \| 월 \| 일 \| 일 \| | \|  \| 정 \| \| --- \| --- \| | \|  \| 정 \| \| --- \| --- \| |
| 7 | \|  \|  \|  \|  \|  \|  \| \| --- \| --- \| --- \| --- \| --- \| --- \| \| 년 \| 년 \| 월 \| 월 \| 일 \| 일 \| | \|  \| 정 \| \| --- \| --- \| | \|  \| 정 \| \| --- \| --- \| |
| 8 | \|  \|  \|  \|  \|  \|  \| \| --- \| --- \| --- \| --- \| --- \| --- \| \| 년 \| 년 \| 월 \| 월 \| 일 \| 일 \| | \|  \| 정 \| \| --- \| --- \| | \|  \| 정 \| \| --- \| --- \| |
| 9 | \|  \|  \|  \|  \|  \|  \| \| --- \| --- \| --- \| --- \| --- \| --- \| \| 년 \| 년 \| 월 \| 월 \| 일 \| 일 \| | \|  \| 정 \| \| --- \| --- \| | \|  \| 정 \| \| --- \| --- \| |
| 10 | \|  \|  \|  \|  \|  \|  \| \| --- \| --- \| --- \| --- \| --- \| --- \| \| 년 \| 년 \| 월 \| 월 \| 일 \| 일 \| | \|  \| 정 \| \| --- \| --- \| | \|  \| 정 \| \| --- \| --- \| |

### Appendix 5. 서명양식

### 의뢰자 서명

서명

임상시험 계획서 승인

임상 시험번호: Naxozol_P4_1

버전 번호: Version 1.2

본 임상시험 계획을 승인하였음을 확인합니다.

| Study Principal Investigator | |  |  |
| --- | --- | --- | --- |
| (서명, 상단) |  |  | 서명일 |
| 성명, 학위 | 문성환, M.D., PhD. | | |
| 직책 | 교수 | | |
| 소속기관 | 세브란스병원 정형외과 | | |
| 주소 | 서울시 서대문구 연세로 50-1 | | |

### 시험책임자 서명

서명

임상 시험번호: Naxozol_P4_1

버전 번호: Version 1.2

본 임상시험 계획서, 임상시험 관리 규정, 기타 해당 법률에 따라 임상시험을 진행할 것을 확인합니다.

Principal Investigator

|  |  |  |  |
| --- | --- | --- | --- |
| (서명: 상단) |  |  | 서명일 |
| 성명, 학위 |  | | |
| 직책 |  | | |
| 소속기관 |  | | |
| 주소 |  | | |

### Appendix 6. 피해자 보상 규약

**피해자 보상에 대한 규약**

1. 보상 원칙

1. 임상시험대상자의 신체적인 손상(사망 포함)에 대해 보상한다.
2. 임상시험과 관련하여 손상이 발생하였을 때 보상 보험액 한도 내에서 임상시험대상자에게 치료비를 지급한다.
3. 임상시험과 관련하여 발생한 이상반응의 교정처치 과정에서 발생한 손상의 경우, 치료비를 지급한다.

2. 비보상 원칙

1. 임상시험용 의약품과 관련되지 않은 의약품으로 인해 발생한 이상반응에 의한 손상
2. 임상시험용 의약품의 적응증에 대한 효과 또는 혜택을 제공하지 못한데 대한 보상
3. 서로 합의한 임상시험계획서에서 이탈함으로써 야기된 손상
4. 임상시험대상자 또는 보호자의 부주의에 의하여 초래된 손상 (예: 임상시험대상자의 부주의로 일어난 교통사고)
5. 임상시험대상자의 고의 또는 중대한 과실로 확대된 손해
6. 시험자의 명백한 과실이나 의무태만에 기인한 손상

3. 보상평가 기준

1. 보상수준은 손상의 본질, 그 정도, 지속성 여부 등에 적절한 액수여야 하며, 한국의 법정에 의해 유사 손상들에 대해 일반적으로 지급되는 것과 동일해야 한다.
2. 보상수준에 대해서 임상시험대상자의 대리인과 임상시험 의뢰자 사이에 이견이 있을 경우, 양자가 수용할 수 있는 전문가의 자문을 구하여야 한다.

본 임상시험 의뢰자는 앞에서 언급한 제반 내용을 참고하여, 임상시험대상자가 본 임상시험에 참여함으로써 어떠한 불이익이라도 받지 않도록 주의하며, 만약 본 임상시험에 의해 문제점이 발생할 경우 본 피해자 보상에 대한 규약에 의거하여 책임을 질 것을 서약합니다.

2014 년 10월 20일

임상시험 의뢰자 세브란스병원 정형외과 교수 문 성 환 (인)

# Attachments 별첨: Documents Controlled & Filed Seperately 별도 관리되는 문서

## 임상시험 실시기관, 시험책임자/담당자/관리약사 및 의뢰자/수탁기관

별도 파일로 관리(별첨1)

- 임상시험 실시기관 목록
- 시험책임자 성명 및 직명 목록
- 시험담당자 성명 및 직명 목록
- 관리 약사 성명 목록
- 의뢰자의 연락처
- 임상시험수탁기관의 연락처

## 시험대상자 동의를 위한 설명문 및 동의서

별도 파일로 관리(별첨2)
